# Supplementary material for: How important are concurrent vehicle control groups in (sub)chronic non-human primate toxicity studies conducted in pharmaceutical development? An opportunity to reduce animal numbers
Source: PLoS One. 2023 Aug 3;18(8):e0282404. doi: 10.1371/journal.pone.0282404 (PMC10399820; doi:10.1371/journal.pone.0282404)
Supplement: S1 Table — (PDF) [file pone.0282404.s001.pdf]

Data in Support of Table 2: Study A, Body temperature

Body Temperature

[redacted]

Group/ Sex	Phase / Day	Predose 8	TEMPR °C Dosing 176	Recovery 90
1/M	Mean	38.7	38.7	38.7
	SD	0.44	0.40	0.64
	N	6	6	2
2/M	Mean	38.3	38.3	-
	SD	0.30	0.31	-
	N	4	4	-
3/M	Mean	38.2	39.2	-
	SD	0.19	0.45	-
	N	4	4	-
4/M	Mean	38.7	38.6	39.0
	SD	0.44	0.39	0.21
	N	6	6	2
	Statistics	A	A	X7

A = ANOVA and Dunnett's
X7 = Not analyzed (mean of actual group sizes too small)

Table
Summary of Physical Examinations
Body Temperature

[redacted]

Group/ Sex	Phase / Day	Predose 9	TEMPR °C Dosing 176	Recovery 85
1/F	Mean	38.4	38.0	38.9
	SD	0.52	0.60	0.64
	N	6	6	2
2/F	Mean	38.7	38.9*	-
	SD	0.77	0.49	-
	N	4	4	-
3/F	Mean	38.2	39.0**	-
	SD	0.38	0.29	-
	N	4	4	-
4/F	Mean	38.3	39.5***	39.4
	SD	0.46	0.26	0.28
	N	6	6	2
	Statistics	A	A	X7

* P<=0.05
** P<=0.01
*** P<=0.001
A = ANOVA and Dunnett's
X7 = Not analyzed (mean of actual group sizes too small)

Data in Support of Table 2: Study A, Serum triglyceride

Table
Summary of Clinical Chemistry

[redacted]

TRIG. mmol/L

Group/ Sex	Phase / Day / Session	Predose 1 / 2	Predose 8 / 2	Dosing 90 / 2	Dosing 181 / 2	Recovery 88 / 2
1/M	Mean	0.28	0.33	0.66	0.50	0.35
	SD	0.114	0.078	0.199	0.132	0.198
	N	6	6	6	6	2
2/M	Mean	0.33	0.31	0.68	0.31*	-
	SD	0.042	0.071	0.311	0.077	-
	N	4	4	4	4	-
3/M	Mean	0.30	0.27	0.63	0.41	-
	SD	0.043	0.052	0.386	0.070	-
	N	4	4	4	4	-
4/M	Mean	0.31	0.36	1.30	0.88*	0.47
	SD	0.078	0.068	0.694	0.294	0.092
	N	6	6	6	6	2
	Statistics	A	A	A	AT	X7

* P<=0.05
** P<=0.01
*** P<=0.001
A = ANOVA and Dunnett's
T = Rank-transformed data

X7 = Not analyzed (mean of actual group sizes too small)

Table
Summary of Clinical Chemistry

[redacted]

		TRIG. mmol/L				
	Phase	Predose		Dosing		Recovery
Group/ Sex	Day Session	1 2	9 2	90 2	182 2	88 2
1/F	Mean	0.51	0.41	0.57	0.54	0.49
	SD	0.213	0.172	0.119	0.170	0.212
	N	6	6	6	6	2
2/F	Mean	0.35	0.34	0.51	0.50	-
	SD	0.084	0.114	0.342	0.135	-
	N	4	4	4	4	-
3/F	Mean	0.48	0.41	0.55	0.68	-
	SD	0.132	0.188	0.184	0.271	-
	N	4	4	4	4	-
4/F	Mean	0.40	0.41	0.56	0.50	0.74
	SD	0.136	0.207	0.358	0.140	0.346
	N	6	6	6	6	2
	Statistics	A	A	A	A	X7

A = ANOVA and Dunnett's
X7 = Not analyzed (mean of actual group sizes too small)

Data in Support of Table 2: Study B, Clinical obs (skin)

[redacted]

Test Item (dosage) 1 2 3

[redacted] --- [redacted] --- [redacted]

Group/ Sex	Animal Number	Observation	Phase	Day(s)
1/M	P0001	Desquamation		
		0: no desquamation	DSNG	1,8,15,22,29,36,43,50,57,64, 71,78,85,92,99,106,113,120, 127,134,141,148,155,162,169, 176,183
		Erythema		
		0: no erythema	DSNG	1,8,15,22,29,36,43,50,57,64, 71,78,85,92,99,106,113,120, 127,134,141,148,155,162,169, 176,183
		Eschar formation		
		no	DSNG	1,8,15,22,29,36,43,50,57,64, 71,78,85,92,99,106,113,120, 127,134,141,148,155,162,169, 176,183
		Fissures		
		0: none	DSNG	1,8,15,22,29,36,43,50,57,64, 71,78,85,92,99,106,113,120, 127,134,141,148,155,162,169, 176,183
		Oedema		
		0: no oedema	DSNG	1,8,15,22,29,36,43,50,57,64, 71,78,85,92,99,106,113,120, 127,134,141,148,155,162,169, 176,183

[redacted]

Table
Individual Clinical Observations
Test Item (dosage) 1 2 3

[redacted] --- [redacted] --- [redacted]

Group/ Sex	Animal Number	Observation	Phase	Day(s)
1/M	P0001	Ulcer		
		no	DSNG	1,8,15,22,29,36,43,50,57,64, 71,78,85,92,99,106,113,120, 127,134,141,148,155,162,169, 176,183

[redacted]

Table
Individual Clinical Observations
Test Item (dosage) 1 2 3

[redacted] --- [redacted] --- [redacted]

Group/ Sex	Animal Number	Observation	Phase	Day(s)
1/M	P0002	Desquamation		
		0: no desquamation	DSNG	1,8,15,22,29,36,43,50,57,64, 71,78,85,92,99,106,113,120, 127,134,141,148,155,162,169, 176,183
		Erythema		
		0: no erythema	DSNG	1,8,15,22,29,36,43,50,57,64, 71,78,85,92,99,106,113,120, 127,134,141,148,155,162,169, 176,183
		Eschar formation		
		no	DSNG	1,8,15,22,29,36,43,50,57,64, 71,78,85,92,99,106,113,120, 127,134,141,148,155,162,169, 176,183
		Fissures		
		0: none	DSNG	1,8,15,22,29,36,43,50,57,64, 71,78,85,92,99,106,113,120, 127,134,141,148,155,162,169, 176,183
		Oedema		
		0: no oedema	DSNG	1,8,15,22,29,36,43,50,57,64, 71,78,85,92,99,106,113,120, 127,134,141,148,155,162,169, 176,183

[redacted]

Table
Individual Clinical Observations
Test Item (dosage) 1 2 3

[redacted] --- [redacted] --- [redacted]

Group/ Sex	Animal Number	Observation	Phase	Day(s)
1/M	P0002	Ulcer		
		no	DSNG	1,8,15,22,29,36,43,50,57,64, 71,78,85,92,99,106,113,120, 127,134,141,148,155,162,169, 176,183

[redacted]

Table
Individual Clinical Observations
Test Item (dosage) 1 2 3

[redacted] --- [redacted] --- [redacted]

Group/ Sex	Animal Number	Observation	Phase	Day(s)
1/M	P0003	Desquamation		
		0: no desquamation	DSNG	1,8,15,22,29,36,43,50,57,64, 71,78,85,92,99,106,113,120, 127,134,141,148,155,157,162, 169,176,183
		Erythema		
		0: no erythema	DSNG	1,8,15,22,29,36,43,50,57,64, 71,78,85,92,99,106,113,120, 127,134,141,148,155,157,162, 169,176,183
		Eschar formation		
		no	DSNG	1,8,15,22,29,36,43,50,57,64, 71,78,85,92,99,106,113,120, 127,134,141,148,155,157,162, 169,176,183
		Fissures		
		0: none	DSNG	1,8,15,22,29,36,43,50,57,64, 71,78,85,92,99,106,113,120, 127,134,141,148,155,157,162, 169,176,183
		Oedema		
		0: no oedema	DSNG	1,8,15,22,29,36,43,50,57,64, 71,78,85,92,99,106,113,120, 127,134,141,148,157,162,169, 176,183

[redacted]

Table
Individual Clinical Observations
Test Item (dosage) 1 2 3

[redacted] --- [redacted] --- [redacted]

Group/ Sex	Animal Number	Observation	Phase	Day(s)
1/M	P0003	Ulcer		
		no	DSNG	1,8,15,22,29,36,43,50,57,64, 71,78,85,92,99,106,113,120, 127,134,141,148,155,157,162, 169,176,183
		Oedema		
		1: slight oedema	DSNG	155

[redacted]

Table
Individual Clinical Observations
Test Item (dosage) 1 2 3

[redacted] --- [redacted] --- [redacted]

Group/ Sex	Animal Number	Observation	Phase	Day(s)
2/M	P0101	Desquamation		
		0: no desquamation	DSNG	1,8,15,22,23,29,36,43,50,57, 64,71,78,85,92,99,106,113, 121,127,134,141,148,155,162, 169,176,183
		Erythema		
		0: no erythema	DSNG	1,8,15,23,29,36,43,50,57,64, 71,78,85,92,99,106,113,120, 121,127,134,141,148,155,162, 169,176,183
		Eschar formation		
		no	DSNG	1,8,15,22,23,29,36,43,50,57, 64,71,78,85,92,99,106,113, 120,121,127,134,141,148,155, 162,169,176,183
		Fissures		
		0: none	DSNG	1,8,15,22,23,29,36,43,50,57, 64,71,78,85,92,99,106,113, 120,121,127,134,141,148,155, 162,169,176,183
		Oedema		
		0: no oedema	DSNG	1,8,15,23,29,36,43,50,57,64, 71,78,85,92,99,106,113,121, 127,134,141,148,155,162,169, 176,183

[redacted]

Table
Individual Clinical Observations
Test Item (dosage) 1 2 3

[redacted] --- [redacted] --- [redacted]

Group/ Sex	Animal Number	Observation	Phase	Day(s)
2/M	P0101	Ulcer		
		no	DSNG	1,8,15,22,23,29,36,43,50,57, 64,71,78,85,92,99,106,113, 120,121,127,134,141,148,155, 162,169,176,183
		Erythema		
		2: moderate erythema, left leg	DSNG	22
		Oedema		
		2: moderate oedema, left leg	DSNG	22
		0: no oedema, right leg	DSNG	120
		3: severe oedema, left leg	DSNG	120

[redacted]

Table
Individual Clinical Observations
Test Item (dosage) 1 2 3

[redacted] --- [redacted] --- [redacted]

Group/ Sex	Animal Number	Observation	Phase	Day(s)
2/M	P0102	Desquamation		
		0: no desquamation	DSNG	1,8,15,22,29,36,43,50,57,64, 71,78,85,92,99,106,113,120, 127,134,141,148,155,162,169, 176,183
		Erythema		
		0: no erythema	DSNG	1,8,15,22,29,36,43,50,57,64, 71,78,85,92,99,106,113,120, 127,134,141,148,155,162,169, 176,183
		Eschar formation		
		no	DSNG	1,8,15,22,29,36,43,50,57,64, 71,78,85,92,99,106,113,120, 127,134,141,148,155,162,169, 176,183
		Fissures		
		0: none	DSNG	1,8,15,22,29,36,43,50,57,64, 71,78,85,92,99,106,113,120, 127,134,141,148,155,162,169, 176,183
		Oedema		
		0: no oedema	DSNG	1,8,15,22,29,36,43,50,57,64, 71,78,85,92,99,106,113,120, 127,134,141,148,155,162,169, 176,183

[redacted]

Table
Individual Clinical Observations
Test Item (dosage) 1 2 3

[redacted] --- [redacted] --- [redacted]

Group/ Sex	Animal Number	Observation	Phase	Day(s)
2/M	P0102	Ulcer		
		no	DSNG	1,8,15,22,29,36,43,50,57,64, 71,78,85,92,99,106,113,120, 127,134,141,148,155,162,169, 176,183

[redacted]

Table
Individual Clinical Observations
Test Item (dosage) 1 2 3

[redacted] --- [redacted] --- [redacted]

Group/ Sex	Animal Number	Observation	Phase	Day(s)
2/M	P0103	Desquamation		
		0: no desquamation	DSNG	1,8,15,22,29,36,43,50,57,64, 71,78,85,92,99,106,113,120, 127,134,141,148,155,162,169, 176,183
		Erythema		
		0: no erythema	DSNG	1,8,15,22,29,36,43,50,57,64, 71,78,85,92,99,106,113,120, 127,134,141,148,155,162,169, 176,183
		Eschar formation		
		no	DSNG	1,8,15,22,29,36,43,50,57,64, 71,78,85,92,99,106,113,120, 127,134,141,148,155,162,169, 176,183
		Fissures		
		0: none	DSNG	1,8,15,22,29,36,43,50,57,64, 71,78,85,92,99,106,113,120, 127,134,141,148,155,162,169, 176,183
		Oedema		
		0: no oedema	DSNG	1,8,15,22,29,36,43,50,57,64, 71,78,85,92,99,106,113,120, 127,134,141,148,155,162,169, 176,183

[redacted]

Table
Individual Clinical Observations
Test Item (dosage) 1 2 3

[redacted] --- [redacted] --- [redacted]

Group/ Sex	Animal Number	Observation	Phase	Day(s)
2/M	P0103	Ulcer		
		no	DSNG	1,8,15,22,29,36,43,50,57,64, 71,78,85,92,99,106,113,120, 127,134,141,148,155,162,169, 176,183

[redacted]

Table
Individual Clinical Observations
Test Item (dosage) 1 2 3
[redacted] --- [redacted] --- [redacted]

Group/ Sex	Animal Number	Observation	Phase	Day(s)
3/M	P0201	Desquamation		
		0: no desquamation	DSNG	1,8,15,22,29,36,43,50,57,64, 71,78,85,92,99,106,113,120, 127,134,141,148,155,162,169, 176,183
		Erythema		
		0: no erythema	DSNG	1,8,15,22,29,36,43,50,57,64, 71,78,80,81,85,92,99,106, 113,120,127,134,141,148,155, 162,169,176,183
		Eschar formation		
		no	DSNG	1,8,15,22,29,36,43,50,57,64, 71,78,85,92,99,106,113,120, 127,134,141,148,155,162,169, 176,183
		Fissures		
		0: none	DSNG	1,8,15,22,29,36,43,50,57,64, 71,78,85,92,99,106,113,120, 127,134,141,148,155,162,169, 176,183
		Oedema		
		0: no oedema	DSNG	1,8,15,22,29,36,43,50,57,64, 71,81,85,92,99,106,113,120, 127,134,141,148,155,162,169, 176,183

[redacted]

Table
Individual Clinical Observations
Test Item (dosage) 1 2 3

[redacted] --- [redacted] --- [redacted]

Group/ Sex	Animal Number	Observation	Phase	Day(s)
3/M	P0201	Ulcer		
		no	DSNG	1,8,15,22,29,36,43,50,57,64, 71,78,85,92,99,106,113,120, 127,134,141,148,155,162,169, 176,183
		Oedema		
		2: moderate oedema, left leg	DSNG	78-80
		Desquamation		
		0: no desquamation, left leg	DSNG	79-81
		Erythema		
		1: slight erythema, left leg	DSNG	79
		Eschar formation		
		no, left leg	DSNG	79-81
		Fissures		
		0: none, left leg	DSNG	79-81
		Ulcer		
		no, left leg	DSNG	79-81

Table
Individual Clinical Observations
Test Item (dosage) 1 2 3
[redacted]

Group/ Sex	Animal Number	Observation	Phase	Day(s)
3/M	P0202	Desquamation		
		0: no desquamation	DSNG	1,8,15,22,29,36,43,50,57,64, 71,78,85-89,92,99,106,113, 120,127,134,141,148,155,162, 169,176,183
		Erythema		
		0: no erythema	DSNG	1,8,15,22,29,36,43,50,57,64, 71,78,85,89,92,99,106,113, 120,127,134,141,148,155,162, 169,176,183
		Eschar formation		
		no	DSNG	1,8,15,22,29,36,43,50,57,64, 71,78,85-89,92,99,106,113, 120,127,134,141,148,155,162, 169,176,183
		Fissures		
		0: none	DSNG	1,8,15,22,29,36,43,50,57,64, 71,78,85-89,92,99,106,113, 120,127,134,141,148,155,162, 169,176,183
		Oedema		
		0: no oedema	DSNG	1,8,15,22,29,36,43,50,57,64, 71,78,87-89,92,99,106,113, 120,127,134,141,148,155,162, 169,176,183

[redacted]

Table
Individual Clinical Observations
Test Item (dosage) 1 2 3

[redacted] --- [redacted] --- [redacted]

Group/ Sex	Animal Number	Observation	Phase	Day(s)
3/M	P0202	Ulcer		
		no	DSNG	1,8,15,22,29,36,43,50,57,64, 71,78,85-89,92,99,106,113, 120,127,134,141,148,155,162, 169,176,183
		Oedema		
		1: slight oedema, left leg	DSNG	85,86
		Erythema		
		2: moderate erythema, left leg	DSNG	86-88

[redacted]

Table
Individual Clinical Observations
Test Item (dosage) 1 2 3
[redacted] --- [redacted] --- [redacted]

Group/ Sex	Animal Number	Observation	Phase	Day(s)
3/M	P0203	Desquamation		
		0: no desquamation	DSNG	1,8,15-20,22,29,36,43,50,57, 64,71,78,85,92,99,106,113, 120,127,134,141,148,155,162, 169,176,183
		Erythema		
		0: no erythema	DSNG	1,8,20,22,29,36,43,50,57,64, 71,78,85,92,99,106,113,120, 127,134,141,148,155,162,169, 176,183
		Eschar formation		
		no	DSNG	1,8,15-20,22,29,36,43,50,57, 64,71,78,85,92,99,106,113, 120,127,134,141,148,155,162, 169,176,183
		Fissures		
		0: none	DSNG	1,8,15-20,22,29,36,43,50,57, 64,71,78,85,92,99,106,113, 120,127,134,141,148,155,162, 169,176,183
		Oedema		
		0: no oedema	DSNG	1,8,20,22,29,36,43,50,57,64, 71,78,85,92,99,106,113,120, 127,134,141,148,155,162,169, 176,183

[redacted]

Table
Individual Clinical Observations
Test Item (dosage) 1 2 3

[redacted] --- [redacted] --- [redacted]

Group/ Sex	Animal Number	Observation	Phase	Day(s)
3/M	P0203	Ulcer		
		no	DSNG	1,8,15-20,22,29,36,43,50,57, 64,71,78,85,92,99,106,113, 120,127,134,141,148,155,162, 169,176,183
		Erythema		
		2: moderate erythema	DSNG	15
		Oedema		
		3: severe oedema	DSNG	15
		Erythema		
		2: moderate erythema, left leg	DSNG	16-18
		Oedema		
		3: severe oedema, left leg	DSNG	16
		1: slight oedema, left leg	DSNG	17-19
		Erythema		
		1: slight erythema, left leg	DSNG	19

[redacted]

Table
Individual Clinical Observations
Test Item (dosage) 1 2 3

[redacted] --- [redacted] --- [redacted]

Group/ Sex	Animal Number	Observation	Phase	Day(s)
1/F	P0301	Desquamation		
		0: no desquamation	DSNG	1,8,15,22,29,36,43,50,57,64, 71,78,85,92,99,106,113,120, 127,134,141,148,155,162,169, 176,183
		Erythema		
		0: no erythema	DSNG	1,8,15,22,29,36,43,50,57,64, 71,78,85,92,99,106,113,120, 127,134,141,148,155,162,169, 176,183
		Eschar formation		
		no	DSNG	1,8,15,22,29,36,43,50,57,64, 71,78,85,92,99,106,113,120, 127,134,141,148,155,162,169, 176,183
		Fissures		
		0: none	DSNG	1,8,15,22,29,36,43,50,57,64, 71,78,85,92,99,106,113,120, 127,134,141,148,155,162,169, 176,183
		Oedema		
		0: no oedema	DSNG	1,8,15,22,29,36,43,50,57,64, 71,78,85,92,99,106,113,120, 127,134,141,148,155,162,169, 176,183

[redacted]

Table
Individual Clinical Observations
Test Item (dosage) 1 2 3

[redacted] --- [redacted] --- [redacted]

Group/ Sex	Animal Number	Observation	Phase	Day(s)
1/F	P0301	Ulcer		
		no	DSNG	1,8,15,22,29,36,43,50,57,64, 71,78,85,92,99,106,113,120, 127,134,141,148,155,162,169, 176,183

[redacted]

Table
Individual Clinical Observations
Test Item (dosage) 1 2 3

[redacted] --- [redacted] --- [redacted]

Group/ Sex	Animal Number	Observation	Phase	Day(s)
1/F	P0302	Desquamation		
		0: no desquamation	DSNG	1,8,15,22,29,36,43,50,57,64, 71,78,85,92,99,106,113,120, 127,134,141,148,155,162,169, 176,183
		Erythema		
		0: no erythema	DSNG	1,8,15,22,29,36,43,50,57,64, 71,78,85,92,99,106,113,120, 127,134,141,148,155,162,169, 176,183
		Eschar formation		
		no	DSNG	1,8,15,22,29,36,43,50,57,64, 71,78,85,92,99,106,113,120, 127,134,141,148,155,162,169, 176,183
		Fissures		
		0: none	DSNG	1,8,15,22,29,36,43,50,57,64, 71,78,85,92,99,106,113,120, 127,134,141,148,155,162,169, 176,183
		Oedema		
		0: no oedema	DSNG	1,8,15,22,29,36,43,50,57,64, 71,78,85,92,99,106,113,120, 127,134,141,148,155,162,169, 176,183

[redacted]

Table
Individual Clinical Observations
Test Item (dosage) 1 2 3

[redacted] --- [redacted] --- [redacted]

Group/ Sex	Animal Number	Observation	Phase	Day(s)
1/F	P0302	Ulcer		
		no	DSNG	1,8,15,22,29,36,43,50,57,64, 71,78,85,92,99,106,113,120, 127,134,141,148,155,162,169, 176,183

[redacted]

Table
Individual Clinical Observations
Test Item (dosage) 1 2 3

[redacted] --- [redacted] --- [redacted]

Group/ Sex	Animal Number	Observation	Phase	Day(s)
1/F	P0303	Desquamation		
		0: no desquamation	DSNG	1,8,15,22-25,29,36,43,50,57, 64,71,78,85,92,99,106,113, 120,127,134,141,148,155,162, 169,176,183
		Erythema		
		0: no erythema	DSNG	1,8,15,25,29,36,43,50,57,64, 71,78,85,92,99,106,113,120, 127,134,141,148,155,162,169, 176,183
		Eschar formation		
		no	DSNG	1,8,15,22-25,29,36,43,50,57, 64,71,78,85,92,99,106,113, 120,127,134,141,148,155,162, 169,176,183
		Fissures		
		0: none	DSNG	1,8,15,22-25,29,36,43,50,57, 64,71,78,85,92,99,106,113, 120,127,134,141,148,155,162, 169,176,183
		Oedema		
		0: no oedema	DSNG	1,8,15,25,29,36,43,50,57,64, 71,78,85,92,99,106,113,120, 127,134,141,148,155,162,169, 176,183

[redacted]

Table
Individual Clinical Observations
Test Item (dosage) 1 2 3

[redacted] --- [redacted] --- [redacted]

Group/ Sex	Animal Number	Observation	Phase	Day(s)
1/F	P0303	Ulcer		
		no	DSNG	1,8,15,22-25,29,36,43,50,57, 64,71,78,85,92,99,106,113, 120,127,134,141,148,155,162, 169,176,183
		Erythema		
		2: moderate erythema, left leg	DSNG	22
		Oedema		
		2: moderate oedema, left leg	DSNG	22
		Erythema		
		1: slight erythema, left leg	DSNG	23,24
		Oedema		
		3: severe oedema, left leg	DSNG	23,24

[redacted]

Table
Individual Clinical Observations
Test Item (dosage) 1 2 3

[redacted] --- [redacted] --- [redacted]

Group/ Sex	Animal Number	Observation	Phase	Day(s)
2/F	P0401	Desquamation		
		0: no desquamation	DSNG	1,8-12,15,22,29,36-39,43,50, 57,64,71,78,85,92,99,106, 113,120,127,134,141,148,155, 162,169,176,183
		Erythema		
		0: no erythema	DSNG	1,12,15,22,29,43,50,57,64, 71,78,85,92,99,106,113,120, 127,134,141,148,155,162,169, 176,183
		Eschar formation		
		no	DSNG	1,8-12,15,22,29,36-39,43,50, 57,64,71,78,85,92,99,106, 113,120,127,134,141,148,155, 162,169,176,183
		Fissures		
		0: none	DSNG	1,8-12,15,22,29,36-39,43,50, 57,64,71,78,85,92,99,106, 113,120,127,134,141,148,155, 162,169,176,183
		Oedema		
		0: no oedema	DSNG	1,8-12,15,22,29,36-39,43,50, 57,64,71,78,85,92,99,106, 113,120,127,134,141,148,155, 162,169,176,183

[redacted]

Table
Individual Clinical Observations
Test Item (dosage) 1 2 3

[redacted] --- [redacted] --- [redacted]

Group/ Sex	Animal Number	Observation	Phase	Day(s)
2/F	P0401	Ulcer		
		no	DSNG	1,8-12,15,22,29,36-39,43,50, 57,64,71,78,85,92,99,106, 113,120,127,134,141,148,155, 162,169,176,183
		Erythema		
		2: moderate erythema, left leg, postdosing (1.observation)	DSNG	8
		2: moderate erythema, left leg	DSNG	9-11
		1: slight erythema, left leg	DSNG	36-38
		0: no erythema, left leg	DSNG	39

[redacted]

Table
Individual Clinical Observations
Test Item (dosage) 1 2 3

[redacted] --- [redacted] --- [redacted]

Group/ Sex	Animal Number	Observation	Phase	Day(s)
2/F	P0402	Desquamation		
		0: no desquamation	DSNG	1,8-15,22,29,36,43,50,57,64, 71,78,85,92,99,106,113,120, 127,134,141,148,155,162,169, 176,183
		Erythema		
		0: no erythema	DSNG	1,14,15,22,29,36,43,50,57, 64,71,78,85,92,99,106,113, 120,127,134,141,148,155,162, 169,176,183
		Eschar formation		
		no	DSNG	1,8-15,22,29,36,43,50,57,64, 71,78,85,92,99,106,113,120, 127,134,141,148,155,162,169, 176,183
		Fissures		
		0: none	DSNG	1,8-15,22,29,36,43,50,57,64, 71,78,85,92,99,106,113,120, 127,134,141,148,155,162,169, 176,183
		Oedema		
		0: no oedema	DSNG	1,8-15,22,29,36,43,50,57,64, 71,78,85,92,99,106,113,120, 127,134,141,148,155,162,169, 176,183

[redacted]

Table
Individual Clinical Observations
Test Item (dosage) 1 2 3

[redacted] [redacted] [redacted]

Group/ Sex	Animal Number	Observation	Phase	Day(s)
2/F	P0402	Ulcer		
		no	DSNG	1,8-15,22,29,36,43,50,57,64, 71,78,85,92,99,106,113,120, 127,134,141,148,155,162,169, 176,183
		Erythema		
		2: moderate erythema, left leg, postdosing (1.observation)	DSNG	8
		2: moderate erythema, left leg	DSNG	9-13

[redacted]

Table
Individual Clinical Observations
Test Item (dosage) 1 2 3

[redacted] --- [redacted] --- [redacted]

Group/ Sex	Animal Number	Observation	Phase	Day(s)
2/F	P0403	Desquamation		
		0: no desquamation	DSNG	1,8-12,15,22,29,36-43,50,57, 64,71,78,85,92,99,106,113, 120,127,134-141,148,155,162, 169,176,183
		Erythema		
		0: no erythema	DSNG	1,12,15,22,29,42,64,71,78, 85,92,99,106,113,120,127, 141,148,155,162,169,176,183
		Eschar formation		
		no	DSNG	1,8-12,15,22,29,36-43,50,57, 64,71,78,85,92,99,106,113, 120,127,134-141,148,155,162, 169,176,183
		Fissures		
		0: none	DSNG	1,8-12,15,22,29,36-43,50,57, 64,71,78,85,92,99,106,113, 120,127,134-141,148,155,162, 169,176,183
		Oedema		
		0: no oedema	DSNG	1,8-12,15,22,29,37-43,50,57, 64,71,78,85,92,99,106,113, 120,127,134-141,148,155,162, 169,176,183

[redacted]

Table
Individual Clinical Observations
Test Item (dosage) 1 2 3
[redacted] --- [redacted] --- [redacted]

Group/ Sex	Animal Number	Observation	Phase	Day(s)
2/F	P0403	Ulcer		
		no	DSNG	1,8-12,15,22,29,36-43,50,57, 64,71,78,85,92,99,106,113, 120,127,134-141,148,155,162, 169,176,183
		Erythema		
		2: moderate erythema, left leg, postdosing (1.observation)	DSNG	8
		2: moderate erythema, left leg	DSNG	9-11,38-41,137
		1: slight erythema, left leg	DSNG	36,37,134-136,138-140
		Oedema		
		2: moderate oedema, left leg	DSNG	36
		Erythema		
		0: no erythema, left leg	DSNG	43,50,57

[redacted]

Table
Individual Clinical Observations
Test Item (dosage) 1 2 3

[redacted] --- [redacted] --- [redacted]

Group/ Sex	Animal Number	Observation	Phase	Day(s)
3/F	P0501	Desquamation		
		0: no desquamation	DSNG	1,8-12,15,22,29,36,43,50,57, 64,71,78,85,92,99,106,113, 120,127,134,141,148,155,162, 169,176,183
		Erythema		
		0: no erythema	DSNG	1,12,15,22,29,36,43,50,57, 64,71,78,85,92,99,106,113, 120,127,134,141,148,155,162, 169,176,183
		Eschar formation		
		no	DSNG	1,8-12,15,22,29,36,43,50,57, 64,71,78,85,92,99,106,113, 120,127,134,141,148,155,162, 169,176,183
		Fissures		
		0: none	DSNG	1,8-12,15,22,29,36,43,50,57, 64,71,78,85,92,99,106,113, 120,127,134,141,148,155,162, 169,176,183
		Oedema		
		0: no oedema	DSNG	1,8-12,15,22,29,36,43,50,57, 64,71,78,85,92,99,106,113, 120,127,134,141,148,155,162, 169,176,183

[redacted]

Table
Individual Clinical Observations
Test Item (dosage) 1 2 3

[redacted] --- [redacted] --- [redacted]

Group/ Sex	Animal Number	Observation	Phase	Day(s)
3/F	P0501	Ulcer		
		no	DSNG	1,8-12,15,22,29,36,43,50,57, 64,71,78,85,92,99,106,113, 120,127,134,141,148,155,162, 169,176,183
		Erythema		
		2: moderate erythema, left leg, postdosing (1.observation)	DSNG	8
		2: moderate erythema, left leg	DSNG	9-11

[redacted]

Table
Individual Clinical Observations
Test Item (dosage) 1 2 3
[redacted]

Group/ Sex	Animal Number	Observation	Phase	Day(s)
3/F	P0502	Desquamation		
		0: no desquamation	DSNG	1,8,15,22,29,36,43,50,57,64, 71,78,85,92,99,106,113,120, 127,134,141,148,155,162,169, 170,176,183
		Erythema		
		0: no erythema	DSNG	1,8,15,22,29,36,43,50,57,64, 71,78,85,92,99,106,113,120, 127,134,141,148,155,162,169, 170,176,183
		Eschar formation		
		no	DSNG	1,8,15,22,29,36,43,50,57,64, 71,78,85,92,99,106,113,120, 127,134,141,148,155,162,169, 170,176,183
		Fissures		
		0: none	DSNG	1,8,15,22,29,36,43,50,57,64, 71,78,85,92,99,106,113,120, 127,134,141,148,155,162,169, 170,176,183
		Oedema		
		0: no oedema	DSNG	1,8,15,22,29,36,43,50,57,64, 71,78,85,92,99,106,113,120, 127,134,141,148,155,162,170, 176,183

[redacted]

Table
Individual Clinical Observations
Test Item (dosage) 1 2 3

[redacted] --- [redacted] --- [redacted]

Group/ Sex	Animal Number	Observation	Phase	Day(s)
3/F	P0502	Ulcer		
		no	DSNG	1,8,15,22,29,36,43,50,57,64, 71,78,85,92,99,106,113,120, 127,134,141,148,155,162,169, 170,176,183
		Oedema		
		3: severe oedema, right leg	DSNG	169

Table [redacted]
Individual Clinical Observations
Test Item (dosage) 1 2 3
[redacted] --- [redacted] --- [redacted]

Group/ Sex	Animal Number	Observation	Phase	Day(s)
3/F	P0503	Desquamation		
		0: no desquamation	DSNG	1,8,15-17,22,29,36,43,50,57, 64,71,78,85,92,99,106,113, 120,127,134,141,148,155,162, 169,176,183
		Erythema		
		0: no erythema	DSNG	1,8,22,29,36,43,50,57,64,71, 78,82,85,92,99,106,113,120, 127,134,141,148,155,162,169, 176,183
		Eschar formation		
		no	DSNG	1,8,15-17,22,29,36,43,50,57, 64,71,78,85,92,99,106,113, 120,127,134,141,148,155,162, 169,176,183
		Fissures		
		0: none	DSNG	1,8,15-17,22,29,36,43,50,57, 64,71,78,85,92,99,106,113, 120,127,134,141,148,155,162, 169,176,183
		Oedema		
		0: no oedema	DSNG	1,8,22,29,36,43,50,57,64,71, 85,92,99,106,113,120,127, 134,141,148,155,162,169,176, 183

Table
Individual Clinical Observations
Test Item (dosage) 1 2 3

[redacted] --- [redacted] --- [redacted]

Group/ Sex	Animal Number	Observation	Phase	Day(s)
3/F	P0503	Ulcer		
		no	DSNG	1,8,15-17,22,29,36,43,50,57, 64,71,78,85,92,99,106,113, 120,127,134,141,148,155,162, 169,176,183
		Erythema		
		1: slight erythema	DSNG	15
		Oedema		
		3: severe oedema	DSNG	15
		Erythema		
		2: moderate erythema, left leg	DSNG	16
		Oedema		
		2: moderate oedema, left leg	DSNG	16
		Erythema		
		0: no erythema, both legs	DSNG	17
		Oedema		
		0: no oedema, both legs	DSNG	17
		1: slight oedema, left leg	DSNG	78
		Desquamation		
		0: no desquamation, left leg	DSNG	79-82
		Erythema		
		1: slight erythema, left leg	DSNG	79-81
		Eschar formation		
		no, left leg	DSNG	79-82

[redacted]

Table
Individual Clinical Observations
Test Item (dosage) 1 2 3

[redacted] --- [redacted] --- [redacted]

Group/ Sex	Animal Number	Observation	Phase	Day(s)
3/F	P0503	Fissures		
		0: none, left leg	DSNG	79-82
		Oedema		
		0: no oedema, left leg	DSNG	79-82
		Ulcer		
		no, left leg	DSNG	79-82

Data in Support of Table 2: Study B, TDAR

Summary of Anti KLH, IgG - Males
Test Item (dosage) 1 2 3
[redacted]

Group/ Sex	Phase / Day	Predose 8	KLHG Dosing 32	39	46	53
1/M	Mean	415	390	5934	59863	62062
	SD	276.8	258.2	5562.4	92249.8	80445.8
	N	3	3	3	3	3
2/M	Mean	184	161	3131	2240	5142*
	SD	146.1	105.1	1996.2	301.8	2843.9
	N	3	3	3	3	3
3/M	Mean	316	204	1942	7830	6618*
	SD	373.5	179.6	89.4	1082.2	1710.7
	N	3	3	3	3	3
	Statistics	X1	A	A	AT	AT

* P<=0.05
** P<=0.01
*** P<=0.001
X1 = No analysis required
A = ANOVA and Dunnett's

T = Rank-transformed data

Summary of Anti KLH, IgG - Males
Test Item (dosage) 1 2 3
[redacted]

Group/ Sex	Phase Day	KLHG Dosing 60	67	74	81
1/M	Mean	66847	32534	19099	23491
	SD	100352.3	28964.5	13220.8	16764.8
	N	3	2	2	2
2/M	Mean	3548*	13065	17601	10468
	SD	2396.1	12759.5	15433.4	12665.4
	N	3	3	3	3
3/M	Mean	2590*	16831	32616	10193
	SD	317.7	10906.3	18432.5	2601.4
	N	3	3	3	3
	Statistics	AT	A	A	AT

* P<=0.05
** P<=0.01
*** P<=0.001
A = ANOVA and Dunnett's
T = Rank-transformed data

Summary of Anti KLH, IgG - Females

Test Item (dosage) 1 2 3

[redacted]

Group/ Sex	Phase / Day	Predose 8	KLHG Dosing 32	39	46	53
1/F	Mean	100	100	1860	8849	34893
	SD	0.0	0.0	362.4	3425.1	4979.9
	N	3	3	3	3	3
2/F	Mean	206	135	2100	8472	8849***
	SD	108.6	61.2	852.9	5305.1	3590.2
	N	3	3	3	3	3
3/F	Mean	253	289**	3710	5810	5386***
	SD	141.2	50.0	2714.6	3034.3	2788.9
	N	3	3	3	3	3
	Statistics	X1	AT	AT	A	A

* P<=0.05
** P<=0.01
*** P<=0.001
X1 = No analysis required
A = ANOVA and Dunnett's
T = Rank-transformed data

Summary of Anti KLH, IgG - Females
Test Item (dosage) 1 2 3
[redacted]

Group/Sex	Phase / Day	KLHG Dosing 60	67	74	81
1/F	Mean	18536	165127	118281	63311
	SD	7283.7	90816.3	56818.1	8954.7
	N	3	3	3	3
2/F	Mean	5244*	10277*	18435*	8202***
	SD	3249.8	5556.1	13652.9	3397.3
	N	3	3	3	3
3/F	Mean	2420**	15832*	15143*	6847***
	SD	191.5	16979.7	13572.1	4671.4
	N	3	3	3	3
	Statistics	A	AT	A	A

* P<=0.05
** P<=0.01
*** P<=0.001
A = ANOVA and Dunnett's
T = Rank-transformed data

Data in Support of Table 2: Study C, Clinical obs (injection site)

Test Item (dosage) 1 2 3 4

[redacted] --- [redacted]

Group/ Sex	Animal Number	Observation	Phase	Day(s)
1/M	P0001	Erythema		
		0: no erythema	DSNG	9,16,23,30,37,44,51,58,65, 72,79,86,93,100,107,114,121, 128,135,142,149,156,163,170, 177
		0: no erythema, field 1	DSNG	2
		Oedema		
		0: no oedema	DSNG	9,16,23,30,37,44,51,58,65, 72,79,86,93,100,107,114,121, 128,135,142,149,156,163,170, 177
		0: no oedema, field 1	DSNG	2
		Desquamation		
		0: no desquamation	DSNG	9,16,23,30,37,44,51,58,65, 72,79,86,93,100,107,114,121, 128,135,142,149,156,163,170, 177
		0: no desquamation, field 1	DSNG	2
		Fissures		
		0: none	DSNG	9,16,23,30,37,44,51,58,65, 72,79,86,93,100,107,114,121, 128,135,142,149,156,163,170, 177
		0: none, field 1	DSNG	2

Table
Individual Clinical Observations
Test Item (dosage) 1 2 3 4

[redacted] ----- [redacted]

Group/ Sex	Animal Number	Observation	Phase	Day(s)
1/M	P0001	Eschar formation		
		no	DSNG	9,16,23,30,37,44,51,58,65, 72,79,86,93,100,107,114,121, 128,135,142,149,156,163,170, 177
		no, field 1	DSNG	2
		Ulcer		
		no	DSNG	9,16,23,30,37,44,51,58,65, 72,79,86,93,100,107,114,121, 128,135,142,149,156,163,170, 177
		no, field 1	DSNG	2

Table
Individual Clinical Observations
Test Item (dosage) 1 2 3 4

[redacted] ----- [redacted] ---

Group/ Sex	Animal Number	Observation	Phase	Day(s)
1/M	P0002	Erythema		
		0: no erythema	DSNG	86,93,100,107,114,121,128, 135,142,149,156,163,170,177
		0: no erythema, field 1	DSNG	2,9,58,79
		Oedema		
		0: no oedema	DSNG	86,93,100,107,114,121,128, 135,142,149,156,163,170,177
		0: no oedema, field 1	DSNG	2,9,58,79
		Desquamation		
		0: no desquamation	DSNG	86,93,100,107,114,121,128, 135,142,149,156,163,170,177
		0: no desquamation, field 1	DSNG	2,9,58,79
		Fissures		
		0: none	DSNG	86,93,100,107,114,121,128, 135,142,149,156,163,170,177
		0: none, field 1	DSNG	2,9,58,79
		Eschar formation		
		no	DSNG	86,93,100,107,114,121,128, 135,142,149,156,163,170,177
		no, field 1	DSNG	2,9,58,79
		Ulcer		
		no	DSNG	86,93,100,107,114,121,128, 135,142,149,156,163,170,177
		no, field 1	DSNG	2,9,58,79

Table
Individual Clinical Observations
Test Item (dosage) 1 2 3 4

[redacted] [redacted]

Group/ Sex	Animal Number	Observation	Phase	Day(s)
1/M	P0003	Erythema		
		0: no erythema	DSNG	9,16,23,30,37,44,51,58,65, 72,79,86,93,100,107,114,121, 128,135,142,149,156,163,170, 177
		0: no erythema, field 1	DSNG	2
		Oedema		
		0: no oedema	DSNG	9,16,23,30,37,44,51,58,65, 72,79,86,93,100,107,114,121, 128,135,142,149,156,163,170, 177
		0: no oedema, field 1	DSNG	2
		Desquamation		
		0: no desquamation	DSNG	9,16,23,30,37,44,51,58,65, 72,79,86,93,100,107,114,121, 128,135,142,149,156,163,170, 177
		0: no desquamation, field 1	DSNG	2
		Fissures		
		0: none	DSNG	9,16,23,30,37,44,51,58,65, 72,79,86,93,100,107,114,121, 128,135,142,149,156,163,170, 177
		0: none, field 1	DSNG	2

Table
Individual Clinical Observations
Test Item (dosage) 1 2 3 4

[redacted] [redacted]

Group/ Sex	Animal Number	Observation	Phase	Day(s)
1/M	P0003	Eschar formation		
		no	DSNG	9,16,23,30,37,44,51,58,65, 72,79,86,93,100,107,114,121, 128,135,142,149,156,163,170, 177
		no, field 1	DSNG	2
		Ulcer		
		no	DSNG	9,16,23,30,37,44,51,58,65, 72,79,86,93,100,107,114,121, 128,135,142,149,156,163,170, 177
		no, field 1	DSNG	2

Table
Individual Clinical Observations
Test Item (dosage) 1 2 3 4

[redacted] ---- [redacted]

Group/ Sex	Animal Number	Observation	Phase	Day(s)
1/M	P0004	Erythema		
		0: no erythema	DSNG	9,16,23,30,37,44,51,58,65, 72,79,86,93,100,107,114,121, 128,135,142,149,156,163,170, 177
		0: no erythema, field 1	DSNG	2
		Oedema		
		0: no oedema	DSNG	9,16,23,30,37,44,51,58,65, 72,79,86,93,100,107,114,121, 128,135,142,149,156,163,170, 177
		0: no oedema, field 1	DSNG	2
		Desquamation		
		0: no desquamation	DSNG	9,16,23,30,37,44,51,58,65, 72,79,86,93,100,107,114,121, 128,135,142,149,156,163,170, 177
		0: no desquamation, field 1	DSNG	2
		Fissures		
		0: none	DSNG	9,16,23,30,37,44,51,58,65, 72,79,86,93,100,107,114,121, 128,135,142,149,156,163,170, 177
		0: none, field 1	DSNG	2

Table
Individual Clinical Observations
Test Item (dosage) 1 2 3 4

[redacted] ----- [redacted] --

Group/ Sex	Animal Number	Observation	Phase	Day(s)
1/M	P0004	Eschar formation		
		no	DSNG	9,16,23,30,37,44,51,58,65, 72,79,86,93,100,107,114,121, 128,135,142,149,156,163,170, 177
		no, field 1	DSNG	2
		Ulcer		
		no	DSNG	9,16,23,30,37,44,51,58,65, 72,79,86,93,100,107,114,121, 128,135,142,149,156,163,170, 177
		no, field 1	DSNG	2

Table
Individual Clinical Observations
Test Item (dosage) 1 2 3 4

[redacted] ----- [redacted]

Group/ Sex	Animal Number	Observation	Phase	Day(s)
1/M	P0005	Erythema		
		0: no erythema	DSNG	9,16,23,30,37,44,51,58,66, 72,73,79,86,93,100,107,114, 121,128,135,142,149,156,163, 170,177
		0: no erythema, field 1	DSNG	2
		2: moderate erythema, field 2, postdosing (2.observation)	DSNG	65
		Oedema		
		0: no oedema	DSNG	9,16,23,30,37,44,51,58,65, 66,72,73,79,86,93,100,107, 114,121,128,135,142,149,156, 163,170,177
		0: no oedema, field 1	DSNG	2
		Desquamation		
		0: no desquamation	DSNG	9,16,23,30,37,44,51,58,65, 66,72,73,79,86,93,100,107, 114,121,128,135,142,149,156, 163,170,177
		0: no desquamation, field 1	DSNG	2

Table
Individual Clinical Observations
Test Item (dosage) 1 2 3 4

[redacted] ----- [redacted] --

Group/ Sex	Animal Number	Observation	Phase	Day(s)
1/M	P0005	Fissures		
		0: none	DSNG	9,16,23,30,37,44,51,58,65, 66,72,73,79,86,93,100,107, 114,121,128,135,142,149,156, 163,170,177
		0: none, field 1	DSNG	2
		Eschar formation		
		no	DSNG	9,16,23,30,37,44,51,58,65, 66,72,73,79,86,93,100,107, 114,121,128,135,142,149,156, 163,170,177
		no, field 1	DSNG	2
		Ulcer		
		no	DSNG	9,16,23,30,37,44,51,58,65, 66,72,73,79,86,93,100,107, 114,121,128,135,142,149,156, 163,170,177
		no, field 1	DSNG	2

Table
Individual Clinical Observations
Test Item (dosage) 1 2 3 4

[redacted] ----- [redacted]

Group/ Sex	Animal Number	Observation	Phase	Day(s)
1/M	P0006	Erythema		
		0: no erythema	DSNG	9,16,23,30,37,44,51,58,65, 72,79,86,93,100,107,114,121, 128,135,142,149,156,163,170, 177
		0: no erythema, field 1	DSNG	2
		Oedema		
		0: no oedema	DSNG	9,16,23,30,37,44,51,58,65, 72,79,86,93,100,107,114,121, 128,135,142,149,156,163,170, 177
		0: no oedema, field 1	DSNG	2
		Desquamation		
		0: no desquamation	DSNG	9,16,23,30,37,44,51,58,65, 72,79,86,93,100,107,114,121, 128,135,142,149,156,163,170, 177
		0: no desquamation, field 1	DSNG	2
		Fissures		
		0: none	DSNG	9,16,23,30,37,44,51,58,65, 72,79,86,93,100,107,114,121, 128,135,142,149,156,163,170, 177
		0: none, field 1	DSNG	2

Table
Individual Clinical Observations
Test Item (dosage) 1 2 3 4

[redacted] ----- [redacted] ---

Group/ Sex	Animal Number	Observation	Phase	Day(s)
1/M	P0006	Eschar formation		
		no	DSNG	9,16,23,30,37,44,51,58,65, 72,79,86,93,100,107,114,121, 128,135,142,149,156,163,170, 177
		no, field 1	DSNG	2
		Ulcer		
		no	DSNG	9,16,23,30,37,44,51,58,65, 72,79,86,93,100,107,114,121, 128,135,142,149,156,163,170, 177
		no, field 1	DSNG	2

Table
Individual Clinical Observations
Test Item (dosage) 1 2 3 4

[redacted] ----[redacted]

Group/ Sex	Animal Number	Observation	Phase	Day(s)
2/M	P0101	Erythema		
		0: no erythema	DSNG	9,16,23,30,37,44,51,58,65, 72,79,86,93,100,107,114,121, 128,135,142,149,156,163,170, 177
		0: no erythema, field 1	DSNG	2
		Oedema		
		0: no oedema	DSNG	9,16,23,30,37,44,51,58,65, 72,79,86,93,100,107,114,121, 128,135,142,149,156,163,170, 177
		0: no oedema, field 1	DSNG	2
		Desquamation		
		0: no desquamation	DSNG	9,16,23,30,37,44,51,58,65, 72,79,86,93,100,107,114,121, 128,135,142,149,156,163,170, 177
		0: no desquamation, field 1	DSNG	2
		Fissures		
		0: none	DSNG	9,16,23,30,37,44,51,58,65, 72,79,86,93,100,107,114,121, 128,135,142,149,156,163,170, 177
		0: none, field 1	DSNG	2

Table
Individual Clinical Observations
Test Item (dosage) 1 2 3 4

[redacted] ----- [redacted]

Group/ Sex	Animal Number	Observation	Phase	Day(s)
2/M	P0101	Eschar formation		
		no	DSNG	9,16,23,30,37,44,51,58,65, 72,79,86,93,100,107,114,121, 128,135,142,149,156,163,170, 177
		no, field 1	DSNG	2
		Ulcer		
		no	DSNG	9,16,23,30,37,44,51,58,65, 72,79,86,93,100,107,114,121, 128,135,142,149,156,163,170, 177
		no, field 1	DSNG	2

Table
Individual Clinical Observations
Test Item (dosage) 1 2 3 4

[redacted] [redacted]

Group/ Sex	Animal Number	Observation	Phase	Day(s)
2/M	P0102	Erythema		
		0: no erythema	DSNG	9,16,23,30,37,44,51,58,65, 72,79,86,93,100,107,114,121, 128,135,142,149,156,163,170, 177
		0: no erythema, field 1	DSNG	2
		Oedema		
		0: no oedema	DSNG	9,16,23,30,37,44,51,58,65, 72,79,86,93,100,107,114,121, 128,135,142,149,156,163,170, 177
		0: no oedema, field 1	DSNG	2
		Desquamation		
		0: no desquamation	DSNG	9,16,23,30,37,44,51,58,65, 72,79,86,93,100,107,114,121, 128,135,142,149,156,163,170, 177
		0: no desquamation, field 1	DSNG	2
		Fissures		
		0: none	DSNG	9,16,23,30,37,44,51,58,65, 72,79,86,93,100,107,114,121, 128,135,142,149,156,163,170, 177
		0: none, field 1	DSNG	2

Table
Individual Clinical Observations
Test Item (dosage) 1 2 3 4

[redacted] [redacted]

Group/ Sex	Animal Number	Observation	Phase	Day(s)
2/M	P0102	Eschar formation		
		no	DSNG	9,16,23,30,37,44,51,58,65, 72,79,86,93,100,107,114,121, 128,135,142,149,156,163,170, 177
		no, field 1	DSNG	2
		Ulcer		
		no	DSNG	9,16,23,30,37,44,51,58,65, 72,79,86,93,100,107,114,121, 128,135,142,149,156,163,170, 177
		no, field 1	DSNG	2

Table
Individual Clinical Observations
Test Item (dosage) 1 2 3 4

[redacted] ----- [redacted]

Group/ Sex	Animal Number	Observation	Phase	Day(s)
2/M	P0103	Erythema		
		0: no erythema	DSNG	9,16,23,30,37,44,51,58,65, 72,79,86,93,100,107,114,121, 128,135,142,149,156,163,170, 177
		0: no erythema, field 1	DSNG	2
		Oedema		
		0: no oedema	DSNG	9,16,23,30,37,44,51,58,65, 72,79,86,93,100,107,114,121, 128,135,142,149,156,163,170, 177
		0: no oedema, field 1	DSNG	2
		Desquamation		
		0: no desquamation	DSNG	9,16,23,30,37,44,51,58,65, 72,79,86,93,100,107,114,121, 128,135,142,149,156,163,170, 177
		0: no desquamation, field 1	DSNG	2
		Fissures		
		0: none	DSNG	9,16,23,30,37,44,51,58,65, 72,79,86,93,100,107,114,121, 128,135,142,149,156,163,170, 177
		0: none, field 1	DSNG	2

Table
Individual Clinical Observations
Test Item (dosage) 1 2 3 4

[redacted] ----- [redacted]

Group/ Sex	Animal Number	Observation	Phase	Day(s)
2/M	P0103	Eschar formation		
		no	DSNG	9,16,23,30,37,44,51,58,65, 72,79,86,93,100,107,114,121, 128,135,142,149,156,163,170, 177
		no, field 1	DSNG	2
		Ulcer		
		no	DSNG	9,16,23,30,37,44,51,58,65, 72,79,86,93,100,107,114,121, 128,135,142,149,156,163,170, 177
		no, field 1	DSNG	2

Table
Individual Clinical Observations
Test Item (dosage) 1 2 3 4

[redacted] [redacted]

Group/ Sex	Animal Number	Observation	Phase	Day(s)
2/M	P0104	Erythema		
		0: no erythema	DSNG	9,16,23,30,37,44,51,58,65, 72,79,86,93,100,107,114,121, 128,135,142,149,156,163,170, 177
		0: no erythema, field 1	DSNG	2
		Oedema		
		0: no oedema	DSNG	9,16,23,30,37,44,51,58,65, 72,79,86,93,100,107,114,121, 128,135,142,149,156,163,170, 177
		0: no oedema, field 1	DSNG	2
		Desquamation		
		0: no desquamation	DSNG	9,16,23,30,37,44,51,58,65, 72,79,86,93,100,107,114,121, 128,135,142,149,156,163,170, 177
		0: no desquamation, field 1	DSNG	2
		Fissures		
		0: none	DSNG	9,16,23,30,37,44,51,58,65, 72,79,86,93,100,107,114,121, 128,135,142,149,156,163,170, 177
		0: none, field 1	DSNG	2

Table
Individual Clinical Observations
Test Item (dosage) 1 2 3 4

[redacted] [redacted]

Group/ Sex	Animal Number	Observation	Phase	Day(s)
2/M	P0104	Eschar formation		
		no	DSNG	9,16,23,30,37,44,51,58,65, 72,79,86,93,100,107,114,121, 128,135,142,149,156,163,170, 177
		no, field 1	DSNG	2
		Ulcer		
		no	DSNG	9,16,23,30,37,44,51,58,65, 72,79,86,93,100,107,114,121, 128,135,142,149,156,163,170, 177
		no, field 1	DSNG	2

Table
Individual Clinical Observations
Test Item (dosage) 1 2 3 4

[redacted] [redacted]

Group/ Sex	Animal Number	Observation	Phase	Day(s)
3/M	P0201	Erythema		
		0: no erythema	DSNG	9,16,23,30,37,44,51,58,65, 72,79,86,93,100,107,114,121, 128,135,142,149,156,163,170, 177
		0: no erythema, field 1	DSNG	2
		Oedema		
		0: no oedema	DSNG	9,16,23,30,37,44,51,58,65, 72,79,86,93,100,107,114,121, 128,135,142,149,156,163,170, 177
		0: no oedema, field 1	DSNG	2
		Desquamation		
		0: no desquamation	DSNG	9,16,23,30,37,44,51,58,65, 72,79,86,93,100,107,114,121, 128,135,142,149,156,163,170, 177
		0: no desquamation, field 1	DSNG	2
		Fissures		
		0: none	DSNG	9,16,23,30,37,44,51,58,65, 72,79,86,93,100,107,114,121, 128,135,142,149,156,163,170, 177
		0: none, field 1	DSNG	2

Table
Individual Clinical Observations
Test Item (dosage) 1 2 3 4

[redacted] --- [redacted] - / [redacted]

Group/ Sex	Animal Number	Observation	Phase	Day(s)
3/M	P0201	Eschar formation		
		no	DSNG	9,16,23,30,37,44,51,58,65, 72,79,86,93,100,107,114,121, 128,135,142,149,156,163,170, 177
		no, field 1	DSNG	2
		Ulcer		
		no	DSNG	9,16,23,30,37,44,51,58,65, 72,79,86,93,100,107,114,121, 128,135,142,149,156,163,170, 177
		no, field 1	DSNG	2

Table
Individual Clinical Observations
Test Item (dosage) 1 2 3 4

[redacted] --- [redacted]

Group/ Sex	Animal Number	Observation	Phase	Day(s)
3/M	P0202	Erythema		
		0: no erythema	DSNG	9,16,23,30,37,44,51,58,65, 72,79,86,93,100,107,114,121, 128,135,142,149,156,163,170, 177
		0: no erythema, field 1	DSNG	2
		Oedema		
		0: no oedema	DSNG	9,16,23,30,37,44,51,58,65, 72,79,86,93,100,107,114,121, 128,135,142,149,156,163,170, 177
		0: no oedema, field 1	DSNG	2
		Desquamation		
		0: no desquamation	DSNG	9,16,23,30,37,44,51,58,65, 72,79,86,93,100,107,114,121, 128,135,142,149,156,163,170, 177
		0: no desquamation, field 1	DSNG	2
		Fissures		
		0: none	DSNG	9,16,23,30,37,44,51,58,65, 72,79,86,93,100,107,114,121, 128,135,142,149,156,163,170, 177
		0: none, field 1	DSNG	2

Table
Individual Clinical Observations
Test Item (dosage) 1 2 3 4

[redacted] ----- [redacted] --

Group/ Sex	Animal Number	Observation	Phase	Day(s)
3/M	P0202	Eschar formation		
		no	DSNG	9,16,23,30,37,44,51,58,65, 72,79,86,93,100,107,114,121, 128,135,142,149,156,163,170, 177
		no, field 1	DSNG	2
		Ulcer		
		no	DSNG	9,16,23,30,37,44,51,58,65, 72,79,86,93,100,107,114,121, 128,135,142,149,156,163,170, 177
		no, field 1	DSNG	2

Table
Individual Clinical Observations
Test Item (dosage) 1 2 3 4

[redacted] [redacted]

Group/ Sex	Animal Number	Observation	Phase	Day(s)
3/M	P0203	Erythema		
		0: no erythema	DSNG	9,16,23,30,37,44,51,58,65, 72,79,86,93,100,107,114,121, 128,135,142,149,156,163,170, 177
		0: no erythema, field 1	DSNG	2
		Oedema		
		0: no oedema	DSNG	9,16,23,30,37,44,51,58,65, 72,79,86,93,100,107,114,121, 128,135,142,149,156,163,170, 177
		0: no oedema, field 1	DSNG	2
		Desquamation		
		0: no desquamation	DSNG	9,16,23,30,37,44,51,58,65, 72,79,86,93,100,107,114,121, 128,135,142,149,156,163,170, 177
		0: no desquamation, field 1	DSNG	2
		Fissures		
		0: none	DSNG	9,16,23,30,37,44,51,58,65, 72,79,86,93,100,107,114,121, 128,135,142,149,156,163,170, 177
		0: none, field 1	DSNG	2

Table
Individual Clinical Observations
Test Item (dosage) 1 2 3 4

[redacted] ----- [redacted] ----------

Group/ Sex	Animal Number	Observation	Phase	Day(s)
3/M	P0203	Eschar formation		
		no	DSNG	9,16,23,30,37,44,51,58,65, 72,79,86,93,100,107,114,121, 128,135,142,149,156,163,170, 177
		no, field 1	DSNG	2
		Ulcer		
		no	DSNG	9,16,23,30,37,44,51,58,65, 72,79,86,93,100,107,114,121, 128,135,142,149,156,163,170, 177
		no, field 1	DSNG	2

Table
Individual Clinical Observations
Test Item (dosage) 1 2 3 4

[redacted] --- [redacted]

Group/ Sex	Animal Number	Observation	Phase	Day(s)
3/M	P0204	Erythema		
		0: no erythema	DSNG	9,16,23,30,37,44,51,58,65, 72,79,86,93,100,107,114,121, 128,135,142,149,156,163,170, 177
		0: no erythema, field 1	DSNG	2
		Oedema		
		0: no oedema	DSNG	9,16,23,30,37,44,51,58,65, 72,79,86,93,100,107,114,121, 128,135,142,149,156,163,170, 177
		0: no oedema, field 1	DSNG	2
		Desquamation		
		0: no desquamation	DSNG	9,16,23,30,37,44,51,58,65, 72,79,86,93,100,107,114,121, 128,135,142,149,156,163,170, 177
		0: no desquamation, field 1	DSNG	2
		Fissures		
		0: none	DSNG	9,16,23,30,37,44,51,58,65, 72,79,86,93,100,107,114,121, 128,135,142,149,156,163,170, 177
		0: none, field 1	DSNG	2

Table
Individual Clinical Observations
Test Item (dosage) 1 2 3 4

[redacted] [redacted]

Group/ Sex	Animal Number	Observation	Phase	Day(s)
3/M	P0204	Eschar formation		
		no	DSNG	9,16,23,30,37,44,51,58,65, 72,79,86,93,100,107,114,121, 128,135,142,149,156,163,170, 177
		no, field 1	DSNG	2
		Ulcer		
		no	DSNG	9,16,23,30,37,44,51,58,65, 72,79,86,93,100,107,114,121, 128,135,142,149,156,163,170, 177
		no, field 1	DSNG	2

Table
Individual Clinical Observations

[redacted]

Group/ Sex	Animal Number	Observation	Phase	Day(s)
4/M	P0301	Erythema		
		0: no erythema	DSNG	9,16,23,30,37,44,51,58,65, 72,79,86,93,100,107,114,121, 128,135,142,149,156,163,170, 177
		0: no erythema, field 1	DSNG	2
		Oedema		
		0: no oedema	DSNG	9,16,23,30,37,44,51,58,65, 72,79,86,93,100,107,114,121, 128,135,142,149,156,163,170, 177
		0: no oedema, field 1	DSNG	2
		Desquamation		
		0: no desquamation	DSNG	9,16,23,30,37,44,51,58,65, 72,79,86,93,100,107,114,121, 128,135,142,149,156,163,170, 177
		0: no desquamation, field 1	DSNG	2
		Fissures		
		0: none	DSNG	9,16,23,30,37,44,51,58,65, 72,79,86,93,100,107,114,121, 128,135,142,149,156,163,170, 177
		0: none, field 1	DSNG	2

Table
Individual Clinical Observations
Test Item (dosage) 1 2 3 4

[redacted] [redacted]

Group/ Sex	Animal Number	Observation	Phase	Day(s)
4/M	P0301	Eschar formation		
		no	DSNG	9,16,23,30,37,44,51,58,65, 72,79,86,93,100,107,114,121, 128,135,142,149,156,163,170, 177
		no, field 1	DSNG	2
		Ulcer		
		no	DSNG	9,16,23,30,37,44,51,58,65, 72,79,86,93,100,107,114,121, 128,135,142,149,156,163,170, 177
		no, field 1	DSNG	2

Table
Individual Clinical Observations
Test Item (dosage) 1 2 3 4
[redacted] [redacted]/[redacted]

Group/ Sex	Animal Number	Observation	Phase	Day(s)
4/M	P0302	Erythema		
		0: no erythema	DSNG	9,16,23,30,37,44,51,58,65, 72,79,86,93,100,107,114,121, 128,135,142,149,156,163,170, 177
		0: no erythema, field 1	DSNG	2
		Oedema		
		0: no oedema	DSNG	9,16,23,30,37,44,51,58,65, 72,79,86,93,100,107,114,121, 128,135,142,149,156,163,170, 177
		0: no oedema, field 1	DSNG	2
		Desquamation		
		0: no desquamation	DSNG	9,16,23,30,37,44,51,58,65, 72,79,86,93,100,107,114,121, 128,135,142,149,156,163,170, 177
		0: no desquamation, field 1	DSNG	2
		Fissures		
		0: none	DSNG	9,16,23,30,37,44,51,58,65, 72,79,86,93,100,107,114,121, 128,135,142,149,156,163,170, 177
		0: none, field 1	DSNG	2

Table
Individual Clinical Observations
Test Item (dosage) 1 2 3 4

[redacted] [redacted]

Group/ Sex	Animal Number	Observation	Phase	Day(s)
4/M	P0302	Eschar formation		
		no	DSNG	9,16,23,30,37,44,51,58,65, 72,79,86,93,100,107,114,121, 128,135,142,149,156,163,170, 177
		no, field 1	DSNG	2
		Ulcer		
		no	DSNG	9,16,23,30,37,44,51,58,65, 72,79,86,93,100,107,114,121, 128,135,142,149,156,163,170, 177
		no, field 1	DSNG	2

Table
Individual Clinical Observations
Test Item (dosage) 1 2 3 4

[redacted] [redacted]

Group/ Sex	Animal Number	Observation	Phase	Day(s)
4/M	P0303	Erythema		
		0: no erythema	DSNG	9,16,23,30,37,44,51,58,65, 72,79,86,93,100,107,114,121, 128,135,142,149,156,163,170, 177-179
		0: no erythema, field 1	DSNG	2
		Oedema		
		0: no oedema	DSNG	9,16,23,30,37,44,51,58,65, 72,79,86,93,100,107,114,121, 128,135,142,149,156,163,170, 179
		0: no oedema, field 1	DSNG	2
		2: moderate oedema, field 1	DSNG	177
		3: severe oedema, field 1	DSNG	178
		Desquamation		
		0: no desquamation	DSNG	9,16,23,30,37,44,51,58,65, 72,79,86,93,100,107,114,121, 128,135,142,149,156,163,170, 177-179
		0: no desquamation, field 1	DSNG	2

Table
Individual Clinical Observations
Test Item (dosage) 1 2 3 4

[redacted] [redacted]

Group/ Sex	Animal Number	Observation	Phase	Day(s)
4/M	P0303	Fissures		
		0: none	DSNG	9,16,23,30,37,44,51,58,65, 72,79,86,93,100,107,114,121, 128,135,142,149,156,163,170, 177-179
		0: none, field 1	DSNG	2
		Eschar formation		
		no	DSNG	9,16,23,30,37,44,51,58,65, 72,79,86,93,100,107,114,121, 128,135,142,149,156,163,170, 177-179
		no, field 1	DSNG	2
		Ulcer		
		no	DSNG	9,16,23,30,37,44,51,58,65, 72,79,86,93,100,107,114,121, 128,135,142,149,156,163,170, 177-179
		no, field 1	DSNG	2

Table
Individual Clinical Observations
Test Item (dosage) 1 2 3 4

[redacted] [redacted]

Group/ Sex	Animal Number	Observation	Phase	Day(s)
4/M	P0304	Erythema		
		0: no erythema	DSNG	9,16,23,30,37,44,51,58,65, 72,79,86,93,100,107,114,121, 128,135,142,149,156,163,170, 177
		0: no erythema, field 1	DSNG	2
		Oedema		
		0: no oedema	DSNG	9,16,23,30,37,44,51,58,65, 72,79,86,93,100,107,114,121, 128,135,142,149,156,163,170, 177
		0: no oedema, field 1	DSNG	2
		Desquamation		
		0: no desquamation	DSNG	9,16,23,30,37,44,51,58,65, 72,79,86,93,100,107,114,121, 128,135,142,149,156,163,170, 177
		0: no desquamation, field 1	DSNG	2
		Fissures		
		0: none	DSNG	9,16,23,30,37,44,51,58,65, 72,79,86,93,100,107,114,121, 128,135,142,149,156,163,170, 177
		0: none, field 1	DSNG	2

Table
Individual Clinical Observations
Test Item (dosage) 1 2 3 4

[redacted] ----- [redacted]

Group/ Sex	Animal Number	Observation	Phase	Day(s)
4/M	P0304	Eschar formation		
		no	DSNG	9,16,23,30,37,44,51,58,65, 72,79,86,93,100,107,114,121, 128,135,142,149,156,163,170, 177
		no, field 1	DSNG	2
		Ulcer		
		no	DSNG	9,16,23,30,37,44,51,58,65, 72,79,86,93,100,107,114,121, 128,135,142,149,156,163,170, 177
		no, field 1	DSNG	2

Table
Individual Clinical Observations
Test Item (dosage) 1 2 3 4

[redacted] [redacted]

Group/ Sex	Animal Number	Observation	Phase	Day(s)
4/M	P0305	Erythema		
		0: no erythema	DSNG	9,16,23,30,37,44,51,58,65, 72,79,86,93,100,107,114,121, 128,135,142,149,156,163,170, 177
		0: no erythema, field 1	DSNG	2
		Oedema		
		0: no oedema	DSNG	9,16,23,30,37,44,51,58,65, 72,79,86,93,100,107,114,121, 128,135,142,149,156,163,170, 177
		0: no oedema, field 1	DSNG	2
		Desquamation		
		0: no desquamation	DSNG	9,16,23,30,37,44,51,58,65, 72,79,86,93,100,107,114,121, 128,135,142,149,156,163,170, 177
		0: no desquamation, field 1	DSNG	2
		Fissures		
		0: none	DSNG	9,16,23,30,37,44,51,58,65, 72,79,86,93,100,107,114,121, 128,135,142,149,156,163,170, 177
		0: none, field 1	DSNG	2

Table
Individual Clinical Observations
Test Item (dosage) 1 2 3 4

[redacted] [redacted]

Group/ Sex	Animal Number	Observation	Phase	Day(s)
4/M	P0305	Eschar formation		
		no	DSNG	9,16,23,30,37,44,51,58,65, 72,79,86,93,100,107,114,121, 128,135,142,149,156,163,170, 177
		no, field 1	DSNG	2
		Ulcer		
		no	DSNG	9,16,23,30,37,44,51,58,65, 72,79,86,93,100,107,114,121, 128,135,142,149,156,163,170, 177
		no, field 1	DSNG	2

Table
Individual Clinical Observations

[redacted]

Group/ Sex	Animal Number	Observation	Phase	Day(s)
4/M	P0306	Erythema		
		0: no erythema	DSNG	9,16,23,30,37,44,51,58,65, 72,79,86,93,100,107,114,121, 128,135,142,149,156,163,170, 177-183
		0: no erythema, field 1	DSNG	2
		Oedema		
		0: no oedema	DSNG	9,16,23,30,37,44,51,58,65, 72,79,86,93,100,107,114,121, 128,135,142,149,156,163,170
		0: no oedema, field 1	DSNG	2
		1: slight oedema	DSNG	182,183
		3: severe oedema, field 1	DSNG	177-181
		Desquamation		
		0: no desquamation	DSNG	9,16,23,30,37,44,51,58,65, 72,79,86,93,100,107,114,121, 128,135,142,149,156,163,170, 177-183
		0: no desquamation, field 1	DSNG	2

Table
Individual Clinical Observations
Test Item (dosage) 1 2 3 4

[redacted] [redacted]

Group/ Sex	Animal Number	Observation	Phase	Day(s)
4/M	P0306	Fissures		
		0: none	DSNG	9,16,23,30,37,44,51,58,65, 72,79,86,93,100,107,114,121, 128,135,142,149,156,163,170, 177-183
		0: none, field 1	DSNG	2
		Eschar formation		
		no	DSNG	9,16,23,30,37,44,51,58,65, 72,79,86,93,100,107,114,121, 128,135,142,149,156,163,170, 177-183
		no, field 1	DSNG	2
		Ulcer		
		no	DSNG	9,16,23,30,37,44,51,58,65, 72,79,86,93,100,107,114,121, 128,135,142,149,156,163,170, 177-183
		no, field 1	DSNG	2

Table
Individual Clinical Observations
Test Item (dosage) 1 2 3 4

[redacted] --- [redacted]

Group/ Sex	Animal Number	Observation	Phase	Day(s)
1/F	P0401	Erythema		
		0: no erythema	DSNG	9,16,23,30,37,44,51,58,65, 72,79,86,93,100,107,114,121, 128,135,142,149,156,163,170, 177
		0: no erythema, field 1	DSNG	2
		Oedema		
		0: no oedema	DSNG	9,16,23,30,37,44,51,58,65, 72,79,86,93,100,107,114,121, 128,135,142,149,156,163,170, 177
		0: no oedema, field 1	DSNG	2
		Desquamation		
		0: no desquamation	DSNG	9,16,23,30,37,44,51,58,65, 72,79,86,93,100,107,114,121, 128,135,142,149,156,163,170, 177
		0: no desquamation, field 1	DSNG	2
		Fissures		
		0: none	DSNG	9,16,23,30,37,44,51,58,65, 72,79,86,93,100,107,114,121, 128,135,142,149,156,163,170, 177
		0: none, field 1	DSNG	2

Table
Individual Clinical Observations
Test Item (dosage) 1 2 3 4

[redacted] ----- [redacted]

Group/ Sex	Animal Number	Observation	Phase	Day(s)
1/F	P0401	Eschar formation		
		no	DSNG	9,16,23,30,37,44,51,58,65, 72,79,86,93,100,107,114,121, 128,135,142,149,156,163,170, 177
		no, field 1	DSNG	2
		Ulcer		
		no	DSNG	9,16,23,30,37,44,51,58,65, 72,79,86,93,100,107,114,121, 128,135,142,149,156,163,170, 177
		no, field 1	DSNG	2

Table
Individual Clinical Observations
Test Item (dosage) 1 2 3 4

[redacted] [redacted]

Group/ Sex	Animal Number	Observation	Phase	Day(s)
1/F	P0402	Erythema		
		0: no erythema	DSNG	9,16,23,30,37,44,51,58,65, 72,79,86,93,100,107,114,121, 128,135,142,149,156,163,170, 177
		0: no erythema, field 1	DSNG	2
		Oedema		
		0: no oedema	DSNG	9,16,23,30,37,44,51,58,65, 72,79,86,93,100,107,114,121, 128,135,142,149,156,163,170, 177
		0: no oedema, field 1	DSNG	2
		Desquamation		
		0: no desquamation	DSNG	9,16,23,30,37,44,51,58,65, 72,79,86,93,100,107,114,121, 128,135,142,149,156,163,170, 177
		0: no desquamation, field 1	DSNG	2
		Fissures		
		0: none	DSNG	9,16,23,30,37,44,51,58,65, 72,79,86,93,100,107,114,121, 128,135,142,149,156,163,170, 177
		0: none, field 1	DSNG	2

Table
Individual Clinical Observations
Test Item (dosage) 1 2 3 4

[redacted] ----- [redacted]

Group/ Sex	Animal Number	Observation	Phase	Day(s)
1/F	P0402	Eschar formation		
		no	DSNG	9,16,23,30,37,44,51,58,65, 72,79,86,93,100,107,114,121, 128,135,142,149,156,163,170, 177
		no, field 1	DSNG	2
		Ulcer		
		no	DSNG	9,16,23,30,37,44,51,58,65, 72,79,86,93,100,107,114,121, 128,135,142,149,156,163,170, 177
		no, field 1	DSNG	2

Table
Individual Clinical Observations
Test Item (dosage) 1 2 3 4

[redacted] [redacted]

Group/ Sex	Animal Number	Observation	Phase	Day(s)
1/F	P0403	Erythema		
		0: no erythema	DSNG	9,16,23,30,37,44,51,58,65, 72,79,86,93,100,107,114,121, 128,135,142,149,156,163,170, 177
		0: no erythema, field 1	DSNG	2
		Oedema		
		0: no oedema	DSNG	9,16,23,30,37,44,51,58,65, 72,79,86,93,100,107,114,121, 128,135,142,149,156,163,170, 177
		0: no oedema, field 1	DSNG	2
		Desquamation		
		0: no desquamation	DSNG	9,16,23,30,37,44,51,58,65, 72,79,86,93,100,107,114,121, 128,135,142,149,156,163,170, 177
		0: no desquamation, field 1	DSNG	2
		Fissures		
		0: none	DSNG	9,16,23,30,37,44,51,58,65, 72,79,86,93,100,107,114,121, 128,135,142,149,156,163,170, 177
		0: none, field 1	DSNG	2

Table
Individual Clinical Observations
Test Item (dosage) 1 2 3 4

[redacted] ----- [redacted]

Group/ Sex	Animal Number	Observation	Phase	Day(s)
1/F	P0403	Eschar formation		
		no	DSNG	9,16,23,30,37,44,51,58,65, 72,79,86,93,100,107,114,121, 128,135,142,149,156,163,170, 177
		no, field 1	DSNG	2
		Ulcer		
		no	DSNG	9,16,23,30,37,44,51,58,65, 72,79,86,93,100,107,114,121, 128,135,142,149,156,163,170, 177
		no, field 1	DSNG	2

Table
Individual Clinical Observations
Test Item (dosage) 1 2 3 4 [redacted]

[redacted] [redacted]

Group/ Sex	Animal Number	Observation	Phase	Day(s)
1/F	P0404	Erythema		
		0: no erythema	DSNG	9,16,23,30,37,44,51,58,65, 72,79,86,93,100,107,114,121, 128,135,142,149,156,163,170, 177
		0: no erythema, field 1	DSNG	2
		Oedema		
		0: no oedema	DSNG	9,16,23,30,37,44,51,58,65, 72,79,86,93,100,107,114,121, 128,135,142,149,156,163,170, 177
		0: no oedema, field 1	DSNG	2
		Desquamation		
		0: no desquamation	DSNG	9,16,23,30,37,44,51,58,65, 72,79,86,93,100,107,114,121, 128,135,142,149,156,163,170, 177
		0: no desquamation, field 1	DSNG	2
		Fissures		
		0: none	DSNG	9,16,23,30,37,44,51,58,65, 72,79,86,93,100,107,114,121, 128,135,142,149,156,163,170, 177
		0: none, field 1	DSNG	2

Table
Individual Clinical Observations
Test Item (dosage) 1 2 3 4

[redacted] [redacted]

Group/ Sex	Animal Number	Observation	Phase	Day(s)
1/F	P0404	Eschar formation		
		no	DSNG	9,16,23,30,37,44,51,58,65, 72,79,86,93,100,107,114,121, 128,135,142,149,156,163,170, 177
		no, field 1	DSNG	2
		Ulcer		
		no	DSNG	9,16,23,30,37,44,51,58,65, 72,79,86,93,100,107,114,121, 128,135,142,149,156,163,170, 177
		no, field 1	DSNG	2

Table
Individual Clinical Observations
Test Item (dosage) 1 2 3 4

[redacted] ----- [redacted]

Group/ Sex	Animal Number	Observation	Phase	Day(s)
1/F	P0405	Erythema		
		0: no erythema	DSNG	9,16,23,30,37,44,51,58,65, 72,79,86,93,100,107,114,121, 128,135,142,149,156,163,170, 177
		0: no erythema, field 1	DSNG	2
		Oedema		
		0: no oedema	DSNG	9,16,23,30,37,44,51,58,65, 72,79,86,93,100,107,114,121, 128,135,142,149,156,163,170, 177
		0: no oedema, field 1	DSNG	2
		Desquamation		
		0: no desquamation	DSNG	9,16,23,30,37,44,51,58,65, 72,79,86,93,100,107,114,121, 128,135,142,149,156,163,170, 177
		0: no desquamation, field 1	DSNG	2
		Fissures		
		0: none	DSNG	9,16,23,30,37,44,51,58,65, 72,79,86,93,100,107,114,121, 128,135,142,149,156,163,170, 177
		0: none, field 1	DSNG	2

Table
Individual Clinical Observations
Test Item (dosage) 1 2 3 4

[redacted] ----- [redacted]

Group/ Sex	Animal Number	Observation	Phase	Day(s)
1/F	P0405	Eschar formation		
		no	DSNG	9,16,23,30,37,44,51,58,65, 72,79,86,93,100,107,114,121, 128,135,142,149,156,163,170, 177
		no, field 1	DSNG	2
		Ulcer		
		no	DSNG	9,16,23,30,37,44,51,58,65, 72,79,86,93,100,107,114,121, 128,135,142,149,156,163,170, 177
		no, field 1	DSNG	2

Table
Individual Clinical Observations
Test Item (dosage) 1 2 3 4

[redacted]

Group/ Sex	Animal Number	Observation	Phase	Day(s)
1/F	P0406	Erythema		
		0: no erythema	DSNG	9,16,23,30,37,44,51,58,65, 72,79,86,93,100,107,114,121, 128,135,142,149,156,163,170, 177
		0: no erythema, field 1	DSNG	2
		Oedema		
		0: no oedema	DSNG	9,16,23,30,37,44,51,58,65, 72,79,86,93,100,107,114,121, 128,135,142,149,156,163,170, 177
		0: no oedema, field 1	DSNG	2
		Desquamation		
		0: no desquamation	DSNG	9,16,23,30,37,44,51,58,65, 72,79,86,93,100,107,114,121, 128,135,142,149,156,163,170, 177
		0: no desquamation, field 1	DSNG	2
		Fissures		
		0: none	DSNG	9,16,23,30,37,44,51,58,65, 72,79,86,93,100,107,114,121, 128,135,142,149,156,163,170, 177
		0: none, field 1	DSNG	2

Table
Individual Clinical Observations
Test Item (dosage) 1 2 3 4

[redacted] [redacted]

Group/ Sex	Animal Number	Observation	Phase	Day(s)
1/F	P0406	Eschar formation		
		no	DSNG	9,16,23,30,37,44,51,58,65, 72,79,86,93,100,107,114,121, 128,135,142,149,156,163,170, 177
		no, field 1	DSNG	2
		Ulcer		
		no	DSNG	9,16,23,30,37,44,51,58,65, 72,79,86,93,100,107,114,121, 128,135,142,149,156,163,170, 177
		no, field 1	DSNG	2

Table
Individual Clinical Observations
Test Item (dosage) 1 2 3 4

[redacted] [redacted]

Group/ Sex	Animal Number	Observation	Phase	Day(s)
2/F	P0501	Erythema		
		0: no erythema	DSNG	9,16,23,30,37,44,51,58,65, 72,79,86,93,100,107,114,121, 128,135,142,149,156,163,170, 177
		0: no erythema, field 1	DSNG	2
		Oedema		
		0: no oedema	DSNG	9,16,23,30,37,44,51,58,65, 72,79,86,93,100,107,114,121, 128,135,142,149,156,163,170, 177
		0: no oedema, field 1	DSNG	2
		Desquamation		
		0: no desquamation	DSNG	9,16,23,30,37,44,51,58,65, 72,79,86,93,100,107,114,121, 128,135,142,149,156,163,170, 177
		0: no desquamation, field 1	DSNG	2
		Fissures		
		0: none	DSNG	9,16,23,30,37,44,51,58,65, 72,79,86,93,100,107,114,121, 128,135,142,149,156,163,170, 177
		0: none, field 1	DSNG	2

Table
Individual Clinical Observations
Test Item (dosage) 1 2 3 4

[redacted] ----- [redacted] --

Group/ Sex	Animal Number	Observation	Phase	Day(s)
2/F	P0501	Eschar formation		
		no	DSNG	9,16,23,30,37,44,51,58,65, 72,79,86,93,100,107,114,121, 128,135,142,149,156,163,170, 177
		no, field 1	DSNG	2
		Ulcer		
		no	DSNG	9,16,23,30,37,44,51,58,65, 72,79,86,93,100,107,114,121, 128,135,142,149,156,163,170, 177
		no, field 1	DSNG	2

Table
Individual Clinical Observations
Test Item (dosage) 1 2 3 4

[redacted] ----- [redacted]

Group/ Sex	Animal Number	Observation	Phase	Day(s)
2/F	P0502	Erythema		
		0: no erythema	DSNG	9,16,23,30,37,44,51,58,65, 72,79,86,93,100,107,114,121, 128,135,142,149,156,163,170, 177
		0: no erythema, field 1	DSNG	2
		Oedema		
		0: no oedema	DSNG	9,16,23,30,37,44,51,58,65, 72,79,86,93,100,107,114,121, 128,135,142,149,156,163,170, 177
		0: no oedema, field 1	DSNG	2
		Desquamation		
		0: no desquamation	DSNG	9,16,23,30,37,44,51,58,65, 72,79,86,93,100,107,114,121, 128,135,142,149,156,163,170, 177
		0: no desquamation, field 1	DSNG	2
		Fissures		
		0: none	DSNG	9,16,23,30,37,44,51,58,65, 72,79,86,93,100,107,114,121, 128,135,142,149,156,163,170, 177
		0: none, field 1	DSNG	2

Table
Individual Clinical Observations
Test Item (dosage) 1 2 3 4

[redacted] ----- [redacted]

Group/ Sex	Animal Number	Observation	Phase	Day(s)
2/F	P0502	Eschar formation		
		no	DSNG	9,16,23,30,37,44,51,58,65, 72,79,86,93,100,107,114,121, 128,135,142,149,156,163,170, 177
		no, field 1	DSNG	2
		Ulcer		
		no	DSNG	9,16,23,30,37,44,51,58,65, 72,79,86,93,100,107,114,121, 128,135,142,149,156,163,170, 177
		no, field 1	DSNG	2

Table
Individual Clinical Observations
Test Item (dosage) 1 2 3 4

[redacted] [redacted]

Group/ Sex	Animal Number	Observation	Phase	Day(s)
2/F	P0503	Erythema		
		0: no erythema	DSNG	9,16,23,30,37,44,51,58,65, 72,79,86,93,100,107,114,121, 128,135,142,149,156,163,170, 177
		0: no erythema, field 1	DSNG	2
		Oedema		
		0: no oedema	DSNG	9,16,23,30,37,44,51,58,65, 72,79,86,93,100,107,114,121, 128,135,142,149,156,163,170, 177
		0: no oedema, field 1	DSNG	2
		Desquamation		
		0: no desquamation	DSNG	9,16,23,30,37,44,51,58,65, 72,79,86,93,100,107,114,121, 128,135,142,149,156,163,170, 177
		0: no desquamation, field 1	DSNG	2
		Fissures		
		0: none	DSNG	9,16,23,30,37,44,51,58,65, 72,79,86,93,100,107,114,121, 128,135,142,149,156,163,170, 177
		0: none, field 1	DSNG	2

Table
Individual Clinical Observations
Test Item (dosage) 1 2 3 4

[redacted] [redacted]

Group/ Sex	Animal Number	Observation	Phase	Day(s)
2/F	P0503	Eschar formation		
		no	DSNG	9,16,23,30,37,44,51,58,65, 72,79,86,93,100,107,114,121, 128,135,142,149,156,163,170, 177
		no, field 1	DSNG	2
		Ulcer		
		no	DSNG	9,16,23,30,37,44,51,58,65, 72,79,86,93,100,107,114,121, 128,135,142,149,156,163,170, 177
		no, field 1	DSNG	2

Table
Individual Clinical Observations
Test Item (dosage) 1 2 3 4

[redacted] ----- [redacted] ---

Group/ Sex	Animal Number	Observation	Phase	Day(s)
2/F	P0504	Erythema		
		0: no erythema	DSNG	9,16,23,30,37,44,51,58,65, 72,79,86,93,100,107,114,121, 128,135,142,149,156,163,170, 177
		0: no erythema, field 1	DSNG	2
		Oedema		
		0: no oedema	DSNG	9,16,23,30,37,44,51,58,65, 72,79,86,93,100,107,114,121, 128,135,142,149,156,163,170, 177
		0: no oedema, field 1	DSNG	2
		Desquamation		
		0: no desquamation	DSNG	9,16,23,30,37,44,51,58,65, 72,79,86,93,100,107,114,121, 128,135,142,149,156,163,170, 177
		0: no desquamation, field 1	DSNG	2
		Fissures		
		0: none	DSNG	9,16,23,30,37,44,51,58,65, 72,79,86,93,100,107,114,121, 128,135,142,149,156,163,170, 177
		0: none, field 1	DSNG	2

Table
Individual Clinical Observations
Test Item (dosage) 1 2 3 4

[redacted] ----- [redacted]

Group/ Sex	Animal Number	Observation	Phase	Day(s)
2/F	P0504	Eschar formation		
		no	DSNG	9,16,23,30,37,44,51,58,65, 72,79,86,93,100,107,114,121, 128,135,142,149,156,163,170, 177
		no, field 1	DSNG	2
		Ulcer		
		no	DSNG	9,16,23,30,37,44,51,58,65, 72,79,86,93,100,107,114,121, 128,135,142,149,156,163,170, 177
		no, field 1	DSNG	2

Table
Individual Clinical Observations
Test Item (dosage) 1 2 3 4

[redacted] [redacted]

Group/ Sex	Animal Number	Observation	Phase	Day(s)
3/F	P0601	Erythema		
		0: no erythema	DSNG	16,23,30,37,44,51,58,65,72, 79,86,93,100,107,114,121, 128,135,142,149,156,163,170, 177
		0: no erythema, field 1	DSNG	2
		0: no erythema, field 2	DSNG	9
		Oedema		
		0: no oedema	DSNG	16,23,30,37,44,51,58,65,72, 79,86,93,100,107,114,121, 128,135,142,149,156,163,170, 177
		0: no oedema, field 1	DSNG	2
		0: no oedema, field 2	DSNG	9
		Desquamation		
		0: no desquamation	DSNG	16,23,30,37,44,51,58,65,72, 79,86,93,100,107,114,121, 128,135,142,149,156,163,170, 177
		0: no desquamation, field 1	DSNG	2
		0: no desquamation, field 2	DSNG	9

Table
Individual Clinical Observations
Test Item (dosage) 1 2 3 4

[redacted] [redacted]

Group/ Sex	Animal Number	Observation	Phase	Day(s)
3/F	P0601	Fissures		
		0: none	DSNG	16,23,30,37,44,51,58,65,72, 79,86,93,100,107,114,121, 128,135,142,149,156,163,170, 177
		0: none, field 1	DSNG	2
		0: none, field 2	DSNG	9
		Eschar formation		
		no	DSNG	16,23,30,37,44,51,58,65,72, 79,86,93,100,107,114,121, 128,135,142,149,156,163,170, 177
		no, field 1	DSNG	2
		no, field 2	DSNG	9
		Ulcer		
		no	DSNG	16,23,30,37,44,51,58,65,72, 79,86,93,100,107,114,121, 128,135,142,149,156,163,170, 177
		no, field 1	DSNG	2
		no, field 2	DSNG	9

Table
Individual Clinical Observations
Test Item (dosage) 1 2 3 4

[redacted] [redacted]

Group/ Sex	Animal Number	Observation	Phase	Day(s)
3/F	P0602	Erythema		
		0: no erythema	DSNG	16,23,30,37,44,51,58,65,72, 79,86,93,100,107,114,121, 128,135,142,149,156,163,170, 177
		0: no erythema, field 1	DSNG	2
		0: no erythema, field 2	DSNG	9
		Oedema		
		0: no oedema	DSNG	16,23,30,37,44,51,58,65,72, 79,86,93,100,107,114,121, 128,135,142,149,156,163,170, 177
		0: no oedema, field 1	DSNG	2
		0: no oedema, field 2	DSNG	9
		Desquamation		
		0: no desquamation	DSNG	16,23,30,37,44,51,58,65,72, 79,86,93,100,107,114,121, 128,135,142,149,156,163,170, 177
		0: no desquamation, field 1	DSNG	2
		0: no desquamation, field 2	DSNG	9

Table
Individual Clinical Observations
Test Item (dosage) 1 2 3 4

[redacted] [redacted]

Group/ Sex	Animal Number	Observation	Phase	Day(s)
3/F	P0602	Fissures		
		0: none	DSNG	16,23,30,37,44,51,58,65,72, 79,86,93,100,107,114,121, 128,135,142,149,156,163,170, 177
		0: none, field 1	DSNG	2
		0: none, field 2	DSNG	9
		Eschar formation		
		no	DSNG	16,23,30,37,44,51,58,65,72, 79,86,93,100,107,114,121, 128,135,142,149,156,163,170, 177
		no, field 1	DSNG	2
		no, field 2	DSNG	9
		Ulcer		
		no	DSNG	16,23,30,37,44,51,58,65,72, 79,86,93,100,107,114,121, 128,135,142,149,156,163,170, 177
		no, field 1	DSNG	2
		no, field 2	DSNG	9

Table
Individual Clinical Observations
Test Item (dosage) 1 2 3 4

[redacted] ----- [redacted]

Group/ Sex	Animal Number	Observation	Phase	Day(s)
3/F	P0603	Erythema		
		0: no erythema	DSNG	16,23,30,37,44,51,58,65,72, 79,86,93,100,107,114,121, 128,135,142,149,156,163,170, 177
		0: no erythema, field 1	DSNG	2
		0: no erythema, field 2	DSNG	9
		Oedema		
		0: no oedema	DSNG	16,23,30,37,44,51,58,65,72, 79,86,93,100,107,114,121, 128,135,142,149,156,163,170, 177
		0: no oedema, field 1	DSNG	2
		0: no oedema, field 2	DSNG	9
		Desquamation		
		0: no desquamation	DSNG	16,23,30,37,44,51,58,65,72, 79,86,93,100,107,114,121, 128,135,142,149,156,163,170, 177
		0: no desquamation, field 1	DSNG	2
		0: no desquamation, field 2	DSNG	9

Table
Individual Clinical Observations
Test Item (dosage) 1 2 3 4

[redacted] ----- [redacted]

Group/ Sex	Animal Number	Observation	Phase	Day(s)
3/F	P0603	Fissures		
		0: none	DSNG	16,23,30,37,44,51,58,65,72, 79,86,93,100,107,114,121, 128,135,142,149,156,163,170, 177
		0: none, field 1	DSNG	2
		0: none, field 2	DSNG	9
		Eschar formation		
		no	DSNG	16,23,30,37,44,51,58,65,72, 79,86,93,100,107,114,121, 128,135,142,149,156,163,170, 177
		no, field 1	DSNG	2
		no, field 2	DSNG	9
		Ulcer		
		no	DSNG	16,23,30,37,44,51,58,65,72, 79,86,93,100,107,114,121, 128,135,142,149,156,163,170, 177
		no, field 1	DSNG	2
		no, field 2	DSNG	9

Table
Individual Clinical Observations
Test Item (dosage) 1 2 3 4

[redacted] [redacted]

Group/ Sex	Animal Number	Observation	Phase	Day(s)
3/F	P0604	Erythema		
		0: no erythema	DSNG	16,23,30,37,44,51,58,65,72, 79,86,93,100,107,114,121, 128,135,142,149,156,163,170, 177
		0: no erythema, field 1	DSNG	2
		0: no erythema, field 2	DSNG	9
		Oedema		
		0: no oedema	DSNG	16,23,30,37,44,51,58,65,72, 79,86,93,100,107,114,121, 128,135,142,149,156,163,170, 177
		0: no oedema, field 1	DSNG	2
		0: no oedema, field 2	DSNG	9
		Desquamation		
		0: no desquamation	DSNG	16,23,30,37,44,51,58,65,72, 79,86,93,100,107,114,121, 128,135,142,149,156,163,170, 177
		0: no desquamation, field 1	DSNG	2
		0: no desquamation, field 2	DSNG	9

Table
Individual Clinical Observations
Test Item (dosage) 1 2 3 4

[redacted] [redacted]

Group/ Sex	Animal Number	Observation	Phase	Day(s)
3/F	P0604	Fissures		
		0: none	DSNG	16,23,30,37,44,51,58,65,72, 79,86,93,100,107,114,121, 128,135,142,149,156,163,170, 177
		0: none, field 1	DSNG	2
		0: none, field 2	DSNG	9
		Eschar formation		
		no	DSNG	16,23,30,37,44,51,58,65,72, 79,86,93,100,107,114,121, 128,135,142,149,156,163,170, 177
		no, field 1	DSNG	2
		no, field 2	DSNG	9
		Ulcer		
		no	DSNG	16,23,30,37,44,51,58,65,72, 79,86,93,100,107,114,121, 128,135,142,149,156,163,170, 177
		no, field 1	DSNG	2
		no, field 2	DSNG	9

Table
Individual Clinical Observations
Test Item (dosage) 1 2 3 4

[redacted] [redacted]

Group/ Sex	Animal Number	Observation	Phase	Day(s)
4/F	P0701	Erythema		
		0: no erythema	DSNG	16,23,30,37,44,51,58,65,72, 79,86,93,100,107,114,121, 128,135,142,149,156,163,170, 177
		0: no erythema, field 1	DSNG	2
		0: no erythema, field 2	DSNG	9
		Oedema		
		0: no oedema	DSNG	16,23,30,37,44,51,58,65,72, 79,86,93,100,107,114,121, 128,135,142,149,156,163,170, 177
		0: no oedema, field 1	DSNG	2
		0: no oedema, field 2	DSNG	9
		Desquamation		
		0: no desquamation	DSNG	16,23,30,37,44,51,58,65,72, 79,86,93,100,107,114,121, 128,135,142,149,156,163,170, 177
		0: no desquamation, field 1	DSNG	2
		0: no desquamation, field 2	DSNG	9

Table
Individual Clinical Observations
Test Item (dosage) 1 2 3 4

[redacted] --- [redacted]

Group/ Sex	Animal Number	Observation	Phase	Day(s)
4/F	P0701	Fissures		
		0: none	DSNG	16,23,30,37,44,51,58,65,72, 79,86,93,100,107,114,121, 128,135,142,149,156,163,170, 177
		0: none, field 1	DSNG	2
		0: none, field 2	DSNG	9
		Eschar formation		
		no	DSNG	16,23,30,37,44,51,58,65,72, 79,86,93,100,107,114,121, 128,135,142,149,156,163,170, 177
		no, field 1	DSNG	2
		no, field 2	DSNG	9
		Ulcer		
		no	DSNG	16,23,30,37,44,51,58,65,72, 79,86,93,100,107,114,121, 128,135,142,149,156,163,170, 177
		no, field 1	DSNG	2
		no, field 2	DSNG	9

Table
Individual Clinical Observations
Test Item (dosage) 1 2 3 4

[redacted] [redacted]

Group/ Sex	Animal Number	Observation	Phase	Day(s)
4/F	P0702	Erythema		
		0: no erythema	DSNG	16,23,30,37,44,51,58,65,72, 79,86,93,100,107,114,121, 128,135,142,149,156,163,170, 177
		0: no erythema, field 1	DSNG	2
		0: no erythema, field 2	DSNG	9
		Oedema		
		0: no oedema	DSNG	16,23,30,37,44,51,58,65,72, 79,86,93,100,107,114,121, 128,135,142,149,156,163,170, 177
		0: no oedema, field 1	DSNG	2
		0: no oedema, field 2	DSNG	9
		Desquamation		
		0: no desquamation	DSNG	16,23,30,37,44,51,58,65,72, 79,86,93,100,107,114,121, 128,135,142,149,156,163,170, 177
		0: no desquamation, field 1	DSNG	2
		0: no desquamation, field 2	DSNG	9

Table
Individual Clinical Observations
Test Item (dosage) 1 2 3 4

[redacted] ----- [redacted]

Group/ Sex	Animal Number	Observation	Phase	Day(s)
4/F	P0702	Fissures		
		0: none	DSNG	16,23,30,37,44,51,58,65,72, 79,86,93,100,107,114,121, 128,135,142,149,156,163,170, 177
		0: none, field 1	DSNG	2
		0: none, field 2	DSNG	9
		Eschar formation		
		no	DSNG	16,23,30,37,44,51,58,65,72, 79,86,93,100,107,114,121, 128,135,142,149,156,163,170, 177
		no, field 1	DSNG	2
		no, field 2	DSNG	9
		Ulcer		
		no	DSNG	16,23,30,37,44,51,58,65,72, 79,86,93,100,107,114,121, 128,135,142,149,156,163,170, 177
		no, field 1	DSNG	2
		no, field 2	DSNG	9

Table
Individual Clinical Observations
Test Item (dosage) 1 2 3 4

[redacted] [redacted]

Group/ Sex	Animal Number	Observation	Phase	Day(s)
4/F	P0703	Erythema		
		0: no erythema	DSNG	16,23,30,37,44,51,58,65,72, 79,86,93,100,107,114,121, 128,135,142,149,156,163,170, 177
		0: no erythema, field 1	DSNG	2
		0: no erythema, field 2	DSNG	9
		Oedema		
		0: no oedema	DSNG	16,23,30,37,44,51,58,65,72, 79,86,93,100,107,114,121, 128,135,142,149,156,163,170, 177
		0: no oedema, field 1	DSNG	2
		0: no oedema, field 2	DSNG	9
		Desquamation		
		0: no desquamation	DSNG	16,23,30,37,44,51,58,65,72, 79,86,93,100,107,114,121, 128,135,142,149,156,163,170, 177
		0: no desquamation, field 1	DSNG	2
		0: no desquamation, field 2	DSNG	9

Table
Individual Clinical Observations
Test Item (dosage) 1 2 3 4

[redacted] [redacted]

Group/ Sex	Animal Number	Observation	Phase	Day(s)
4/F	P0703	Fissures		
		0: none	DSNG	16,23,30,37,44,51,58,65,72, 79,86,93,100,107,114,121, 128,135,142,149,156,163,170, 177
		0: none, field 1	DSNG	2
		0: none, field 2	DSNG	9
		Eschar formation		
		no	DSNG	16,23,30,37,44,51,58,65,72, 79,86,93,100,107,114,121, 128,135,142,149,156,163,170, 177
		no, field 1	DSNG	2
		no, field 2	DSNG	9
		Ulcer		
		no	DSNG	16,23,30,37,44,51,58,65,72, 79,86,93,100,107,114,121, 128,135,142,149,156,163,170, 177
		no, field 1	DSNG	2
		no, field 2	DSNG	9

Table
Individual Clinical Observations
Test Item (dosage) 1 2 3 4

[redacted] ----- [redacted]

Group/ Sex	Animal Number	Observation	Phase	Day(s)
4/F	P0704	Erythema		
		0: no erythema	DSNG	16,23,30,37,44,51,58,65,72, 79,86,93,100,107,114,121, 128,135,142,149,156,163,170, 177
		0: no erythema, field 1	DSNG	2
		0: no erythema, field 2	DSNG	9
		Oedema		
		0: no oedema	DSNG	16,23,30,37,44,51,58,65,72, 79,86,93,100,107,114,121, 128,135,142,149,156,163,170, 177
		0: no oedema, field 1	DSNG	2
		0: no oedema, field 2	DSNG	9
		Desquamation		
		0: no desquamation	DSNG	16,23,30,37,44,51,58,65,72, 79,86,93,100,107,114,121, 128,135,142,149,156,163,170, 177
		0: no desquamation, field 1	DSNG	2
		0: no desquamation, field 2	DSNG	9

Table
Individual Clinical Observations
Test Item (dosage) 1 2 3 4

[redacted] ----- [redacted]

Group/ Sex	Animal Number	Observation	Phase	Day(s)
4/F	P0704	Fissures		
		0: none	DSNG	16,23,30,37,44,51,58,65,72, 79,86,93,100,107,114,121, 128,135,142,149,156,163,170, 177
		0: none, field 1	DSNG	2
		0: none, field 2	DSNG	9
		Eschar formation		
		no	DSNG	16,23,30,37,44,51,58,65,72, 79,86,93,100,107,114,121, 128,135,142,149,156,163,170, 177
		no, field 1	DSNG	2
		no, field 2	DSNG	9
		Ulcer		
		no	DSNG	16,23,30,37,44,51,58,65,72, 79,86,93,100,107,114,121, 128,135,142,149,156,163,170, 177
		no, field 1	DSNG	2
		no, field 2	DSNG	9

Table
Individual Clinical Observations
Test Item (dosage) 1 2 3 4

[redacted] ----- [redacted] ----------

Group/ Sex	Animal Number	Observation	Phase	Day(s)
4/F	P0705	Erythema		
		0: no erythema	DSNG	16,23,30,37,44,51,58,65,72, 79,86,93,100,107,114,121, 128,135,142,149,156,163,170, 177
		0: no erythema, field 1	DSNG	2
		0: no erythema, field 2	DSNG	9
		Oedema		
		0: no oedema	DSNG	16,23,30,37,44,51,58,65,72, 79,86,93,100,107,114,121, 128,135,142,149,156,163,170, 177
		0: no oedema, field 1	DSNG	2
		0: no oedema, field 2	DSNG	9
		Desquamation		
		0: no desquamation	DSNG	16,23,30,37,44,51,58,65,72, 79,86,93,100,107,114,121, 128,135,142,149,156,163,170, 177
		0: no desquamation, field 1	DSNG	2
		0: no desquamation, field 2	DSNG	9

Table
Individual Clinical Observations
Test Item (dosage) 1 2 3 4

[redacted] [redacted]

Group/ Sex	Animal Number	Observation	Phase	Day(s)
4/F	P0705	Fissures		
		0: none	DSNG	16,23,30,37,44,51,58,65,72, 79,86,93,100,107,114,121, 128,135,142,149,156,163,170, 177
		0: none, field 1	DSNG	2
		0: none, field 2	DSNG	9
		Eschar formation		
		no	DSNG	16,23,30,37,44,51,58,65,72, 79,86,93,100,107,114,121, 128,135,142,149,156,163,170, 177
		no, field 1	DSNG	2
		no, field 2	DSNG	9
		Ulcer		
		no	DSNG	16,23,30,37,44,51,58,65,72, 79,86,93,100,107,114,121, 128,135,142,149,156,163,170, 177
		no, field 1	DSNG	2
		no, field 2	DSNG	9

Table
Individual Clinical Observations
Test Item (dosage) 1 2 3 4

[redacted] [redacted]

Group/ Sex	Animal Number	Observation	Phase	Day(s)
4/F	P0706	Erythema		
		0: no erythema	DSNG	16,23,30,37,44,51,58,65,72, 79,86,93,100,107,114,121, 128,135,142,149,156,163,170, 177
		0: no erythema, field 1	DSNG	2
		0: no erythema, field 2	DSNG	9
		Oedema		
		0: no oedema	DSNG	16,23,30,37,44,51,58,65,72, 79,86,93,100,107,114,121, 128,135,142,149,156,163,170, 177
		0: no oedema, field 1	DSNG	2
		0: no oedema, field 2	DSNG	9
		Desquamation		
		0: no desquamation	DSNG	16,23,30,37,44,51,58,65,72, 79,86,93,100,107,114,121, 128,135,142,149,156,163,170, 177
		0: no desquamation, field 1	DSNG	2
		0: no desquamation, field 2	DSNG	9

Table
Individual Clinical Observations
Test Item (dosage) 1 2 3 4

[redacted] [redacted]

Group/ Sex	Animal Number	Observation	Phase	Day(s)
4/F	P0706	Fissures		
		0: none	DSNG	16,23,30,37,44,51,58,65,72, 79,86,93,100,107,114,121, 128,135,142,149,156,163,170, 177
		0: none, field 1	DSNG	2
		0: none, field 2	DSNG	9
		Eschar formation		
		no	DSNG	16,23,30,37,44,51,58,65,72, 79,86,93,100,107,114,121, 128,135,142,149,156,163,170, 177
		no, field 1	DSNG	2
		no, field 2	DSNG	9
		Ulcer		
		no	DSNG	16,23,30,37,44,51,58,65,72, 79,86,93,100,107,114,121, 128,135,142,149,156,163,170, 177
		no, field 1	DSNG	2
		no, field 2	DSNG	9

Data in Support of Table 2: Study C, Serum IL-6

[redacted]

Summary of Cytokine Data - Male

tem

[redacted] [redacted]

Group/ Sex	Phase / Day / Session / Name	PIL6 pg/mL Dosing 36 1 03:00	36 2 24:00	85 1 03:00	85 2 24:00	176 1 03:00	176 2 24:00
1/M	Mean	<2.44	<2.44	<2.44	<2.44	<2.75	<2.44
	SD	0.000	0.000	0.000	0.000	0.751	0.000
	N	5	5	6	6	6	5
2/M	Mean	<2.44	<2.44	<3.62	<3.32	<3.04	<2.44
	SD	0.000	0.000	2.355	1.765	1.190	0.000
	N	4	4	4	4	4	4
3/M	Mean	<2.44	<2.44	<4.41	<2.44	<2.45	<2.44
	SD	0.000	0.000	3.196	0.000	0.015	0.000
	N	4	4	4	4	4	4
4/M	Mean	<2.44	<2.48	<4.10	<2.85	<3.05	<2.78
	SD	0.000	0.090	2.622	1.012	1.223	0.575
	N	6	6	6	6	6	6
	Statistics	X5	X5	X5	X5	X5	X5

X5 = Not analyzed (values above/below the limit of quantitation)

Summary of Cytokine Data - Female [redacted]
[redacted] tem [redacted]

Group/ Sex	Phase / Day / Session / Name	PIL6 pg/mL Dosing 36 / 1 / 03:00	36 / 2 / 24:00	85 / 1 / 03:00	85 / 2 / 24:00	176 / 1 / 03:00	176 / 2 / 24:00
1/F	Mean	<2.44	<2.44	<2.44	<2.44	<2.58	<2.44
	SD	0.000	0.000	0.000	0.000	0.335	0.000
	N	6	6	6	6	6	6
2/F	Mean	<2.44	<2.44	<2.44	<2.44	8.39	<2.44
	SD	0.000	0.000	0.000	0.000	5.993	0.000
	N	4	4	4	4	4	4
3/F	Mean	<2.44	<2.44	<2.52	<2.44	<6.16	<2.44
	SD	0.000	0.000	0.160	0.000	2.866	0.000
	N	4	4	4	4	4	4
4/F	Mean	<2.67	<2.46	<5.90	<2.44	<9.28	<2.44
	SD	0.572	0.041	6.697	0.000	10.825	0.000
	N	6	6	6	5	6	6
	Statistics	X5	X5	X5	X5	X5	X5

X5 = Not analyzed (values above/below the limit of quantitation)

Data in Support of Table 2: Study C, Mic (injection site)

Incidence of Microscopic Observations
Terminal Sacrifice
Test Item (dosage) 1 2 3 4

[redacted]

Tissue/ Observation	Group/Sex: Number of Animals:	1/M 4	2/M 4	3/M 4	4/M 4	1/F 4	2/F 4	3/F 4	4/F 4
[redacted],	Number Examined:	4	4	4	4	4	4	4	4
	Unremarkable:	4	4	4	4	4	4	4	4
[redacted],	Number Examined:	4	4	4	4	4	4	4	4
	Unremarkable:	4	4	4	4	4	4	4	4
[redacted]	Number Examined:	4	4	4	4	4	4	4	4
	Unremarkable:	4	4	4	3	4	4	4	4
[redacted]		0	0	0	1	0	0	0	0
[redacted]	Number Examined:	4	4	4	4	4	4	4	4
	Unremarkable:	4	4	4	4	4	4	4	4
[redacted]	Number Examined:	4	4	4	4	4	4	4	4
	Unremarkable:	4	4	4	3	2	4	3	4
[redacted]		0	0	0	0	1	0	0	0
		0	0	0	1	1	0	1	0
Subcutaneous Injection Site	Number Examined:	4	4	4	4	4	4	4	4
	Unremarkable:	4	4	2	1	4	1	1	0
Infiltrate, mononuclear cells, perivascular		0	0	2	3	0	3	3	4

Data in Support of Table 2: Study D, Soft feces

Group	Animal	Liquid Feces (days of occurrence)							Soft Feces (days of occurrence)						
Study month		1	2	3	4	5	6	**T**	1	2	3	4	5	6	**T**
Study days		1 – 30	31-60	61-90	91-120	121-150	151-183		1 – 30	31-60	61-90	91-120	121-150	151-183	
1F	P0403	-	-	-	-	-	158, 159, 160, 161, 162, 163, 164, 165, 166, 167, 168, 169, 170, 171, 172, 173, 174, 175, 176, 177, 178, 180, 181, 182	**24**	-	-	-	-	-	179, 183	**2**
2M	P0102	-	-	-	105, 106, 110, 111, 112, 113	128, 130, 131, 132, 140, 141, 144, 147	153, 159	**16**	-	-	31, 32, 33, 34, 35, 36, 76, 77, 90	91, 94, 96, 97, 98, 100, 101, 102, 104, 104, 107, 108, 109, 114	124, 125, 126, 127, 129, 133, 134, 139, 145, 146	154, 155, 156, 157, 158, 160, 161, 162, 163, 164, 165, 167, 168, 169, 170, 171, 172, 173, 174, 181, 182, 183	**55**

Group	Animal	**Liquid Feces** (days of occurrence)							**Soft Feces** (days of occurrence)						
Study month		1	2	3	4	5	6	**T**	1	2	3	4	5	6	**T**
Study days		1 – 30	31-60	61-90	91-120	121-150	151-183		1 – 30	31-60	61-90	91-120	121-150	151-183	
2F	P0504	-	-	-	-	-	-	**0**	-	45, 46, 47, 55, 56, 57	61, 73	119, 120	121, 122, 127, 128	168, 169, 170, 171, 172, 173, 174, 175, 176, 177, 178, 179 180, 181, 182, 183	**30**
3F	P0602	5, 6, 15, 16, 17, 18, 20	31, 32, 33, 34, 36, 37, 45, 46, 47, 48	82, 83, 84, 85	97, 98, 109, 110, 111, 112, 117	-	-	**28**	21, 22, 23, 30	35, 39, 49, 50, 51, 52, 53, 54, 55, 56, 57, 58, 59, 60,	61, 67, 86, 87, 88, 89, 90	91, 92, 93, 95, 96, 99, 100, 101, 102, 103, 104, 113, 118, 119, 120	121, 122, 123, 124, 125, 126, 127, 128, 138, 139, 140, 141, 142, 143, 144, 145, 146	-	**57**
4M	P0306	17, 18, 19, 25	33, 34, 35, 36, 38, 39, 51, 52, 55,	69, 72, 73, 74, 76, 80	-	135, 136, 137, 140, 150	157, 158, 159, 161, 163, 166, 170, 171, 172, 173, 177	**35**	20, 21, 22, 24, 26, 28, 29, 30	31, 32, 40, 41, 42, 43, 44, 45, 46, 47, 48, 49, 50, 53, 58, 59	64, 66, 67, 68, 70, 71, 75, 77, 78, 79, 81,	116, 117, 118,	121, 122, 125, 126, 127, 128, 129, 130, 131, 132, 133, 134, 138, 139, 141, 142, 143, 144, 145, 146, 147, 148, 149	151, 152, 153, 154, 155, 156, 160, 162, 164, 165, 167, 168, 169, 174, 175, 176, 178, 179, 180, 181, 182, 183	**83**
4F	P0704	6, 7, 8, 9, 10, 11, 14, 15, 16, 17, 18	-	61, 62, 73	110, 11, 112	-	-	**17**	12, 13, 25, 26, 27, 28	37, 38, 39, 40, 41, 42, 43, 44, 45, 46, 47, 48, 54	63, 65, 66, 67, 68, 69, 70, 71, 72	113, 114, 115, 116, 117, 118, 119, 120, 121	-	-	**37**
4F	P0706	33	-	-	-	-	-	**1**	-	34, 35, 36	89, 90	91, 92	-	-	7

Data in Support of Table 2: Study D, Blood eosinophils

Table
Summary of Hematology

[redacted]

Group/ Sex	Phase / Day / Session / Name	Predose 2/3 1	Predose 9/11 1	Predose 16 1 ClinPath	Dosing 85 1 ClinPath	Dosing 182 1 ClinPath	Recovery 84 1 ClinPath
		EOS. %					
1/M	Mean	1.8	1.9	2.1	1.5	1.8	0.9
	SD	0.97	1.24	1.36	0.88	1.36	0.71
	N	6	6	6	6	6	2
2/M	Mean	1.7	2.3	1.9	1.1	1.0	-
	SD	0.97	0.43	0.59	0.55	0.57	-
	N	4	4	4	4	4	-
3/M	Mean	1.5	1.7	1.9	0.6	0.6	-
	SD	1.26	1.09	1.18	0.56	0.48	-
	N	4	4	4	4	4	-
4/M	Mean	1.2	1.4	1.8	0.6	0.6	0.4
	SD	0.85	0.92	1.44	0.75	0.67	0.35
	N	6	6	6	6	6	2
	Statistics	A	A	A	A	AT	X7

A = ANOVA and Dunnett's
T = Rank-transformed data
X7 = Not analyzed (mean of actual group sizes too small)

Table
Summary of Hematology

[redacted]

Group/ Sex	Phase / Day / Session / Name	Predose 2/3 1	Predose 9/11 1	Predose 16 1 ClinPath	Dosing 85 1 ClinPath	Dosing 182 1 ClinPath	Recovery 83 1 ClinPath
		EOS. %					
1/F	Mean	1.2	1.3	1.6	1.2	1.1	1.4
	SD	0.50	0.95	0.83	0.50	0.50	1.20
	N	6	6	6	6	6	2
2/F	Mean	2.1	1.6	2.4	0.5	1.1	-
	SD	1.67	0.88	1.53	0.43	0.91	-
	N	4	4	4	4	4	-
3/F	Mean	1.1	1.0	1.5	0.7	0.4	-
	SD	0.74	0.46	0.50	0.65	0.51	-
	N	4	4	4	4	4	-
4/F	Mean	3.5*	2.4	3.7	0.9	1.0	0.4
	SD	1.80	0.82	2.88	0.80	0.86	0.57
	N	6	6	6	6	6	2
	Statistics	A	A	A	A	A	X7

* P<=0.05
** P<=0.01
*** P<=0.001
A = ANOVA and Dunnett's
X7 = Not analyzed (mean of actual group sizes too small)

Data in Support of Table 2: Study E, Serum globulin

Summary of Clinical Chemistry

[redacted]

		GLOBgdL. g/dL					
	Phase	Predose				Dosing	
Group/ Sex	Day Session Name	8 1 Clin Path	15 1 Clin Path	72 1 Clin Path	78 1 Clin Path	15 2 0 Hour	32 1 Clin Path
1/M	Mean	2.94	2.86	-	-	3.19	3.13
	SD	0.085	0.372	-	-	0.104	0.123
	N	3	3	-	-	3	3
2/M	Mean	2.30*	2.45	-	-	2.75	3.47
	SD	0.226	0.203	-	-	0.247	0.368
	N	2	3	-	-	3	2
3/M	Mean	3.09	3.05	-	-	3.73	3.80
	SD	0.284	0.185	-	-	0.459	0.699
	N	3	3	-	-	3	3
	Statistics	A	A	-	-	A	A

* P<=0.05
** P<=0.01
*** P<=0.001
A = ANOVA and Dunnett's

Summary of Clinical Chemistry

[redacted]

Group/ Sex	Phase / Day / Session / Name	GLOBgdL. g/dL Dosing 60 / 1 / Clin Path	116 / 1 / Clin Path	138 / 1 / Check	163 / 1 / moribund	186 / 1 / Clin Path
1/M	Mean	2.95	3.18	-	-	3.11
	SD	0.413	0.091	-	-	0.085
	N	3	3	-	-	3
2/M	Mean	2.86	3.66	-	-	3.61
	SD	0.262	0.131	-	-	0.114
	N	3	3	-	-	3
3/M	Mean	4.03*	4.02*	2.91	3.38	4.22**
	SD	0.628	0.521	-	-	0.417
	N	3	3	1	1	2
	Statistics	A	A	X	X	A

* P<=0.05
** P<=0.01
*** P<=0.001
A = ANOVA and Dunnett's
X = No analysis performed

Data in Support of Table 2: Study F, Body weight

[redacted]

[redacted]

Group/ Sex	Phase	PRED		DSNG			
	Day	2	5	1	5	8	12
		Data Presented in "kg"					
1/M	Mean	5.9	5.9	5.9	6.0	5.9	6.1
	SD	0.92	0.89	0.96	0.90	0.94	1.01
	N	6	6	6	6	6	6
2/M	Mean	7.0	7.0	7.0	7.1	7.0	7.2
	SD	0.85	0.81	0.81	0.80	0.83	0.87
	N	4	4	4	4	4	4
3/M	Mean	6.5	6.5	6.5	6.5	6.6	6.7
	SD	0.53	0.58	0.60	0.58	0.62	0.62
	N	4	4	4	4	4	4
4/M	Mean	7.4	7.4	7.4	7.4	7.5	7.6
	SD	1.06	1.09	1.07	1.11	1.14	1.20
	N	6	6	6	6	6	6
	Statistics	A	A	A	A	A	A

A = ANOVA and Dunnett's

Table
Summary of Body Weight

[redacted]

Group/Sex	Phase / Day	DSNG 15	DSNG 19	DSNG 22	DSNG 26	DSNG 29	DSNG 33
		Data Presented in "kg"					
1/M	Mean	6.0	6.1	6.2	5.9	6.1	6.2
	SD	0.96	0.92	1.01	0.94	0.92	0.90
	N	6	6	6	6	6	6
2/M	Mean	7.2	7.2	7.2	7.1	7.2	7.2
	SD	0.99	0.99	1.11	0.99	1.04	1.10
	N	4	4	4	4	4	4
3/M	Mean	6.6	6.7	6.8	6.6	6.7	6.9
	SD	0.74	0.76	0.78	0.73	0.82	0.84
	N	4	4	4	4	4	4
4/M	Mean	7.6	7.8	7.8	7.6	7.7	7.8
	SD	1.20	1.21	1.24	1.24	1.24	1.25
	N	6	6	6	6	6	6
	Statistics	A	A	A	A	A	A

A = ANOVA and Dunnett's

Table
Summary of Body Weight

[redacted]

Group/Sex	Phase / Day	DSNG 36	40	43	47	50	54
		Data Presented in "kg"					
1/M	Mean	6.2	6.3	6.2	6.3	6.2	6.3
	SD	0.95	0.95	0.94	0.91	0.92	0.92
	N	6	6	6	6	6	6
2/M	Mean	7.3	7.4	7.4	7.4	7.4	7.4
	SD	1.16	1.14	1.13	1.18	1.14	1.24
	N	4	4	4	4	4	4
3/M	Mean	7.0	7.1	7.1	7.1	7.2	7.3
	SD	0.87	0.87	0.83	0.90	0.87	0.95
	N	4	4	4	4	4	4
4/M	Mean	7.9	7.9	8.0	8.0	8.0	8.0
	SD	1.30	1.29	1.28	1.28	1.34	1.32
	N	6	6	6	6	6	6
	Statistics	A	A	A	A	A	A

A = ANOVA and Dunnett's

Table
Summary of Body Weight

[redacted]

Group/ Sex	Phase / Day	Data Presented in "kg" DSNG 57	61	64	68	71	75
1/M	Mean	6.3	6.3	6.4	6.3	6.4	6.4
	SD	0.90	0.93	0.94	0.89	0.93	0.88
	N	6	6	6	6	6	6
2/M	Mean	7.4	7.4	7.5	7.5	7.6	7.6
	SD	1.16	1.16	1.16	1.26	1.33	1.24
	N	4	4	4	4	4	4
3/M	Mean	7.3	7.3	7.4	7.4	7.5	7.4
	SD	0.95	1.03	1.03	1.07	1.11	1.10
	N	4	4	4	4	4	4
4/M	Mean	8.0	8.1	8.1	8.1	8.2	8.1
	SD	1.32	1.29	1.32	1.38	1.38	1.45
	N	6	6	6	6	6	6
	Statistics	A	A	A	A	A	A

A = ANOVA and Dunnett's

Table
Summary of Body Weight

[redacted]

Group/Sex	Phase / Day	DSNG 78	DSNG 82	DSNG 85	DSNG 89	DSNG 91	RECO 1
		Data Presented in "kg"					
1/M	Mean	6.4	6.4	6.4	6.5	6.4	6.2
	SD	0.96	0.97	0.96	0.93	0.88	1.48
	N	6	6	6	6	6	2
2/M	Mean	7.6	7.6	7.6	7.7	7.7	-
	SD	1.26	1.21	1.31	1.26	1.17	-
	N	4	4	4	4	4	-
3/M	Mean	7.5	7.5	7.5	7.6	7.5	-
	SD	1.11	1.11	1.11	1.11	1.08	-
	N	4	4	4	4	4	-
4/M	Mean	8.2	8.2	8.1	8.2	8.0	7.2
	SD	1.50	1.47	1.52	1.51	1.50	1.98
	N	6	6	6	6	6	2
	Statistics	A	A	A	A	A	X7

A = ANOVA and Dunnett's
X7 = Not analyzed (mean of actual group sizes too small)

Table
Summary of Body Weight

[redacted]

Group/ Sex	Phase / Day	RECO 5	RECO 8	RECO 12	RECO 15	RECO 19	RECO 22
		Data Presented in "kg"					
1/M	Mean	6.3	6.3	6.4	6.3	6.4	6.3
	SD	1.48	1.63	1.63	1.48	1.63	1.70
	N	2	2	2	2	2	2
4/M	Mean	7.5	7.5	7.5	7.4	7.5	7.5
	SD	1.77	1.77	1.91	1.91	1.91	1.98
	N	2	2	2	2	2	2
	Statistics	X7	X7	X7	X7	X7	X7

X7 = Not analyzed (mean of actual group sizes too small)

Table
Summary of Body Weight

[redacted]

Group/ Sex	Phase / Day	RECO 26	RECO 29	RECO 33	RECO 36	RECO 40	RECO 43
		Data Presented in "kg"					
1/M	Mean	6.4	6.4	6.4	6.4	6.4	6.4
	SD	1.63	1.77	1.70	1.84	1.77	1.63
	N	2	2	2	2	2	2
4/M	Mean	7.6	7.6	7.7	7.8	7.8	7.8
	SD	1.98	1.98	1.98	1.98	1.98	2.12
	N	2	2	2	2	2	2
	Statistics	X7	X7	X7	X7	X7	X7

X7 = Not analyzed (mean of actual group sizes too small)

Table
Summary of Body Weight

[redacted]

Group/ Sex	Phase / Day	RECO 47	RECO 50	RECO 54	RECO 57	RECO 61	RECO 64
		Data Presented in "kg"					
1/M	Mean	6.3	6.4	6.3	6.4	6.4	6.4
	SD	1.70	1.63	1.70	1.77	1.70	1.70
	N	2	2	2	2	2	2
4/M	Mean	7.8	7.9	7.8	7.9	7.9	8.0
	SD	2.05	2.05	2.12	2.26	2.26	2.26
	N	2	2	2	2	2	2
	Statistics	X7	X7	X7	X7	X7	X7

X7 = Not analyzed (mean of actual group sizes too small)

Table
Summary of Body Weight

[redacted]

Group/Sex	Phase / Day	RECO 68	71	75	78	82	84
		Data Presented in "kg"					
1/M	Mean	6.4	6.4	6.4	6.4	6.3	6.4
	SD	1.70	1.63	1.63	1.63	1.70	1.63
	N	2	2	2	2	2	2
4/M	Mean	8.0	8.1	8.1	8.1	8.1	8.1
	SD	2.19	2.40	2.33	2.40	2.33	2.33
	N	2	2	2	2	2	2
	Statistics	X7	X7	X7	X7	X7	X7

X7 = Not analyzed (mean of actual group sizes too small)

[redacted]

Table
Summary of Body Weight

[redacted]

Group/Sex	Phase / Day	PRED 2	PRED 5	DSNG 1	DSNG 5	DSNG 8	DSNG 12
		Data Presented in "kg"					
1/F	Mean	4.4	4.3	4.3	4.3	4.3	4.4
	SD	0.96	0.95	0.95	0.91	0.91	0.95
	N	6	6	6	6	6	6
2/F	Mean	4.4	4.4	4.4	4.4	4.4	4.4
	SD	0.99	1.02	1.05	1.05	1.01	1.06
	N	4	4	4	4	4	4
3/F	Mean	3.5	3.5	3.5	3.5	3.5	3.5
	SD	0.57	0.57	0.57	0.57	0.56	0.53
	N	4	4	4	4	4	4
4/F	Mean	5.5	5.5	5.5	5.4	5.4	5.4
	SD	1.62	1.64	1.60	1.58	1.56	1.52
	N	6	6	6	6	6	6
	Statistics	AT	A	A	A	A	A

A = ANOVA and Dunnett's
T = Rank-transformed data

Table
Summary of Body Weight

[redacted]

Group/ Sex	Phase / Day	DSNG 15	DSNG 19	DSNG 22	DSNG 26	DSNG 29	DSNG 33
		Data Presented in "kg"					
1/F	Mean	4.4	4.4	4.3	4.3	4.3	4.4
	SD	0.91	0.90	0.87	0.89	0.92	0.96
	N	6	6	6	6	6	6
2/F	Mean	4.5	4.5	4.5	4.5	4.5	4.5
	SD	1.08	1.14	1.12	1.08	1.08	1.10
	N	4	4	4	4	4	4
3/F	Mean	3.6	3.6	3.6	3.5	3.6	3.6
	SD	0.57	0.50	0.48	0.47	0.45	0.45
	N	4	4	4	4	4	4
4/F	Mean	5.4	5.4	5.4	5.4	5.3	5.3
	SD	1.50	1.50	1.49	1.46	1.45	1.47
	N	6	6	6	6	6	6
	Statistics	A	A	A	A	A	A

A = ANOVA and Dunnett's

Table
Summary of Body Weight

[redacted]

Group/ Sex	Phase / Day	Data Presented in "kg" DSNG 36	40	43	47	50	54
1/F	Mean	4.4	4.4	4.4	4.4	4.4	4.5
	SD	0.98	0.94	0.89	0.87	0.90	0.90
	N	6	6	6	6	6	6
2/F	Mean	4.6	4.6	4.6	4.6	4.6	4.7
	SD	1.14	1.14	1.16	1.16	1.18	1.23
	N	4	4	4	4	4	4
3/F	Mean	3.6	3.6	3.6	3.7	3.6	3.6
	SD	0.40	0.40	0.42	0.50	0.43	0.40
	N	4	4	4	4	4	4
4/F	Mean	5.3	5.3	5.3	5.3	5.3	5.3
	SD	1.44	1.42	1.41	1.39	1.44	1.36
	N	6	6	6	6	6	6
	Statistics	A	A	A	A	A	A

A = ANOVA and Dunnett's

Table
Summary of Body Weight

[redacted]

Group/ Sex	Phase / Day	DSNG 57	61	64	68	71	75
		Data Presented in "kg"					
1/F	Mean	4.4	4.5	4.5	4.5	4.6	4.6
	SD	0.93	0.94	0.96	1.00	1.00	1.02
	N	6	6	6	6	6	6
2/F	Mean	4.7	4.7	4.8	4.7	4.8	4.8
	SD	1.20	1.25	1.25	1.21	1.27	1.27
	N	4	4	4	4	4	4
3/F	Mean	3.6	3.6	3.6	3.7	3.7	3.7
	SD	0.36	0.32	0.36	0.33	0.33	0.38
	N	4	4	4	4	4	4
4/F	Mean	5.2	5.2	5.2	5.3	5.3	5.3
	SD	1.41	1.38	1.38	1.34	1.35	1.33
	N	6	6	6	6	6	6
	Statistics	A	A	A	A	AT	A

A = ANOVA and Dunnett's
T = Rank-transformed data

Table
Summary of Body Weight

[redacted]

Group/Sex	Phase / Day	DSNG 78	DSNG 82	DSNG 85	DSNG 89	DSNG 91	RECO 1
		Data Presented in "kg"					
1/F	Mean	4.6	4.5	4.7	4.6	4.6	4.5
	SD	1.05	1.04	1.04	1.03	1.00	1.41
	N	6	6	6	6	6	2
2/F	Mean	4.8	4.9	4.9	4.9	4.8	-
	SD	1.25	1.31	1.31	1.33	1.29	-
	N	4	4	4	4	4	-
3/F	Mean	3.7	3.7	3.7	3.6	3.5	-
	SD	0.38	0.38	0.40	0.36	0.36	-
	N	4	4	4	4	4	-
4/F	Mean	5.2	5.2	5.2	5.2	5.1	3.7
	SD	1.36	1.33	1.33	1.34	1.36	0.35
	N	6	6	6	6	6	2
	Statistics	A	A	A	AT	AT	X7

A = ANOVA and Dunnett's
T = Rank-transformed data
X7 = Not analyzed (mean of actual group sizes too small)

Table
Summary of Body Weight

[redacted]

Group/Sex	Phase / Day	RECO 5	RECO 8	RECO 12	RECO 15	RECO 19	RECO 22
		Data Presented in "kg"					
1/F	Mean	4.5	4.5	4.5	4.5	4.6	4.6
	SD	1.41	1.48	1.56	1.56	1.48	1.48
	N	2	2	2	2	2	2
4/F	Mean	3.8	3.7	3.7	3.7	3.7	3.7
	SD	0.35	0.28	0.35	0.35	0.42	0.49
	N	2	2	2	2	2	2
	Statistics	X7	X7	X7	X7	X7	X7

X7 = Not analyzed (mean of actual group sizes too small)

Table
Summary of Body Weight

[redacted]

Group/ Sex	Phase / Day	RECO 26	RECO 29	RECO 33	RECO 36	RECO 40	RECO 43
		Data Presented in "kg"					
1/F	Mean	4.5	4.5	4.6	4.7	4.7	4.6
	SD	1.63	1.48	1.48	1.63	1.63	1.70
	N	2	2	2	2	2	2
4/F	Mean	3.8	3.7	3.8	3.9	3.8	3.7
	SD	0.49	0.57	0.64	0.64	0.64	0.57
	N	2	2	2	2	2	2
	Statistics	X7	X7	X7	X7	X7	X7

X7 = Not analyzed (mean of actual group sizes too small)

Table
Summary of Body Weight

[redacted]

Group/ Sex	Phase / Day	RECO 47	RECO 50	RECO 54	RECO 57	RECO 61	RECO 64
		Data Presented in "kg"					
1/F	Mean	4.6	4.7	4.6	4.7	4.7	4.8
	SD	1.70	1.77	1.70	1.77	1.77	1.77
	N	2	2	2	2	2	2
4/F	Mean	3.7	3.8	3.8	3.8	3.8	3.8
	SD	0.57	0.57	0.71	0.57	0.71	0.71
	N	2	2	2	2	2	2
	Statistics	X7	X7	X7	X7	X7	X7

X7 = Not analyzed (mean of actual group sizes too small)

Table
Summary of Body Weight

[redacted]

Group/Sex	Phase / Day	RECO 68	RECO 71	RECO 75	RECO 78	RECO 82	RECO 84
		Data Presented in "kg"					
1/F	Mean	4.8	4.8	4.9	4.9	4.8	4.8
	SD	1.84	1.84	1.91	1.91	1.84	1.91
	N	2	2	2	2	2	2
4/F	Mean	3.9	3.9	3.9	3.8	3.8	3.8
	SD	0.78	0.78	0.78	0.71	0.71	0.71
	N	2	2	2	2	2	2
	Statistics	X7	X7	X7	X7	X7	X7

X7 = Not analyzed (mean of actual group sizes too small)

Data in Support of Table 2: Study F, Mic (testis)

PATHOLOGY REPORT
SUMMARY TABLES [redacted]

TEST ITEM : [redacted]
TEST SYSTEM : M. CYNOMOLGUS, 13-week, Gavage [redacted]
SPONSOR : [redacted]

SUMMARY INCIDENCE OF GRADINGS BY ORGAN/GROUP/SEX
STATUS AT NECROPSY: K0
FINDINGS IN ALL ANIMALS ON STUDY

DOSE GROUP:	1		2		3		4	
SEX :	M	F	M	F	M	F	M	F
NO.ANIMALS:	4	4	4	4	4	4	4	4
SPLEEN :	4	4	4	4	4	4	4	4
- [redacted]								
GRADE 1 :	-	2	-	1	1	3	-	1
GRADE 2 :	2	2	3	1	-	-	3	2
GRADE 3 :	2	-	1	2	3	1	1	1
TOTAL AFFECTED :	4	4	4	4	4	4	4	4
MEAN GRADE/TISS.AFF.:	2.5	1.5	2.3	2.3	2.5	1.5	2.3	2.0
STOMACH :	4	4	4	4	4	4	4	4
- [redacted]								
GRADE 3 :	-	-	-	-	-	1	-	-
TOTAL AFFECTED :	-	-	-	-	-	1	-	-
MEAN GRADE/TISS.AFF.:	-	-	-	-	-	3.0	-	-
- [redacted]								
GRADE 1 :	-	-	-	-	-	-	1	-
TOTAL AFFECTED :	-	-	-	-	-	-	1	-
MEAN GRADE/TISS.AFF.:	-	-	-	-	-	-	1.0	-
TESTES :	4	-	4	-	4	-	4	-
- Degeneration Tubular								
GRADE 1 :	-	-	-	-	-	-	1	-
GRADE 2 :	-	-	-	-	-	-	1	-
TOTAL AFFECTED :	-	-	-	-	-	-	2	-
MEAN GRADE/TISS.AFF.:	-	-	-	-	-	-	1.5	-
- Depletion,Spermatids								
GRADE 2 :	-	-	-	-	-	-	2	-
GRADE 4 :	-	-	-	-	-	-	2	-
TOTAL AFFECTED :	-	-	-	-	-	-	4	-
MEAN GRADE/TISS.AFF.:	-	-	-	-	-	-	3.0	-

PATHOLOGY REPORT
SUMMARY TABLES [redacted]

TEST ITEM : [redacted]
TEST SYSTEM : M. CYNOMOLGUS, 13-week, Gavage
SPONSOR : [redacted]

[redacted]

SUMMARY INCIDENCE OF GRADINGS BY ORGAN/GROUP/SEX
STATUS AT NECROPSY: K0
FINDINGS IN ALL ANIMALS ON STUDY

DOSE GROUP:	1		2		3		4	
SEX :	M	F	M	F	M	F	M	F
NO.ANIMALS:	4	4	4	4	4	4	4	4
TESTES CONT'D.	4	-	4	-	4	-	4	-
- Dilation, Tubular								
GRADE 2 :	-	-	-	-	1	-	3	-
TOTAL AFFECTED :	-	-	-	-	1	-	3	-
MEAN GRADE/TISS.AFF.:	-	-	-	-	2.0	-	2.0	-
- Giant Cell, Multinuc								
GRADE 2 :	-	-	-	-	-	-	1	-
GRADE 3 :	-	-	-	-	-	-	1	-
GRADE 4 :	-	-	-	-	-	-	2	-
TOTAL AFFECTED :	-	-	-	-	-	-	4	-
MEAN GRADE/TISS.AFF.:	-	-	-	-	-	-	3.3	-
THYMUS :	4	4	4	4	4	4	4	4
- [redacted])								
GRADE 1 :	-	1	-	-	-	-	-	2
GRADE 2 :	-	-	-	-	1	2	1	1
TOTAL AFFECTED :	-	1	-	-	1	2	1	3
MEAN GRADE/TISS.AFF.:	-	1.0	-	-	2.0	2.0	2.0	1.3
- [redacted]								
GRADE 1 :	2	2	1	3	1	2	-	2
GRADE 2 :	1	-	-	1	1	2	1	1
GRADE 3 :	1	1	2	-	2	-	2	-
GRADE 4 :	-	-	-	-	-	-	1	-
TOTAL AFFECTED :	4	3	3	4	4	4	4	3
MEAN GRADE/TISS.AFF.:	1.8	1.7	2.3	1.3	2.3	1.5	3.0	1.3

Data in Support of Table 2: Study F, Mac (testis)

TEST ITEM : [redacted]
TEST SYSTEM : M. CYNOMOLGUS, 13-week, Gavage [redacted]
SPONSOR : [redacted]

CORRELATION TABLE: NECROPSY - MICROSCOPY DOSE GROUP 4, MALE

NECROPSY OBSERVATION	CORRESPONDING MICROSCOPIC FINDING
	ANIMAL NO: 28666
STOMACH	
- 01: [redacted]	[redacted]
	ANIMAL NO: 28678
TESTES	
- 01: Abnormal consistency; bilateral; soft.	- Depletions, tubular, bilateral, grade 4. Giant cell, multinucleate, bilateral, grade 3.
- 02: Discoloration; bilateral; pale.	- Depletions, tubular, bilateral, grade 4. Giant cell, multinucleate, bilateral, grade 3.
	ANIMAL NO: 28702
CECUM	
- 01: [redacted]	[redacted]

Data in Support of Table 2: Study F, Organ weight (liver)

[redacted]

Table
Summary of Organ Weights and Organ Weight Ratios
Terminal Sacrifice

[redacted]

Group/ Sex		Terminal Body Weight (g)	[redacted] Unadjusted (g)	[redacted] Body Weight (%)	[redacted] Brain Weight (%)	Liver Unadjusted (g)	Liver Body Weight (%)	Liver Brain Weight (%)
1/M	Mean	6430	12.295	0.1915	16.5450	107.190	1.6650	144.2917
	SD	855.7	1.5438	0.00909	2.94698	15.8933	0.06387	28.43016
	N	4	4	4	4	4	4	4
2/M	Mean	7494	13.400	0.1810	18.8472	131.154	1.7701	185.8638
	SD	1224.0	2.8644	0.04239	2.17954	18.3196	0.28001	13.27292
	N	4	4	4	4	4	4	4
3/M	Mean	7390	12.345	0.1681	16.8131	124.801	1.6896	169.6081
	SD	1089.4	1.2045	0.01159	1.33899	19.2346	0.08633	20.78808
	N	4	4	4	4	4	4	4
4/M	Mean	8349	14.019	0.1708	19.5813	170.953**	2.0850	238.7848**
	SD	1387.2	1.2103	0.02555	2.46579	24.8973	0.45906	40.09340
	N	4	4	4	4	4	4	4
	Statistics	A	A	AT	A	A	AT	A

* P<=0.05
** P<=0.01
*** P<=0.001
A = ANOVA and Dunnett's
T = Rank-transformed data

[redacted]

Table
Summary of Organ Weights and Organ Weight Ratios
Terminal Sacrifice

[redacted]

Group/ Sex		Terminal Body Weight (g)	[redacted] Unadjusted (g)	Body Weight (%)	Brain Weight (%)	Liver Unadjusted (g)	Liver Body Weight (%)	Liver Brain Weight (%)
1/F	Mean	4494	8.535	0.1958	13.1860	80.238	1.8186	124.2275
	SD	1059.0	1.1675	0.04374	1.59040	10.7244	0.18764	17.26357
	N	4	4	4	4	4	4	4
2/F	Mean	4686	8.476	0.1817	12.8794	85.672	1.8547	130.5795
	SD	1272.3	2.1445	0.01037	2.28856	18.8309	0.17653	19.11878
	N	4	4	4	4	4	4	4
3/F	Mean	3483	7.043	0.2029	11.1444	70.111	2.0114	110.9191
	SD	354.8	0.9250	0.02662	1.49986	8.2590	0.07821	12.69242
	N	4	4	4	4	4	4	4
4/F	Mean	5781	9.705	0.1687	14.4552	121.241*	2.0941	180.2153**
	SD	940.1	1.3798	0.01231	1.20268	21.7929	0.09853	21.37740
	N	4	4	4	4	4	4	4
	Statistics	AT	A	A	A	AT	A	A

* P<=0.05
** P<=0.01
*** P<=0.001
A = ANOVA and Dunnett's
T = Rank-transformed data

Data in Support of Table 2: Study F, Organ weight (spleen)

[redacted]

Table
Summary of Organ Weights and Organ Weight Ratios
Terminal Sacrifice

[redacted]

Group/ Sex		Terminal Body Weight (g)	Spleen Unadjusted (g)	Spleen Body Weight (%)	Spleen Brain Weight (%)	[redacted] Unadjusted (g)	[redacted] Body Weight (%)	[redacted] Brain Weight (%)
1/M	Mean	6430	9.015	0.1394	12.1617	20.767	0.3186	28.1088
	SD	855.7	1.7180	0.00982	2.91057	5.6709	0.05206	8.89694
	N	4	4	4	4	4	4	4
2/M	Mean	7494	10.573	0.1428	15.2089	18.831	0.2577	26.7739
	SD	1224.0	2.4436	0.03465	4.08916	2.3765	0.05788	2.56969
	N	4	4	4	4	4	4	4
3/M	Mean	7390	11.556	0.1582	15.7183	24.440	0.3330	33.3656
	SD	1089.4	0.9718	0.01988	0.45844	3.2526	0.04032	4.66006
	N	4	4	4	4	4	4	4
4/M	Mean	8349	13.583**	0.1662	18.9845*	13.925	0.1666**	19.4089
	SD	1387.2	1.7010	0.03319	3.03370	2.5849	0.00815	3.65674
	N	4	4	4	4	4	4	4
	Statistics	A	A	A	A	A	A	A

* P<=0.05
** P<=0.01
*** P<=0.001
A = ANOVA and Dunnett's

Table
Summary of Organ Weights and Organ Weight Ratios
Terminal Sacrifice

[redacted]

Group/ Sex		Terminal Body Weight (g)	Spleen Unadjusted (g)	Spleen Body Weight (%)	Spleen Brain Weight (%)	[redacted] Unadjusted (g)	[redacted] Body Weight (%)	[redacted] Brain Weight (%)
1/F	Mean	4494	6.695	0.1501	10.2636	3.915	0.0890	5.9977
	SD	1059.0	2.2024	0.03425	2.89254	1.3075	0.03026	1.74372
	N	4	4	4	4	4	4	4
2/F	Mean	4686	6.676	0.1420	10.1120	2.896	0.0600	4.3575
	SD	1272.3	2.3119	0.03086	2.96487	1.1995	0.01216	1.55361
	N	4	4	4	4	4	4	4
3/F	Mean	3483	6.074	0.1744	9.6101	1.983	0.0583	3.1578*
	SD	354.8	1.2478	0.03262	1.95634	0.6412	0.02110	1.08514
	N	4	4	4	4	4	4	4
4/F	Mean	5781	9.523	0.1660	14.2360	3.921	0.0685	5.8702
	SD	940.1	1.7493	0.02571	2.41932	0.7482	0.01388	1.17839
	N	4	4	4	4	4	4	4
	Statistics	AT	A	A	A	A	A	A

* P<=0.05
** P<=0.01
*** P<=0.001
A = ANOVA and Dunnett's
T = Rank-transformed data

Data in Support of Table 2: Study F, Vomiting

Test Item (dosage) 1 2 3 4

[redacted]

Group/ Sex	Animal Number	Observation	Phase	Day(s)
1/M	28039M	Post Dosing Status		
		normal, before dosing	DSNG	1-91
		normal, postdosing (1.observation)	DSNG	1-6,8-14,16-25,27-30,32-34, 36-72,74-80,82-89,91
		normal, postdosing (2.observation)	DSNG	1-91
		normal, postdosing (3.observation)	DSNG	1-91
		Mouth/ vomiting		
		emesis of mash, postdosing (1.observation) /during dosing	DSNG	7
		emesis of liquid, clear, postdosing (1.observation)	DSNG	15,26
		emesis of liquid, foamy, postdosing (1.observation)	DSNG	31,90
		emesis of mash, postdosing (1.observation)	DSNG	35,73,81
1/M	28040M	Post Dosing Status		
		normal, before dosing	DSNG	1-91
		normal, postdosing (1.observation)	DSNG	1-91
		normal, postdosing (2.observation)	DSNG	1-91
		normal, postdosing (3.observation)	DSNG	1-91

Table
Individual Clinical Observations
Test Item (dosage) 1 2 3 4

[redacted]

Group/ Sex	Animal Number	Observation	Phase	Day(s)
1/M	28797M	Post Dosing Status		
		normal, before dosing	DSNG	1-91
		normal, postdosing (1.observation)	DSNG	1-54,56-91
		normal, postdosing (2.observation)	DSNG	1-91
		normal, postdosing (3.observation)	DSNG	1-91
		Mouth/ vomiting		
		emesis of liquid, foamy, postdosing (1.observation)	DSNG	55
1/M	28801M	Post Dosing Status		
		normal, before dosing	DSNG	1-92
		normal, postdosing (1.observation)	DSNG	1-92
		normal, postdosing (2.observation)	DSNG	1-92
		normal, postdosing (3.observation)	DSNG	1-92

Table
Individual Clinical Observations
Test Item (dosage) 1 2 3 4

[redacted]

Group/ Sex	Animal Number	Observation	Phase	Day(s)
1/M	28804M	Post Dosing Status		
		normal, before dosing	DSNG	1-92
		normal, postdosing (1.observation)	DSNG	1,3-10,12,13,15-45,47-53, 56-62,64,66-71,73-78,82,84, 85,87,89-92
		normal, postdosing (2.observation)	DSNG	1-92
		normal, postdosing (3.observation)	DSNG	1-92
		Mouth/ vomiting		
		emesis of liquid, clear, postdosing (1.observation)	DSNG	2,11,54,55,72,79,80,86
		emesis of liquid, white, postdosing (1.observation)	DSNG	14
		emesis of mash, postdosing (1.observation)	DSNG	46,63,81
		emesis of liquid, foamy, postdosing (1.observation)	DSNG	65,83,88
1/M	28808M	Post Dosing Status		
		normal, before dosing	DSNG	1-91
		normal, postdosing (1.observation)	DSNG	1-91
		normal, postdosing (2.observation)	DSNG	1-91
		normal, postdosing (3.observation)	DSNG	1-91

Table
Individual Clinical Observations
Test Item (dosage) 1 2 3 4

[redacted]

Group/ Sex	Animal Number	Observation	Phase	Day(s)
2/M	28680M	Post Dosing Status		
		normal, before dosing	DSNG	1-91
		normal, postdosing (1.observation)	DSNG	1-91
		normal, postdosing (2.observation)	DSNG	1-91
		normal, postdosing (3.observation)	DSNG	1-91
2/M	28684M	Post Dosing Status		
		normal, before dosing	DSNG	1-91
		normal, postdosing (1.observation)	DSNG	1-91
		normal, postdosing (2.observation)	DSNG	1-91
		normal, postdosing (3.observation)	DSNG	1-91
2/M	28802M	Post Dosing Status		
		normal, before dosing	DSNG	1-92
		normal, postdosing (1.observation)	DSNG	1-92
		normal, postdosing (2.observation)	DSNG	1-92
		normal, postdosing (3.observation)	DSNG	1-92

Table
Individual Clinical Observations
Test Item (dosage) 1 2 3 4

[redacted]

Group/ Sex	Animal Number	Observation	Phase	Day(s)
2/M	28806M	Post Dosing Status		
		normal, before dosing	DSNG	1-92
		normal, postdosing (1.observation)	DSNG	1-92
		normal, postdosing (2.observation)	DSNG	1-92
		normal, postdosing (3.observation)	DSNG	1-92

Table
Individual Clinical Observations
Test Item (dosage) 1 2 3 4

[redacted]

Group/ Sex	Animal Number	Observation	Phase	Day(s)
3/M	28698M	Post Dosing Status		
		normal, before dosing	DSNG	1-92
		normal, postdosing (1.observation)	DSNG	1-8,10-13,17,19-23,29,30,34, 35,37,45-48,52-55,57,61, 67-69,71,72,75,76,83-85,89, 91,92
		normal, postdosing (2.observation)	DSNG	1-92
		normal, postdosing (3.observation)	DSNG	1-92

Table
Individual Clinical Observations
Test Item (dosage) 1 2 3 4

[redacted]

Group/ Sex	Animal Number	Observation	Phase	Day(s)
3/M	28698M	Mouth/ vomiting		
		emesis of liquid, clear, postdosing (1.observation)	DSNG	9,49,86-88
		emesis of mash, postdosing (1.observation)	DSNG	14,31,32,38,39,50,90
		emesis of liquid, white, postdosing (1.observation)	DSNG	15,16,18,36,40,56,64,70
		emesis of liquid, foamy, postdosing (1.observation)	DSNG	24-28,33,41-44,51,58-60,65, 66,77-82
		emesis of liquid during dosing, postdosing (1.observation)	DSNG	56,86
		emesis of foam, white, postdosing (1.observation)	DSNG	62,63
		emesis of liquid during dosing, white, postdosing (1.observation)	DSNG	70,82
		emesis of liquid, cloudy, postdosing (1.observation)	DSNG	73,74
3/M	28703M	Post Dosing Status		
		normal, before dosing	DSNG	1-92
		normal, postdosing (1.observation)	DSNG	1-92
		normal, postdosing (2.observation)	DSNG	1-92
		normal, postdosing (3.observation)	DSNG	1-92

Table
Individual Clinical Observations
Test Item (dosage) 1 2 3 4

[redacted]

Group/ Sex	Animal Number	Observation	Phase	Day(s)
3/M	28979M	Post Dosing Status		
		normal, before dosing	DSNG	1-91
		normal, postdosing (1.observation)	DSNG	1-91
		normal, postdosing (2.observation)	DSNG	1-91
		normal, postdosing (3.observation)	DSNG	1-91
3/M	28985M	Post Dosing Status		
		normal, before dosing	DSNG	1-91
		normal, postdosing (1.observation)	DSNG	1-91
		normal, postdosing (2.observation)	DSNG	1-91
		normal, postdosing (3.observation)	DSNG	1-91

Table
Individual Clinical Observations
Test Item (dosage) 1 2 3 4

[redacted]

Group/ Sex	Animal Number	Observation	Phase	Day(s)
4/M	28666M	Post Dosing Status		
		normal, before dosing	DSNG	1-91
		normal, postdosing (1.observation)	DSNG	1-6,8,9,11-91
		normal, postdosing (2.observation)	DSNG	1-91
		normal, postdosing (3.observation)	DSNG	1-91
		Mouth/ vomiting		
		emesis of liquid, white, postdosing (1.observation) /after dosing	DSNG	7
		emesis of liquid, white, postdosing (1.observation)	DSNG	10

Table
Individual Clinical Observations
Test Item (dosage) 1 2 3 4

[redacted]

Group/ Sex	Animal Number	Observation	Phase	Day(s)
4/M	28670M	Post Dosing Status		
		normal, before dosing	DSNG	1-91
		normal, postdosing (1.observation)	DSNG	1-32,34-37,39-41,43-61, 63-66,68-88,91
		normal, postdosing (2.observation)	DSNG	1-91
		normal, postdosing (3.observation)	DSNG	1-91
		Mouth/ vomiting		
		emesis of liquid, white, postdosing (1.observation)	DSNG	33,38,62,67,89,90
		emesis of liquid, foamy, postdosing (1.observation)	DSNG	42

Table
Individual Clinical Observations
Test Item (dosage) 1 2 3 4

[redacted]

Group/ Sex	Animal Number	Observation	Phase	Day(s)
4/M	28678M	Post Dosing Status		
		normal, before dosing	DSNG	1-91
		normal, postdosing (1.observation)	DSNG	1-91
		normal, postdosing (2.observation)	DSNG	1-81,86-91
		normal, postdosing (3.observation)	DSNG	1-80,87-91
		Behavior/appearance		
		movement abnormalities, both legs, slight, postdosing (3.observation)	DSNG	81-85
		ataxia, postdosing (2.observation)	DSNG	82-85
		tremor, extremity/ies, slight, postdosing (2.observation)	DSNG	82-85
		prostrate, postdosing (3.observation)	DSNG	86
4/M	28687M	Post Dosing Status		
		normal, before dosing	DSNG	1-91
		normal, postdosing (1.observation)	DSNG	1-91
		normal, postdosing (2.observation)	DSNG	1-91
		normal, postdosing (3.observation)	DSNG	1-91

Table
Individual Clinical Observations
Test Item (dosage) 1 2 3 4

[redacted]

Group/ Sex	Animal Number	Observation	Phase	Day(s)
4/M	28697M	Post Dosing Status		
		normal, before dosing	DSNG	1-92
		normal, postdosing (1.observation)	DSNG	1-92
		normal, postdosing (2.observation)	DSNG	1-92
		normal, postdosing (3.observation)	DSNG	1-92
4/M	28702M	Post Dosing Status		
		normal, before dosing	DSNG	1-92
		normal, postdosing (1.observation)	DSNG	1-51,53-92
		normal, postdosing (2.observation)	DSNG	1-92
		normal, postdosing (3.observation)	DSNG	1-92
		Mouth/ vomiting		
		emesis of liquid, white, postdosing (1.observation)	DSNG	52
		emesis of liquid during dosing, white, postdosing (1.observation)	DSNG	52

Table
Individual Clinical Observations
Test Item (dosage) 1 2 3 4

[redacted]

Group/ Sex	Animal Number	Observation	Phase	Day(s)
1/F	28738F	Post Dosing Status		
		normal, before dosing	DSNG	1-91
		normal, postdosing (1.observation)	DSNG	1-91
		normal, postdosing (2.observation)	DSNG	1-91
		normal, postdosing (3.observation)	DSNG	1-91
1/F	28741F	Post Dosing Status		
		normal, before dosing	DSNG	1-91
		normal, postdosing (1.observation)	DSNG	1-91
		normal, postdosing (2.observation)	DSNG	1-91
		normal, postdosing (3.observation)	DSNG	1-91
1/F	28756F	Post Dosing Status		
		normal, before dosing	DSNG	1-92
		normal, postdosing (1.observation)	DSNG	1-92
		normal, postdosing (2.observation)	DSNG	1-92
		normal, postdosing (3.observation)	DSNG	1-92

Table
Individual Clinical Observations
Test Item (dosage) 1 2 3 4

[redacted]

Group/ Sex	Animal Number	Observation	Phase	Day(s)
1/F	28757F	Post Dosing Status		
		normal, before dosing	DSNG	1-91
		normal, postdosing (1.observation)	DSNG	1-91
		normal, postdosing (2.observation)	DSNG	1-91
		normal, postdosing (3.observation)	DSNG	1-91
1/F	28761F	Post Dosing Status		
		normal, before dosing	DSNG	1-92
		normal, postdosing (1.observation)	DSNG	1-92
		normal, postdosing (2.observation)	DSNG	1-92
		normal, postdosing (3.observation)	DSNG	1-92
1/F	28763F	Post Dosing Status		
		normal, before dosing	DSNG	1-91
		normal, postdosing (1.observation)	DSNG	1-91
		normal, postdosing (2.observation)	DSNG	1-91
		normal, postdosing (3.observation)	DSNG	1-91

Table
Individual Clinical Observations
Test Item (dosage) 1 2 3 4

[redacted]

Group/ Sex	Animal Number	Observation	Phase	Day(s)
2/F	27825F	Post Dosing Status		
		normal, before dosing	DSNG	1-91
		normal, postdosing (1.observation)	DSNG	1-91
		normal, postdosing (2.observation)	DSNG	1-91
		normal, postdosing (3.observation)	DSNG	1-91
2/F	28203F	Post Dosing Status		
		normal, before dosing	DSNG	1-91
		normal, postdosing (1.observation)	DSNG	1-91
		normal, postdosing (2.observation)	DSNG	1-91
		normal, postdosing (3.observation)	DSNG	1-91
2/F	28713F	Post Dosing Status		
		normal, before dosing	DSNG	1-92
		normal, postdosing (1.observation)	DSNG	1-92
		normal, postdosing (2.observation)	DSNG	1-92
		normal, postdosing (3.observation)	DSNG	1-92

Table
Individual Clinical Observations
Test Item (dosage) 1 2 3 4

[redacted]

Group/ Sex	Animal Number	Observation	Phase	Day(s)
2/F	28762F	Post Dosing Status		
		normal, before dosing	DSNG	1-92
		normal, postdosing (1.observation)	DSNG	1-92
		normal, postdosing (2.observation)	DSNG	1-92
		normal, postdosing (3.observation)	DSNG	1-92
3/F	27329F	Post Dosing Status		
		normal, before dosing	DSNG	1-91
		normal, postdosing (1.observation)	DSNG	1-91
		normal, postdosing (2.observation)	DSNG	1-91
		normal, postdosing (3.observation)	DSNG	1-91
3/F	28173F	Post Dosing Status		
		normal, before dosing	DSNG	1-91
		normal, postdosing (1.observation)	DSNG	1-91
		normal, postdosing (2.observation)	DSNG	1-91
		normal, postdosing (3.observation)	DSNG	1-91

Table
Individual Clinical Observations
Test Item (dosage) 1 2 3 4

[redacted]

Group/ Sex	Animal Number	Observation	Phase	Day(s)
3/F	28755F	Post Dosing Status		
		normal, before dosing	DSNG	1-92
		normal, postdosing (1.observation)	DSNG	1-92
		normal, postdosing (2.observation)	DSNG	1-92
		normal, postdosing (3.observation)	DSNG	1-92
3/F	28764F	Post Dosing Status		
		normal, before dosing	DSNG	1-92
		normal, postdosing (1.observation)	DSNG	1-92
		normal, postdosing (2.observation)	DSNG	1-92
		normal, postdosing (3.observation)	DSNG	1-92
4/F	28529F	Post Dosing Status		
		normal, before dosing	DSNG	1-91
		normal, postdosing (1.observation)	DSNG	1-91
		normal, postdosing (2.observation)	DSNG	1-91
		normal, postdosing (3.observation)	DSNG	1-91

Table
Individual Clinical Observations
Test Item (dosage) 1 2 3 4

[redacted]

Group/ Sex	Animal Number	Observation	Phase	Day(s)
4/F	28531F	Post Dosing Status		
		normal, before dosing	DSNG	1-91
		normal, postdosing (1.observation)	DSNG	1-28,30-32,34,36-38,40-43, 46,47,51,53,56,57,59-61,63, 68,69,71-78,82-89,91
		normal, postdosing (2.observation)	DSNG	1-91
		normal, postdosing (3.observation)	DSNG	1-91
		Mouth/ vomiting		
		emesis of liquid, white, postdosing (1.observation)	DSNG	29,33,35,39,44,45,48-50,52, 54,55,58,62,64-67,79-81,90
		emesis of mash, postdosing (1.observation)	DSNG	58,90
		emesis of foam, white, postdosing (1.observation)	DSNG	70
4/F	28535F	Post Dosing Status		
		normal, before dosing	DSNG	1-92
		normal, postdosing (1.observation)	DSNG	1-92
		normal, postdosing (2.observation)	DSNG	1-92
		normal, postdosing (3.observation)	DSNG	1-92

Table
Individual Clinical Observations
Test Item (dosage) 1 2 3 4

[redacted]

Group/ Sex	Animal Number	Observation	Phase	Day(s)
4/F	28539F	Post Dosing Status		
		normal, before dosing	DSNG	1-92
		normal, postdosing (1.observation)	DSNG	1-7,9-92
		normal, postdosing (2.observation)	DSNG	1-92
		normal, postdosing (3.observation)	DSNG	1-92
		Mouth/ vomiting		
		emesis of liquid, white, postdosing (1.observation)	DSNG	8
4/F	28740F	Post Dosing Status		
		normal, before dosing	DSNG	1-91
		normal, postdosing (1.observation)	DSNG	1-15,17-91
		normal, postdosing (2.observation)	DSNG	1-91
		normal, postdosing (3.observation)	DSNG	1-91
		Mouth/ vomiting		
		emesis of liquid, white, postdosing (1.observation)	DSNG	16

Table
Individual Clinical Observations
Test Item (dosage) 1 2 3 4

[redacted]

Group/ Sex	Animal Number	Observation	Phase	Day(s)
4/F	28754F	Post Dosing Status		
		normal, before dosing	DSNG	1-91
		normal, postdosing (1.observation)	DSNG	1-91
		normal, postdosing (2.observation)	DSNG	1-91
		normal, postdosing (3.observation)	DSNG	1-91

Data in Support of Table 2: Study F, Blood pressure

[redacted]

Group/ Sex	Phase / Day / Session / Name	Predose 1 1 HDO	Predose 5 1 HDO	Dosing 5 2 0 Hour	Dosing 5 4 8 Hour	Dosing 22 2 0 Hour	Dosing 22 3 4 Hour
		SYS mmHg					
1/M	Mean	157	132	170	129	132	121
	SD	23.3	10.2	30.1	8.3	13.0	4.5
	N	6	6	6	6	6	6
2/M	Mean	153	128	148	133	132	118
	SD	39.8	20.4	42.8	21.8	13.6	12.8
	N	4	4	4	4	4	4
3/M	Mean	129	134	129	126	125	116
	SD	9.7	1.7	17.9	7.1	7.9	3.2
	N	4	4	4	4	4	4
4/M	Mean	140	138	131	135	127	117
	SD	22.1	13.2	11.2	5.8	11.0	6.2
	N	6	6	6	6	6	6
	Statistics	AT	A	A	AT	A	A

A = ANOVA and Dunnett's
T = Rank-transformed data

Table
Summary of Blood Pressure

[redacted]

Group/Sex	Phase / Day / Session / Name	SYS mmHg Dosing 85 2 0 Hour	SYS mmHg Dosing 85 3 4 Hour	SYS mmHg Recovery 78 1 HDO
1/M	Mean	129	128	172
	SD	10.2	8.7	27.6
	N	6	6	2
2/M	Mean	129	127	-
	SD	16.7	32.6	-
	N	4	4	-
3/M	Mean	129	132	-
	SD	6.6	12.3	-
	N	4	4	-
4/M	Mean	136	130	128
	SD	11.9	10.3	17.7
	N	6	6	2
	Statistics	A	A	X7

A = ANOVA and Dunnett's
X7 = Not analyzed (mean of actual group sizes too small)

Table
Summary of Blood Pressure

[redacted]

Group/Sex	Phase / Day / Session / Name	Predose 1 1 HDO	Predose 5 1 HDO	Dosing 5 2 0 Hour	Dosing 5 4 8 Hour	Dosing 22 2 0 Hour	Dosing 22 3 4 Hour
		DIA mmHg					
1/M	Mean	88	67	108	61	71	60
	SD	20.6	12.1	27.2	14.9	20.4	9.4
	N	6	6	6	6	6	6
2/M	Mean	90	67	83	71	71	54
	SD	37.4	16.9	38.3	14.8	15.2	13.3
	N	4	4	4	4	4	4
3/M	Mean	68	69	61**	54	60	58
	SD	7.8	2.9	11.2	4.1	9.7	2.7
	N	4	4	4	4	4	4
4/M	Mean	72	71	71*	70	64	58
	SD	18.3	9.1	9.7	12.2	18.2	9.7
	N	6	6	6	6	6	6
	Statistics	AT	A	AT	A	A	A

* P<=0.05
** P<=0.01
*** P<=0.001
A = ANOVA and Dunnett's
T = Rank-transformed data

Table
Summary of Blood Pressure

[redacted]

		DIA mmHg		
	Phase	Dosing		Recovery
Group/ Sex	Day	85		78
	Session	2	3	1
	Name	0 Hour	4 Hour	HDO
1/M	Mean	66	65	90
	SD	16.5	9.7	15.6
	N	6	6	2
2/M	Mean	63	64	-
	SD	15.4	30.6	-
	N	4	4	-
3/M	Mean	63	61	-
	SD	7.8	2.6	-
	N	4	4	-
4/M	Mean	74	63	78
	SD	12.0	10.5	21.9
	N	6	6	2
	Statistics	A	AT	X7

A = ANOVA and Dunnett's
T = Rank-transformed data
X7 = Not analyzed (mean of actual group sizes too small)

Table
Summary of Blood Pressure

[redacted]

Group/Sex	Phase / Day / Session / Name	Predose 1 / 1 / HDO	Predose 5 / 1 / HDO	Dosing 5 / 2 / 0 Hour	Dosing 5 / 4 / 8 Hour	Dosing 22 / 2 / 0 Hour	Dosing 22 / 3 / 4 Hour
		MAP mmHg					
1/M	Mean	112	90	130	85	93	82
	SD	21.3	11.0	28.0	11.7	17.0	7.2
	N	6	6	6	6	6	6
2/M	Mean	112	89	106	93	93	77
	SD	38.3	17.4	39.7	16.9	14.1	12.8
	N	4	4	4	4	4	4
3/M	Mean	90	92	85*	80	83	79
	SD	8.3	1.5	12.9	2.6	9.1	2.4
	N	4	4	4	4	4	4
4/M	Mean	96	95	92*	93	87	79
	SD	19.3	8.1	9.6	9.4	15.2	7.3
	N	6	6	6	6	6	6
	Statistics	AT	A	AT	A	A	A

* P<=0.05
** P<=0.01
*** P<=0.001
A = ANOVA and Dunnett's
T = Rank-transformed data

Table
Summary of Blood Pressure

[redacted]

		MAP mmHg		
	Phase	Dosing		Recovery
Group/ Sex	Day	85		78
	Session	2	3	1
	Name	0 Hour	4 Hour	HDO
1/M	Mean	88	88	119
	SD	13.8	7.5	19.1
	N	6	6	2
2/M	Mean	87	87	-
	SD	16.1	31.4	-
	N	4	4	-
3/M	Mean	86	86	-
	SD	7.3	5.5	-
	N	4	4	-
4/M	Mean	96	87	96
	SD	11.2	9.5	20.5
	N	6	6	2
	Statistics	A	AT	X7

A = ANOVA and Dunnett's
T = Rank-transformed data
X7 = Not analyzed (mean of actual group sizes too small)

Table
Summary of Blood Pressure

[redacted]

Group/ Sex	Phase / Day / Session / Name	Predose 1 1 HDO	Predose 5 1 HDO	SYS mmHg Dosing 5 2 0 Hour	Dosing 5 4 8 Hour	Dosing 22 2 0 Hour	Dosing 22 3 4 Hour
1/F	Mean	136	156	142	144	117	131
	SD	16.4	11.9	10.5	11.6	6.6	16.6
	N	6	6	6	6	6	6
2/F	Mean	137	159	150	141	123	152
	SD	20.1	27.3	32.4	24.9	21.8	37.8
	N	4	4	4	4	4	4
3/F	Mean	122	145	131	129	122	130
	SD	11.2	19.5	4.6	21.6	18.4	17.8
	N	4	4	4	4	4	4
4/F	Mean	142	159	160	157	126	152
	SD	20.9	24.6	24.9	25.3	8.6	31.7
	N	6	6	6	6	6	6
	Statistics	A	A	AT	A	AT	A

A = ANOVA and Dunnett's
T = Rank-transformed data

Table
Summary of Blood Pressure

[redacted]

Group/ Sex	Phase / Day / Session / Name	SYS mmHg Dosing 85 / 2 / 0 Hour	SYS mmHg Dosing 85 / 3 / 4 Hour	Recovery 78 / 1 / HDO
1/F	Mean	169	135	154
	SD	25.6	13.7	1.4
	N	6	5	2
2/F	Mean	153	150	-
	SD	43.7	32.0	-
	N	4	4	-
3/F	Mean	139	128	-
	SD	16.5	11.4	-
	N	4	4	-
4/F	Mean	146	142	132
	SD	24.2	21.6	21.9
	N	6	6	2
	Statistics	A	A	X7

A = ANOVA and Dunnett's
X7 = Not analyzed (mean of actual group sizes too small)

Table
Summary of Blood Pressure

[redacted]

Group/Sex	Phase / Day / Session / Name	Predose Day 1 Session 1 HDO	Predose Day 5 Session 1 HDO	Dosing (DIA mmHg) Day 5 Session 2 0 Hour	Dosing Day 5 Session 4 8 Hour	Dosing Day 22 Session 2 0 Hour	Dosing Day 22 Session 3 4 Hour
1/F	Mean	83	90	81	82	62	75
	SD	20.6	9.7	7.9	6.2	6.3	14.9
	N	6	6	6	6	6	6
2/F	Mean	83	101	100	86	76	94
	SD	22.2	16.8	26.7	15.1	14.6	26.9
	N	4	4	4	4	4	4
3/F	Mean	72	93	88	68	64	75
	SD	6.2	12.0	9.5	8.9	11.7	11.6
	N	4	4	4	4	4	4
4/F	Mean	84	99	92	90	70	89
	SD	17.2	10.2	17.8	18.2	9.0	33.4
	N	6	6	6	6	6	6
	Statistics	A	A	A	A	A	A

A = ANOVA and Dunnett's

Table
Summary of Blood Pressure

[redacted]

Group/Sex	Phase / Day / Session / Name	DIA mmHg Dosing Day 85 Session 2 0 Hour	DIA mmHg Dosing Day 85 Session 3 4 Hour	DIA mmHg Recovery Day 78 Session 1 HDO
1/F	Mean	95	79	95
	SD	15.2	8.2	0.7
	N	6	5	2
2/F	Mean	91	91	-
	SD	33.4	21.2	-
	N	4	4	-
3/F	Mean	79	76	-
	SD	6.7	8.5	-
	N	4	4	-
4/F	Mean	67	82	79
	SD	33.5	19.2	0.7
	N	6	6	2
	Statistics	A	A	X7

A = ANOVA and Dunnett's
X7 = Not analyzed (mean of actual group sizes too small)

Table
Summary of Blood Pressure

[redacted]

Group/Sex	Phase / Day / Session / Name	Predose: Day 1, Session 1, HDO	Predose: Day 5, Session 1, HDO	Dosing: Day 5, Session 2, 0 Hour	Dosing: Day 5, Session 4, 8 Hour	Dosing: Day 22, Session 2, 0 Hour	Dosing: Day 22, Session 3, 4 Hour
		MAP mmHg					
1/F	Mean	102	113	102	104	81	95
	SD	18.1	9.4	6.3	7.0	5.8	15.2
	N	6	6	6	6	6	6
2/F	Mean	103	122	118	105	93	114
	SD	20.2	20.2	28.6	18.1	16.9	30.0
	N	4	4	4	4	4	4
3/F	Mean	90	112	104	90	84	95
	SD	7.8	14.1	6.2	12.8	13.8	12.0
	N	4	4	4	4	4	4
4/F	Mean	105	120	116	113	90	111
	SD	17.5	13.7	19.9	19.6	8.3	31.9
	N	6	6	6	6	6	6
	Statistics	A	A	AT	A	A	A

A = ANOVA and Dunnett's
T = Rank-transformed data

Table
Summary of Blood Pressure

[redacted]

Group/ Sex	Phase / Day / Session / Name	MAP mmHg Dosing 85 / 2 / 0 Hour	MAP mmHg Dosing 85 / 3 / 4 Hour	Recovery 78 / 1 / HDO
1/F	Mean	121	99	116
	SD	17.0	9.8	0.7
	N	6	5	2
2/F	Mean	113	112	-
	SD	36.5	24.7	-
	N	4	4	-
3/F	Mean	100	95	-
	SD	9.6	9.7	-
	N	4	4	-
4/F	Mean	95	103	98
	SD	25.9	19.1	7.8
	N	6	6	2
	Statistics	A	A	X7

A = ANOVA and Dunnett's
X7 = Not analyzed (mean of actual group sizes too small)

Data in Support of Table 2: Study G, Platelets

Table
Summary of Hematology
Test Item (dosage) 1 2
[redacted] [redacted] [redacted] [redacted] [redacted] [redacted]

PLT. 10E9/L

Group/ Sex	Phase	Predose		Dosing		Recovery	
	Day	22	29/31	40	83	43	58
	Session	1		1	1	1	1
	Name	Hema/IPT/Ki	1	Hema/IPT/Ki	Hema/IPT/Ki	Hema/IPT/Ki	Hema/IPT/Ki
1/M	Mean	279	295	255	250	296	323
	SD	82.5	92.2	67.7	75.6	58.0	27.6
	N	5	5	5	5	2	2
2/M	Mean	310	356	316	319	-	-
	SD	62.7	57.7	20.3	57.4	-	-
	N	3	3	3	3	-	-
	%-Diff	11%	21%	24%	28%	-	-
3/M	Mean	244	345	327	328	-	-
	SD	98.0	124.7	96.5	96.5	-	-
	N	3	3	3	3	-	-
	%-Diff	-13%	17%	28%	31%	-	-
4/M	Mean	310	308	338	337	366	345
	SD	69.7	92.8	49.1	33.9	31.8	2.8
	N	5	5	5	5	2	2
	%-Diff	11%	4%	33%	35%	24%	7%
	Statistics	A	A	A	A	X7	X7

A = ANOVA and Dunnett's
X7 = Not analyzed (mean of actual group sizes too small)

Table
Summary of Hematology
Test Item (dosage) 1 2
[redacted] [redacted] [redacted] [redacted] [redacted] [redacted]

PLT. 10E9/L

Group/ Sex	Phase / Day / Session / Name	Predose 22 / 1 / Hema/IPT/Ki	Predose 29/31 / / 1	Dosing 40 / 1 / Hema/IPT/Ki	Dosing 83 / 1 / Hema/IPT/Ki	Recovery 43 / 1 / Hema/IPT/Ki	Recovery 58 / 1 / Hema/IPT/Ki
1/F	Mean	276	290	286	353	355	319
	SD	99.4	95.1	57.8	46.5	100.4	101.1
	N	5	5	5	5	2	2
2/F	Mean	349	396	374	375	-	-
	SD	47.6	54.7	74.5	71.0	-	-
	N	3	3	3	3	-	-
	%-Diff	26%	37%	31%	6%	-	-
3/F	Mean	437	455	480**	458	-	-
	SD	38.6	87.8	48.1	117.0	-	-
	N	3	3	3	3	-	-
	%-Diff	58%	57%	68%	30%	-	-
4/F	Mean	328	364	340	260	262	322
	SD	54.1	94.7	57.4	158.7	32.5	94.0
	N	5	5	5	5	2	2
	%-Diff	19%	26%	19%	-26%	-26%	1%
	Statistics	A	A	A	AT	X7	X7

* P<=0.05
** P<=0.01
*** P<=0.001
A = ANOVA and Dunnett's
T = Rank-transformed data

X7 = Not analyzed (mean of actual group sizes too small)

Data in Support of Table 2: Study G, Mic (lymph node)

Incidence of Microscopic Observations
Terminal Sacrifice

Test Item	(dosage)	1	2	3	4
[redacted]	[redacted]	[redacted]	[redacted]	[redacted]	[redacted]

Tissue/ Observation	Group/Sex: Number of Animals:	1/M 3	2/M 3	3/M 3	4/M 3	1/F 3	2/F 3	3/F 3	4/F 3
Liver	Number Examined:	3	3	3	3	3	3	3	3
	Unremarkable:	0	1	1	0	0	0	0	0
[redacted]		3	2	2	3	3	3	3	3
Lung	Number Examined:	3	3	3	3	3	3	3	3
	Unremarkable:	2	2	1	1	3	3	1	1
[redacted]		1	1	1	0	0	0	2	1
		0	0	0	1	0	0	0	0
[redacted]		0	0	2	2	0	0	0	2
Lymph Node, Axillary	Number Examined:	3	3	3	3	3	3	3	3
	Unremarkable:	3	0	0	0	3	0	0	0
Foamy macrophages		0	3	3	3	0	2	2	3
Increased cellularity, lymphocytes		0	0	0	0	0	1	1	0
Lymph Node, Mandibular	Number Examined:	3	3	3	3	3	3	3	3
	Unremarkable:	3	3	2	1	3	3	2	0
Foamy macrophages		0	0	1	2	0	0	0	3
Infiltrate, neutrophils		0	0	0	0	0	0	1	0

Incidence of Microscopic Observations
Terminal Sacrifice

Test Item	(dosage)	1	2	3	4
[redacted]	[redacted]	[redacted]	[redacted]	[redacted]	[redacted]

Tissue/ Observation	Group/Sex: Number of Animals:	1/M 3	2/M 3	3/M 3	4/M 3	1/F 3	2/F 3	3/F 3	4/F 3
Lymph Node, Mesenteric	Number Examined:	3	3	3	3	3	3	3	3
	Unremarkable:	2	3	1	0	3	1	1	0
Foamy macrophages		0	0	2	3	0	2	1	3
Hematopoiesis, extramedullary		0	0	0	0	0	0	1	0
Increased cellularity, lymphocytes		1	0	0	0	0	0	1	0
Mammary Gland	Number Examined:	3	3	3	3	3	3	3	3
	Unremarkable:	3	3	3	3	3	3	3	3
Mandibular Salivary Gland	Number Examined:	3	3	3	3	3	3	3	3
	Unremarkable:	2	2	3	0	1	1	3	0
[redacted]		1	1	0	3	2	2	0	3
Muscle, Skeletal	Number Examined:	3	3	3	3	3	3	3	3
	Unremarkable:	3	3	3	3	3	3	3	3
Nerve, Optic	Number Examined:	3	3	3	3	3	3	3	3
	Unremarkable:	3	3	3	3	3	3	3	3
Nerve, Sciatic	Number Examined:	3	3	3	3	3	3	3	3
	Unremarkable:	3	3	3	3	3	3	3	2
[redacted]		0	0	0	0	0	0	0	1

Data in Support of Table 2: Study G, Mac (lymph node)

Incidence of Macroscopic Observations
Terminal Sacrifice

[redacted]est Item (dosage) 1 2 3 4

[redacted] [redacted] [redacted] [redacted] [redacted] [redacted]

Tissue/ Observation	Group/Sex: Number of Animals:	1/M 3	2/M 3	3/M 3	4/M 3	1/F 3	2/F 3	3/F 3	4/F 3
	Unremarkable:	1	1	2	0	2	2	1	1
Lymph Node, Axillary	Number Examined:	3	3	3	3	3	3	3	3
	Unremarkable:	3	1	2	2	3	2	1	2
Enlargement		0	2	1	1	0	1	2	1
Lymph Node, Mandibular	Number Examined:	3	3	3	3	3	3	3	3
	Unremarkable:	3	3	3	3	3	3	3	3
Lymph Node, Mesenteric	Number Examined:	3	3	3	3	3	3	3	3
	Unremarkable:	2	3	3	2	3	3	2	1
Enlargement		1	0	0	1	0	0	1	2
Mammary Gland	Number Examined:	3	3	3	3	3	3	3	3
	Unremarkable:	3	3	3	3	3	3	3	3
Mandibular Salivary Gland	Number Examined:	3	3	3	3	3	3	3	3
	Unremarkable:	3	3	3	3	3	3	3	3

Data in Support of Table 2: Study H, Mic (injection site)

[redacted]

Incidence of Microscopic Observations - Terminal Sacrifice
Test Item (dosage) 1 2 3 4 5

[redacted] [redacted]

Tissue/ Observation	Group/Sex: Number of Animals:	1/M 4	2/M 4	3/M 4	4/M 4	5/M 4	1/F 4	2/F 4	3/F 4	4/F 4	5/F 4
Subcutaneous Injection Site	Number Examined:	4	4	4	4	4	4	4	4	4	4
	Unremarkable:	3	3	2	3	0	3	2	3	0	0
Fibrosis		1	1	1	0	2	1	0	0	0	1
Folliculitis		0	0	0	0	0	0	0	1	0	0
Hemorrhage		0	0	0	0	0	0	1	0	2	0
Infiltration of inflammatory cells		0	0	2	1	4	0	2	1	4	4
Inflammation		0	0	0	0	0	0	1	0	2	0
Ulceration, epidermis		0	0	0	0	0	0	0	0	1	0
Testis	Number Examined:	4	4	4	4	4	0	0	0	0	0
	Unremarkable:	0	0	0	0	0	0	0	0	0	0
[redacted]		4	4	4	4	4	0	0	0	0	0
Thymus	Number Examined:	4	4	4	4	4	4	4	4	4	4
	Unremarkable:	4	4	4	4	4	4	4	4	4	4
Thyroid	Number Examined:	4	4	4	4	4	4	4	4	4	4
	Unremarkable:	3	3	3	3	2	3	3	2	2	3
[redacted]		0	1	0	0	0	0	1	2	0	0
		1	1	1	1	2	1	0	0	2	1

Data in Support of Table 2: Study H, Serum AP

Table
Summary of Clinical Chemistry
Test Item (dosage) 1 2 3 4 5
[redacted] [redacted] [redacted]

Group/Sex	Phase / Day	Predose 2	Predose 11	Dosing 22	Dosing 83	Dosing 174	Recovery 23
		ALP. U/L					
1/M	Mean	744.04	728.62	713.94	683.96	673.29	690.73
	SD	106.582	76.739	121.872	115.645	108.179	175.787
	N	6	6	6	5	6	2
2/M	Mean	1047.63	935.85	863.84	886.99	790.23	-
	SD	295.680	277.803	220.071	156.276	114.677	-
	N	4	4	4	4	4	-
3/M	Mean	1191.42	976.78	905.22	1041.47	936.23	-
	SD	361.434	376.586	289.400	372.723	306.030	-
	N	4	4	4	2	4	-
4/M	Mean	727.13	680.89	685.51	688.14	694.92	-
	SD	165.243	140.311	176.824	105.445	145.319	-
	N	4	4	4	4	4	-
5/M	Mean	1005.96	864.43	1020.66	1271.94***	1077.90	822.00
	SD	349.616	293.549	369.607	341.240	429.437	173.432
	N	6	6	6	6	6	2
	Statistics	A	A	A	AT	AT	X7

* P<=0.05
** P<=0.01
*** P<=0.001
A = ANOVA and Dunnett's
T = Rank-transformed data

X7 = Not analyzed (mean of actual group sizes too small)

Table
Summary of Clinical Chemistry
Test Item (dosage) 1 2 3 4 5
[redacted] [redacted] [redacted]

Group/Sex	Phase / Day	Predose 2	Predose 17	Dosing 22	Dosing 83	Dosing 174	Recovery 23
		ALP. U/L					
1/F	Mean	986.28	898.47	822.81	810.40	832.62	1091.26
	SD	232.451	199.953	94.735	181.436	178.672	350.477
	N	6	6	4	6	6	2
2/F	Mean	756.21	757.97	774.62	809.99	757.21	-
	SD	91.104	64.799	118.032	34.770	90.360	-
	N	4	4	4	4	4	-
3/F	Mean	922.80	867.74	825.58	797.55	807.82	-
	SD	285.352	289.648	215.060	133.017	106.948	-
	N	4	4	4	4	4	-
4/F	Mean	875.21	834.69	834.76	891.06	868.33	-
	SD	242.519	291.273	239.049	282.920	358.768	-
	N	4	4	4	4	4	-
5/F	Mean	877.34	927.27	1135.49	1315.93**	1113.31	819.82
	SD	198.508	232.417	213.837	291.309	388.727	33.736
	N	6	6	6	6	6	2
	Statistics	A	A	A	A	A	X7

* P<=0.05
** P<=0.01
*** P<=0.001
A = ANOVA and Dunnett's
X7 = Not analyzed (mean of actual group sizes too small)

Data in Support of Table 2: Study H, TDAR

Summary of Anti-KLH IgG Males

Test Item (dosage) 1 2 3 4 5

[redacted]

Group/Sex	Phase Day	KLHG Dosing 110	117	124	131	138
1/M	Mean	142	2157	7809	8373	9335
	SD	90.7	235.5	3284.9	3193.6	1906.9
	N	6	6	6	6	6
2/M	Mean	231	2316	3949	5835	4562
	SD	113.6	1635.6	1513.2	2773.1	1757.4
	N	4	4	4	4	4
3/M	Mean	237	4142	10462	9584	5497
	SD	180.0	5148.3	13015.5	10566.7	4486.7
	N	4	4	4	4	4
4/M	Mean	183	1470	4240	6062	5972
	SD	82.6	701.1	2573.5	2977.8	3724.9
	N	4	4	4	4	4
5/M	Mean	100	789**	2860	3399	2949**
	SD	0.0	526.6	3822.6	3819.2	3344.4
	N	6	6	6	6	6
	Statistics	AT	AT	AT	A	A

* P<=0.05
** P<=0.01
*** P<=0.001
A = ANOVA and Dunnett's
T = Rank-transformed data

Summary of Anti-KLH IgG Males

Test Item (dosage) 1 2 3 4 5

[redacted] [redacted] [redacted] [redacted] [redacted] [redacted] [redacted] [redacted] [redacted]

Group/ Sex	Phase / Day	KLHG Dosing 145	152	159	166	173
1/M	Mean	5621	30276	67698	41702	23314
	SD	2593.9	17166.3	42043.8	17857.0	14023.9
	N	6	6	6	6	6
2/M	Mean	3153	52137	42953	18948	12718
	SD	998.7	5063.1	12914.8	11008.1	4927.5
	N	4	4	4	4	4
3/M	Mean	4623	54546	47444	34664	15712
	SD	4938.1	3743.0	8280.6	16337.8	6976.0
	N	4	4	4	4	4
4/M	Mean	5581	67714	95852	33364	26236
	SD	3803.5	89904.9	115905.8	28161.6	24124.0
	N	4	4	4	4	4
5/M	Mean	2660	23698	29154	16289	17303
	SD	3146.6	18880.7	22960.9	12416.1	16725.0
	N	6	6	6	6	6
	Statistics	A	AT	AT	AT	AT

A = ANOVA and Dunnett's

T = Rank-transformed data

Summary of Anti-KLH IgG Females

Test Item (dosage) 1 2 3 4 5

[redacted]

Group/ Sex	Phase / Day	KLHG Dosing 110	117	124	131	138
1/F	Mean	209	3386	9688	14872	9650
	SD	173.8	2468.7	1864.2	11724.4	2187.3
	N	6	6	6	6	6
2/F	Mean	114	2264	17525	12729	8658
	SD	10.0	83.6	16550.0	7128.7	4589.7
	N	4	4	4	4	4
3/F	Mean	186	1446	2876*	5677	5531
	SD	172.5	924.7	2078.7	4441.9	4119.1
	N	4	4	4	4	4
4/F	Mean	100	1561	6033	8030	11240
	SD	0.0	826.7	4534.7	3311.0	8511.7
	N	4	4	4	4	4
5/F	Mean	112	2480	5061	6313	5320
	SD	29.0	2350.2	4472.5	3791.2	3601.9
	N	6	6	6	6	6
	Statistics	AT	A	AT	A	A

* P<=0.05
** P<=0.01
*** P<=0.001
A = ANOVA and Dunnett's
T = Rank-transformed data

Summary of Anti-KLH IgG Females

Test Item (dosage) 1 2 3 4 5

[redacted]

Group/ Sex	Phase / Day	KLHG Dosing 145	152	159	166	173
1/F	Mean	8362	130615	117703	79504	34002
	SD	2421.0	103437.6	86413.4	44982.5	16232.0
	N	6	6	6	6	6
2/F	Mean	7143	>232450	105335	117872	41205
	SD	4879.3	78167.6	77330.6	97606.7	19239.6
	N	4	4	4	4	4
3/F	Mean	4458	77572	88355	26787	19702
	SD	3592.1	118418.1	131426.6	22626.1	20828.7
	N	4	4	4	4	4
4/F	Mean	9338	>151894	>146824	79143	53696
	SD	7207.4	125013.6	121353.1	61142.3	7424.7
	N	4	4	4	4	4
5/F	Mean	2949	60690	47286	41980	37526
	SD	2939.8	33061.2	13137.6	13841.7	12849.1
	N	6	6	6	6	6
	Statistics	A	X5	X5	AT	A

A = ANOVA and Dunnett's
X5 = Not analyzed (values above/below the limit of quantitation)
T = Rank-transformed data

Data in Support of Table 2: Study H, Clinical obs (skin)

Test Item	(dosage)	1 2 3 4 5
[redacted]	[redacted]	[redacted]

Group/ Sex	Animal Number	Observation	Phase	Day(s)
1/M	P0001	NORMAL		
		No remarkable observations	DSNG	1,8,15,22,29,36,43,50,57,64, 71,78,85,92,99,106,113,120, 127,134,141,148,155,162,169, 176,182
1/M	P0002	NORMAL		
		No remarkable observations	DSNG	1,8,22,29,36,43,50,57,64,71, 78,85,92,99,106,113,120,127, 134,141,148,155,162,169,182
		Extremity/ies		
		lesion/s, right upper leg, crusted	DSNG	176
		Head		
		lesion/s, forehead, crusted	DSNG	15

Table
Individual Clinical Observations

Test Item	(dosage)	1 2 3 4 5
[redacted]	[redacted]	[redacted]

Group/ Sex	Animal Number	Observation	Phase	Day(s)
1/M	P0003	NORMAL		
		No remarkable observations	DSNG	1,8,15,22,113,120,127,134, 141,148,155,162
		Genital region/s		
		lesion/s, left scrotum, crusted	DSNG	169,176
		lesion/s, right scrotum, crusted	DSNG	36
		lesion/s, scrotum, wet	DSNG	182
		Trunk		
		lesion/s, tip of tail, crusted	DSNG	29,36,43,50,57,64,71,78,85, 92,99,106
1/M	P0004	NORMAL		
		No remarkable observations	DSNG	1,8,15,22,29,36,43,57,64,71, 92,99,106,113,120,127,134, 141,148,155
		Trunk		
		discolored skin, right inguen, red, small area	DSNG	162,169,176,182
		lesion/s, tail, bloody	DSNG	50
		lesion/s, tip of tail, bloody	DSNG	78
		lesion/s, tip of tail, crusted	DSNG	85

Table
Individual Clinical Observations
Test Item (dosage) 1 2 3 4 5

[redacted] [redacted] [redacted]

Group/ Sex	Animal Number	Observation	Phase	Day(s)
1/M	P0005	NORMAL		
		No remarkable observations	DSNG	1,71,78,85,120,127,134,155, 162,169,176,182
			RECO	7,14,21,28
		Head		
		lesion/s, chin, crusted	DSNG	15
		lesion/s, forehead, crusted	DSNG	8
		lesion/s, nose, crusted	DSNG	141,148
		lesion/s, right nasal wing, crusted	DSNG	15
		Trunk		
		lesion/s, tip of tail, bloody	DSNG	148
		lesion/s, tip of tail, crusted	DSNG	15,22,29,36,43,50,57,64,92, 99,106
		lesion/s, upper part of back, crusted	DSNG	113

Table
Individual Clinical Observations

Test Item	(dosage)	1 2 3 4 5
[redacted]	[redacted]	[redacted]

Group/ Sex	Animal Number	Observation	Phase	Day(s)
1/M	P0006	NORMAL		
		No remarkable observations	DSNG	1,8,29,36,43,50,57,64,78,85, 92,99,106,113,120,127,155, 182
			RECO	7,14,21,28
		Extremity/ies		
		lesion/s, right upper leg, crusted	DSNG	162,169
		Genital region/s		
		lesion/s, right scrotum, crusted	DSNG	71
		lesion/s, scrotum, crusted	DSNG	134,141,148
		spots, right scrotum, red	DSNG	176
		Trunk		
		discolored skin, left inguen, red, small area	DSNG	15,22

Table
Individual Clinical Observations
Test Item (dosage) 1 2 3 4 5

[redacted] [redacted] [redacted]

Group/ Sex	Animal Number	Observation	Phase	Day(s)
2/M	P0101	NORMAL		
		No remarkable observations	DSNG	8,15,22,29,43,64,71,78,85, 92,99,106,113,120,127,134
		Extremity/ies		
		lesion/s, left upper leg, crusted	DSNG	162,169
		Eye/s		
		discolored skin, right eyebrow, blue, small area	DSNG	155
		swelling/s, slight, right eyebrow, soft	DSNG	155
		Genital region/s		
		lesion/s, left scrotum, crusted	DSNG	1
		spots, left scrotum, single	DSNG	36
		spots, scrotum, red	DSNG	50,57
		Trunk		
		lesion/s, tail, bloody	DSNG	141,148
		lesion/s, tail, crusted	DSNG	155,162,169,176,182

Table
Individual Clinical Observations

Test Item	(dosage)	1 2 3 4 5
[redacted]	[redacted]	[redacted]

Group/ Sex	Animal Number	Observation	Phase	Day(s)
2/M	P0102	NORMAL		
		No remarkable observations	DSNG	1,36,43,50,64
		Eye/s		
		discolored skin, both eyebrows, red, large area	DSNG	182
		Head		
		lesion/s, face, crusted	DSNG	8
		lesion/s, forehead, bloody	DSNG	176
		lesion/s, forehead, crusted	DSNG	15,22

Table
Individual Clinical Observations
Test Item (dosage) 1 2 3 4 5

[redacted] [redacted] [redacted]

Group/ Sex	Animal Number	Observation	Phase	Day(s)
2/M	P0102	Trunk		
		discolored skin, both inguinal, red, small area	DSNG	120,127,134,141,148,155,162, 169,176,182
		discolored skin, left inguen, red, large area	DSNG	113
		discolored skin, left inguen, red, small area	DSNG	57,71,78,85,92,99,106
		discolored skin, right axilla, red, small area	DSNG	92
		discolored skin, right inguen, red, small area	DSNG	113
		lesion/s, tip of tail, crusted	DSNG	22,29
		lesion/s, upper part of back, crusted	DSNG	29
		squamous skin, slight, both inguinal, small area	DSNG	148,155,162,169,176,182

Table
Individual Clinical Observations

Test Item	(dosage)	1 2 3 4 5
[redacted]	[redacted]	[redacted]

Group/ Sex	Animal Number	Observation	Phase	Day(s)
2/M	P0103	NORMAL		
		No remarkable observations	DSNG	15,22,29,43,50,57,64,71,78, 85,92,99,106,113,120,127, 134,141,148,155,162,169
		Genital region/s		
		spots, left scrotum, single	DSNG	36
		Trunk		
		lesion/s, lower part of back, crusted	DSNG	1,8
		lesion/s, upper part of back, crusted	DSNG	176,182
2/M	P0104	NORMAL		
		No remarkable observations	DSNG	1,8,29,36,43,50,57,64,71,78, 85,106,113,120,127,134,141, 148,155,162,169,176,182
		Eye/s		
		discolored skin, right upper eyelid, red, small area	DSNG	22
		Head		
		lesion/s, forehead, crusted	DSNG	15
		Trunk		
		lesion/s, tip of tail, crusted	DSNG	92,99

Table
Individual Clinical Observations
Test Item (dosage) 1 2 3 4 5

[redacted] [redacted] [redacted]

Group/ Sex	Animal Number	Observation	Phase	Day(s)
3/M	P0201	NORMAL		
		No remarkable observations	DSNG	1,15,29,43,50,64,71,78,85, 92,99,106,113,120,127,134
		Eye/s		
		discolored skin, left upper eyelid, blue, large area	DSNG	57
		Fur: discolored		
		injection site/s, field 1, yellow	DSNG	57
		Head		
		lesion/s, left side of head, crusted	DSNG	8
		Trunk		
		discolored skin, both inguinal, red, small area	DSNG	141,148,155,162,169,176,182
		lesion/s, lower part of back, crusted	DSNG	22,36
		lesion/s, upper part of back, crusted	DSNG	22
		squamous skin, slight, right inguen, small area	DSNG	148,155,162,169,176,182

Table
Individual Clinical Observations

Test Item	(dosage)	1 2 3 4 5
[redacted]	[redacted]	[redacted]

Group/ Sex	Animal Number	Observation	Phase	Day(s)
3/M	P0202	NORMAL		
		No remarkable observations	DSNG	1,8,15,22,29,36,57,64,71,78, 85,92,99,106,113,120,127, 134,141,148,155,162,169,176, 182
		Trunk		
		lesion/s, upper part of back, crusted	DSNG	43,50

Table
Individual Clinical Observations

Test Item	(dosage)	1 2 3 4 5
[redacted]	[redacted]	[redacted]

Group/ Sex	Animal Number	Observation	Phase	Day(s)
3/M	P0203	NORMAL		
		No remarkable observations	DSNG	8,15,64,71,78,85,92,99,106, 113,120,127,134,141,148,155, 162,169,176,182
		Fur: discolored		
		injection site/s, field 1, yellow	DSNG	57
		Head		
		lesion/s, chin, crusted	DSNG	22
		lesion/s, left cheek, crusted	DSNG	1
		swelling/s, slight, left cheek, indurated	DSNG	1
		Trunk		
		discolored skin, both inguinal, red, small area	DSNG	29,36,43,50
		lesion/s, upper part of back, crusted	DSNG	50

Table
Individual Clinical Observations
Test Item (dosage) 1 2 3 4 5

[redacted] [redacted] [redacted]

Group/ Sex	Animal Number	Observation	Phase	Day(s)
3/M	P0204	NORMAL		
		No remarkable observations	DSNG	1,43,50,71,78,85,92,99,106, 113,134,141,148,155,162,169, 176,182
		Fur: discolored		
		injection site/s, field 1, yellow	DSNG	57
		injection site/s, injection site/s, yellow	DSNG	64
		Head		
		lesion/s, head, crusted	DSNG	15
		Trunk		
		swelling/s, slight, both inguinal, soft	DSNG	8,15,22,29,36,120,127
4/M	P0301	NORMAL		
		No remarkable observations	DSNG	1,8,15,22,29,43,50,57,64,71, 78,85,92,99,106,113,120,127, 134,141,148,155,162,169,176, 182
		Head		
		lesion/s, forehead, crusted	DSNG	36

Table
Individual Clinical Observations

Test Item	(dosage)	1 2 3 4 5
[redacted]	[redacted]	[redacted]

Group/ Sex	Animal Number	Observation	Phase	Day(s)
4/M	P0302	NORMAL		
		No remarkable observations	DSNG	8,15,22,113,120,127,134,141, 148,155,162,176,182
		Fur: discolored		
		injection site/s, field 1, yellow	DSNG	57
		injection site/s, injection site/s, yellow	DSNG	64
		Head		
		discolored skin, forehead, red, small area	DSNG	1
		lesion/s, forehead, crusted	DSNG	169
		Trunk		
		lesion/s, lower part of back, crusted	DSNG	85,92,99
		lesion/s, tip of tail, crusted	DSNG	29,36,43,50,71,78,85,92,99, 106
		lesion/s, whole back, crusted	DSNG	78

Table
Individual Clinical Observations
Test Item (dosage) 1 2 3 4 5
[redacted] [redacted] [redacted]

Group/ Sex	Animal Number	Observation	Phase	Day(s)
4/M	P0303	NORMAL		
		No remarkable observations	DSNG	1,8,15,22,29,36,43,50,71,78, 85,92,99,106,113,120,127, 134,148,155,162,169,176,182
		Fur: discolored		
		injection site/s, field 1, yellow	DSNG	57
		injection site/s, injection site/s, yellow	DSNG	64
		Head		
		lesion/s, chin, crusted	DSNG	141

Table
Individual Clinical Observations
Test Item (dosage)   1 2 3 4 5
---------- ------------------ - - -- -- ---
[redacted]

Group/ Sex	Animal Number	Observation	Phase	Day(s)
4/M	P0304	NORMAL		
		No remarkable observations	DSNG	127
		Extremity/ies		
		discolored skin, both arms, red, large area	DSNG	85,92
		discolored skin, both arms, red, small area	DSNG	99
		discolored skin, both upper arms, red, large area	DSNG	71,78
		discolored skin, both upper legs, red, small area	DSNG	141,148,155,162,169,176,182
		squamous skin, slight, both upper arms, large area	DSNG	71,78
		squamous skin, slight, left arm, large area	DSNG	85,92
		Fur: discolored		
		injection site/s, field 1, yellow	DSNG	57
		injection site/s, injection site/s, yellow	DSNG	64

Table
Individual Clinical Observations
Test Item (dosage) 1 2 3 4 5

[redacted] [redacted] [redacted]

Group/ Sex	Animal Number	Observation	Phase	Day(s)
4/M	P0304	Trunk		
		discolored skin, both axillae, red, large area	DSNG	71,78,85,92,162,169,176,182
		discolored skin, both axillae, red, small area	DSNG	99
		discolored skin, both inguinal, red, large area	DSNG	1,8,15,29,36,43,50,57,64,71, 78,85,92,134,141,148,155, 162,169,176,182
		discolored skin, both inguinal, red, small area	DSNG	99,120
		discolored skin, lower abdomen, red, small area	DSNG	64,71,78
		discolored skin, neck, red, large area	DSNG	71,78,85,92,176,182
		discolored skin, whole abdomen, red, large area	DSNG	85,92
		discolored skin, whole abdomen, red, small area	DSNG	99
		lesion/s, both inguinal, crusted	DSNG	8,15
		lesion/s, lower part of back, crusted	DSNG	36

Table
Individual Clinical Observations
Test Item (dosage) 1 2 3 4 5

[redacted] [redacted] [redacted]

Group/ Sex	Animal Number	Observation	Phase	Day(s)
4/M	P0304	Trunk		
		lesion/s, tip of tail, crusted	DSNG	15,22,29,71,78,85,92,99,106, 113,120
		lesion/s, upper part of back, crusted	DSNG	169,176
		scabby skin, lower abdomen, large area	DSNG	162,169,176,182
		squamous skin, severe, both inguinal, large area	DSNG	22
		squamous skin, slight, both axillae, large area	DSNG	71,78,85,92
		squamous skin, slight, both inguinal, large area	DSNG	29,36,43,50,57,64,71,78,85, 92,148,155,162,169,176,182
		squamous skin, slight, both inguinal, small area	DSNG	8,15
		swelling/s, slight, right inguen, soft	DSNG	8,15

Table
Individual Clinical Observations
Test Item (dosage) 1 2 3 4 5

[redacted] [redacted] [redacted]

Group/ Sex	Animal Number	Observation	Phase	Day(s)
5/M	P0401	NORMAL		
		No remarkable observations	DSNG	8,64,71,78,85,99,113,120, 127,134,141,148,155,162,169, 176,182
		Eye/s		
		lesion/s, right upper eyelid, crusted	DSNG	1
		Head		
		lesion/s, forehead, bloody	DSNG	106
		lesion/s, nose, bloody	DSNG	92
		Trunk		
		lesion/s, tip of tail, crusted	DSNG	15,22,29,36,43,50,57
5/M	P0402	NORMAL		
		No remarkable observations	DSNG	1,8,15,22,29,36,43,50,57,64, 78,85,92,99,106,113,120,127, 134,141,148,155,162,169,176, 182
		Trunk		
		discolored skin, chest, red, small area	DSNG	71

Table
Individual Clinical Observations
Test Item (dosage) 1 2 3 4 5

[redacted] [redacted] [redacted]

Group/ Sex	Animal Number	Observation	Phase	Day(s)
5/M	P0403	NORMAL		
		No remarkable observations	DSNG	1,8,15,22,29,36,50,57,64,71, 78,85,92,99,106,120,127,155, 162,169,176,182
		Extremity/ies		
		spots, left upper leg, crusted	DSNG	141
		squamous skin, slight, right foot, large area	DSNG	43
		Trunk		
		lesion/s, tail distal, wet	DSNG	113,134
		lesion/s, tail, crusted	DSNG	141,148

Table
Individual Clinical Observations

Test Item	(dosage)	1 2 3 4 5
[redacted]	[redacted]	[redacted]

Group/ Sex	Animal Number	Observation	Phase	Day(s)
5/M	P0404	NORMAL		
		No remarkable observations	DSNG	1,8,36,43,50,57,64,71,78,85
		Head		
		discolored skin, chin, blue, large area	DSNG	141
		lesion/s, forehead, crusted	DSNG	15,22
		Trunk		
		discolored skin, both inguinal, red, large area	DSNG	176,182
		discolored skin, both inguinal, red, small area	DSNG	148,155
		lesion/s, tip of tail, crusted	DSNG	15,22,29,92,99,106,113,120, 127,134,141,148,155,162,169, 176,182
		swelling/s, slight, right inguen, soft	DSNG	120,127

Table
Individual Clinical Observations

Test Item	(dosage)	1 2 3 4 5
[redacted]	[redacted]	[redacted]

Group/ Sex	Animal Number	Observation	Phase	Day(s)
5/M	P0405	NORMAL		
		No remarkable observations	DSNG	1,8,15,22,29,36,43,50,106, 113,120,127,134,176,182
			RECO	7,14,21,28
		Head		
		discolored skin, head, red, small area	DSNG	148,155
		Trunk		
		lesion/s, tip of tail, crusted	DSNG	57,64,71,78,85,92,99,141, 148,155,162,169

Table
Individual Clinical Observations

Test Item	(dosage)	1 2 3 4 5
[redacted]	[redacted]	[redacted]

Group/ Sex	Animal Number	Observation	Phase	Day(s)
5/M	P0406	NORMAL		
		No remarkable observations	DSNG	1,15,22,29,50,57,64,71,78, 85,92,99,113,120,127,134
			RECO	28
		Head		
		lesion/s, both nasal wings, bloody	DSNG	169
		lesion/s, face, crusted	DSNG	43
		lesion/s, forehead, crusted	DSNG	36
		lesion/s, head, bloody	DSNG	155
		lesion/s, head, crusted	RECO	14
		lesion/s, left side of head, crusted	DSNG	8,169,176,182
		lesion/s, nose, crusted	DSNG	155
		lesion/s, right side of head, crusted	DSNG	106,148
		swelling/s, slight, nose, soft	DSNG	169
		Trunk		
		lesion/s, tip of tail, crusted	DSNG	141,148,155,162,169,182
			RECO	7,14,21
		lesion/s, upper part of back, crusted	DSNG	141

Table
Individual Clinical Observations
Test Item (dosage) 1 2 3 4 5

[redacted] [redacted] [redacted]

Group/ Sex	Animal Number	Observation	Phase	Day(s)
1/F	P0501	NORMAL		
		No remarkable observations	PRED	17
			DSNG	1,8,15,22,29,36,50,57,127, 148,162
		Eye/s		
		lesion/s, right upper eyelid, crusted	DSNG	155
		Head		
		lesion/s, back part of head, crusted	DSNG	78,85,92
		Trunk		
		lesion/s, left inguen, crusted	DSNG	155
		lesion/s, lower part of back, crusted	DSNG	141
		lesion/s, right shoulder, crusted	DSNG	71
		lesion/s, tip of tail, crusted	DSNG	64,71,78,85,92,99,106,113, 120
		lesion/s, upper part of back, crusted	DSNG	43,99,106,134
		lesion/s, whole back, crusted	DSNG	169,176,182

Table
Individual Clinical Observations
Test Item (dosage) 1 2 3 4 5

[redacted] [redacted] [redacted]

Group/ Sex	Animal Number	Observation	Phase	Day(s)
1/F	P0502	NORMAL		
		No remarkable observations	PRED	17
			DSNG	1,8,106,113,120,148,155,162, 169,182
		Eye/s		
		discolored skin, right upper eyelid, blue, small area	DSNG	176
		Head		
		lesion/s, forehead, crusted	DSNG	36
		lesion/s, nose, crusted	DSNG	78,176
		Trunk		
		lesion/s, lower part of back, crusted	DSNG	78,85
		lesion/s, tip of tail, bloody	DSNG	36,64,71
		lesion/s, tip of tail, crusted	DSNG	15,29,43,50,57,78,85,92,99, 127,134,141
		lesion/s, tip of tail, wet	DSNG	22

Table
Individual Clinical Observations

Test Item	(dosage)	1 2 3 4 5
[redacted]	[redacted]	[redacted]

Group/ Sex	Animal Number	Observation	Phase	Day(s)
1/F	P0503	NORMAL		
		No remarkable observations	PRED	17
			DSNG	1,8,15,43,50,120,127,134, 141,148
		Extremity/ies		
		lesion/s, left lower leg, bloody	DSNG	99
		Trunk		
		discolored skin, both inguinal, red, small area	DSNG	162,169,176,182
		discolored skin, lower abdomen, red, small area	DSNG	155
		lesion/s, tail, bloody	DSNG	57
		lesion/s, tip of tail, crusted	DSNG	22,29,36,64,71,78,85,92,99, 106,113

Table
Individual Clinical Observations
Test Item (dosage)   1 2 3 4 5
---------- ------------------ - - -- -- ---
[redacted]

Group/ Sex	Animal Number	Observation	Phase	Day(s)
1/F	P0504	NORMAL		
		No remarkable observations	PRED	17
			DSNG	1,8,15,22,29,36,43,50,64,71, 78,85,92,99,106,113,120,127, 134,155,162,169,176,182
		Head		
		discolored skin, face, red, small area	DSNG	141,148
		lesion/s, forehead, bloody	DSNG	57
1/F	P0505	NORMAL		
		No remarkable observations	PRED	17
			DSNG	1,8,15,22,29,36,43,50,57,64, 71,78,85,92,99,106,113,120, 127,134,141,148,155,162,176, 182
			RECO	7,14,21
		Head		
		lesion/s, chin, crusted	DSNG	169
		Trunk		
		lesion/s, tip of tail, crusted	RECO	28

Table
Individual Clinical Observations

Test Item	(dosage)	1 2 3 4 5
[redacted]	[redacted]	[redacted]

Group/ Sex	Animal Number	Observation	Phase	Day(s)
1/F	P0506	NORMAL		
		No remarkable observations	PRED	17
			DSNG	1,8,15,22,29,36,43,50,57, 148,155,162,169,176,182
			RECO	7,14,21,28
		Head		
		lesion/s, nose, crusted	DSNG	141
		Trunk		
		lesion/s, tip of tail, crusted	DSNG	64,71,78,85,92,99,106,113, 120,127,134,141

Table
Individual Clinical Observations

Test Item	(dosage)	1 2 3 4 5
[redacted]	[redacted]	[redacted]

Group/ Sex	Animal Number	Observation	Phase	Day(s)
2/F	P0601	NORMAL		
		No remarkable observations	PRED	17
			DSNG	29,36,50,99,106,113,120,127, 141,148,155,162,169,176,182
		Head		
		lesion/s, face, crusted	DSNG	78
		lesion/s, forehead, crusted	DSNG	8,15,22,134
		lesion/s, head, crusted	DSNG	15,22,57,78,85
		lesion/s, left side of head, bloody	DSNG	8
		lesion/s, nose, bloody	DSNG	1
		Trunk		
		lesion/s, tip of tail, crusted	DSNG	64,71,78,85,92
		lesion/s, whole back, crusted	DSNG	43
2/F	P0602	NORMAL		
		No remarkable observations	PRED	17
			DSNG	1,8,15,22,29,36,43,50,57,64, 99,106,113,120,127,134,141, 148,155,162,169,182
		Trunk		
		lesion/s, tip of tail, crusted	DSNG	71,78,85,92,176

Table
Individual Clinical Observations
Test Item (dosage) 1 2 3 4 5

[redacted] [redacted] [redacted]

Group/ Sex	Animal Number	Observation	Phase	Day(s)
2/F	P0603	NORMAL		
		No remarkable observations	PRED	17
			DSNG	1,15,22,29,43,50,57,155,162
		Head		
		lesion/s, forehead, crusted	DSNG	106
		lesion/s, head, bloody	DSNG	64
		lesion/s, left side of head, crusted	DSNG	106
		Mouth/ vomiting		
		lesion/s, lower lip, crusted	DSNG	134
		Trunk		
		lesion/s, chest, crusted	DSNG	148
		lesion/s, tip of tail, crusted	DSNG	78,85,92,99,106,113,120,127, 134,141,169,176,182
		lesion/s, upper part of back, crusted	DSNG	71
		lesion/s, whole back, crusted	DSNG	8,36,78,85,92,99

Table
Individual Clinical Observations
Test Item (dosage) 1 2 3 4 5

[redacted] [redacted] [redacted]

Group/ Sex	Animal Number	Observation	Phase	Day(s)
2/F	P0604	NORMAL		
		No remarkable observations	DSNG	8,15,22,29,36,43,64,71,78, 85,92,99,106,113,120,127, 134,141,148,155,162,169,176, 182
		Extremity/ies		
		spots, left arm, red	PRED	17
		spots, right arm, red	DSNG	1
		Trunk		
		lesion/s, tip of tail, crusted	DSNG	50,57

Table
Individual Clinical Observations

Test Item	(dosage)	1 2 3 4 5
[redacted]	[redacted]	[redacted]

Group/ Sex	Animal Number	Observation	Phase	Day(s)
3/F	P0701	NORMAL		
		No remarkable observations	DSNG	29
		Head		
		discolored skin, face, red, large area	DSNG	169

Table
Individual Clinical Observations
Test Item (dosage) 1 2 3 4 5

[redacted] [redacted] [redacted]

Group/ Sex	Animal Number	Observation	Phase	Day(s)
3/F	P0701	Trunk		
		discolored skin, both inguinal, orange, large area	DSNG	141,148,155,162,169,176,182
		discolored skin, both inguinal, orange, small area	DSNG	134
		discolored skin, both inguinal, red, large area	PRED	17
			DSNG	1,8,78,85,92,106,113,120,127
		discolored skin, both inguinal, red, small area	DSNG	99
		discolored skin, left inguen, red, large area	DSNG	36,50,57,64,71
		discolored skin, left inguen, red, small area	DSNG	15,22,43
		discolored skin, lower abdomen, orange, large area	DSNG	162,169,176,182
		discolored skin, lower abdomen, red, small area	PRED	17
			DSNG	1,8
		discolored skin, right inguen, red, small area	DSNG	36,50,57,64,71

Table
Individual Clinical Observations
Test Item (dosage) 1 2 3 4 5

[redacted] [redacted] [redacted]

Group/ Sex	Animal Number	Observation	Phase	Day(s)
3/F	P0701	Trunk		
		discolored skin, upper abdomen, red, small area	DSNG	176,182
		lesion/s, lower part of back, crusted	DSNG	57
		squamous skin, slight, left inguen, large area	DSNG	36
		squamous skin, slight, right inguen, small area	DSNG	36
3/F	P0702	NORMAL		
		No remarkable observations	PRED	17
			DSNG	1,8,15,22,29,36,43,50,57,64, 71,78,85,92,99,106,113,120, 127,134,141,148,155,162,169, 182
		Trunk		
		lesion/s, tip of tail, crusted	DSNG	176

Table
Individual Clinical Observations

Test Item	(dosage)	1 2 3 4 5
[redacted]	[redacted]	[redacted]

Group/ Sex	Animal Number	Observation	Phase	Day(s)
3/F	P0703	NORMAL		
		No remarkable observations	DSNG	22,29,36,43,57,64,71,78,85, 92,99,106,113,120,127,134, 141,148,155,162,169,176,182
		Eye/s		
		discolored skin, left upper eyelid, red, small area	DSNG	1
		Fur: discolored		
		injection site/s, field 1, yellow	DSNG	50
		Head		
		discolored skin, chin, blue, small area	PRED	17
		lesion/s, back part of head, crusted	DSNG	8,15
		lesion/s, chin, crusted	PRED	17

Table
Individual Clinical Observations
Test Item (dosage) 1 2 3 4 5

[redacted] [redacted] [redacted]

Group/ Sex	Animal Number	Observation	Phase	Day(s)
3/F	P0704	NORMAL		
		No remarkable observations	PRED	17
			DSNG	1,8,15,22,29,36,50,57,99, 106,113,120,127,134,141,148, 155,162,169,176,182
		Trunk		
		lesion/s, tail distal, crusted	DSNG	43,64,71,78,85,92
		lesion/s, upper part of back, crusted	DSNG	78,85
4/F	P0801	NORMAL		
		No remarkable observations	PRED	17
			DSNG	1,8,15,22,29,36,43,64,71,78, 85,92,99,106,113,120,127, 134,141,148,155,162,169,176, 182
		Fur: discolored		
		injection site/s, field 1, yellow	DSNG	50
		injection site/s, injection site/s, yellow	DSNG	57

Table
Individual Clinical Observations
Test Item (dosage)   1 2 3 4 5
---------- ------------------ - - -- -- ---
[redacted]

Group/ Sex	Animal Number	Observation	Phase	Day(s)
4/F	P0802	NORMAL		
		No remarkable observations	DSNG	1,15,22,36,43,50,99,106,113, 120,127,134,141,148,155,162, 169,182
		Extremity/ies		
		lesion/s, left upper leg, crusted	DSNG	176
		Head		
		discolored skin, right side of head, blue, small area	DSNG	57
		lesion/s, back part of head, crusted	PRED	17
			DSNG	85,92,176
		lesion/s, forehead, crusted	PRED	17
		swelling/s, moderate, right side of head, soft	DSNG	57
		Trunk		
		lesion/s, lower part of back, crusted	DSNG	64,71
		lesion/s, tip of tail, crusted	DSNG	64,71,78,85
		lesion/s, whole back, crusted	DSNG	8,29,78

Table
Individual Clinical Observations
Test Item (dosage) 1 2 3 4 5

[redacted] [redacted] [redacted]

Group/ Sex	Animal Number	Observation	Phase	Day(s)
4/F	P0803	NORMAL		
		No remarkable observations	PRED	17
			DSNG	8,15,22,36,78,85,120,127, 134,141,148,155,169,176,182
		Extremity/ies		
		discolored skin, left leg, red, small area	DSNG	113
		Fur: discolored		
		injection site/s, injection site/s, yellow	DSNG	50

Table
Individual Clinical Observations
Test Item (dosage) 1 2 3 4 5

[redacted] [redacted] [redacted]

Group/ Sex	Animal Number	Observation	Phase	Day(s)
4/F	P0803	Trunk		
		discolored skin, chest, brown, small area	DSNG	92,99,106,113
		discolored skin, right inguen, blue, small area	DSNG	43
		lesion/s, lower part of back, crusted	DSNG	29,57,64,71
		lesion/s, right inguen, crusted	DSNG	50
		scabby skin, chest, large area	DSNG	162
		spots, upper part of back, red	DSNG	1
		squamous skin, slight, chest, small area	DSNG	92
		squamous skin, slight, neck, small area	DSNG	92

Table
Individual Clinical Observations
Test Item (dosage) 1 2 3 4 5
---------- ------------------ - - -- -- ---
[redacted] [redacted] [redacted]

Group/ Sex	Animal Number	Observation	Phase	Day(s)
4/F	P0804	NORMAL		
		No remarkable observations	PRED	17
			DSNG	1,15,22,29,36,43,57,64,71, 85,92,99,106,113,120,127, 134,141,148,155,162,169,182
		Extremity/ies		
		discolored skin, right upper leg, blue, small area	DSNG	78
		lesion/s, left upper leg, crusted	DSNG	176
		Fur: discolored		
		injection site/s, field 1, yellow	DSNG	50
		Trunk		
		lesion/s, lower part of back, crusted	DSNG	8

Table
Individual Clinical Observations
Test Item (dosage) 1 2 3 4 5

[redacted] [redacted] [redacted]

Group/ Sex	Animal Number	Observation	Phase	Day(s)
5/F	P0901	NORMAL		
		No remarkable observations	PRED	17
			DSNG	29,36,57,134,141,148
		Extremity/ies		
		lesion/s, right arm, crusted	DSNG	1
		lesion/s, right lower arm, crusted	DSNG	169,176
		Head		
		discolored skin, chin, blue, small area	DSNG	22
		lesion/s, head, crusted	DSNG	15,22,71
		Trunk		
		lesion/s, lower part of back, crusted	DSNG	15,22,155,162,169,176,182
		lesion/s, upper part of back, crusted	DSNG	50
		lesion/s, whole back, crusted	DSNG	1,8,43,64,71,78,85,92,99, 106,113,120,127

Table
Individual Clinical Observations
Test Item (dosage) 1 2 3 4 5

[redacted] [redacted] [redacted]

Group/ Sex	Animal Number	Observation	Phase	Day(s)
5/F	P0902	NORMAL		
		No remarkable observations	PRED	17
			DSNG	15,29,36,50,64,71,78,99,106, 113,120,127,134,141,148,155
		Extremity/ies		
		lesion/s, right finger/s, crusted	DSNG	1,8
		Head		
		lesion/s, face, crusted	DSNG	1,8,57,85,176
		lesion/s, forehead, crusted	DSNG	182
		lesion/s, right nasal wing, bloody	DSNG	22
		Trunk		
		lesion/s, tail proximal, crusted	DSNG	43
		lesion/s, tail, crusted	DSNG	57
		lesion/s, tip of tail, crusted	DSNG	85,92,162,169,176,182

Table
Individual Clinical Observations

Test Item	(dosage)	1 2 3 4 5
[redacted]	[redacted]	[redacted]

Group/ Sex	Animal Number	Observation	Phase	Day(s)
5/F	P0903	NORMAL		
		No remarkable observations	PRED	17
			DSNG	1,36,43,57,64,71,78,85,92, 99,106,113,120,127,134,141, 148,155,162,169,182
		Extremity/ies		
		discolored skin, right upper leg, blue, small area	DSNG	50
		lesion/s, left upper leg, crusted	DSNG	176
		Trunk		
		lesion/s, tip of tail, crusted	DSNG	8,15,22,29,176

Table
Individual Clinical Observations
Test Item (dosage) 1 2 3 4 5

[redacted] [redacted] [redacted]

Group/ Sex	Animal Number	Observation	Phase	Day(s)
5/F	P0904	NORMAL		
		No remarkable observations	PRED	17
			DSNG	1,8,15,29,36,64,71,78,85,92, 99,106,113,120,127,134,141, 148,155,162,169,176,182
		Eye/s		
		discolored skin, right upper eyelid, blue, small area	DSNG	43
		Fur: discolored		
		injection site/s, field 1, yellow	DSNG	50
		Head		
		lesion/s, forehead, bloody	DSNG	22,43
		lesion/s, head, crusted	DSNG	57
		lesion/s, left side of head, crusted	DSNG	50

Table
Individual Clinical Observations
Test Item (dosage)   1 2 3 4 5
---------- ------------------ - - -- -- ---
[redacted]

Group/ Sex	Animal Number	Observation	Phase	Day(s)
5/F	P0905	NORMAL		
		No remarkable observations	PRED	17
			DSNG	1,22,29,36,43,50,57,64,71, 78,85,92,99,106,113,120,127, 141,148,155,169,176,182
			RECO	7,14,21,28
		Head		
		lesion/s, nose, crusted	DSNG	134
		Trunk		
		lesion/s, upper part of back, crusted	DSNG	8,15
		lesion/s, whole back, crusted	DSNG	162
5/F	P0906	NORMAL		
		No remarkable observations	PRED	17
			DSNG	1,8,15,22,29,36,43,50,57,64, 71,78,85,92,99,106,113,120, 127,134,141,148,155,162,169, 176,182
			RECO	7,14,21,28

Data in Support of Table 2: Study H, Patellar reflex

Test Item	(dosage)	1 2 3 4 5
[redacted]	[redacted]	[redacted]

Group/ Sex	Animal Number	Observation	Phase	Day(s)
1/M	P0001	General observations		
		normal behavior	PRED	1
			DSNG	90,180
		Pupillary reflex		
		normal, both sides	PRED	1
			DSNG	90,180
		Orbicularis-oculi-reflex		
		normal, both sides	PRED	1
			DSNG	90,180
		Patellar reflex		
		normal, both sides	PRED	1
			DSNG	90
		not detectable, both sides	DSNG	180
		Anal reflex		
		normal	PRED	1
			DSNG	90,180
		Foot grip reflex		
		normal, both sides	PRED	1
			DSNG	90,180

Table
Individual Neurological Examinations

Test Item	(dosage)	1 2 3 4 5
[redacted]	[redacted]	[redacted]

Group/ Sex	Animal Number	Observation	Phase	Day(s)
1/M	P0002	General observations		
		normal behavior	PRED	1
			DSNG	90,180
		Pupillary reflex		
		normal, both sides	PRED	1
			DSNG	90,180
		Orbicularis-oculi-reflex		
		normal, both sides	PRED	1
			DSNG	90,180
		Patellar reflex		
		normal, both sides	PRED	1
			DSNG	90,180
		Anal reflex		
		normal	PRED	1
			DSNG	90,180
		Foot grip reflex		
		normal, both sides	PRED	1
			DSNG	90,180

Table
Individual Neurological Examinations
Test Item (dosage) 1 2 3 4 5

[redacted] [redacted] [redacted]

Group/ Sex	Animal Number	Observation	Phase	Day(s)
1/M	P0003	General observations		
		normal behavior	PRED	1
			DSNG	90,180
		Pupillary reflex		
		normal, both sides	PRED	1
			DSNG	90,180
		Orbicularis-oculi-reflex		
		normal, both sides	PRED	1
			DSNG	90,180
		Patellar reflex		
		normal, both sides	PRED	1
			DSNG	90,180
		Anal reflex		
		normal	PRED	1
			DSNG	90,180
		Foot grip reflex		
		normal, both sides	PRED	1
			DSNG	90,180

Table
Individual Neurological Examinations
Test Item (dosage) 1 2 3 4 5

[redacted] [redacted] [redacted]

Group/ Sex	Animal Number	Observation	Phase	Day(s)
1/M	P0004	General observations		
		normal behavior	PRED	1
			DSNG	90,180
		Pupillary reflex		
		normal, both sides	PRED	1
			DSNG	90,180
		Orbicularis-oculi-reflex		
		normal, both sides	PRED	1
			DSNG	90,180
		Patellar reflex		
		normal, both sides	PRED	1
			DSNG	90,180
		Anal reflex		
		normal	PRED	1
			DSNG	90,180
		Foot grip reflex		
		normal, both sides	PRED	1
			DSNG	90,180

Table
Individual Neurological Examinations

Test Item	(dosage)	1 2 3 4 5
[redacted]	[redacted]	[redacted]

Group/ Sex	Animal Number	Observation	Phase	Day(s)
1/M	P0005	General observations		
		normal behavior	PRED	1
			DSNG	90,180
			RECO	27
		Pupillary reflex		
		normal, both sides	PRED	1
			DSNG	90,180
			RECO	27
		Orbicularis-oculi-reflex		
		normal, both sides	PRED	1
			DSNG	90,180
			RECO	27
		Patellar reflex		
		normal, both sides	PRED	1
			DSNG	90,180
			RECO	27
		Anal reflex		
		normal	PRED	1
			DSNG	90,180
			RECO	27
		Foot grip reflex		
		normal, both sides	PRED	1
			DSNG	90,180
			RECO	27

Table
Individual Neurological Examinations

Test Item	(dosage)	1 2 3 4 5
[redacted]	[redacted]	[redacted]

Group/ Sex	Animal Number	Observation	Phase	Day(s)
1/M	P0006	General observations		
		normal behavior	PRED	1
			DSNG	90,180
			RECO	27
		Pupillary reflex		
		normal, both sides	PRED	1
			DSNG	90,180
			RECO	27
		Orbicularis-oculi-reflex		
		normal, both sides	PRED	1
			DSNG	90,180
			RECO	27
		Patellar reflex		
		normal, both sides	PRED	1
			DSNG	90
			RECO	27
		not detectable, both sides	DSNG	180
		Anal reflex		
		normal	PRED	1
			DSNG	90,180
			RECO	27

Table
Individual Neurological Examinations

Test Item	(dosage)	1 2 3 4 5
[redacted]	[redacted]	[redacted]

Group/ Sex	Animal Number	Observation	Phase	Day(s)
1/M	P0006	Foot grip reflex		
		normal, both sides	PRED	1
			DSNG	90,180
			RECO	27
2/M	P0101	General observations		
		normal behavior	PRED	1
			DSNG	90,180
		Pupillary reflex		
		normal, both sides	PRED	1
			DSNG	90,180
		Orbicularis-oculi-reflex		
		normal, both sides	PRED	1
			DSNG	90,180
		Patellar reflex		
		normal, both sides	PRED	1
			DSNG	90,180
		Anal reflex		
		normal	PRED	1
			DSNG	90,180
		Foot grip reflex		
		normal, both sides	PRED	1
			DSNG	90,180

Table
Individual Neurological Examinations
Test Item (dosage) 1 2 3 4 5
---------- ------------------ - - -- -- ---
[redacted] [redacted] [redacted]

Group/ Sex	Animal Number	Observation	Phase	Day(s)
2/M	P0102	General observations		
		normal behavior	PRED	1
			DSNG	90,180
		Pupillary reflex		
		normal, both sides	PRED	1
			DSNG	90,180
		Orbicularis-oculi-reflex		
		normal, both sides	PRED	1
			DSNG	90,180
		Patellar reflex		
		normal, both sides	PRED	1
			DSNG	90,180
		Anal reflex		
		normal	PRED	1
			DSNG	90,180
		Foot grip reflex		
		normal, both sides	PRED	1
			DSNG	90,180

Table
Individual Neurological Examinations

Test Item	(dosage)	1 2 3 4 5
[redacted]	[redacted]	[redacted]

Group/ Sex	Animal Number	Observation	Phase	Day(s)
2/M	P0103	General observations		
		normal behavior	PRED	1
			DSNG	90,180
		Pupillary reflex		
		normal, both sides	PRED	1
			DSNG	90,180
		Orbicularis-oculi-reflex		
		normal, both sides	PRED	1
			DSNG	90,180
		Patellar reflex		
		normal, both sides	PRED	1
			DSNG	90
		not detectable, both sides	DSNG	180
		Anal reflex		
		normal	PRED	1
			DSNG	90,180
		Foot grip reflex		
		normal, both sides	PRED	1
			DSNG	90,180

Table
Individual Neurological Examinations

Test Item	(dosage)	1 2 3 4 5
[redacted]	[redacted]	[redacted]

Group/ Sex	Animal Number	Observation	Phase	Day(s)
2/M	P0104	General observations		
		normal behavior	PRED	1
			DSNG	90,180
		Pupillary reflex		
		normal, both sides	PRED	1
			DSNG	90,180
		Orbicularis-oculi-reflex		
		normal, both sides	PRED	1
			DSNG	90,180
		Patellar reflex		
		normal, both sides	PRED	1
			DSNG	90,180
		Anal reflex		
		normal	PRED	1
			DSNG	90,180
		Foot grip reflex		
		normal, both sides	PRED	1
			DSNG	90,180

Table
Individual Neurological Examinations

Test Item	(dosage)	1 2 3 4 5
[redacted]	[redacted]	[redacted]

Group/ Sex	Animal Number	Observation	Phase	Day(s)
3/M	P0201	General observations		
		normal behavior	PRED	1
			DSNG	90,180
		Pupillary reflex		
		normal, both sides	PRED	1
			DSNG	90,180
		Orbicularis-oculi-reflex		
		normal, both sides	PRED	1
			DSNG	90,180
		Patellar reflex		
		normal, both sides	PRED	1
			DSNG	90,180
		Anal reflex		
		normal	PRED	1
			DSNG	90,180
		Foot grip reflex		
		normal, both sides	PRED	1
			DSNG	90,180

Table
Individual Neurological Examinations
Test Item (dosage) 1 2 3 4 5
---------- ------------------ - - -- -- ---
[redacted] [redacted] [redacted]

Group/ Sex	Animal Number	Observation	Phase	Day(s)
3/M	P0202	General observations		
		normal behavior	PRED	1
			DSNG	90,180
		Pupillary reflex		
		normal, both sides	PRED	1
			DSNG	90,180
		Orbicularis-oculi-reflex		
		normal, both sides	PRED	1
			DSNG	90,180
		Patellar reflex		
		normal, both sides	PRED	1
			DSNG	90
		not detectable, both sides	DSNG	180
		Anal reflex		
		normal	PRED	1
			DSNG	90,180
		Foot grip reflex		
		normal, both sides	PRED	1
			DSNG	90,180

Table
Individual Neurological Examinations
Test Item (dosage) 1 2 3 4 5
---------- ------------------ - - -- -- ---
[redacted] [redacted] [redacted]

Group/ Sex	Animal Number	Observation	Phase	Day(s)
3/M	P0203	General observations		
		normal behavior	PRED	1
			DSNG	90,180
		Pupillary reflex		
		normal, both sides	PRED	1
			DSNG	90,180
		Orbicularis-oculi-reflex		
		normal, both sides	PRED	1
			DSNG	90,180
		Patellar reflex		
		normal, both sides	PRED	1
			DSNG	90,180
		Anal reflex		
		normal	PRED	1
			DSNG	90,180
		Foot grip reflex		
		normal, both sides	PRED	1
			DSNG	90,180

Table
Individual Neurological Examinations

Test Item (dosage) 1 2 3 4 5

[redacted] [redacted] [redacted]

Group/ Sex	Animal Number	Observation	Phase	Day(s)
3/M	P0204	General observations		
		normal behavior	PRED	1
			DSNG	90,180
		Pupillary reflex		
		normal, both sides	PRED	1
			DSNG	90,180
		Orbicularis-oculi-reflex		
		normal, both sides	PRED	1
			DSNG	90,180
		Patellar reflex		
		normal, both sides	PRED	1
			DSNG	90,180
		Anal reflex		
		normal	PRED	1
			DSNG	90,180
		Foot grip reflex		
		normal, both sides	PRED	1
			DSNG	90,180

Table
Individual Neurological Examinations

Test Item	(dosage)	1 2 3 4 5
[redacted]	[redacted]	[redacted]

Group/ Sex	Animal Number	Observation	Phase	Day(s)
4/M	P0301	General observations		
		normal behavior	PRED	1
			DSNG	90,180
		Pupillary reflex		
		normal, both sides	PRED	1
			DSNG	90,180
		Orbicularis-oculi-reflex		
		normal, both sides	PRED	1
			DSNG	90,180
		Patellar reflex		
		normal, both sides	PRED	1
			DSNG	90
		not detectable, both sides	DSNG	180
		Anal reflex		
		normal	PRED	1
			DSNG	90,180
		Foot grip reflex		
		normal, both sides	PRED	1
			DSNG	90,180

Table
Individual Neurological Examinations
Test Item (dosage) 1 2 3 4 5
---------- ------------------ - - -- -- ---
[redacted] [redacted] [redacted]

Group/ Sex	Animal Number	Observation	Phase	Day(s)
4/M	P0302	General observations		
		normal behavior	PRED	1
			DSNG	90,180
		Pupillary reflex		
		normal, both sides	PRED	1
			DSNG	90,180
		Orbicularis-oculi-reflex		
		normal, both sides	PRED	1
			DSNG	90,180
		Patellar reflex		
		normal, both sides	PRED	1
			DSNG	90,180
		Anal reflex		
		normal	PRED	1
			DSNG	90,180
		Foot grip reflex		
		normal, both sides	PRED	1
			DSNG	90,180

Table
Individual Neurological Examinations

Test Item	(dosage)	1 2 3 4 5
[redacted]	[redacted]	[redacted]

Group/ Sex	Animal Number	Observation	Phase	Day(s)
4/M	P0303	General observations		
		normal behavior	PRED	1
			DSNG	90,180
		Pupillary reflex		
		normal, both sides	PRED	1
			DSNG	90,180
		Orbicularis-oculi-reflex		
		normal, both sides	PRED	1
			DSNG	90,180
		Patellar reflex		
		normal, both sides	PRED	1
			DSNG	90,180
		Anal reflex		
		normal	PRED	1
			DSNG	90,180
		Foot grip reflex		
		normal, both sides	PRED	1
			DSNG	90,180

Table
Individual Neurological Examinations
Test Item (dosage) 1 2 3 4 5

[redacted] [redacted] [redacted]

Group/ Sex	Animal Number	Observation	Phase	Day(s)
4/M	P0304	General observations		
		normal behavior	PRED	1
			DSNG	90,180
		Pupillary reflex		
		normal, both sides	PRED	1
			DSNG	90,180
		Orbicularis-oculi-reflex		
		normal, both sides	PRED	1
			DSNG	90,180
		Patellar reflex		
		normal, both sides	PRED	1
			DSNG	90
		not detectable, both sides	DSNG	180
		Anal reflex		
		normal	PRED	1
			DSNG	90,180
		Foot grip reflex		
		normal, both sides	PRED	1
			DSNG	90,180

Table
Individual Neurological Examinations

Test Item	(dosage)	1 2 3 4 5
[redacted]	[redacted]	[redacted]

Group/ Sex	Animal Number	Observation	Phase	Day(s)
5/M	P0401	General observations		
		normal behavior	PRED	1
			DSNG	90,180
		Pupillary reflex		
		normal, both sides	PRED	1
			DSNG	90,180
		Orbicularis-oculi-reflex		
		normal, both sides	PRED	1
			DSNG	90,180
		Patellar reflex		
		normal, both sides	PRED	1
			DSNG	90
		not detectable, both sides	DSNG	180
		Anal reflex		
		normal	PRED	1
			DSNG	90,180
		Foot grip reflex		
		normal, both sides	PRED	1
			DSNG	90,180

Table
Individual Neurological Examinations
Test Item (dosage) 1 2 3 4 5

[redacted] [redacted] [redacted]

Group/ Sex	Animal Number	Observation	Phase	Day(s)
5/M	P0402	General observations		
		normal behavior	PRED	1
			DSNG	90,180
		Pupillary reflex		
		normal, both sides	PRED	1
			DSNG	90,180
		Orbicularis-oculi-reflex		
		normal, both sides	PRED	1
			DSNG	90,180
		Patellar reflex		
		normal, both sides	PRED	1
			DSNG	90,180
		Anal reflex		
		normal	PRED	1
			DSNG	90,180
		Foot grip reflex		
		normal, both sides	PRED	1
			DSNG	90,180

Table
Individual Neurological Examinations

Test Item	(dosage)	1 2 3 4 5
[redacted]	[redacted]	[redacted]

Group/ Sex	Animal Number	Observation	Phase	Day(s)
5/M	P0403	General observations		
		normal behavior	PRED	1
			DSNG	90,180
		Pupillary reflex		
		normal, both sides	PRED	1
			DSNG	90,180
		Orbicularis-oculi-reflex		
		normal, both sides	PRED	1
			DSNG	90,180
		Patellar reflex		
		normal, both sides	PRED	1
			DSNG	90,180
		Anal reflex		
		normal	PRED	1
			DSNG	90,180
		Foot grip reflex		
		normal, both sides	PRED	1
			DSNG	90,180

Table
Individual Neurological Examinations

Test Item	(dosage)	1 2 3 4 5
[redacted]	[redacted]	[redacted]

Group/ Sex	Animal Number	Observation	Phase	Day(s)
5/M	P0404	General observations		
		normal behavior	PRED	1
			DSNG	90,180
		Pupillary reflex		
		normal, both sides	PRED	1
			DSNG	90,180
		Orbicularis-oculi-reflex		
		normal, both sides	PRED	1
			DSNG	90,180
		Patellar reflex		
		normal, both sides	PRED	1
			DSNG	90,180
		Anal reflex		
		normal	PRED	1
			DSNG	90,180
		Foot grip reflex		
		normal, both sides	PRED	1
			DSNG	90,180

Table
Individual Neurological Examinations
Test Item (dosage) 1 2 3 4 5

[redacted] [redacted] [redacted]

Group/ Sex	Animal Number	Observation	Phase	Day(s)
5/M	P0405	General observations		
		normal behavior	PRED	1
			DSNG	90,180
			RECO	27
		Pupillary reflex		
		normal, both sides	PRED	1
			DSNG	90,180
			RECO	27
		Orbicularis-oculi-reflex		
		normal, both sides	PRED	1
			DSNG	90,180
			RECO	27
		Patellar reflex		
		normal, both sides	PRED	1
			DSNG	90,180
			RECO	27
		Anal reflex		
		normal	PRED	1
			DSNG	90,180
			RECO	27
		Foot grip reflex		
		normal, both sides	PRED	1
			DSNG	90,180
			RECO	27

Table
Individual Neurological Examinations

Test Item	(dosage)	1 2 3 4 5
[redacted]	[redacted]	[redacted]

Group/ Sex	Animal Number	Observation	Phase	Day(s)
5/M	P0406	General observations		
		normal behavior	PRED	1
			DSNG	90,180
			RECO	27
		Pupillary reflex		
		normal, both sides	PRED	1
			DSNG	90,180
			RECO	27
		Orbicularis-oculi-reflex		
		normal, both sides	PRED	1
			DSNG	90,180
			RECO	27
		Patellar reflex		
		normal, both sides	PRED	1
			DSNG	90,180
			RECO	27
		Anal reflex		
		normal	PRED	1
			DSNG	90,180
			RECO	27
		Foot grip reflex		
		normal, both sides	PRED	1
			DSNG	90,180
			RECO	27

Table
Individual Neurological Examinations

Test Item	(dosage)	1 2 3 4 5
[redacted]	[redacted]	[redacted]

Group/ Sex	Animal Number	Observation	Phase	Day(s)
1/F	P0501	General observations		
		normal behavior	PRED	6
			DSNG	91,180
		Pupillary reflex		
		normal, both sides	PRED	6
			DSNG	91,180
		Orbicularis-oculi-reflex		
		normal, both sides	PRED	6
			DSNG	91,180
		Patellar reflex		
		normal, both sides	PRED	6
			DSNG	91,180
		Anal reflex		
		normal	PRED	6
			DSNG	91,180
		Foot grip reflex		
		normal, both sides	PRED	6
			DSNG	91,180

Table
Individual Neurological Examinations
Test Item (dosage) 1 2 3 4 5

[redacted] [redacted] [redacted]

Group/ Sex	Animal Number	Observation	Phase	Day(s)
1/F	P0502	General observations		
		normal behavior	PRED	6
			DSNG	91,180
		Pupillary reflex		
		normal, both sides	PRED	6
			DSNG	91,180
		Orbicularis-oculi-reflex		
		normal, both sides	PRED	6
			DSNG	91,180
		Patellar reflex		
		normal, both sides	PRED	6
			DSNG	91,180
		Anal reflex		
		normal	PRED	6
			DSNG	91,180
		Foot grip reflex		
		normal, both sides	PRED	6
			DSNG	91,180

Table
Individual Neurological Examinations

Test Item	(dosage)	1 2 3 4 5
[redacted]	[redacted]	[redacted]

Group/ Sex	Animal Number	Observation	Phase	Day(s)
1/F	P0503	General observations		
		normal behavior	PRED	6
			DSNG	91,180
		Pupillary reflex		
		normal, both sides	PRED	6
			DSNG	91,180
		Orbicularis-oculi-reflex		
		normal, both sides	PRED	6
			DSNG	91,180
		Patellar reflex		
		normal, both sides	PRED	6
			DSNG	91,180
		Anal reflex		
		normal	PRED	6
			DSNG	91,180
		Foot grip reflex		
		normal, both sides	PRED	6
			DSNG	91,180

Table
Individual Neurological Examinations
Test Item (dosage) 1 2 3 4 5
---------- ------------------ - - -- -- ---
[redacted] [redacted] [redacted]

Group/ Sex	Animal Number	Observation	Phase	Day(s)
1/F	P0504	General observations		
		normal behavior	PRED	6
			DSNG	91,180
		Pupillary reflex		
		normal, both sides	PRED	6
			DSNG	91,180
		Orbicularis-oculi-reflex		
		normal, both sides	PRED	6
			DSNG	91,180
		Patellar reflex		
		normal, both sides	PRED	6
			DSNG	91
		not detectable, both sides	DSNG	180
		Anal reflex		
		normal	PRED	6
			DSNG	91,180
		Foot grip reflex		
		normal, both sides	PRED	6
			DSNG	91,180

Table
Individual Neurological Examinations
Test Item (dosage) 1 2 3 4 5

[redacted] [redacted] [redacted]

Group/ Sex	Animal Number	Observation	Phase	Day(s)
1/F	P0505	General observations		
		normal behavior	PRED	6
			DSNG	91,180
			RECO	27
		Pupillary reflex		
		normal, both sides	PRED	6
			DSNG	91,180
			RECO	27
		Orbicularis-oculi-reflex		
		normal, both sides	PRED	6
			DSNG	91,180
			RECO	27
		Patellar reflex		
		normal, both sides	PRED	6
			DSNG	91,180
			RECO	27
		Anal reflex		
		normal	PRED	6
			DSNG	91,180
			RECO	27
		Foot grip reflex		
		normal, both sides	PRED	6
			DSNG	91,180
			RECO	27

Table
Individual Neurological Examinations
Test Item (dosage) 1 2 3 4 5
---------- ------------------ - - -- -- ---
[redacted] [redacted] [redacted]

Group/ Sex	Animal Number	Observation	Phase	Day(s)
1/F	P0506	General observations		
		normal behavior	PRED	6
			DSNG	91,180
			RECO	27
		Pupillary reflex		
		normal, both sides	PRED	6
			DSNG	91,180
			RECO	27
		Orbicularis-oculi-reflex		
		normal, both sides	PRED	6
			DSNG	91,180
			RECO	27
		Patellar reflex		
		normal, both sides	PRED	6
			DSNG	91
			RECO	27
		not detectable, both sides	DSNG	180
		Anal reflex		
		normal	PRED	6
			DSNG	91,180
			RECO	27

Table
Individual Neurological Examinations

Test Item	(dosage)	1 2 3 4 5
[redacted]	[redacted]	[redacted]

Group/ Sex	Animal Number	Observation	Phase	Day(s)
1/F	P0506	Foot grip reflex		
		normal, both sides	PRED	6
			DSNG	91,180
			RECO	27
2/F	P0601	General observations		
		normal behavior	PRED	6
			DSNG	91,180
		Pupillary reflex		
		normal, both sides	PRED	6
			DSNG	91,180
		Orbicularis-oculi-reflex		
		normal, both sides	PRED	6
			DSNG	91,180
		Patellar reflex		
		normal, both sides	PRED	6
			DSNG	91,180
		Anal reflex		
		normal	PRED	6
			DSNG	91,180
		Foot grip reflex		
		normal, both sides	PRED	6
			DSNG	91,180

Table
Individual Neurological Examinations

Test Item (dosage) 1 2 3 4 5

[redacted] [redacted] [redacted]

Group/ Sex	Animal Number	Observation	Phase	Day(s)
2/F	P0602	General observations		
		normal behavior	PRED	6
			DSNG	91,180
		Pupillary reflex		
		normal, both sides	PRED	6
			DSNG	91,180
		Orbicularis-oculi-reflex		
		normal, both sides	PRED	6
			DSNG	91,180
		Patellar reflex		
		normal, both sides	PRED	6
			DSNG	91,180
		Anal reflex		
		normal	PRED	6
			DSNG	91,180
		Foot grip reflex		
		normal, both sides	PRED	6
			DSNG	91,180

Table
Individual Neurological Examinations

Test Item	(dosage)	1 2 3 4 5
[redacted]	[redacted]	[redacted]

Group/ Sex	Animal Number	Observation	Phase	Day(s)
2/F	P0603	General observations		
		normal behavior	PRED	6
			DSNG	91,180
		Pupillary reflex		
		normal, both sides	PRED	6
			DSNG	91,180
		Orbicularis-oculi-reflex		
		normal, both sides	PRED	6
			DSNG	91,180
		Patellar reflex		
		normal, both sides	PRED	6
			DSNG	91,180
		Anal reflex		
		normal	PRED	6
			DSNG	91,180
		Foot grip reflex		
		normal, both sides	PRED	6
			DSNG	91,180

Table
Individual Neurological Examinations
Test Item (dosage) 1 2 3 4 5

[redacted] [redacted] [redacted]

Group/ Sex	Animal Number	Observation	Phase	Day(s)
2/F	P0604	General observations		
		normal behavior	PRED	6
			DSNG	91,180
		Pupillary reflex		
		normal, both sides	PRED	6
			DSNG	91,180
		Orbicularis-oculi-reflex		
		normal, both sides	PRED	6
			DSNG	91,180
		Patellar reflex		
		normal, both sides	PRED	6
			DSNG	91,180
		Anal reflex		
		normal	PRED	6
			DSNG	91,180
		Foot grip reflex		
		normal, both sides	PRED	6
			DSNG	91,180

Table
Individual Neurological Examinations

Test Item	(dosage)	1 2 3 4 5
[redacted]	[redacted]	[redacted]

Group/ Sex	Animal Number	Observation	Phase	Day(s)
3/F	P0701	General observations		
		normal behavior	PRED	6
			DSNG	91,180
		Pupillary reflex		
		normal, both sides	PRED	6
			DSNG	91,180
		Orbicularis-oculi-reflex		
		normal, both sides	PRED	6
			DSNG	91,180
		Patellar reflex		
		normal, both sides	PRED	6
			DSNG	91,180
		Anal reflex		
		normal	PRED	6
			DSNG	91,180
		Foot grip reflex		
		normal, both sides	PRED	6
			DSNG	91,180

Table
Individual Neurological Examinations

Test Item	(dosage)	1 2 3 4 5
[redacted]	[redacted]	[redacted]

Group/ Sex	Animal Number	Observation	Phase	Day(s)
3/F	P0702	General observations		
		normal behavior	PRED	6
			DSNG	91,180
		Pupillary reflex		
		normal, both sides	PRED	6
			DSNG	91,180
		Orbicularis-oculi-reflex		
		normal, both sides	PRED	6
			DSNG	91,180
		Patellar reflex		
		normal, both sides	PRED	6
			DSNG	91,180
		Anal reflex		
		normal	PRED	6
			DSNG	91,180
		Foot grip reflex		
		normal, both sides	PRED	6
			DSNG	91,180

Table
Individual Neurological Examinations

Test Item	(dosage)	1 2 3 4 5
[redacted]	[redacted]	[redacted]

Group/ Sex	Animal Number	Observation	Phase	Day(s)
3/F	P0703	General observations		
		normal behavior	PRED	6
			DSNG	91,180
		Pupillary reflex		
		normal, both sides	PRED	6
			DSNG	91,180
		Orbicularis-oculi-reflex		
		normal, both sides	PRED	6
			DSNG	91,180
		Patellar reflex		
		normal, both sides	PRED	6
			DSNG	91,180
		Anal reflex		
		normal	PRED	6
			DSNG	91,180
		Foot grip reflex		
		normal, both sides	PRED	6
			DSNG	91,180

Table
Individual Neurological Examinations
Test Item (dosage) 1 2 3 4 5

[redacted] [redacted] [redacted]

Group/ Sex	Animal Number	Observation	Phase	Day(s)
3/F	P0704	General observations		
		normal behavior	PRED	6
			DSNG	91,180
		Pupillary reflex		
		normal, both sides	PRED	6
			DSNG	91,180
		Orbicularis-oculi-reflex		
		normal, both sides	PRED	6
			DSNG	91,180
		Patellar reflex		
		normal, both sides	PRED	6
			DSNG	91,180
		Anal reflex		
		normal	PRED	6
			DSNG	91,180
		Foot grip reflex		
		normal, both sides	PRED	6
			DSNG	91,180

Table
Individual Neurological Examinations

Test Item (dosage) 1 2 3 4 5

[redacted]

Group/ Sex	Animal Number	Observation	Phase	Day(s)
4/F	P0801	General observations		
		normal behavior	PRED	6
			DSNG	91,180
		Pupillary reflex		
		normal, both sides	PRED	6
			DSNG	91,180
		Orbicularis-oculi-reflex		
		normal, both sides	PRED	6
			DSNG	91,180
		Patellar reflex		
		normal, both sides	PRED	6
			DSNG	91,180
		Anal reflex		
		normal	PRED	6
			DSNG	91,180
		Foot grip reflex		
		normal, both sides	PRED	6
			DSNG	91,180

Table
Individual Neurological Examinations

Test Item	(dosage)	1 2 3 4 5
[redacted]	[redacted]	[redacted]

Group/ Sex	Animal Number	Observation	Phase	Day(s)
4/F	P0802	General observations		
		normal behavior	PRED	6
			DSNG	91,180
		Pupillary reflex		
		normal, both sides	PRED	6
			DSNG	91,180
		Orbicularis-oculi-reflex		
		normal, both sides	PRED	6
			DSNG	91,180
		Patellar reflex		
		normal, both sides	PRED	6
			DSNG	91,180
		Anal reflex		
		normal	PRED	6
			DSNG	91,180
		Foot grip reflex		
		normal, both sides	PRED	6
			DSNG	91,180

Table
Individual Neurological Examinations

Test Item	(dosage)	1 2 3 4 5
[redacted]	[redacted]	[redacted]

Group/ Sex	Animal Number	Observation	Phase	Day(s)
4/F	P0803	General observations		
		normal behavior	PRED	6
			DSNG	91,180
		Pupillary reflex		
		normal, both sides	PRED	6
			DSNG	91,180
		Orbicularis-oculi-reflex		
		normal, both sides	DSNG	180
		Patellar reflex		
		normal, both sides	PRED	6
			DSNG	91,180
		Anal reflex		
		normal	PRED	6
			DSNG	91,180
		Foot grip reflex		
		normal, both sides	PRED	6
			DSNG	91,180

Table
Individual Neurological Examinations
Test Item (dosage) 1 2 3 4 5
---------- ------------------ - - -- -- ---
[redacted] [redacted] [redacted]

Group/ Sex	Animal Number	Observation	Phase	Day(s)
4/F	P0804	General observations		
		normal behavior	PRED	6
			DSNG	91,180
		Pupillary reflex		
		normal, both sides	PRED	6
			DSNG	91,180
		Orbicularis-oculi-reflex		
		normal, both sides	PRED	6
			DSNG	91,180
		Patellar reflex		
		normal, both sides	PRED	6
			DSNG	91,180
		Anal reflex		
		normal	PRED	6
			DSNG	91,180
		Foot grip reflex		
		normal, both sides	PRED	6
			DSNG	91,180

Table
Individual Neurological Examinations

Test Item	(dosage)	1 2 3 4 5
[redacted]	[redacted]	[redacted]

Group/ Sex	Animal Number	Observation	Phase	Day(s)
5/F	P0901	General observations		
		normal behavior	PRED	6
			DSNG	91,180
		Pupillary reflex		
		normal, both sides	PRED	6
			DSNG	91,180
		Orbicularis-oculi-reflex		
		normal, both sides	PRED	6
			DSNG	91,180
		Patellar reflex		
		normal, both sides	PRED	6
			DSNG	91,180
		Anal reflex		
		normal	PRED	6
			DSNG	91,180
		Foot grip reflex		
		normal, both sides	PRED	6
			DSNG	91,180

Table
Individual Neurological Examinations

Test Item	(dosage)	1 2 3 4 5
[redacted]	[redacted]	[redacted]

Group/ Sex	Animal Number	Observation	Phase	Day(s)
5/F	P0902	General observations		
		normal behavior	PRED	6
			DSNG	91,180
		Pupillary reflex		
		normal, both sides	PRED	6
			DSNG	91,180
		Orbicularis-oculi-reflex		
		normal, both sides	PRED	6
			DSNG	91,180
		Patellar reflex		
		normal, both sides	PRED	6
			DSNG	91,180
		Anal reflex		
		normal	PRED	6
			DSNG	91,180
		Foot grip reflex		
		normal, both sides	PRED	6
			DSNG	91,180

Table
Individual Neurological Examinations
Test Item (dosage) 1 2 3 4 5
---------- ------------------ - - -- -- ---
[redacted] [redacted] [redacted]

Group/ Sex	Animal Number	Observation	Phase	Day(s)
5/F	P0903	General observations		
		normal behavior	PRED	6
			DSNG	91,180
		Pupillary reflex		
		normal, both sides	PRED	6
			DSNG	91,180
		Orbicularis-oculi-reflex		
		normal, both sides	PRED	6
			DSNG	91,180
		Patellar reflex		
		normal, both sides	PRED	6
			DSNG	91,180
		Anal reflex		
		normal	PRED	6
			DSNG	91,180
		Foot grip reflex		
		normal, both sides	PRED	6
			DSNG	91,180

Table
Individual Neurological Examinations

Test Item	(dosage)	1 2 3 4 5
[redacted]	[redacted]	[redacted]

Group/ Sex	Animal Number	Observation	Phase	Day(s)
5/F	P0904	General observations		
		normal behavior	PRED	6
			DSNG	91,180
		Pupillary reflex		
		normal, both sides	PRED	6
			DSNG	91,180
		Orbicularis-oculi-reflex		
		normal, both sides	PRED	6
			DSNG	91,180
		Patellar reflex		
		normal, both sides	PRED	6
			DSNG	91
		not detectable, both sides	DSNG	180
		Anal reflex		
		normal	PRED	6
			DSNG	91,180
		Foot grip reflex		
		normal, both sides	PRED	6
			DSNG	91,180

Table
Individual Neurological Examinations

Test Item	(dosage)	1 2 3 4 5
[redacted]	[redacted]	[redacted]

Group/ Sex	Animal Number	Observation	Phase	Day(s)
5/F	P0905	General observations		
		normal behavior	PRED	6
			DSNG	91,180
			RECO	27
		Pupillary reflex		
		normal, both sides	PRED	6
			DSNG	91,180
			RECO	27
		Orbicularis-oculi-reflex		
		normal, both sides	PRED	6
			DSNG	91,180
			RECO	27
		Patellar reflex		
		normal, both sides	PRED	6
			DSNG	91,180
			RECO	27
		Anal reflex		
		normal	PRED	6
			DSNG	91,180
			RECO	27
		Foot grip reflex		
		normal, both sides	PRED	6
			DSNG	91,180
			RECO	27

Table
Individual Neurological Examinations

Test Item	(dosage)	1 2 3 4 5
[redacted]	[redacted]	[redacted]

Group/ Sex	Animal Number	Observation	Phase	Day(s)
5/F	P0906	General observations		
		normal behavior	PRED	6
			DSNG	91,180
			RECO	27
		Pupillary reflex		
		normal, both sides	PRED	6
			DSNG	91,180
			RECO	27
		Orbicularis-oculi-reflex		
		normal, both sides	PRED	6
			DSNG	91,180
			RECO	27
		Patellar reflex		
		normal, both sides	PRED	6
			DSNG	91
			RECO	27
		not detectable, both sides	DSNG	180
		Anal reflex		
		normal	PRED	6
			DSNG	91,180
			RECO	27

Table
Individual Neurological Examinations
Test Item (dosage)   1 2 3 4 5
---------- ------------------ - - -- -- ---
[redacted]

Group/ Sex	Animal Number	Observation	Phase	Day(s)
5/F	P0906	Foot grip reflex		
		normal, both sides	PRED	6
			DSNG	91,180
			RECO	27

Data in Support of Table 2: Study I, Salivation

[redacted]

Test Item (dosage) 1 2 3 4

[redacted] [redacted]

Group/ Sex	Animal Number	Observation	Phase	Day(s)
1/M	P0001	NORMAL		
		No visible finding	DSNG	1-90,92
1/M	P0002	NORMAL		
		No visible finding	DSNG	1-90,92
		Mouth/ vomiting		
		lesion/s, right angle of mouth, bloody	DSNG	6,21
1/M	P0003	NORMAL		
		No visible finding	DSNG	1-90,92
2/M	P0101	NORMAL		
		No visible finding	DSNG	1-90,92
2/M	P0102	NORMAL		
		No visible finding	DSNG	1-90,92
		Mouth/ vomiting		
		emesis of liquid, after dosing, mucoid	DSNG	21
2/M	P0103	NORMAL		
		No visible finding	DSNG	1-90,92
3/M	P0201	Eye/s		
		lesion/s, left eyebrow, bloody	DSNG	1
		lesion/s, left eyebrow, wet	DSNG	2,3
		lesion/s, left eyebrow, crusted	DSNG	4-9
		NORMAL		
		No visible finding	DSNG	10-90,92

Table
Individual Clinical Observations
Test Item (dosage) 1 2 3 4
--------- -------- - - -- --
[redacted] [redacted]

Group/ Sex	Animal Number	Observation	Phase	Day(s)
3/M	P0202	NORMAL		
		No visible finding	DSNG	1-90,92
3/M	P0203	NORMAL		
		No visible finding	DSNG	1-90,92
		Mouth/ vomiting		
		excessive salivation, before dosing, little amount	DSNG	26,52,53,55,81,83
		excessive salivation, after dosing, little amount	DSNG	63,81,83
		excessive salivation, little amount, after dosing	DSNG	70
		excessive salivation, little amount, before dosing	DSNG	70
4/M	P0301	NORMAL		
		No visible finding	DSNG	1-90,92
		Mouth/ vomiting		
		excessive salivation, after dosing, little amount	DSNG	23,67

[redacted]

Table
Individual Clinical Observations
Test Item (dosage) 1 2 3 4
--------- -------- - - -- --
[redacted] [redacted]

Group/ Sex	Animal Number	Observation	Phase	Day(s)
4/M	P0302	Mouth/ vomiting		
		lesion/s, upper lip, wet	DSNG	1,2
		lesion/s, upper lip, crusted	DSNG	3-9
		NORMAL		
		No visible finding	DSNG	10-90,92
		Mouth/ vomiting		
		excessive salivation, before dosing, little amount	DSNG	63
4/M	P0303	NORMAL		
		No visible finding	DSNG	1-90,92
		Mouth/ vomiting		
		excessive salivation, after dosing, mucoid	DSNG	7,10
		excessive salivation, before dosing, large amount	DSNG	18-20,23-26,34,35,37,40,41, 44,45,49,52-55,58,61,63,65, 67,69,70,77,79,80,83,84,86
		excessive salivation, before dosing, little amount	DSNG	30
		excessive salivation, large amount, before dosing	DSNG	88
1/F	P0401	NORMAL		
		No visible finding	DSNG	1-90,92,93

[redacted]

Table
Individual Clinical Observations
Test Item (dosage) 1 2 3 4
--------- -------- - - -- --
[redacted] [redacted]

Group/ Sex	Animal Number	Observation	Phase	Day(s)
1/F	P0402	NORMAL		
		No visible finding	DSNG	1-90,92,93
1/F	P0403	NORMAL		
		No visible finding	DSNG	1-90,92,93
2/F	P0501	NORMAL		
		No visible finding	DSNG	1-90,92,93
2/F	P0502	NORMAL		
		No visible finding	DSNG	1-90,92,93
2/F	P0503	NORMAL		
		No visible finding	DSNG	1-90,92,93
3/F	P0601	NORMAL		
		No visible finding	DSNG	1-90,92,93
3/F	P0602	NORMAL		
		No visible finding	DSNG	1-90,92,93
3/F	P0603	NORMAL		
		No visible finding	DSNG	1-90,92,93
		Mouth/ vomiting		
		emesis of liquid, after dosing	DSNG	37,63
4/F	P0701	NORMAL		
		No visible finding	DSNG	1-3,10-90,92,93
		Eye/s		
		lesion/s, right eyebrow, crusted	DSNG	4-9

[redacted]

Table
Individual Clinical Observations
Test Item (dosage) 1 2 3 4
--------- -------- - - -- --
[redacted] [redacted]

Group/ Sex	Animal Number	Observation	Phase	Day(s)
4/F	P0702	NORMAL		
		No visible finding	DSNG	1-90,92,93
4/F	P0703	NORMAL		
		No visible finding	DSNG	1-90,92,93

Data in Support of Table 2: Study I, Soft feces

[redacted]

Test Item (dosage) 1 2 3 4

[redacted] [redacted]

Group/ Sex	Animal Number	Observation	Phase	Day(s)
1/M	P0001	Excretion		
		feces: normal (1.observation)	DSNG	1-92
1/M	P0002	Excretion		
		feces: normal (1.observation)	DSNG	1-92
1/M	P0003	Excretion		
		feces: normal (1.observation)	DSNG	1-37,39-92
		feces: liquid feces (1.observation)	DSNG	38
2/M	P0101	Excretion		
		feces: normal (1.observation)	DSNG	1-9,11-92
		feces: soft feces (1.observation)	DSNG	10
2/M	P0102	Excretion		
		feces: normal (1.observation)	DSNG	1-9,11-92
		feces: soft feces (1.observation)	DSNG	10
2/M	P0103	Excretion		
		feces: normal (1.observation)	DSNG	1-9,11-92
		feces: soft feces (1.observation)	DSNG	10
3/M	P0201	Excretion		
		feces: normal (1.observation)	DSNG	1-8,11-92
		feces: soft feces (1.observation)	DSNG	9,10

[redacted]

Table
Individual Clinical Observations
Test Item (dosage) 1 2 3 4
--------- -------- - - -- --
[redacted] [redacted]

Group/ Sex	Animal Number	Observation	Phase	Day(s)
3/M	P0202	Excretion		
		feces: normal (1.observation)	DSNG	1-8,11-92
		feces: soft feces (1.observation)	DSNG	9,10
3/M	P0203	Excretion		
		feces: normal (1.observation)	DSNG	1-8,11-92
		feces: soft feces (1.observation)	DSNG	9,10
4/M	P0301	Excretion		
		feces: normal (1.observation)	DSNG	1-8,17-22,32-50,56
		feces: soft feces (1.observation)	DSNG	9-16,23-31,53-55,58-88
		feces: liquid feces (1.observation)	DSNG	51,52,57,89-92
4/M	P0302	Excretion		
		feces: normal (1.observation)	DSNG	1-51,56-92
		feces: soft feces (1.observation)	DSNG	52-55
4/M	P0303	Excretion		
		feces: normal (1.observation)	DSNG	1-8,17-51,56-92
		feces: soft feces (1.observation)	DSNG	9-16,52-55
1/F	P0401	Excretion		
		feces: normal (1.observation)	DSNG	1-8,10-93
		feces: soft feces (1.observation)	DSNG	9

[redacted]

Table
Individual Clinical Observations
Test Item (dosage) 1 2 3 4
--------- -------- - - -- --
[redacted] [redacted]

Group/ Sex	Animal Number	Observation	Phase	Day(s)
1/F	P0402	Excretion		
		feces: normal (1.observation)	DSNG	1-8,10-93
		feces: soft feces (1.observation)	DSNG	9
1/F	P0403	Excretion		
		feces: normal (1.observation)	DSNG	1-8,10-93
		feces: soft feces (1.observation)	DSNG	9
2/F	P0501	Excretion		
		feces: normal (1.observation)	DSNG	1-93
2/F	P0502	Excretion		
		feces: normal (1.observation)	DSNG	1-93
2/F	P0503	Excretion		
		feces: normal (1.observation)	DSNG	1-93
3/F	P0601	Excretion		
		feces: normal (1.observation)	DSNG	1-8,11-93
		feces: soft feces (1.observation)	DSNG	9,10
3/F	P0602	Excretion		
		feces: normal (1.observation)	DSNG	1-8,11-39,43-93
		feces: soft feces (1.observation)	DSNG	9,10,40-42

[redacted]

Table
Individual Clinical Observations
Test Item (dosage) 1 2 3 4
--------- -------- - - -- --
[redacted] [redacted]

Group/ Sex	Animal Number	Observation	Phase	Day(s)
3/F	P0603	Excretion		
		feces: normal (1.observation)	DSNG	1-8,11-39,43-50,52,56,82-93
		feces: soft feces (1.observation)	DSNG	9,10,42,51,53-55,58-81
		feces: liquid feces (1.observation)	DSNG	40,41,57
4/F	P0701	Excretion		
		feces: normal (1.observation)	DSNG	1-3,12-37,39-50,52,56-81, 90-93
		feces: soft feces (1.observation)	DSNG	9-11,38,51,53-55,82-89
		feces: liquid feces (1.observation)	DSNG	4-8
4/F	P0702	Excretion		
		feces: normal (1.observation)	DSNG	1-8,12-37,39-52,56-93
		feces: soft feces (1.observation)	DSNG	9-11,38,53-55
4/F	P0703	Excretion		
		feces: normal (1.observation)	DSNG	1-8,12-22,24-37,39-52,56-81, 90-93
		feces: soft feces (1.observation)	DSNG	9-11,23,38,53-55,82-88
		feces: liquid feces (1.observation)	DSNG	89

Data in Support of Table 2: Study I, Clinical obs (skin)

[redacted]

Test Item (dosage) 1 2 3 4
--------- -------- - - -- --
[redacted] [redacted]

Group/ Sex	Animal Number	Observation	Phase	Day(s)
1/M	P0001	NORMAL		
		No remarkable observations	PRED	7
			DSNG	1,8,15,22,29,36,43,50,57,64, 71,78,85
		Trunk		
		lesion/s, tail distal, crusted	DSNG	91
1/M	P0002	NORMAL		
		No remarkable observations	PRED	7
			DSNG	1,36,43,50,57,64,71,78,85,91
		Mouth/ vomiting		
		lesion/s, right angle of mouth, bloody	DSNG	8
		lesion/s, right angle of mouth, crusted	DSNG	15,22
		Head		
		lesion/s, forehead, crusted	DSNG	29

[redacted]

Table
Individual Clinical Observations
Test Item (dosage) 1 2 3 4
--------- -------- - - -- --
[redacted] [redacted]

Group/ Sex	Animal Number	Observation	Phase	Day(s)
1/M	P0003	NORMAL		
		No remarkable observations	PRED	7
			DSNG	1,8,15,29,57,64,71,78,85,91
		Head		
		lesion/s, forehead, crusted	DSNG	22
		Trunk		
		lesion/s, tail, crusted	DSNG	36,43,50
		Head		
		lesion/s, right side of head, bloody	DSNG	50
2/M	P0101	NORMAL		
		No remarkable observations	PRED	7
			DSNG	1,8,15,22,29,36,43,50,57,64, 71,78,85,91
2/M	P0102	NORMAL		
		No remarkable observations	PRED	7
			DSNG	1,8,15,22,29,36,43,50,57,64, 71,78,85,91
2/M	P0103	NORMAL		
		No remarkable observations	PRED	7
			DSNG	1,8,15,22,50,57,64,71,78,85, 91
		Trunk		
		lesion/s, tail, crusted	DSNG	29,36,43

[redacted]

Table
Individual Clinical Observations
Test Item (dosage) 1 2 3 4
--------- -------- - - -- --

[redacted] [redacted]

Group/ Sex	Animal Number	Observation	Phase	Day(s)
3/M	P0201	NORMAL		
		No remarkable observations	PRED	7
			DSNG	1,8,15,22,29,36,43,50,57,64, 71,78,85,91
3/M	P0202	NORMAL		
		No remarkable observations	PRED	7
			DSNG	1,8,15,22,29,36,43,50,57,64, 71,78,85,91
3/M	P0203	NORMAL		
		No remarkable observations	PRED	7
			DSNG	1,8,15,22,29,36,43,50,57,64, 71,78,85
		Trunk		
		lesion/s, right inguen, crusted	DSNG	91
4/M	P0301	NORMAL		
		No remarkable observations	PRED	7
			DSNG	1,8,15,29,36,43,50,57,64,71, 78,85,91
		Extremity/ies		
		squamous skin, slight, both arms, large area	DSNG	22

[redacted]

Table
Individual Clinical Observations
Test Item (dosage) 1 2 3 4
--------- -------- - - -- --
[redacted] [redacted]

Group/ Sex	Animal Number	Observation	Phase	Day(s)
4/M	P0302	NORMAL		
		No remarkable observations	PRED	7
			DSNG	1,15,22,29,36,43,50,57,64, 71,78,85
		Extremity/ies		
		discolored skin, left lower arm, blue, large area	DSNG	8
		Mouth/ vomiting		
		lesion/s, lower lip, crusted	DSNG	91

[redacted]

Table
Individual Clinical Observations
Test Item (dosage) 1 2 3 4
--------- -------- - - -- --
[redacted] [redacted]

Group/ Sex	Animal Number	Observation	Phase	Day(s)
4/M	P0303	NORMAL		
		No remarkable observations	PRED	7
			DSNG	1,8
		Mouth/ vomiting		
		excessive salivation, before dosing, mucoid	DSNG	15,22,50,57,64,78,91
		Extremity/ies		
		squamous skin, slight, right arm, large area	DSNG	22
		Mouth/ vomiting		
		excessive salivation, mucoid, before dosing	DSNG	29,36,43,71,85
		Extremity/ies		
		squamous skin, slight, both arms, large area	DSNG	29,36,43,50,57
		discolored skin, both arms, grey, large area	DSNG	36,43,50,57,64,71
		Mouth/ vomiting		
		excessive salivation, large amount, after dosing	DSNG	85
		excessive salivation, after dosing, large amount	DSNG	91

[redacted]

Table
Individual Clinical Observations
Test Item (dosage) 1 2 3 4
--------- -------- - - -- --
[redacted] [redacted]

Group/ Sex	Animal Number	Observation	Phase	Day(s)
1/F	P0401	NORMAL		
		No remarkable observations	PRED	7
			DSNG	1,15,22,29,36,71,78,85,91
		Trunk		
		lesion/s, upper part of back, crusted	DSNG	8
		Behavior/appearance		
		thin	DSNG	43,50,57,64
1/F	P0402	NORMAL		
		No remarkable observations	PRED	7
			DSNG	1,8,22,29
		Head		
		discolored skin, chin, red, small area	DSNG	15
		Behavior/appearance		
		thin	DSNG	36,43,50,57,64,71,78,85,91
1/F	P0403	NORMAL		
		No remarkable observations	PRED	7
		Trunk		
		lesion/s, upper part of back, crusted	DSNG	1,8
		NORMAL		
		No remarkable observations	DSNG	15,22,29,36,43,57,64,71,78, 85,91
		Head		
		lesion/s, forehead, bloody	DSNG	50

[redacted]

Table
Individual Clinical Observations
Test Item (dosage) 1 2 3 4
--------- -------- - - -- --

[redacted] [redacted]

Group/ Sex	Animal Number	Observation	Phase	Day(s)
2/F	P0501	NORMAL		
		No remarkable observations	PRED	7
			DSNG	1,8,29,36,50,57,85,91
		Trunk		
		lesion/s, tail, bloody	DSNG	15,22
		lesion/s, tail, crusted	DSNG	43
		lesion/s, tail distal, crusted	DSNG	64,71,78
2/F	P0502	NORMAL		
		No remarkable observations	PRED	7
		Head		
		lesion/s, nose, crusted	DSNG	1
		NORMAL		
		No remarkable observations	DSNG	8,15,22,36,43,50,57,64,71, 78,85,91
		Trunk		
		squamous skin, slight, chest, small area	DSNG	29
2/F	P0503	NORMAL		
		No remarkable observations	PRED	7
			DSNG	1,8
		Trunk		
		lesion/s, tail, crusted	DSNG	15,22,29,36,43,50
		lesion/s, tail distal, bloody	DSNG	57
		lesion/s, tail distal, crusted	DSNG	64,71,78,85,91

[redacted]

Table
Individual Clinical Observations
Test Item (dosage) 1 2 3 4

[redacted] [redacted]

Group/ Sex	Animal Number	Observation	Phase	Day(s)
3/F	P0601	NORMAL		
		No remarkable observations	PRED	7
			DSNG	1,8,15,22,29,36,43,50,57,64, 78,85,91
		Trunk		
		discolored skin, both inguinal, orange, small area	DSNG	71
3/F	P0602	NORMAL		
		No remarkable observations	PRED	7
			DSNG	1,8,15,22,29,36,43,50,57,64, 71,78,85,91
3/F	P0603	NORMAL		
		No remarkable observations	PRED	7
			DSNG	1,8,15
		Behavior/appearance		
		thin	DSNG	22,29,36,43,50,57,64,71,78, 85,91

[redacted]

Table
Individual Clinical Observations
Test Item (dosage) 1 2 3 4
--------- -------- - - -- --
[redacted] [redacted]

Group/ Sex	Animal Number	Observation	Phase	Day(s)
4/F	P0701	NORMAL		
		No remarkable observations	PRED	7
		Trunk		
		lesion/s, lower part of back, crusted	DSNG	1,15,57,64,71,78
		Head		
		lesion/s, forehead, crusted	DSNG	8
		swelling/s, slight, nose, indurated	DSNG	8,15,22,29,36,43,50
		Trunk		
		squamous skin, slight, both shoulders, large area	DSNG	15,22,29,36
		squamous skin, slight, upper abdomen, large area	DSNG	15,22,29,36
		Head		
		discolored skin, nose, red, small area	DSNG	29
		Extremity/ies		
		squamous skin, slight, both arms, large area	DSNG	36
		Behavior/appearance		
		thin	DSNG	78,85,91
		Trunk		
		lesion/s, tip of tail, crusted	DSNG	85

[redacted]

Table
Individual Clinical Observations
Test Item (dosage) 1 2 3 4
--------- -------- - - -- --
[redacted] [redacted]

Group/ Sex	Animal Number	Observation	Phase	Day(s)
4/F	P0701	Mouth/ vomiting		
		excessive salivation, after dosing, mucoid	DSNG	91
		excessive salivation, large amount, after dosing	DSNG	91
		Trunk		
		lesion/s, tip of tail, bloody	DSNG	91
4/F	P0702	NORMAL		
		No remarkable observations	PRED	7
			DSNG	1,8,43,50,57,64,71,78,85,91
		Extremity/ies		
		squamous skin, severe, both arms, large area	DSNG	15,22,29
		Trunk		
		squamous skin, severe, both axillae, large area	DSNG	22,29
		squamous skin, severe, upper abdomen, large area	DSNG	22,29
		squamous skin, slight, both axillae, large area	DSNG	36
		squamous skin, slight, upper abdomen, large area	DSNG	36
		Extremity/ies		
		squamous skin, slight, both arms, large area	DSNG	36

[redacted]

Table
Individual Clinical Observations
Test Item (dosage) 1 2 3 4
--------- -------- - - -- --
[redacted] [redacted]

Group/ Sex	Animal Number	Observation	Phase	Day(s)
4/F	P0703	NORMAL		
		No remarkable observations	PRED	7
		Eye/s		
		spots, left eye, blue	DSNG	1
		NORMAL		
		No remarkable observations	DSNG	8,78,85,91
		Trunk		
		squamous skin, severe, both axillae, small area	DSNG	15,22,29,36
		squamous skin, slight, both inguinal, large area	DSNG	15,22,29,36
		Extremity/ies		
		squamous skin, severe, both arms, large area	DSNG	15,22
		Trunk		
		squamous skin, severe, whole abdomen, large area	DSNG	22
		squamous skin, slight, whole abdomen, small area	DSNG	29,36
		Extremity/ies		
		squamous skin, slight, both arms, large area	DSNG	29,36

[redacted]

Table
Individual Clinical Observations
Test Item (dosage) 1 2 3 4
--------- -------- - - -- --

[redacted] [redacted]

Group/ Sex	Animal Number	Observation	Phase	Day(s)
4/F	P0703	Trunk		
		squamous skin, slight, lower abdomen, large area	DSNG	43,50,57
		discolored skin, lower abdomen, orange, large area	DSNG	50,57,64,71
		discolored skin, neck, red, small area	DSNG	71

Data in Support of Table 2: Study I, Serum cholesterole

[redacted]

Table
Summary of Clinical Chemistry
Test Item (dosage) 1 2 3 4
[redacted] [redacted]

Group/ Sex	Phase / Day	GLU. mmol/L Predose 8	GLU. mmol/L Dosing 26	GLU. mmol/L Dosing 91	CHOL. mmol/L Predose 8	CHOL. mmol/L Dosing 26	CHOL. mmol/L Dosing 91
1/M	Mean	3.94	4.73	4.47	2.38	2.60	2.78
	SD	0.639	0.854	0.254	0.400	0.221	0.427
	N	3	3	3	3	3	3
2/M	Mean	3.33	4.05	4.29	4.03	3.54	3.80
	SD	0.382	0.505	0.505	0.960	0.626	0.515
	N	3	3	3	3	3	3
3/M	Mean	3.69	3.91	4.51	3.54	2.86	3.42
	SD	0.803	0.358	0.714	1.057	0.504	0.937
	N	3	3	3	3	3	3
4/M	Mean	3.44	4.07	3.95	3.97	2.22	2.23
	SD	0.140	0.481	0.271	1.101	0.816	0.710
	N	3	3	3	3	3	3
	Statistics	AT	A	A	A	A	A

A = ANOVA and Dunnett's
T = Rank-transformed data

[redacted]

Table
Summary of Clinical Chemistry
Test Item (dosage) 1 2 3 4
[redacted] [redacted]

Group/ Sex	Phase / Day	GLU. mmol/L Predose 8	GLU. mmol/L Dosing 26	GLU. mmol/L Dosing 91	CHOL. mmol/L Predose 8	CHOL. mmol/L Dosing 26	CHOL. mmol/L Dosing 91
1/F	Mean	3.41	4.15	4.23	4.30	4.56	4.95
	SD	0.857	0.348	0.206	1.172	1.484	1.376
	N	3	3	3	3	3	3
2/F	Mean	3.90	3.86	5.29	3.62	3.53	3.91
	SD	0.962	0.365	1.743	0.411	0.287	0.557
	N	3	3	3	3	3	3
3/F	Mean	3.88	3.43	3.97	4.06	3.84	3.76
	SD	0.998	0.505	0.263	1.405	1.262	1.167
	N	3	3	3	3	3	3
4/F	Mean	3.55	3.86	4.07	3.22	1.98	2.15
	SD	0.569	0.167	0.115	0.521	0.736	0.820
	N	3	3	3	3	3	3
	Statistics	A	A	AT	A	A	A

A = ANOVA and Dunnett's
T = Rank-transformed data

Data in Support of Table 2: Study I, Organ weight (liver)

[redacted]

Summary of Organ Weights and Organ Weight Ratios
Terminal Sacrifice
[redacted] t Item [redacted]

Group/ Sex		Terminal Body Weight (g)	[redacted] Unadjusted (g)	[redacted] Body Weight (%)	[redacted] Brain Weight (%)	Liver Unadjusted (g)	Liver Body Weight (%)	Liver Brain Weight (%)
1/M	Mean	6067	19.184	0.3159	27.5324	97.674	1.6204	140.8334
	SD	874.7	2.9654	0.00355	1.94833	7.7474	0.11841	6.24050
	N	3	3	3	3	3	3	3
2/M	Mean	5165	17.062	0.3315	22.7190	87.389	1.7111	116.3942*
	SD	1184.9	3.8345	0.02958	3.60716	13.1033	0.13001	5.34050
	N	3	3	3	3	3	3	3
	%-Diff	-15%	-11%	5%	-17%	-11%	6%	-17%
3/M	Mean	5560	19.990	0.3577	27.5564	106.595	1.9073*	146.8132
	SD	962.9	4.9958	0.04451	6.48164	24.0608	0.11481	30.11467
	N	3	3	3	3	3	3	3
	%-Diff	-8%	4%	13%	0%	9%	18%	4%
4/M	Mean	6472	21.073	0.3273	31.5693	132.853	2.0469**	199.0018
	SD	603.3	0.2920	0.02793	0.50647	18.7549	0.09666	28.06548
	N	3	3	3	3	3	3	3
	%-Diff	7%	10%	4%	15%	36%	26%	41%
	Statistics	A	A	A	A	A	A	AT

* P<=0.05
** P<=0.01
*** P<=0.001
A = ANOVA and Dunnett's
T = Rank-transformed data

[redacted]

Summary of Organ Weights and Organ Weight Ratios
Terminal Sacrifice
[redacted]t Item [redacted]

Group/ Sex		Terminal Body Weight (g)	[redacted] Unadjusted (g)	Body Weight (%)	Brain Weight (%)	Liver Unadjusted (g)	Body Weight (%)	Brain Weight (%)
1/F	Mean	2970	12.273	0.4117	19.5220	58.177	1.9596	93.9296
	SD	212.5	2.3651	0.06027	2.01338	4.5146	0.09267	13.76966
	N	3	3	3	3	3	3	3
2/F	Mean	3007	11.800	0.3905	18.5536	63.868	2.1273	100.9395
	SD	212.2	2.9647	0.07837	3.91703	2.6242	0.06637	1.69946
	N	3	3	3	3	3	3	3
	%-Diff	1%	-4%	-5%	-5%	10%	9%	7%
3/F	Mean	3035	10.402	0.3447	16.0103	61.979	2.0494	96.2057
	SD	456.4	1.3851	0.04017	1.48321	7.0147	0.07735	15.72788
	N	3	3	3	3	3	3	3
	%-Diff	2%	-15%	-16%	-18%	7%	5%	2%
4/F	Mean	3260	11.232	0.3524	21.4167	73.781	2.2683*	142.4238
	SD	553.4	0.7510	0.07124	2.11709	11.7185	0.13186	37.14217
	N	3	3	3	3	3	3	3
	%-Diff	10%	-8%	-14%	10%	27%	16%	52%
	Statistics	A	A	A	A	A	A	A

* P<=0.05
** P<=0.01
*** P<=0.001
A = ANOVA and Dunnett's

Data in Support of Table 2: Study I, Blood RBC

[redacted]

Table
Summary of Hematology
Test Item (dosage) 1 2 3 4
[redacted] [redacted]

Group/ Sex	Phase / Day	RBC. 10E12/L Predose 8	Dosing 26	Dosing 91	[redacted] Predose 8	Dosing 26	Dosing 91
1/M	Mean	6.01	5.69	6.07	9.3	8.9	9.4
	SD	0.239	0.232	0.550	0.40	0.32	0.64
	N	3	3	3	3	3	3
2/M	Mean	6.19	5.78	5.90	9.1	8.6	8.7
	SD	0.380	0.067	0.139	0.65	0.26	0.17
	N	3	3	3	3	3	3
3/M	Mean	6.02	5.57	5.66	8.7	8.1	8.1
	SD	0.969	0.682	0.848	0.85	0.67	0.72
	N	3	3	3	3	3	3
4/M	Mean	5.53	4.79	5.86	9.0	8.3	8.3
	SD	1.104	1.701	0.007	0.26	0.47	0.64
	N	3	3	2	3	3	3
	Statistics	AT	AT	A	A	A	A

A = ANOVA and Dunnett's
T = Rank-transformed data

[redacted]

Table
Summary of Hematology
Test Item (dosage) 1 2 3 4
[redacted] [redacted]

Group/ Sex	Phase / Day	RBC. 10E12/L Predose 8	RBC. 10E12/L Dosing 26	RBC. 10E12/L Dosing 91	[redacted] Predose 8	[redacted] Dosing 26	[redacted] Dosing 91
1/F	Mean	5.53	5.14	5.58	8.2	7.7	8.3
	SD	0.155	0.285	0.197	0.26	0.60	0.32
	N	3	3	3	3	3	3
2/F	Mean	6.38	5.63	5.93	8.9	8.1	8.5
	SD	0.474	0.431	0.142	0.65	0.68	0.46
	N	3	3	3	3	3	3
3/F	Mean	5.90	5.55	5.58	8.7	8.2	8.2
	SD	0.558	0.387	0.418	0.76	0.46	0.51
	N	3	3	3	3	3	3
4/F	Mean	5.63	5.21	5.29	8.5	7.8	7.9
	SD	0.301	0.390	0.291	0.10	0.35	0.06
	N	3	3	3	3	3	3
	Statistics	A	A	A	A	A	A

A = ANOVA and Dunnett's

Data in Support of Table 2: Study K, Mic (liver)

Incidence of Microscopic Observations
Terminal Necropsy Study Day 283
Test Item (dosage) 1 2 3 4
---------- ------------ - - -- --
[redacted]

Tissue/ Observation	Group/Sex: Number of Animals:	1/M 4	2/M 4	3/M 4	4/M 4	1/F 4	2/F 4	3/F 4	4/F 4
Kidney	Number Examined:	4	4	4	4	4	4	4	4
	Unremarkable:	1	1	0	0	1	1	0	0
[redacted]		0	0	0	4	0	0	0	2
[redacted]		0	0	0	0	0	0	1	0
[redacted]		0	0	0	0	0	0	1	0
[redacted]		3	3	4	3	3	3	4	4
[redacted]		1	0	0	0	0	0	0	0
Liver	Number Examined:	4	4	4	4	4	4	4	4
	Unremarkable:	3	1	2	0	2	3	1	0
Basophilic granules, Kupffer cells		0	0	0	4	0	0	0	4
Infiltrate, mixed cells		0	1	0	0	0	0	0	0
Infiltrate, mononuclear cells		1	2	2	4	2	1	3	2
Lung	Number Examined:	4	4	4	4	4	4	4	4
	Unremarkable:	4	4	3	4	4	3	3	4
[redacted]		0	0	0	0	0	1	1	0
[redacted]		0	0	1	0	0	1	1	0
Lymph Node, Axillary	Number Examined:	4	4	4	4	4	4	4	4
	Unremarkable:	4	1	2	0	3	2	1	0
[redacted]		0	3	2	4	0	2	3	4
[redacted]		0	1	1	0	1	0	1	1

Data in Support of Table 2: Study K, Mic (kidney)

Incidence of Microscopic Observations
Terminal Necropsy Study Day 283
Test Item (dosage) 1 2 3 4
---------- ------------ - - -- --
[redacted]

Tissue/ Observation	Group/Sex:	1/M	2/M	3/M	4/M	1/F	2/F	3/F	4/F
	Number of Animals:	4	4	4	4	4	4	4	4
Kidney	Number Examined:	4	4	4	4	4	4	4	4
	Unremarkable:	1	1	0	0	1	1	0	0
Basophilic granules, tubule		0	0	0	4	0	0	0	2
Basophilic, tubule		0	0	0	0	0	0	1	0
Fibrosis		0	0	0	0	0	0	1	0
Infiltrate, mononuclear cells		3	3	4	3	3	3	4	4
Inflammation, pelvis		1	0	0	0	0	0	0	0
Liver	Number Examined:	4	4	4	4	4	4	4	4
	Unremarkable:	3	1	2	0	2	3	1	0
[redacted]		0	0	0	4	0	0	0	4
[redacted]		0	1	0	0	0	0	0	0
[redacted]		1	2	2	4	2	1	3	2
Lung	Number Examined:	4	4	4	4	4	4	4	4
	Unremarkable:	4	4	3	4	4	3	3	4
[redacted]		0	0	0	0	0	1	1	0
[redacted]		0	0	1	0	0	1	1	0
Lymph Node, Axillary	Number Examined:	4	4	4	4	4	4	4	4
	Unremarkable:	4	1	2	0	3	2	1	0
[redacted]		0	3	2	4	0	2	3	4
[redacted]		0	1	1	0	1	0	1	1

Data in Support of Table 2: Study K, Mic (lymph node)

Incidence of Microscopic Observations
Terminal Necropsy Study Day 283
Test Item (dosage) 1 2 3 4

[redacted]

Tissue/ Observation	Group/Sex:	1/M	2/M	3/M	4/M	1/F	2/F	3/F	4/F
	Number of Animals:	4	4	4	4	4	4	4	4
Kidney	Number Examined:	4	4	4	4	4	4	4	4
	Unremarkable:	1	1	0	0	1	1	0	0
[redacted]		0	0	0	4	0	0	0	2
[redacted]		0	0	0	0	0	0	1	0
[redacted]		0	0	0	0	0	0	1	0
[redacted]		3	3	4	3	3	3	4	4
[redacted]		1	0	0	0	0	0	0	0
Liver	Number Examined:	4	4	4	4	4	4	4	4
	Unremarkable:	3	1	2	0	2	3	1	0
[redacted]		0	0	0	4	0	0	0	4
[redacted]		0	1	0	0	0	0	0	0
[redacted]		1	2	2	4	2	1	3	2
Lung	Number Examined:	4	4	4	4	4	4	4	4
	Unremarkable:	4	4	3	4	4	3	3	4
[redacted]		0	0	0	0	0	1	1	0
[redacted]		0	0	1	0	0	1	1	0
Lymph Node, Axillary	Number Examined:	4	4	4	4	4	4	4	4
	Unremarkable:	4	1	2	0	3	2	1	0
Granular macrophages		0	3	2	4	0	2	3	4
Hyperplasia, lymphoid		0	1	1	0	1	0	1	1

Incidence of Microscopic Observations
Terminal Necropsy Study Day 283
Test Item (dosage) 1 2 3 4
---------- ------------ - - -- --
[redacted]

Tissue/ Observation	Group/Sex: Number of Animals:	1/M 4	2/M 4	3/M 4	4/M 4	1/F 4	2/F 4	3/F 4	4/F 4
Lymph Node, Mandibular	Number Examined:	4	4	4	4	4	4	4	4
	Unremarkable:	4	3	4	0	4	3	3	2
Granular macrophages		0	0	0	4	0	0	1	2
Hyperplasia, lymphoid		0	1	0	0	0	1	0	0
Lymph Node, Mesenteric	Number Examined:	4	4	4	4	4	4	4	4
	Unremarkable:	4	3	2	2	4	3	2	0
Granular macrophages		0	1	2	2	0	1	2	4
Mammary Gland	Number Examined:	4	4	4	4	4	4	4	4
	Unremarkable:	4	4	4	4	4	4	4	4
Mandibular Salivary Gland	Number Examined:	4	4	4	4	4	4	4	4
	Unremarkable:	0	1	2	0	1	2	1	0
[redacted]		4	3	2	4	3	2	3	4
Muscle, Quadriceps, Left	Number Examined:	0	0	0	0	0	1	0	0
	Unremarkable:	0	0	0	0	0	1	0	0
Muscle, Skeletal	Number Examined:	4	4	4	4	4	4	4	4
	Unremarkable:	4	4	4	4	4	4	4	4

Data in Support of Table 2: Study L, Serum globulin

Table
Summary of Clinical Chemistry
Test Item (dosage) 1 2 3 4
[redacted] [redacted]

Group/ Sex	Study Day / Session / Name	GLOBgdL. g/dL -21 / 3 / CC	57 / 4 / 48:00	85 / 4 / 48:00	141 / 4 / 48:00	197 / 4 / 48:00	253 / 4 / 48:00
1/M	Mean	3.28	3.16	2.97	3.08	2.99	3.24
	SD	0.189	0.314	0.516	0.253	0.264	0.340
	N	6	6	6	6	6	6
2/M	Mean	2.97	2.87	2.54	2.77	2.58	3.06
	SD	0.377	0.235	0.255	0.335	0.311	0.276
	N	4	4	4	4	4	4
	%-Diff	-9%	-9%	-14%	-10%	-14%	-6%
3/M	Mean	3.02	2.55**	2.31	2.49**	2.55	2.82
	SD	0.118	0.048	0.600	0.147	0.109	0.070
	N	4	4	4	4	4	4
	%-Diff	-8%	-19%	-22%	-19%	-15%	-13%
4/M	Mean	3.05	2.89	2.64	2.73	2.77	3.06
	SD	0.120	0.216	0.390	0.252	0.306	0.235
	N	6	6	6	5	5	5
	%-Diff	-7%	-9%	-11%	-11%	-7%	-6%
	Statistics	A	A	A	A	A	A

* P<=0.05
** P<=0.01
*** P<=0.001
A = ANOVA and Dunnett's

Table
Summary of Clinical Chemistry
Test Item (dosage) 1 2 3 4
[redacted] [redacted]

Group/ Sex	Study Day / Session / Name	GLOBgdL. g/dL 281 / 4 / 48:00	372 / 3 / CC
1/M	Mean	3.11	3.19
	SD	0.277	0.247
	N	6	2
2/M	Mean	2.75	-
	SD	0.275	-
	N	4	-
	%-Diff	-12%	-
3/M	Mean	2.67	-
	SD	0.078	-
	N	4	-
	%-Diff	-14%	-
4/M	Mean	2.92	2.81
	SD	0.374	0.071
	N	5	2
	%-Diff	-6%	-12%
	Statistics	AT	X7

A = ANOVA and Dunnett's
T = Rank-transformed data
X7 = Not analyzed (mean of actual group sizes too small)

Table
Summary of Clinical Chemistry
Test Item (dosage) 1 2 3 4
[redacted] [redacted]

Group/ Sex	Study Day / Session / Name	-21 / 3 / CC	57 / 4 / 48:00	85 / 4 / 48:00	141 / 4 / 48:00	197 / 4 / 48:00	253 / 4 / 48:00
		GLOBgdL. g/dL					
1/F	Mean	3.27	3.24	3.37	3.38	3.33	3.67
	SD	0.288	0.138	0.190	0.175	0.195	0.275
	N	6	6	6	6	6	6
2/F	Mean	3.01	2.82*	2.95	2.78**	2.99	3.21
	SD	0.152	0.193	0.205	0.158	0.095	0.080
	N	4	4	4	4	4	4
	%-Diff	-8%	-13%	-12%	-18%	-10%	-13%
3/F	Mean	2.86	2.76**	2.82*	2.75**	2.87	3.15*
	SD	0.231	0.222	0.310	0.337	0.400	0.277
	N	4	4	4	4	4	4
	%-Diff	-13%	-15%	-16%	-19%	-14%	-14%
4/F	Mean	3.18	2.75**	2.87*	2.75***	2.80*	3.18*
	SD	0.349	0.220	0.337	0.263	0.342	0.355
	N	6	6	6	6	6	6
	%-Diff	-3%	-15%	-15%	-19%	-16%	-13%
	Statistics	A	A	A	A	A	A

* P<=0.05
** P<=0.01
*** P<=0.001
A = ANOVA and Dunnett's

Table
Summary of Clinical Chemistry
Test Item (dosage) 1 2 3 4
[redacted] [redacted]

Group/ Sex	Study Day / Session / Name	GLOBgdL. g/dL 281 / 4 / 48:00	372 / 3 / CC
1/F	Mean	3.19	3.70
	SD	0.176	0.106
	N	6	2
2/F	Mean	2.59**	-
	SD	0.199	-
	N	4	-
	%-Diff	-19%	-
3/F	Mean	2.68**	-
	SD	0.294	-
	N	4	-
	%-Diff	-16%	-
4/F	Mean	2.66**	3.08
	SD	0.247	0.127
	N	6	2
	%-Diff	-17%	-17%
	Statistics	A	X7

* P<=0.05
** P<=0.01
*** P<=0.001
A = ANOVA and Dunnett's
X7 = Not analyzed (mean of actual group sizes too small)

Data in Support of Table 2: Study L, Mic (liver)

Incidence of Microscopic Observations
Terminal Sacrifice 1
Test Item (dosage) 1 2 3 4

[redacted]

Tissue/ Observation	Group/Sex: Number of Animals:	1/M 4	2/M 4	3/M 4	4/M 3	1/F 4	2/F 4	3/F 4	4/F 4
Ileum	Number Examined:	4	4	4	3	4	4	4	4
	Unremarkable:	4	4	4	3	4	4	4	4
Jejunum	Number Examined:	4	4	4	3	4	4	4	4
	Unremarkable:	4	4	4	3	4	4	4	4
Kidney	Number Examined:	4	4	4	3	4	4	4	4
	kable:	2	1	3	1	4	3	3	2
[redacted]		0	0	0	2	0	0	1	1
[redacted]		2	3	1	1	0	1	0	2
Larynx	Number Examined:	4	4	4	3	4	4	4	4
	Unremarkable:	4	4	4	3	4	4	4	4
Liver	Number Examined:	4	4	4	3	4	4	4	4
	Unremarkable:	4	4	2	0	4	4	4	0
Basophilic granules, hepatocytes		0	0	0	1	0	0	0	1
Basophilic granules, Kupffer cells		0	0	2	3	0	0	0	4
Lung	Number Examined:	4	4	4	3	4	4	4	4
	Unremarkable:	4	4	4	3	4	4	3	4
[redacted]		0	0	0	0	0	0	1	0

Data in Support of Table 2: Study L, Mic (kidney)

Incidence of Microscopic Observations
Terminal Sacrifice 1
Test Item (dosage) 1 2 3 4

[redacted]

Tissue/ Observation	Group/Sex:	1/M	2/M	3/M	4/M	1/F	2/F	3/F	4/F
	Number of Animals:	4	4	4	3	4	4	4	4
Ileum	Number Examined:	4	4	4	3	4	4	4	4
	Unremarkable:	4	4	4	3	4	4	4	4
Jejunum	Number Examined:	4	4	4	3	4	4	4	4
	Unremarkable:	4	4	4	3	4	4	4	4
Kidney	Number Examined:	4	4	4	3	4	4	4	4
	Unremarkable:	2	1	3	1	4	3	3	2
Basophilic granules, cortical tubular cells		0	0	0	2	0	0	1	1
Infiltrate, mononuclear cells		2	3	1	1	0	1	0	2
Larynx	Number Examined:	4	4	4	3	4	4	4	4
	Unremarkable:	4	4	4	3	4	4	4	4
Liver	Number Examined:	4	4	4	3	4	4	4	4
[redacted]	le:	4	4	2	0	4	4	4	0
		0	0	0	1	0	0	0	1
		0	0	2	3	0	0	0	4
Lung	Number Examined:	4	4	4	3	4	4	4	4
	Unremarkable:	4	4	4	3	4	4	3	4
[redacted]		0	0	0	0	0	0	1	0

Data in Support of Table 2: Study L, Mic (lymph node)

Incidence of Microscopic Observations
Terminal Sacrifice 1

3 4
[redacted]

Tissue/ Observation	Group/Sex:	1/M	2/M	3/M	4/M	1/F	2/F	3/F	4/F
	Number of Animals:	4	4	4	3	4	4	4	4
Lymph Node, Axillary	Number Examined:	4	4	4	3	4	4	4	4
	Unremarkable:	3	0	0	0	2	0	1	1
Erythrocytes, sinus		1	2	2	3	2	0	0	0
Granular macrophages		0	4	4	3	0	4	3	3
Sinus histiocytosis		0	0	0	0	1	0	0	0
Lymph Node, Mandibular	Number Examined:	4	4	4	3	4	4	4	4
	Unremarkable:	3	3	2	0	3	4	0	1
Erythrocytes, sinus		1	0	0	1	0	0	1	0
Granular macrophages		0	0	2	3	0	0	1	3
Sinus histiocytosis		0	1	0	0	1	0	3	0
Lymph Node, Mesenteric	Number Examined:	4	4	4	3	3	4	4	4
	Unremarkable:	4	1	0	0	1	1	0	0
Granular macrophages		0	3	4	3	0	2	4	4
Sinus histiocytosis		0	0	0	0	2	1	0	0
Mammary Gland	Number Examined:	4	4	4	3	4	4	4	4
	Unremarkable:	4	4	4	3	4	4	4	4

Data in Support of Table 2: Study L, Mic (injection site)

8378066

Incidence of Microscopic Observations
Terminal Sacrifice 1
Test Item (dosage) 1 2 3 4

[redacted]

Tissue/ Observation	Group/Sex: / Number of Animals:	1/M 4	2/M 4	3/M 4	4/M 3	1/F 4	2/F 4	3/F 4	4/F 4
Subcutaneous Injection Site	Number Examined:	4	4	4	3	4	4	4	4
	Unremarkable:	4	3	2	0	4	4	3	3
Hemorrhage		0	0	1	0	0	0	0	0
Infiltrate, mononuclear cells, perivascular		0	1	1	3	0	0	1	1
Testis	Number Examined:	4	4	4	3	0	0	0	0
	Unremarkable:	1	2	1	2	0	0	0	0
[redacted]		0	1	0	0	0	0	0	0
[redacted]		2	0	0	1	0	0	0	0
[redacted]		0	1	3	0	0	0	0	0
[redacted]		1	0	0	0	0	0	0	0
Thymus	Number Examined:	4	4	4	3	4	4	4	4
	Unremarkable:	0	1	2	0	2	0	0	2
[redacted]		4	3	2	3	2	4	4	2
Thyroid	Number Examined:	4	4	4	3	4	4	4	4
	[redacted]kable:	2	2	3	2	3	3	4	2
[redacted]		0	2	1	0	1	0	0	2
[redacted]		2	0	0	1	0	1	0	0

Data in Support of Table 2: Study M, Serum IgG

Summary of Immunoglobulin

[redacted] em [redacted]

IGG. g/L

Group/ Sex	Phase	Predose		Dosing			
	Day	6	50	31	46	52	58
	Session	1	1	1	1	1	1
	Name	Clin Path	Clin Path	Clin Path	Clin Path	Clin Path	Clin Path
1/M	Mean	11.05	10.83	11.24	10.67	11.32	10.93
	SD	1.419	2.181	2.369	1.973	2.295	2.294
	N	3	5	5	5	5	5
2/M	Mean	12.17	11.44	3.75***	3.77***	4.08***	3.94***
	SD	1.685	1.973	0.468	0.429	0.416	0.382
	N	6	6	6	6	6	6
	Statistics	S	S	S	S	S	ST

* P<=0.05
** P<=0.01
*** P<=0.001
S = Two-sample t-test
T = Rank-transformed data

Table
Summary of Immunoglobulin
[redacted] em [redacted]

Group/ Sex	Phase / Day / Session / Name	IGG. g/L Dosing 67 / 1 / Clin Path	73 / 1 / Clin Path	79 / 1 / Clin Path	86 / 1 / Clin Path	94 / 1 / Clin Path	100 / 1 / Clin Path
1/M	Mean	10.72	11.37	10.51	11.87	11.45	11.88
	SD	1.698	2.516	2.200	2.898	1.918	2.210
	N	5	5	5	3	5	5
2/M	Mean	4.21***	4.50***	4.16***	6.58**	4.42***	4.67***
	SD	0.339	0.477	0.368	1.048	0.550	0.762
	N	6	6	6	6	6	6
	Statistics	S	S	S	ST	S	S

* P<=0.05
** P<=0.01
*** P<=0.001
S = Two-sample t-test
T = Rank-transformed data

Table
Summary of Immunoglobulin
em
[redacted] [redacted] [redacted]

Group/ Sex	Phase / Day / Session / Name	IGG. g/L Dosing 106 1 Clin Path	115 1 Clin Path	121 1 Clin Path	130 1 Clin Path	136 1 Clin Path	142 1 Clin Path
1/M	Mean	11.00	11.82	10.72	11.76	11.55	11.01
	SD	1.933	2.077	2.424	2.089	2.282	1.861
	N	5	5	5	5	5	5
2/M	Mean	4.18***	4.33***	4.13***	4.31***	4.16***	4.15***
	SD	0.541	0.540	0.516	0.543	0.616	0.623
	N	6	6	6	6	6	6
	Statistics	S	S	S	S	S	S

* P<=0.05
** P<=0.01
*** P<=0.001
S = Two-sample t-test

Table
Summary of Immunoglobulin

[redacted] em [redacted] [redacted]

		IGG. g/L					
	Phase	Dosing					Recovery
Group/ Sex	Day Session Name	151 1 Clin Path	157 1 Clin Path	163 1 Clin Path	169 1 Clin Path	176 1 Clin Path	8 1 Clin Path
1/M	Mean	12.32	10.60	11.57	11.38	11.83	12.41
	SD	2.219	1.011	1.476	2.037	2.355	2.326
	N	5	4	5	4	5	2
2/M	Mean	4.76***	4.43***	4.42***	4.21**	5.61***	2.69
	SD	0.824	0.537	0.529	0.535	0.928	0.665
	N	6	6	6	6	6	2
	Statistics	S	S	S	ST	S	X7

* P<=0.05
** P<=0.01
*** P<=0.001
S = Two-sample t-test
T = Rank-transformed data

X7 = Not analyzed (mean of actual group sizes too small)

Table
Summary of Immunoglobulin

[redacted] em [redacted] [redacted]

Group/ Sex	Phase / Day / Session / Name	IGG. g/L Recovery 22 / 1 / Clin Path	36 / 1 / Clin Path	53 / 1 / Clin Path
1/M	Mean	13.48	12.41	14.01
	SD	2.312	3.253	3.486
	N	2	2	2
2/M	Mean	8.03	11.39	13.40
	SD	1.110	0.651	2.058
	N	2	2	2
	Statistics	X7	X7	X7

X7 = Not analyzed (mean of actual group sizes too small)

Table
Summary of Immunoglobulin
Test Item (dosage)

[redacted]

Group/Sex	Phase / Day / Session / Name	Predose 50 1 Clin Path	Predose 94 1 Clin Path	Dosing 3 1 Clin Path	Dosing 10 1 Clin Path	Dosing 16 1 Clin Path	Dosing 25 1 Clin Path
		IGG. g/L					
1/F	Mean	9.80	9.63	-	-	-	-
	SD	1.420	1.993	-	-	-	-
	N	5	5	-	-	-	-
2/F	Mean	10.52	10.71	8.87	3.54	5.05	4.67
	SD	1.314	1.592	0.389	0.028	2.984	2.008
	N	8	8	2	2	2	2
	Statistics	S	S	X	X	X	X

S = Two-sample t-test
X = No analysis performed

Table
Summary of Immunoglobulin

[redacted] em [redacted]

Group/ Sex	Phase / Day / Session / Name	IGG. g/L Dosing 31 1 Clin Path	37 1 Clin Path	46 1 Clin Path	52 1 Clin Path	58 1 Clin Path	67 1 Clin Path
1/F	Mean	10.08	-	9.53	9.62	9.86	9.27
	SD	1.936	-	2.171	2.244	1.771	1.168
	N	5	-	4	5	5	5
2/F	Mean	5.42*	3.20	3.86**	4.16***	3.89***	3.21***
	SD	4.223	0.120	2.122	1.680	1.761	0.707
	N	8	2	5	8	8	7
	Statistics	S	X	S	S	S	S

* P<=0.05
** P<=0.01
*** P<=0.001
S = Two-sample t-test
X = No analysis performed

Table
Summary of Immunoglobulin

[redacted] em [redacted]

Group/ Sex	Phase / Day / Session / Name	IGG. g/L Dosing 73 1 Clin Path	79 1 Clin Path	86 1 Clin Path	94 1 Clin Path	100 1 Clin Path	106 1 Clin Path
1/F	Mean	9.71	8.53	10.78	10.75	10.40	9.28
	SD	1.779	1.508	1.819	1.537	1.482	1.828
	N	5	5	3	5	5	5
2/F	Mean	3.52***	3.45***	5.38***	3.72***	3.96***	3.60***
	SD	0.920	0.982	0.884	0.791	0.954	0.559
	N	7	7	6	7	7	7
	Statistics	S	S	S	S	S	ST

* P<=0.05
** P<=0.01
*** P<=0.001
S = Two-sample t-test
T = Rank-transformed data

Table
Summary of Immunoglobulin

[redacted] em [redacted] [redacted]

Group/ Sex		Phase / Day / Session / Name	IGG. g/L Dosing 115 / 1 / Clin Path	121 / 1 / Clin Path	130 / 1 / Clin Path	136 / 1 / Clin Path	142 / 1 / Clin Path	151 / 1 / Clin Path
1/F	Mean		10.38	10.14	10.27	9.82	9.27	11.22
	SD		1.704	1.777	2.022	2.066	1.650	2.003
	N		5	5	5	5	5	5
2/F	Mean		3.57***	3.35***	3.43***	3.63***	3.26***	4.19***
	SD		0.759	0.689	0.843	0.440	0.500	0.699
	N		7	7	7	7	7	7
	Statistics		S	S	S	S	S	S

* P<=0.05
** P<=0.01
*** P<=0.001
S = Two-sample t-test

Table
Summary of Immunoglobulin

[redacted] em [redacted] [redacted]

Group/ Sex	Phase	Dosing				Recovery	
		IGG. g/L					
	Day	157	163	169	176	8	22
	Session	1	1	1	1	1	1
	Name	Clin Path	Clin Path	Clin Path	Clin Path	Clin Path	Clin Path
1/F	Mean	11.02	11.16	10.21	10.12	10.07	11.03
	SD	1.673	1.934	1.631	2.041	3.302	2.652
	N	5	5	5	5	2	2
2/F	Mean	3.81***	3.44***	3.29***	4.30***	1.64	7.48
	SD	0.434	0.683	0.467	0.841	0.170	0.148
	N	7	7	7	7	2	2
	Statistics	ST	ST	ST	S	X7	X7

* P<=0.05
** P<=0.01
*** P<=0.001
S = Two-sample t-test
T = Rank-transformed data

X7 = Not analyzed (mean of actual group sizes too small)

Table
Summary of Immunoglobulin

[redacted] em [redacted] [redacted]

Group/ Sex	Phase / Day / Session / Name	IGG. g/L Recovery	
	Day	36	53
	Session	1	1
	Name	Clin Path	Clin Path
1/F	Mean	10.30	12.05
	SD	2.638	3.571
	N	2	2
2/F	Mean	10.07	13.20
	SD	0.007	0.049
	N	2	2
	Statistics	X7	X7

X7 = Not analyzed (mean of actual group sizes too small)

Data in Support of Table 2: Study M, Serum albumin

Table
Summary of Clinical
Chemistry Test Item (dosage)

[redacted] [redacted] [redacted]

		ALB. g/L					
	Phase	Predose		Dosing			
Group/ Sex	Day	6	50	3	10	16	25
	Session	1	1	1	1	1	1
	Name	Clin Path	Clin Path	Clin Path	Clin Path	Clin Path	Clin Path
1/M	Mean	51.47	44.28	41.80	44.02	44.82	46.79
	SD	7.817	4.663	2.086	6.026	5.965	7.160
	N	3	5	4	5	5	5
2/M	Mean	43.19	43.12	42.09	40.45	39.16	37.78**
	SD	5.205	5.730	6.434	6.664	2.310	1.879
	N	6	6	6	6	6	6
	Statistics	S	S	S	S	S	ST

* P<=0.05
** P<=0.01
*** P<=0.001
S = Two-sample t-test
T = Rank-transformed data

Table
Summary of Clinical Chemistry

[redacted] em [redacted] [redacted]

Group/ Sex	Phase / Day / Session / Name	ALB. g/L Dosing 31 / 1 / Clin Path	37 / 1 / Clin Path	46 / 1 / Clin Path	52 / 1 / Clin Path	58 / 1 / Clin Path	67 / 1 / Clin Path
1/M	Mean	46.57	46.26	43.87	43.66	41.83	41.26
	SD	6.415	5.313	5.053	5.334	1.664	1.392
	N	5	5	5	5	5	5
2/M	Mean	38.18**	38.37*	37.05*	36.19*	35.90***	35.87**
	SD	2.445	2.601	2.064	1.999	1.570	2.306
	N	6	6	6	6	6	6
	Statistics	ST	S	S	S	S	S

* P<=0.05
** P<=0.01
*** P<=0.001
S = Two-sample t-test
T = Rank-transformed data

Table
Summary of Clinical Chemistry

[redacted] em [redacted] [redacted]

Group/ Sex	Phase	ALB. g/L Dosing					
	Day	73	79	86	94	100	106
	Session	1	1	1	1	1	1
	Name	Clin Path	Clin Path	Clin Path	Clin Path	Clin Path	Clin Path
1/M	Mean	41.47	39.71	40.84	41.50	41.05	40.76
	SD	2.593	2.269	2.595	2.672	1.499	1.263
	N	5	5	3	5	5	5
2/M	Mean	36.39**	34.07**	34.93**	35.71**	34.72***	34.61***
	SD	2.066	1.927	1.503	2.992	2.011	1.974
	N	6	6	6	6	6	6
	Statistics	S	S	S	S	S	S

* P<=0.05
** P<=0.01
*** P<=0.001
S = Two-sample t-test

Table
Summary of Clinical Chemistry

[redacted]em[redacted] [redacted]

Group/ Sex	Phase / Day / Session / Name	ALB. g/L Dosing 115 1 Clin Path	121 1 Clin Path	130 1 Clin Path	136 1 Clin Path	142 1 Clin Path	151 1 Clin Path
1/M	Mean	41.30	41.91	40.75	42.17	39.64	40.11
	SD	2.265	1.522	1.716	1.531	1.919	2.192
	N	5	5	5	5	5	5
2/M	Mean	34.20***	35.44***	35.29***	36.69***	34.63***	34.66***
	SD	1.346	1.934	1.342	2.066	1.301	1.301
	N	6	6	6	6	6	6
	Statistics	S	S	S	S	S	S

* P<=0.05
** P<=0.01
*** P<=0.001
S = Two-sample t-test

Table
Summary of Clinical Chemistry

[redacted] em [redacted] [redacted]

Group/ Sex	Phase / Day / Session / Name	Dosing 157 1 Clin Path	Dosing 163 1 Clin Path	Dosing 169 1 Clin Path	Dosing 176 1 Clin Path	Recovery 8 1 Clin Path	Recovery 22 1 Clin Path
		ALB. g/L					
1/M	Mean	40.61	41.41	41.48	46.97	39.09	40.69
	SD	2.317	2.502	2.576	5.946	2.150	2.234
	N	4	5	4	5	2	2
2/M	Mean	35.95**	35.47***	35.82**	36.77***	34.41	39.19
	SD	1.670	1.300	1.364	2.004	0.396	1.315
	N	6	6	6	6	2	2
	Statistics	S	S	S	ST	X7	X7

* P<=0.05
** P<=0.01
*** P<=0.001
S = Two-sample t-test
T = Rank-transformed data

X7 = Not analyzed (mean of actual group sizes too small)

Table
Summary of Clinical Chemistry

[redacted] em [redacted] [redacted]

Group/ Sex	Phase / Day / Session / Name	ALB. g/L Recovery 36 / 1 / Clin Path	ALB. g/L Recovery 53 / 1 / Clin Path
1/M	Mean	40.08	41.07
	SD	0.007	0.453
	N	2	2
2/M	Mean	38.71	39.13
	SD	1.450	0.156
	N	2	2
	Statistics	X7	X7

X7 = Not analyzed (mean of actual group sizes too small)

Table
Summary of Clinical
Chemistry Test Item (dosage)

[redacted]

Group/ Sex	Phase / Day / Session / Name	Predose: 50 / 1 / Clin Path	Predose: 94 / 1 / Clin Path	Dosing: 3 / 1 / Clin Path	Dosing: 10 / 1 / Clin Path	Dosing: 16 / 1 / Clin Path	Dosing: 25 / 1 / Clin Path
		ALB. g/L					
1/F	Mean	40.77	39.98	39.77	41.32	41.51	41.87
	SD	0.818	0.752	1.495	0.339	2.021	1.181
	N	5	5	4	5	5	5
2/F	Mean	41.05	40.54	38.63	37.45***	35.73***	36.50**
	SD	1.257	2.439	2.157	1.491	2.012	2.559
	N	8	8	8	8	8	8
	Statistics	S	S	S	S	S	S

* P<=0.05
** P<=0.01
*** P<=0.001
S = Two-sample t-test

Table
Summary of Clinical Chemistry

[redacted]em[redacted] [redacted]

Group/ Sex	Phase / Day / Session / Name	ALB. g/L Dosing 31 1 Clin Path	37 1 Clin Path	46 1 Clin Path	52 1 Clin Path	58 1 Clin Path	67 1 Clin Path
1/F	Mean	41.56	41.51	40.33	38.94	40.42	40.05
	SD	1.387	1.788	2.078	1.945	1.414	1.020
	N	5	5	4	5	5	5
2/F	Mean	36.03***	34.77***	35.42*	36.03*	36.69**	36.51*
	SD	2.381	2.149	2.634	2.412	2.140	2.359
	N	8	7	5	8	8	7
	Statistics	S	S	S	S	S	S

* P<=0.05
** P<=0.01
*** P<=0.001
S = Two-sample t-test

Table
Summary of Clinical Chemistry

[redacted] em [redacted] [redacted]

Group/ Sex	Phase / Day / Session / Name	ALB. g/L Dosing 73 1 Clin Path	79 1 Clin Path	86 1 Clin Path	94 1 Clin Path	100 1 Clin Path	106 1 Clin Path
1/F	Mean	40.55	37.43	36.59	39.45	39.38	38.91
	SD	2.013	1.262	1.216	1.688	1.986	0.984
	N	5	5	3	5	5	5
2/F	Mean	36.78**	34.79	34.93	35.47*	34.98*	35.39*
	SD	2.033	3.291	2.726	2.496	3.987	2.951
	N	7	7	6	7	7	7
	Statistics	S	S	S	S	S	ST

* P<=0.05
** P<=0.01
*** P<=0.001
S = Two-sample t-test
T = Rank-transformed data

Table
Summary of Clinical Chemistry

[redacted] em [redacted] [redacted]

Group/ Sex	Phase / Day / Session / Name	ALB. g/L Dosing 115 / 1 / Clin Path	121 / 1 / Clin Path	130 / 1 / Clin Path	136 / 1 / Clin Path	142 / 1 / Clin Path	151 / 1 / Clin Path
1/F	Mean	38.88	40.90	40.27	40.82	38.36	38.41
	SD	1.456	1.928	1.725	2.289	0.700	2.061
	N	5	5	5	5	5	5
2/F	Mean	35.72*	36.79*	36.31*	37.40	36.00	35.52
	SD	2.483	2.803	3.023	2.899	2.976	2.638
	N	7	7	7	7	7	7
	Statistics	S	S	S	S	S	S

* P<=0.05
** P<=0.01
*** P<=0.001
S = Two-sample t-test

Table
Summary of Clinical Chemistry

[redacted] em [redacted]

ALB. g/L

Group/ Sex	Phase / Day / Session / Name	Dosing 157 1 Clin Path	Dosing 163 1 Clin Path	Dosing 169 1 Clin Path	Dosing 176 1 Clin Path	Recovery 8 1 Clin Path	Recovery 22 1 Clin Path
1/F	Mean	39.14	38.87	38.95	40.52	37.94	38.74
	SD	1.851	0.380	2.773	1.631	2.454	1.223
	N	5	5	5	5	2	2
2/F	Mean	37.01	35.57*	35.36*	36.42*	33.66	36.94
	SD	2.890	2.966	2.151	2.732	1.039	2.418
	N	7	7	7	7	2	2
	Statistics	S	ST	S	S	X7	X7

* P<=0.05
** P<=0.01
*** P<=0.001
S = Two-sample t-test
T = Rank-transformed data

X7 = Not analyzed (mean of actual group sizes too small)

Table
Summary of Clinical Chemistry

[redacted] em [redacted] [redacted]

Group/ Sex	Phase<br>Day<br>Session<br>Name	ALB. g/L Recovery<br>36<br>1<br>Clin Path	 53<br>1<br>Clin Path
1/F	Mean	37.98	39.68
	SD	0.544	0.672
	N	2	2
2/F	Mean	37.57	38.47
	SD	3.323	4.214
	N	2	2
	Statistics	X7	X7

X7 = Not analyzed (mean of actual group sizes too small)

Data in Support of Table 2: Study M, Serum cholesterole

Table
Summary of Clinical
Chemistry Test Item (dosage)

[redacted]

Group/ Sex	Phase / Day / Session / Name	Predose 6 1 Clin Path	Predose 50 1 Clin Path	Dosing 31 1 Clin Path	Dosing 86 1 Clin Path	Dosing 136 1 Clin Path	Dosing 176 1 Clin Path
		CHOL. mmol/L					
1/M	Mean	2.99	3.02	2.95	2.70	3.02	3.31
	SD	0.469	0.450	0.566	0.767	0.665	0.665
	N	3	5	5	3	5	5
2/M	Mean	3.20	2.82	3.85**	3.75**	4.02*	4.34*
	SD	0.378	0.339	0.175	0.177	0.372	0.494
	N	6	6	6	6	6	6
	Statistics	S	S	S	ST	S	S

* P<=0.05
** P<=0.01
*** P<=0.001
S = Two-sample t-test
T = Rank-transformed data

Table
Summary of Clinical Chemistry

em
[redacted] [redacted] [redacted]

Group/ Sex	Phase / Day / Session / Name	CHOL. mmol/L Recovery 53 1 Clin Path
1/M	Mean	3.36
	SD	0.120
	N	2
2/M	Mean	2.82
	SD	0.240
	N	2
	Statistics	X7

X7 = Not analyzed (mean of actual group sizes too small)

Table
Summary of Clinical
Chemistry Test Item (dosage)

[redacted] [redacted] [redacted]

Group/ Sex	Phase / Day / Session / Name	Predose 50 1 Clin Path	Predose 94 1 Clin Path	Dosing 31 1 Clin Path	Dosing 58 1 Clin Path	Dosing 86 1 Clin Path	Dosing 136 1 Clin Path
		CHOL. mmol/L					
1/F	Mean	4.23	4.21	4.55	-	4.17	4.44
	SD	0.637	1.102	1.213	-	1.170	1.243
	N	5	5	5	-	3	5
2/F	Mean	3.61	3.47	4.44	6.64	3.78	4.33
	SD	0.781	0.732	1.233	-	0.762	0.799
	N	8	8	8	1	6	7
	Statistics	S	S	S	X	S	S

S = Two-sample t-test
X = No analysis performed

Table
Summary of Clinical
Chemistry Test Item (dosage)

[redacted] [redacted] [redacted]

		CHOL. mmol/L	
	Phase	Dosing	Recovery
Group/ Sex	Day	176	53
	Session	1	1
	Name	Clin Path	Clin Path
1/F	Mean	4.83	4.87
	SD	1.201	0.403
	N	5	2
2/F	Mean	4.50	2.51
	SD	0.761	0.474
	N	7	2
	Statistics	S	X7

S = Two-sample t-test
X7 = Not analyzed (mean of actual group sizes too small)

Data in Support of Table 2: Study M, TDAR

Individual KLH IGG
Test Item (dosage) 1 2
--- --
[redacted] ---- [redacted] ---- [redacted]

Group/ Sex	Animal Number	Phase Day	KLHG Dosing 77	84	91	98	105
1/M	17435M		100	2332	11523	9154	10748
	17451M		100	8664	52300	51974	51929
	17452M		100	6802	31712	39580	36177
	19397M		100	2343	11973	26007	36356
	19416M		100	2168	10619	11167	10950
	Mean		100	4462	23625	27576	29232
	SD		0.0	3058.7	18296.4	18374.2	17958.5
	N		5	5	5	5	5

Individual KLH IGG
Test Item (dosage)

1 2

[redacted] --- [redacted] -- [redacted]

Group/ Sex	Animal Number	Phase / Day	KLHG Dosing 140	147	154	161	Recovery 35
1/M	17435M		7792	46541	45793	37421	-
	17451M		26983	27291	18718	62448	-
	17452M		10407	54466	50517	48957	-
	19397M		55760	46529	47308	43699	58388
	19416M		9745	61365	61900	60562	24620
	Mean		22137	47238	44847	50617	41504
	SD		20315.8	12754.3	15910.9	10766.0	23877.6
	N		5	5	5	5	2

Individual KLH IGG
Test Item (dosage)

1 2

[redacted] --- [redacted] -- [redacted]

Group/ Sex	Animal Number	Phase / Day	KLHG Recovery 42	49	56
1/M	19397M		269649	266699	248201
	19416M		>312500#	207921	167901
	Mean		>291075	237310	208051
	SD		30300.2	41562.3	56780.7
	N		2	2	2

# = Value shown used in descriptive statistics

Individual KLH IGG
Test Item (dosage)

[redacted] --- [redacted] 1 -- [redacted] 2

Group/ Sex	Animal Number	Phase Day	KLHG Dosing 77	84	91	98	105
2/M	18785M		346	10464	10204	7246	2429
	18787M		100	11477	8654	7991	6733
	18882M		100	10133	10512	7085	7186
	18897M		100	9496	6825	5769	5273
	19007M		100	4961	11574	9194	6779
	19012M		100	11291	33046	8349	8347
	Mean		141	9637	13469	7606	6125
	SD		100.4	2405.7	9732.2	1183.0	2061.4
	N		6	6	6	6	6

Individual KLH IGG
Test Item (dosage)

1 2

[redacted] --- [redacted] -- [redacted]

Group/ Sex	Animal Number	Phase / Day	KLHG Dosing 140	147	154	161	Recovery 35
2/M	18785M		1743	23365	11086	9363	-
	18787M		2465	31642	26578	17739	-
	18882M		1809	14779	31948	12374	-
	18897M		2197	29782	43437	11891	-
	19007M		2458	60718	38451	15678	19314
	19012M		2377	62097	35060	11509	7613
	Mean		2175	37064	31093	13092	13464
	SD		324.4	19763.0	11345.9	3054.5	8273.9
	N		6	6	6	6	2

Individual KLH IGG
Test Item (dosage)

[redacted] [redacted] 1 2 [redacted]

Group/ Sex	Animal Number	Phase Day	KLHG Recovery 42	49	56
2/M	19007M		215941	202884	229972
	19012M		177443	58867	47785
	Mean		196692	130876	138879
	SD		27222.2	101835.4	128825.7
	N		2	2	2

Individual KLH IGG
[redacted]st Item (dosage) 1 2
[redacted]

Group/ Sex	Animal Number	Phase Day	KLHG Dosing 77	84	91	98	105
1/F	18361F		100	12203	30904	18563	19314
	18585F		100	27979	53743	55516	59566
	19234F		100	9104	32009	22947	25331
	19248F		100	7111	12030	12406	26437
	19257F		100	11075	47696	44134	45135
	Mean		100	13494	35276	30713	35157
	SD		0.0	8326.9	16320.6	18292.0	16725.3
	N		5	5	5	5	5

Individual KLH IGG
Test Item (dosage)

1 2

[redacted] --- [redacted] -- [redacted]

Group/ Sex	Animal Number	Phase / Day	KLHG Dosing 140	147	154	161	Recovery 35
1/F	18361F		12248	22632	21108	12102	-
	18585F		50431	44072	28467	15590	-
	19234F		11944	36095	25576	24137	-
	19248F		8764	50318	52217	45435	9929
	19257F		14416	37681	30904	12172	32244
	Mean		19561	38160	31654	21887	21087
	SD		17374.6	10348.7	12059.4	14047.7	15779.1
	N		5	5	5	5	2

Individual KLH IGG
Test Item (dosage)

[redacted] --- [redacted] 1 -- 2 [redacted]

Group/ Sex	Animal Number	Phase / Day	KLHG Recovery		
			42	49	56
1/F	19248F		62095	56947	42202
	19257F		211812	217857	188206
	Mean		136954	137402	115204
	SD		105865.9	113780.6	103240.4
	N		2	2	2

Individual KLH IGG
Test Item (dosage)

[redacted] --- [redacted] [1] -- [redacted] [2]

Group/ Sex	Animal Number	Phase / Day	KLHG Dosing 77	84	91	98	105
2/F	17644F		100	11820	26343	11424	10754
	18219F		100	9872	11500	11605	9407
	18231F		100	9336	11398	10427	8503
	18624F		100	23444	29881	11791	9852
	18627F		100	7940	11999	10077	6183
	19056F		100	8100	11546	9785	7535
	19221F		100	6324	9973	5961	3302
	Mean		100	10977	16091	10153	7934
	SD		0.0	5762.0	8298.6	2008.1	2543.1
	N		7	7	7	7	7

Individual KLH IGG
Test Item (dosage)

[redacted] --- [redacted] 1 -- 2 [redacted]

Group/ Sex	Animal Number	Phase Day	KLHG Dosing 140	147	154	161	Recovery 35
2/F	17644F		7102	35534	54248	30340	-
	18219F		10717	50135	57059	21860	-
	18231F		6168	30922	47214	27308	12421
	18624F		2568	45611	51533	23260	12357
	18627F		4842	18077	50924	18487	-
	19056F		1956	56158	59082	44781	-
	19221F		1991	59694	29969	12207	-
	Mean		5049	42304	50004	25463	12389
	SD		3232.9	14882.5	9683.0	10348.9	45.3
	N		7	7	7	7	2

Individual KLH IGG
Test Item (dosage)

[redacted] --- [redacted] 1 -- 2 [redacted]

Group/ Sex	Animal Number	Phase / Day	KLHG Recovery 42	49	56
2/F	18231F		215914	203210	172931
	18624F		247294	238319	231060
	Mean		231604	220765	201996
	SD		22189.0	24825.8	41103.4
	N		2	2	2

Data in Support of Table 2: Study O, Mic (testis)

TEST ITEM : [redacted]
TEST SYSTEM : M. CYNOMOLGUS, 4-Week, Gavage
SPONSOR : [redacted]

[redacted]

SUMMARY INCIDENCE OF GRADINGS BY ORGAN/GROUP/SEX
STATUS AT NECROPSY: K0
ALL GRADED FINDINGS IN ALL ANIMALS ON STUDY

DOSE GROUP:	1		2		3		4	
SEX :	M	F	M	F	M	F	M	F
NO.ANIMALS:	3	3	3	3	3	3	3	3
SKIN/SUBCUTIS CONT'D.	3	3	3	3	3	3	3	3
– [redacted]								
GRADE 1 :	1	–	–	–	–	–	–	–
GRADE 2 :	1	–	–	–	–	–	–	–
TOTAL AFFECTED :	2	–	–	–	–	–	–	–
MEAN GRADE/TISS.AFF.:	1.5	–	–	–	–	–	–	–
SPLEEN :	3	3	3	3	3	3	3	3
– [redacted]								
GRADE 1 :	1	2	1	3	–	–	–	1
GRADE 2 :	2	–	1	–	2	2	1	2
GRADE 3 :	–	1	1	–	–	–	2	–
GRADE 4 :	–	–	–	–	1	–	–	–
TOTAL AFFECTED :	3	3	3	3	3	2	3	3
MEAN GRADE/TISS.AFF.:	1.7	1.7	2.0	1.0	2.7	2.0	2.7	1.7
TESTES :	3	–	3	–	3	–	3	–
– Deg./Atrophy Tubular								
GRADE 1 :	–	–	1	–	–	–	–	–
TOTAL AFFECTED :	–	–	1	–	–	–	–	–
MEAN GRADE/TISS.AFF.:	–	–	1.0	–	–	–	–	–
– Dilation, Tubular								
GRADE 1 :	1	–	–	–	–	–	–	–
GRADE 3 :	–	–	–	–	–	–	1	–
GRADE 4 :	–	–	–	–	–	–	1	–
TOTAL AFFECTED :	1	–	–	–	–	–	2	–
MEAN GRADE/TISS.AFF.:	1.0	–	–	–	–	–	3.5	–

TEST ITEM : [redacted]
TEST SYSTEM : M. CYNOMOLGUS, 4–Week, Gavage
SPONSOR : [redacted]

[redacted]

SUMMARY INCIDENCE OF GRADINGS BY ORGAN/GROUP/SEX
STATUS AT NECROPSY: K0
ALL GRADED FINDINGS IN ALL ANIMALS ON STUDY

DOSE GROUP:	1		2		3		4	
SEX :	M	F	M	F	M	F	M	F
NO.ANIMALS:	3	3	3	3	3	3	3	3
TESTES CONT'D.	3	–	3	–	3	–	3	–
– Giant Cell, Multinuc								
GRADE 1 :	–	–	–	–	1	–	–	–
GRADE 2 :	–	–	–	–	–	–	2	–
GRADE 3 :	–	–	–	–	1	–	1	–
TOTAL AFFECTED :	–	–	–	–	2	–	3	–
MEAN GRADE/TISS.AFF.:	–	–	–	–	2.0	–	2.3	–
THYMUS :	3	3	3	3	3	3	3	3
– [redacted]								
GRADE 1 :	–	–	1	1	–	1	–	1
GRADE 2 :	–	2	1	2	2	1	–	–
GRADE 3 :	2	–	1	–	–	1	2	1
GRADE 4 :	1	–	–	–	–	–	1	–
TOTAL AFFECTED :	3	2	3	3	2	3	3	2
MEAN GRADE/TISS.AFF.:	3.3	2.0	2.0	1.7	2.0	2.0	3.3	2.0
THYROID GLAND :	3	3	3	3	3	3	3	3
– [redacted]								
GRADE 1 :	–	1	–	–	–	–	–	–
TOTAL AFFECTED :	–	1	–	–	–	–	–	–
MEAN GRADE/TISS.AFF.:	–	1.0	–	–	–	–	–	–
– [redacted]								
GRADE 1 :	–	–	–	–	–	1	–	–
GRADE 4 :	–	–	–	–	–	–	–	1
TOTAL AFFECTED :	–	–	–	–	–	1	–	1
MEAN GRADE/TISS.AFF.:	–	–	–	–	–	1.0	–	4.0

Data in Support of Table 2: Study O, Mac (testis)

Data in Support of Table 2: Study O, Blood RBC

Table
Summary of Hematology
ge)

[redacted] [redacted]

Group/ Sex	Phase / Day	Predose 3	Dosing 3	Dosing 24	Recovery 58
		RBC. 10E12/L			
1/M	Mean	7.08	6.72	6.64	7.34
	SD	0.249	0.432	0.274	0.325
	N	5	5	5	2
2/M	Mean	7.07	6.83	6.91	-
	SD	0.364	0.101	0.164	-
	N	3	3	3	-
3/M	Mean	7.39	7.16	5.65	-
	SD	0.240	0.333	1.675	-
	N	3	3	3	-
4/M	Mean	7.35	6.62	6.45	7.03
	SD	0.200	0.445	0.381	0.240
	N	5	5	5	2
	Statistics	A	A	AT	X7

A = ANOVA and Dunnett's
T = Rank-transformed data
X7 = Not analyzed (mean of actual group sizes too small)

Table
Summary of Hematology
[redacted] ge) [redacted]

Group/ Sex	Phase / Day	Predose 3	Dosing 3	Dosing 24	Recovery 58
		RBC. 10E12/L			
1/F	Mean	6.53	6.16	6.60	6.85
	SD	0.383	0.589	0.508	0.106
	N	5	5	5	2
2/F	Mean	6.31	6.07	6.12	-
	SD	0.569	0.793	0.610	-
	N	3	3	3	-
3/F	Mean	6.15	5.90	6.06	-
	SD	0.243	0.101	0.044	-
	N	3	3	3	-
4/F	Mean	6.51	6.20	5.95	7.10
	SD	0.489	0.498	0.513	0.007
	N	5	5	5	2
	Statistics	A	A	A	X7

A = ANOVA and Dunnett's
X7 = Not analyzed (mean of actual group sizes too small)

Data in Support of Table 2: Study P, Serum ALT

Table
Summary of Clinical Chemistry
[redacted] ge) [redacted]

Group/ Sex	Phase / Day / Session / Name	Predose: 1 / 1 / Clin Path	Predose: 8 / 1 / Clin Path	Dosing: 44 / 5 / 48 Hour	Dosing: 65 / 5 / 48 Hour	Dosing: 93 / 5 / 48 Hour	Dosing: 135 / 5 / 48 Hour
		ALT. U/L					
1/M	Mean	54.02	39.53	37.24	50.10	40.83	40.67
	SD	20.180	12.770	16.099	12.241	12.264	12.987
	N	6	6	6	6	6	6
2/M	Mean	63.89	50.11	36.28	50.90	58.52	52.56
	SD	11.100	5.218	5.206	3.730	10.395	8.669
	N	4	4	4	4	4	4
	%-Diff	18%	27%	-3%	2%	43%	29%
3/M	Mean	47.46	43.83	41.12	58.85	55.34	61.95
	SD	10.545	7.454	17.045	25.401	23.460	35.572
	N	4	4	4	4	4	4
	%-Diff	-12%	11%	10%	17%	36%	52%
4/M	Mean	46.56	36.97	72.18	58.36	50.13	61.16
	SD	18.833	9.407	62.113	23.327	17.385	31.624
	N	7	7	7	7	7	7
	%-Diff	-14%	-6%	94%	16%	23%	50%
	Statistics	A	A	A	A	A	A

A = ANOVA and Dunnett's

Table
Summary of Clinical Chemistry
[redacted] ge)

Group/Sex	Phase / Day / Session / Name	ALT. U/L Dosing 184 / 5 / 48 Hour	233 / 5 / 48 Hour	275 / 5 / 48 Hour	Recovery 84 / 1 / Clin Path	182 / 1 / Clin Path
1/M	Mean	46.93	44.41	60.98	39.15	47.15
	SD	15.729	11.504	25.330	7.135	4.801
	N	5	6	6	2	2
2/M	Mean	71.98	67.56	81.63	-	-
	SD	18.562	15.222	25.698	-	-
	N	4	4	4	-	-
	%-Diff	53%	52%	34%	-	-
3/M	Mean	48.53	65.63	73.56	-	-
	SD	4.744	23.425	39.876	-	-
	N	3	4	4	-	-
	%-Diff	3%	48%	21%	-	-
4/M	Mean	55.42	68.18	86.41	64.16	53.91
	SD	10.081	33.598	21.180	8.726	15.542
	N	6	7	7	2	2
	%-Diff	18%	54%	42%	64%	14%
	Statistics	A	A	A	X7	X7

A = ANOVA and Dunnett's
X7 = Not analyzed (mean of actual group sizes too small)

Table
Summary of Clinical Chemistry
[redacted]

Group/ Sex	Phase / Day / Session / Name	Predose: 1 / 1 / Clin Path	Predose: 8 / 1 / Clin Path	Dosing: 44 / 5 / 48 Hour	Dosing: 65 / 5 / 48 Hour	Dosing: 93 / 5 / 48 Hour	Dosing: 135 / 5 / 48 Hour
		ALT. U/L					
1/F	Mean	58.44	49.48	44.72	47.93	46.54	44.24
	SD	13.403	4.776	9.510	10.084	8.908	9.540
	N	6	6	6	6	6	6
2/F	Mean	34.28*	36.66*	37.60	34.31	45.24	32.03
	SD	5.669	6.711	12.221	7.617	18.757	11.490
	N	4	4	4	3	4	4
	%-Diff	-41%	-26%	-16%	-28%	-3%	-28%
3/F	Mean	40.20	35.56*	38.02	44.43	42.48	43.93
	SD	12.158	6.430	9.940	4.179	6.819	13.258
	N	4	4	4	4	4	4
	%-Diff	-31%	-28%	-15%	-7%	-9%	-1%
4/F	Mean	50.09	52.59	48.38	122.70	80.65	79.15*
	SD	14.779	13.810	12.005	168.520	40.845	36.026
	N	7	7	7	7	7	7
	%-Diff	-14%	6%	8%	156%	73%	79%
	Statistics	A	AT	A	A	AT	AT

* P<=0.05
** P<=0.01
*** P<=0.001
A = ANOVA and Dunnett's
T = Rank-transformed data

Table
Summary of Clinical Chemistry
[redacted] ge) [redacted]

Group/Sex	Phase / Day / Session / Name	Dosing 184 5 48 Hour	Dosing 233 5 48 Hour	Dosing 275 5 48 Hour	Recovery 84 1 Clin Path	Recovery 182 1 Clin Path
		ALT. U/L				
1/F	Mean	66.63	45.04	75.13	30.79	41.36
	SD	22.672	9.956	22.300	0.163	1.916
	N	6	6	6	2	2
2/F	Mean	55.04	35.44	47.78*	-	-
	SD	28.449	13.163	10.264	-	-
	N	4	4	4	-	-
	%-Diff	-17%	-21%	-36%	-	-
3/F	Mean	69.35	46.55	84.54	-	-
	SD	16.466	5.860	8.881	-	-
	N	4	4	4	-	-
	%-Diff	4%	3%	13%	-	-
4/F	Mean	86.57	96.85**	71.82	55.87	42.49
	SD	34.404	48.590	10.328	26.227	19.495
	N	7	7	6	2	2
	%-Diff	30%	115%	-4%	81%	3%
	Statistics	A	AT	A	X7	X7

* P<=0.05
** P<=0.01
*** P<=0.001
A = ANOVA and Dunnett's
T = Rank-transformed data

X7 = Not analyzed (mean of actual group sizes too small)

Data in Support of Table 2: Study P, Serum AP

Table
Summary of Clinical Chemistry
[redacted]

ALP. U/L

Group/ Sex	Phase / Day / Session / Name	Predose: 1 / 1 / Clin Path	Predose: 8 / 1 / Clin Path	Dosing: 44 / 5 / 48 Hour	Dosing: 65 / 5 / 48 Hour	Dosing: 93 / 5 / 48 Hour	Dosing: 135 / 5 / 48 Hour
1/M	Mean	589.91	502.85	572.23	635.16	607.11	659.57
	SD	232.245	165.414	127.199	120.802	93.111	176.951
	N	6	6	6	6	6	6
2/M	Mean	700.22	616.71	717.14	841.15	929.36	1054.23
	SD	185.255	242.733	333.701	425.535	530.193	719.403
	N	4	4	4	4	4	4
	%-Diff	19%	23%	25%	32%	53%	60%
3/M	Mean	814.65	770.08	789.77	879.12	859.08	943.21
	SD	75.810	53.392	194.299	262.988	234.289	250.003
	N	4	4	4	4	4	4
	%-Diff	38%	53%	38%	38%	42%	43%
4/M	Mean	590.14	531.46	581.65	613.87	604.45	684.55
	SD	212.447	196.199	229.485	235.997	219.191	249.483
	N	7	7	7	7	7	7
	%-Diff	0%	6%	2%	-3%	0%	4%
	Statistics	A	A	A	A	AT	AT

A = ANOVA and Dunnett's
T = Rank-transformed data

Table
Summary of Clinical Chemistry
[redacted] ge) [redacted]

		ALP. U/L				
	Phase	Dosing			Recovery	
Group/ Sex	Day	184	233	275	84	182
	Session	5	5	5	1	1
	Name	48 Hour	48 Hour	48 Hour	Clin Path	Clin Path
1/M	Mean	731.52	686.17	583.94	758.34	760.61
	SD	336.213	257.581	147.272	169.904	157.034
	N	5	6	6	2	2
2/M	Mean	1012.42	979.92	825.00	-	-
	SD	641.405	584.228	520.033	-	-
	N	4	4	4	-	-
	%-Diff	38%	43%	41%	-	-
3/M	Mean	1203.78	953.34	755.85	-	-
	SD	351.942	245.677	143.032	-	-
	N	3	4	4	-	-
	%-Diff	65%	39%	29%	-	-
4/M	Mean	914.60	825.74	664.82	664.99	553.70
	SD	378.344	347.157	237.384	206.694	143.889
	N	6	7	7	2	2
	%-Diff	25%	20%	14%	-12%	-27%
	Statistics	A	A	AT	X7	X7

A = ANOVA and Dunnett's
T = Rank-transformed data
X7 = Not analyzed (mean of actual group sizes too small)

Table
Summary of Clinical Chemistry
[redacted] ge) [redacted]

Group/ Sex	Phase / Day / Session / Name	Predose<br>1<br>1<br>Clin Path	Predose<br>8<br>1<br>Clin Path	Dosing<br>44<br>5<br>48 Hour	Dosing<br>65<br>5<br>48 Hour	Dosing<br>93<br>5<br>48 Hour	Dosing<br>135<br>5<br>48 Hour
		ALP. U/L					
1/F	Mean	478.78	445.33	412.81	424.55	363.51	366.46
	SD	121.037	112.699	126.894	146.671	91.524	116.399
	N	6	6	6	6	6	6
2/F	Mean	519.04	474.05	467.68	409.17	456.54	430.22
	SD	159.536	110.602	96.304	41.782	70.085	82.610
	N	4	4	4	3	4	4
	%-Diff	8%	6%	13%	-4%	26%	17%
3/F	Mean	526.69	446.56	441.28	463.24	470.59	394.14
	SD	92.729	67.848	72.198	51.820	78.246	31.520
	N	4	4	4	4	4	4
	%-Diff	10%	0%	7%	9%	29%	8%
4/F	Mean	428.21	390.24	408.43	413.33	375.50	391.34
	SD	76.753	69.255	91.858	92.960	83.831	76.816
	N	7	7	7	7	7	7
	%-Diff	-11%	-12%	-1%	-3%	3%	7%
	Statistics	A	A	A	A	A	A

A = ANOVA and Dunnett's

Table
Summary of Clinical Chemistry
[redacted]ge)

Group/ Sex	Phase / Day / Session / Name	Dosing 184 5 48 Hour	Dosing 233 5 48 Hour	Dosing 275 5 48 Hour	Recovery 84 1 Clin Path	Recovery 182 1 Clin Path
		ALP. U/L				
1/F	Mean	345.89	320.02	287.34	222.61	213.41
	SD	106.649	78.663	83.148	68.059	76.749
	N	6	6	6	2	2
2/F	Mean	419.70	395.47	345.33	-	-
	SD	90.880	103.453	76.546	-	-
	N	4	4	4	-	-
	%-Diff	21%	24%	20%	-	-
3/F	Mean	401.80	350.91	301.68	-	-
	SD	43.784	56.379	35.724	-	-
	N	4	4	4	-	-
	%-Diff	16%	10%	5%	-	-
4/F	Mean	377.18	343.14	386.23	301.28	259.75
	SD	101.638	90.780	113.464	163.752	77.831
	N	7	7	7	2	2
	%-Diff	9%	7%	34%	35%	22%
	Statistics	A	A	A	X7	X7

A = ANOVA and Dunnett's
X7 = Not analyzed (mean of actual group sizes too small)

Data in Support of Table 2: Study P, Serum GLDH

Table
Summary of Clinical Chemistry
[redacted]ge)[redacted]

		GLDH. U/L					
	Phase	Predose		Dosing			
Group/ Sex	Day	1	8	44	65	93	135
	Session	1	1	5	5	5	5
	Name	Clin Path	Clin Path	48 Hour	48 Hour	48 Hour	48 Hour
1/M	Mean	17.24	14.50	14.31	19.92	13.42	14.84
	SD	3.157	5.064	4.815	5.067	3.116	3.333
	N	6	6	6	6	6	6
2/M	Mean	17.45	20.15	19.19	19.52	26.67	23.57*
	SD	3.496	5.321	4.509	5.386	4.916	6.105
	N	4	4	4	4	4	4
	%-Diff	1%	39%	34%	-2%	99%	59%
3/M	Mean	19.87	17.17	23.00	26.76	26.76	36.32*
	SD	5.433	3.106	10.026	13.956	14.169	28.750
	N	4	4	4	4	4	4
	%-Diff	15%	18%	61%	34%	99%	145%
4/M	Mean	14.71	13.24	26.59	25.05	20.40	26.51*
	SD	6.340	5.539	16.275	13.734	9.067	14.705
	N	7	7	7	7	7	7
	%-Diff	-15%	-9%	86%	26%	52%	79%
	Statistics	A	A	AT	A	A	AT

* P<=0.05
** P<=0.01
*** P<=0.001
A = ANOVA and Dunnett's
T = Rank-transformed data

Table
Summary of Clinical Chemistry
[redacted] ge) [redacted]

GLDH

. U/L

Group/ Sex	Phase / Day / Session Name	Dosing 184 5 48 Hour	Dosing 233 5 48 Hour	Dosing 275 5 48 Hour	Recovery 84 1 Clin Path	Recovery 182 1 Clin Path
1/M	Mean	13.72	17.34	15.16	14.69	14.95
	SD	4.617	3.900	6.401	5.006	3.253
	N	5	6	6	2	2
2/M	Mean	24.59	26.93	26.98	-	-
	SD	10.744	8.377	14.224	-	-
	N	4	4	4	-	-
	%-Diff	79%	55%	78%	-	-
3/M	Mean	18.60	35.12	22.00	-	-
	SD	3.096	17.076	9.162	-	-
	N	3	4	4	-	-
	%-Diff	36%	103%	45%	-	-
4/M	Mean	18.67	31.89	23.18	25.63	21.08
	SD	2.616	13.392	3.740	2.475	8.167
	N	6	7	7	2	2
	%-Diff	36%	84%	53%	74%	41%
	Statistics	A	A	A	X7	X7

A = ANOVA and Dunnett's
X7 = Not analyzed (mean of actual group sizes too small)

Table
Summary of Clinical Chemistry
ge)
[redacted]

Group/Sex	Phase / Day / Session / Name	Predose: 1 / 1 / Clin Path	Predose: 8 / 1 / Clin Path	Dosing: 44 / 5 / 48 Hour	Dosing: 65 / 5 / 48 Hour	Dosing: 93 / 5 / 48 Hour	Dosing: 135 / 5 / 48 Hour
		GLDH. U/L					
1/F	Mean	21.61	16.77	16.00	14.77	14.00	15.41
	SD	9.560	5.154	5.438	3.875	3.172	4.628
	N	6	6	6	6	6	6
2/F	Mean	11.63	12.46	13.55	12.76	15.31	15.77
	SD	1.315	2.556	3.565	1.200	2.838	3.494
	N	4	4	4	3	4	4
	%-Diff	-46%	-26%	-15%	-14%	9%	2%
3/F	Mean	12.61	10.21	12.65	17.89	15.22	23.33
	SD	3.168	2.765	4.044	6.336	6.881	12.134
	N	4	4	4	4	4	4
	%-Diff	-42%	-39%	-21%	21%	9%	51%
4/F	Mean	16.22	15.91	20.66	64.74	39.14	40.98**
	SD	3.844	6.032	9.325	94.015	32.668	20.411
	N	7	7	7	7	7	7
	%-Diff	-25%	-5%	29%	338%	180%	166%
	Statistics	A	A	A	A	A	A

* P<=0.05
** P<=0.01
*** P<=0.001
A = ANOVA and Dunnett's

Table
Summary of Clinical Chemistry
[redacted] ge) [redacted]

		GLDH. U/L				
	Phase	Dosing			Recovery	
Group/ Sex	Day	184	233	275	84	182
	Session	5	5	5	1	1
	Name	48 Hour	48 Hour	48 Hour	Clin Path	Clin Path
1/F	Mean	22.86	15.20	19.64	12.78	15.97
	SD	7.604	3.555	7.207	0.290	0.693
	N	6	6	6	2	2
2/F	Mean	21.67	17.47	18.15	-	-
	SD	8.446	5.352	5.713	-	-
	N	4	4	4	-	-
	%-Diff	-5%	15%	-8%	-	-
3/F	Mean	29.49	21.13	23.11	-	-
	SD	21.298	5.760	5.535	-	-
	N	4	4	4	-	-
	%-Diff	29%	39%	18%	-	-
4/F	Mean	37.84	55.24***	29.93*	29.04	15.00
	SD	17.845	31.701	7.432	1.881	7.679
	N	7	7	6	2	2
	%-Diff	66%	264%	52%	127%	-6%
	Statistics	A	AT	A	X7	X7

* P<=0.05
** P<=0.01
*** P<=0.001
A = ANOVA and Dunnett's
T = Rank-transformed data

X7 = Not analyzed (mean of actual group sizes too small)

Data in Support of Table 2: Study P, Mic (liver)

Test Item (dosage) 1 2 3 4

[redacted]

Tissue/ Observation	Group/Sex: Number of Animals:	1/M 4	2/M 4	3/M 4	4/M 5	1/F 4	2/F 4	3/F 4	4/F 5
Ad[redacted]	mber Examined:	4	4	4	5	4	4	4	5
[redacted]		0	1	2	2	2	0	0	1
		0	0	0	0	0	0	1	0
Liver	Number Examined:	4	4	4	5	4	4	4	5
Infiltration of inflammatory cells		3	1	2	1	3	1	1	3
Basophilic granules, Kupffer cells		0	0	1	5	0	0	4	5
Cytoplasmic granules, hepatocytes		0	0	0	4	0	0	0	2
Th[redacted]	Number Examined:	4	4	4	5	4	4	4	5
		0	1	0	0	0	0	1	1
		0	0	2	1	1	0	2	1
Lymph Node, M[redacted]	:	4	4	4	5	4	4	4	5
		2	0	4	5	0	0	4	5
Th[redacted]	mber Examined:	4	4	4	5	4	4	4	5
		0	0	0	1	0	0	0	0
		0	0	0	1	0	0	0	0
Pa oid	Number Examined:	2	2	4	5	3	4	4	5
[redacted]		0	1	1	0	0	0	0	0
He[redacted]	:	4	4	4	5	4	4	4	5
		2	0	1	1	0	0	0	2

Data in Support of Table 2: Study P, Mic (kidney)

Incidence of Microscopic Observations - Terminal Sacrifice

Test Item (dosage) 1 2 3 4

[redacted]

Tissue/ Observation	Group/Sex: Number of Animals:	1/M 4	2/M 4	3/M 4	4/M 5	1/F 4	2/F 4	3/F 4	4/F 5
Lu[redacted]	:	4	4	4	5	4	4	4	5
[redacted]		0	1	0	0	0	0	0	0
		0	0	0	0	0	1	0	0
		0	0	0	1	0	0	0	0
Pancreas	Number Examined:	4	4	4	5	4	4	4	5
[redacted]		0	0	0	2	0	0	1	0
		0	0	0	0	0	1	0	0
Kidney	Number Examined:	4	4	4	5	4	4	4	5
Infiltration of inflammatory cells		2	2	3	2	4	3	1	3
Basophilic, tubule		0	0	1	0	0	1	0	0
Cytoplasmic granules, tubular epithelial cells		0	0	1	5	0	0	1	4
Ov[redacted]	Number Examined:	0	0	0	0	4	4	4	5
		0	0	0	0	4	4	4	5
		0	0	0	0	2	1	3	3
Tes[redacted]	Number Examined:	4	4	4	5	0	0	0	0
		2	1	2	2	0	0	0	0
		0	1	1	3	0	0	0	0
		2	2	1	0	0	0	0	0
Ep[redacted]	Number Examined:	4	4	4	5	0	0	0	0
		2	1	1	2	0	0	0	0
		0	1	0	3	0	0	0	0

Data in Support of Table 2: Study P, Mic (lymph node)

Incidence of Microscopic Observations - Terminal Sacrifice

Test Item (dosage) 1 2 3 4

[redacted]

Tissue/ Observation	Group/Sex: Number of Animals:	1/M 4	2/M 4	3/M 4	4/M 5	1/F 4	2/F 4	3/F 4	4/F 5
Br[redacted]	r Examined:	4	4	4	5	4	4	4	5
[redacted]r		1	0	1	2	0	0	0	0
[redacted]		0	0	0	1	0	0	0	0
[redacted]		0	0	1	0	0	0	0	1
To[redacted]	:	4	4	4	5	4	4	4	5
[redacted]		0	0	0	1	0	0	0	0
Sk[redacted]	:	4	4	4	5	4	4	4	5
[redacted]		1	0	1	1	0	0	0	0
Mammary Gland	Number Examined:	4	4	4	5	4	4	4	5
S[redacted]		3	4	3	5	0	0	0	0
D[redacted]		0	2	0	1	0	0	0	0
Lymph Node, Axillary	Number Examined:	4	4	4	5	4	4	4	5
Vacuolated macrophages, sinuses		0	4	4	5	0	4	3	5
Erythrocytes, sinus		0	0	0	0	1	0	0	0
Cellularity, general, increased		0	1	2	0	0	1	0	1
Subcutaneous I[redacted]	:	4	4	4	5	4	4	4	5
[redacted]		1	3	4	5	0	3	3	5
[redacted]		0	0	0	0	0	0	0	1
La[redacted]	Examined:	4	4	4	5	4	4	4	5
[redacted]		0	0	0	1	0	0	0	1

Data in Support of Table 2: Study P, Mic (injection site)

Incidence of Microscopic Observations - Terminal Sacrifice

Test Item (dosage) 1 2 3 4

[redacted]

Tissue/ Observation	Group/Sex: Number of Animals:	1/M 4	2/M 4	3/M 4	4/M 5	1/F 4	2/F 4	3/F 4	4/F 5
Br[redacted]	Examined:	4	4	4	5	4	4	4	5
[redacted]		1	0	1	2	0	0	0	0
		0	0	0	1	0	0	0	0
[redacted]		0	0	1	0	0	0	0	1
To[redacted]	:	4	4	4	5	4	4	4	5
[redacted]		0	0	0	1	0	0	0	0
Sk[redacted]	:	4	4	4	5	4	4	4	5
[redacted]		1	0	1	1	0	0	0	0
Mammary Gland	Number Examined:	4	4	4	5	4	4	4	5
		3	4	3	5	0	0	0	0
[redacted]		0	2	0	1	0	0	0	0
Lymph Node, A[redacted]	ned:	4	4	4	5	4	4	4	5
[redacted]		0	4	4	5	0	4	3	5
		0	0	0	0	1	0	0	0
[redacted]		0	1	2	0	0	1	0	1
Subcutaneous Injection Site	Number Examined:	4	4	4	5	4	4	4	5
Infiltration of inflammatory cells		1	3	4	5	0	3	3	5
Ulcer		0	0	0	0	0	0	0	1
La[redacted]	Examined:	4	4	4	5	4	4	4	5
[redacted]		0	0	0	1	0	0	0	1

Data in Support of Table 2: Study P, Organ weight (liver)

Summary of Organ Weights and Organ Weight Ratios - Terminal Sacrifice

Test Item (dosage) 1 2 3 4

[redacted] [redacted] [redacted] [redacted] [redacted]

Group/ Sex		Terminal Body Weight (g)	[redacted]			Liver		
			Unadjusted (g)	Body Weight (%)	Brain Weight (%)	Unadjusted (g)	Body Weight (%)	Brain Weight (%)
1/M	Mean	3890	14.442	0.3859	21.0176	63.765	1.6634	93.0499
	SD	1037.2	1.2489	0.08033	2.02941	12.8225	0.19251	21.06096
	N	4	4	4	4	4	4	4
2/M	Mean	3940	14.537	0.3705	20.6231	65.084	1.6781	92.2669
	SD	704.1	3.0445	0.05231	5.10297	6.5372	0.22943	13.59647
	N	4	4	4	4	4	4	4
	%-Diff	1%	1%	-4%	-2%	2%	1%	-1%
3/M	Mean	3843	16.446	0.4340	23.9512	75.725	1.9584	109.1552
	SD	659.2	2.1441	0.06496	2.37870	16.9477	0.11108	14.26964
	N	4	4	4	4	4	4	4
	%-Diff	-1%	14%	12%	14%	19%	18%	17%
4/M	Mean	3326	14.037	0.4245	20.5121	71.102	2.1431**	103.7611
	SD	516.7	1.7566	0.03556	2.28388	10.9139	0.18709	13.52899
	N	5	5	5	5	5	5	5
	%-Diff	-14%	-3%	10%	-2%	12%	29%	12%
	Statistics	A	A	A	A	A	A	A

* P<=0.05
** P<=0.01
*** P<=0.001
A = ANOVA and Dunnett's

Summary of Organ Weights and Organ Weight Ratios - Terminal Sacrifice

Test Item [redacted] (dosage) 1 [redacted] 2 [redacted] 3 [redacted] 4 [redacted]

Group/ Sex		Terminal Body Weight (g)	[redacted] Unadjusted (g)	[redacted] Body Weight (%)	[redacted] Brain Weight (%)	Liver Unadjusted (g)	Liver Body Weight (%)	Liver Brain Weight (%)
1/F	Mean	3586	13.093	0.3698	20.6321	67.398	1.8672	106.0722
	SD	586.0	0.9317	0.04093	1.77428	16.8471	0.24068	26.94819
	N	4	4	4	4	4	4	4
2/F	Mean	3184	13.025	0.4074	19.0360	60.314	1.8976	88.2094
	SD	257.8	2.1641	0.04079	2.96929	5.1326	0.13866	7.00838
	N	4	4	4	4	4	4	4
	%-Diff	-11%	-1%	10%	-8%	-11%	2%	-17%
3/F	Mean	2594***	11.135	0.4289	18.0858	52.382	2.0175	84.9751
	SD	116.1	1.4368	0.04782	3.03151	4.3562	0.10153	10.33099
	N	4	4	4	4	4	4	4
	%-Diff	-28%	-15%	16%	-12%	-22%	8%	-20%
4/F	Mean	2750***	11.114*	0.4048	17.0327	61.490	2.2349*	94.0633
	SD	162.1	0.6947	0.02634	0.96822	5.6799	0.13423	5.50931
	N	5	5	5	5	5	5	5
	%-Diff	-23%	-15%	9%	-17%	-9%	20%	-11%
	Statistics	AT	AT	A	A	A	A	A

* P<=0.05
** P<=0.01
*** P<=0.001
A = ANOVA and Dunnett's
T = Rank-transformed data

Data in Support of Table 2: Study P, Soft feces

Test Item (dosage) 1 2 3 4

[redacted]

Group/ Sex	Animal Number	Observation	Phase	Day(s)
1/M	19893M	Excretion		
		feces: normal (1.observation)	PRED	1-15
			DSNG	1-21,23-275
		Qualitative food consumption		
		food consumption: normal (1.observation)	DSNG	1-85,87-183,185-267,269-274
		Excretion		
		feces: soft feces (1.observation)	DSNG	22
		Qualitative food consumption		
		fasting overnight	DSNG	86,184,268,275
1/M	19899M	Excretion		
		feces: normal (1.observation)	PRED	1-15
			DSNG	1-21,23-275
		Qualitative food consumption		
		food consumption: normal (1.observation)	DSNG	1-85,87-183,185-267,269-274
		Excretion		
		feces: soft feces (1.observation)	DSNG	22
		Qualitative food consumption		
		fasting overnight	DSNG	86,184,268,275

Table
Individual Clinical Observations
Test Item (dosage) 1 2 3 4

[redacted]

Group/ Sex	Animal Number	Observation	Phase	Day(s)
1/M	19917M	Excretion		
		feces: normal (1.observation)	PRED	1-15
			DSNG	1-21,23-275
		Qualitative food consumption		
		food consumption: normal (1.observation)	DSNG	1-85,87-183,185-267,269-274
		Excretion		
		feces: soft feces (1.observation)	DSNG	22
		Qualitative food consumption		
		fasting overnight	DSNG	86,184,268,275
1/M	70237M	Excretion		
		feces: normal (1.observation)	PRED	1-15
			DSNG	1-21,23-275
		Qualitative food consumption		
		food consumption: normal (1.observation)	DSNG	1-85,87-183,185-267,269-274
		Excretion		
		feces: soft feces (1.observation)	DSNG	22
		Qualitative food consumption		
		fasting overnight	DSNG	86,184,268,275

Table
Individual Clinical Observations
Test Item (dosage) 1 2 3 4

[redacted]

Group/ Sex	Animal Number	Observation	Phase	Day(s)
1/M	70251M	Excretion		
		feces: normal (1.observation)	PRED	1-15
			DSNG	1-21,23-274
		Qualitative food consumption		
		food consumption: normal (1.observation)	DSNG	1-85,87-183,185-267,269-274
		Excretion		
		feces: soft feces (1.observation)	DSNG	22
		Qualitative food consumption		
		fasting overnight	DSNG	86,184,268
		Excretion		
		feces: normal (1.observation)	RECO	2-182
		Qualitative food consumption		
		food consumption: normal (1.observation)	RECO	2-181
		fasting overnight	RECO	182

Table
Individual Clinical Observations
Test Item (dosage) 1 2 3 4

[redacted]

Group/ Sex	Animal Number	Observation	Phase	Day(s)
1/M	70253M	Excretion		
		feces: normal (1.observation)	PRED	1-15
			DSNG	1-21,23-274
		Qualitative food consumption		
		food consumption: normal (1.observation)	DSNG	1-85,87-183,185-267,269-274
		Excretion		
		feces: soft feces (1.observation)	DSNG	22
		Qualitative food consumption		
		fasting overnight	DSNG	86,184,268
		Excretion		
		feces: normal (1.observation)	RECO	2-182
		Qualitative food consumption		
		food consumption: normal (1.observation)	RECO	2-181
		fasting overnight	RECO	182

Table
Individual Clinical Observations
Test Item (dosage) 1 2 3 4

[redacted]

Group/ Sex	Animal Number	Observation	Phase	Day(s)
2/M	19704M	Excretion		
		feces: normal (1.observation)	PRED	1-15
			DSNG	1,3-275
		Qualitative food consumption		
		food consumption: normal (1.observation)	DSNG	1-85,87-183,185-267,269-274
		Excretion		
		feces: liquid feces (1.observation)	DSNG	2
		Qualitative food consumption		
		fasting overnight	DSNG	86,184,268,275
2/M	19918M	Excretion		
		feces: normal (1.observation)	PRED	1-15
			DSNG	1-275
		Qualitative food consumption		
		food consumption: normal (1.observation)	DSNG	1-85,87-183,185-267,269-274
		fasting overnight	DSNG	86,184,268,275

Table
Individual Clinical Observations
Test Item (dosage) 1 2 3 4

[redacted]

Group/ Sex	Animal Number	Observation	Phase	Day(s)
2/M	70093M	Excretion		
		feces: normal (1.observation)	PRED	1-7,9,10
		feces: liquid feces (1.observation)	PRED	8,11,12,15
		feces: soft feces (1.observation)	PRED	13,14
		feces: normal (1.observation)	DSNG	1-275
		Qualitative food consumption		
		food consumption: normal (1.observation)	DSNG	1-85,87-183,185-267,269-274
		fasting overnight	DSNG	86,184,268,275
2/M	70113M	Excretion		
		feces: normal (1.observation)	PRED	1-7,9,10
		feces: liquid feces (1.observation)	PRED	8,11,12,15
		feces: soft feces (1.observation)	PRED	13,14
		feces: normal (1.observation)	DSNG	1-275
		Qualitative food consumption		
		food consumption: normal (1.observation)	DSNG	1-85,87-183,185-267,269-274
		fasting overnight	DSNG	86,184,268,275

Table
Individual Clinical Observations
Test Item (dosage) 1 2 3 4

[redacted]

Group/ Sex	Animal Number	Observation	Phase	Day(s)
3/M	70084M	Excretion		
		feces: normal (1.observation)	PRED	1-7,9,10
		feces: liquid feces (1.observation)	PRED	8,11,12
		feces: soft feces (1.observation)	PRED	13-15
		feces: normal (1.observation)	DSNG	1,2,8-10,15,20,21,31-38, 40-275
		Qualitative food consumption		
		food consumption: normal (1.observation)	DSNG	1-85,87-183,185-267,269-274
		Excretion		
		feces: liquid feces (1.observation)	DSNG	3-7,11-14,17-19,22-24
		feces: soft feces (1.observation)	DSNG	16,25-30,39
		Qualitative food consumption		
		fasting overnight	DSNG	86,184,268,275

Table
Individual Clinical Observations
Test Item (dosage) 1 2 3 4

[redacted]

Group/ Sex	Animal Number	Observation	Phase	Day(s)
3/M	70087M	Excretion		
		feces: normal (1.observation)	PRED	1-7,9,10
		feces: liquid feces (1.observation)	PRED	8,11,12
		feces: soft feces (1.observation)	PRED	13-15
		feces: normal (1.observation)	DSNG	1,2,8-10,14,15,18-21,24-69, 73-275
		Qualitative food consumption		
		food consumption: normal (1.observation)	DSNG	1-85,87-183,185-267,269-274
		Excretion		
		feces: liquid feces (1.observation)	DSNG	3-7,11-13,17,22,23
		feces: soft feces (1.observation)	DSNG	16,70-72
		Qualitative food consumption		
		fasting overnight	DSNG	86,184,268,275

Table
Individual Clinical Observations
Test Item (dosage) 1 2 3 4

[redacted]

Group/ Sex	Animal Number	Observation	Phase	Day(s)
3/M	70102M	Excretion		
		feces: normal (1.observation)	PRED	1-7,9,10
		feces: liquid feces (1.observation)	PRED	8,11,12,15
		feces: soft feces (1.observation)	PRED	13,14
		feces: normal (1.observation)	DSNG	1,2,8-10,14,15,18-21,24-275
		Qualitative food consumption		
		food consumption: normal (1.observation)	DSNG	1-85,87-183,185-267,269-274
		Excretion		
		feces: liquid feces (1.observation)	DSNG	3-7,11-13,17,22,23
		feces: soft feces (1.observation)	DSNG	16
		Qualitative food consumption		
		fasting overnight	DSNG	86,184,268,275

Table
Individual Clinical Observations
Test Item (dosage) 1 2 3 4

[redacted]

Group/ Sex	Animal Number	Observation	Phase	Day(s)
3/M	70107M	Excretion		
		feces: normal (1.observation)	PRED	1-7,9,10
		feces: liquid feces (1.observation)	PRED	8,11,12,15
		feces: soft feces (1.observation)	PRED	13,14
		feces: normal (1.observation)	DSNG	1,2,8-10,14,15,18-21,24-37, 40-275
		Qualitative food consumption		
		food consumption: normal (1.observation)	DSNG	1-85,87-183,185-267,269-274
		Excretion		
		feces: liquid feces (1.observation)	DSNG	3-7,11-13,17,22,23
		feces: soft feces (1.observation)	DSNG	16,38,39
		Qualitative food consumption		
		fasting overnight	DSNG	86,184,268,275
4/M	19780M	Excretion		
		feces: normal (1.observation)	PRED	1-15
			DSNG	1-275
		Qualitative food consumption		
		food consumption: normal (1.observation)	DSNG	1-85,87-183,185-267,269-274
		fasting overnight	DSNG	86,184,268,275

Table
Individual Clinical Observations
Test Item (dosage) 1 2 3 4

[redacted]

Group/ Sex	Animal Number	Observation	Phase	Day(s)
4/M	19786M	Excretion		
		feces: normal (1.observation)	PRED	1-15
			DSNG	1-275
		Qualitative food consumption		
		food consumption: normal		
		(1.observation)	DSNG	1-85,87-183,185-267,269-274
		fasting overnight	DSNG	86,184,268,275
4/M	19829M	Excretion		
		feces: normal (1.observation)	PRED	1-15
			DSNG	1-275
		Qualitative food consumption		
		food consumption: normal		
		(1.observation)	DSNG	1-85,87-183,185-267,269-274
		fasting overnight	DSNG	86,184,268,275

Table
Individual Clinical Observations
Test Item (dosage) 1 2 3 4

[redacted]

Group/ Sex	Animal Number	Observation	Phase	Day(s)
4/M	70246M	Excretion		
		feces: normal (1.observation)	PRED	1-15
			DSNG	1-274
		Qualitative food consumption		
		food consumption: normal (1.observation)	DSNG	1-85,87-183,185-267,269-274
		fasting overnight	DSNG	86,184,268
		Excretion		
		feces: normal (1.observation)	RECO	2-182
		Qualitative food consumption		
		food consumption: normal (1.observation)	RECO	2-181
		fasting overnight	RECO	182
4/M	70252M	Excretion		
		feces: normal (1.observation)	PRED	1-15
			DSNG	1-275
		Qualitative food consumption		
		food consumption: normal (1.observation)	DSNG	1-85,87-183,185-267,269-274
		fasting overnight	DSNG	86,184,268,275

Table
Individual Clinical Observations
Test Item (dosage) 1 2 3 4

[redacted]

Group/ Sex	Animal Number	Observation	Phase	Day(s)
4/M	70255M	Excretion		
		feces: normal (1.observation)	PRED	1-15
			DSNG	1-274
		Qualitative food consumption		
		food consumption: normal		
		(1.observation)	DSNG	1-85,87-183,185-267,269-274
		fasting overnight	DSNG	86,184,268
		Excretion		
		feces: normal (1.observation)	RECO	2-182
		Qualitative food consumption		
		food consumption: normal		
		(1.observation)	RECO	2-181
		fasting overnight	RECO	182
4/M	70269M	Excretion		
		feces: normal (1.observation)	PRED	1-15
			DSNG	1-275
		Qualitative food consumption		
		food consumption: normal		
		(1.observation)	DSNG	1-85,87-183,185-267,269-274
		fasting overnight	DSNG	86,184,268,275

Table
Individual Clinical Observations
Test Item (dosage) 1 2 3 4

[redacted]

Group/ Sex	Animal Number	Observation	Phase	Day(s)
1/F	18022F	Excretion		
		feces: normal (1.observation)	PRED	1-6,8,9,12,13,15
		feces: liquid feces (1.observation)	PRED	7,10,14
		feces: soft feces (1.observation)	PRED	11
		feces: normal (1.observation)	DSNG	1-26,28-34,36,38-55,62-275
		Qualitative food consumption		
		food consumption: normal (1.observation)	DSNG	1-85,87-183,185-267,269-274
		Excretion		
		feces: liquid feces (1.observation)	DSNG	27,35,37,56,57
		feces: soft feces (1.observation)	DSNG	58-61
		Qualitative food consumption		
		fasting overnight	DSNG	86,184,268,275

Table
Individual Clinical Observations
Test Item (dosage) 1 2 3 4

[redacted]

Group/ Sex	Animal Number	Observation	Phase	Day(s)
1/F	19730F	Excretion		
		feces: normal (1.observation)	PRED	1-13,15
		feces: liquid feces (1.observation)	PRED	14
		feces: normal (1.observation)	DSNG	1-26,28-34,36,38-275
		Qualitative food consumption		
		food consumption: normal (1.observation)	DSNG	1-85,87-183,185-267,269-274
		Excretion		
		feces: liquid feces (1.observation)	DSNG	27,35,37
		Qualitative food consumption		
		fasting overnight	DSNG	86,184,268,275

Table
Individual Clinical Observations
Test Item (dosage) 1 2 3 4

[redacted]

Group/ Sex	Animal Number	Observation	Phase	Day(s)
1/F	19967F	Excretion		
		feces: normal (1.observation)	PRED	1,2,6,8,9,12,13,15
		feces: liquid feces (1.observation)	PRED	3,4,7,10,14
		feces: soft feces (1.observation)	PRED	5,11
		feces: normal (1.observation)	DSNG	1-26,28-34,36,38-275
		Qualitative food consumption		
		food consumption: normal (1.observation)	DSNG	1-85,87-183,185-267,269-274
		Excretion		
		feces: liquid feces (1.observation)	DSNG	27,35,37
		Qualitative food consumption		
		fasting overnight	DSNG	86,184,268,275

Table
Individual Clinical Observations
Test Item (dosage) 1 2 3 4

[redacted]

Group/ Sex	Animal Number	Observation	Phase	Day(s)
1/F	19971F	Excretion		
		feces: normal (1.observation)	PRED	1-13,15
		feces: liquid feces (1.observation)	PRED	14
		feces: normal (1.observation)	DSNG	1-26,28-34,36,38-275
		Qualitative food consumption		
		food consumption: normal (1.observation)	DSNG	1-85,87-183,185-267,269-274
		Excretion		
		feces: liquid feces (1.observation)	DSNG	27,35,37
		Qualitative food consumption		
		fasting overnight	DSNG	86,184,268,275

Table
Individual Clinical Observations
Test Item (dosage) 1 2 3 4

[redacted]

Group/ Sex	Animal Number	Observation	Phase	Day(s)
1/F	70181F	Excretion		
		feces: normal (1.observation)	PRED	1-13,15
		feces: liquid feces (1.observation)	PRED	14
		feces: normal (1.observation)	DSNG	1-26,28-34,36,39-274
		Qualitative food consumption		
		food consumption: normal (1.observation)	DSNG	1-85,87-183,185-267,269-274
		Excretion		
		feces: liquid feces (1.observation)	DSNG	27,35,37,38
		Qualitative food consumption		
		fasting overnight	DSNG	86,184,268
		Excretion		
		feces: normal (1.observation)	RECO	1-182
		Qualitative food consumption		
		food consumption: normal (1.observation)	RECO	1-181
		fasting overnight	RECO	182

Table
Individual Clinical Observations
Test Item (dosage) 1 2 3 4

[redacted]

Group/ Sex	Animal Number	Observation	Phase	Day(s)
1/F	70194F	Excretion		
		feces: normal (1.observation)	PRED	1-13,15
		feces: liquid feces (1.observation)	PRED	14
		feces: normal (1.observation)	DSNG	1-26,28-36,39-274
		Qualitative food consumption		
		food consumption: normal (1.observation)	DSNG	1-85,87-183,185-267,269-274
		Excretion		
		feces: liquid feces (1.observation)	DSNG	27,37,38
		Qualitative food consumption		
		fasting overnight	DSNG	86,184,268
		Excretion		
		feces: normal (1.observation)	RECO	1-182
		Qualitative food consumption		
		food consumption: normal (1.observation)	RECO	1-181
		fasting overnight	RECO	182

Table
Individual Clinical Observations
Test Item (dosage) 1 2 3 4

[redacted]

Group/ Sex	Animal Number	Observation	Phase	Day(s)
2/F	19847F	Excretion		
		feces: normal (1.observation)	PRED	1-6,8,9,12-15
		feces: liquid feces (1.observation)	PRED	7,10
		feces: soft feces (1.observation)	PRED	11
		feces: normal (1.observation)	DSNG	1-275
		Qualitative food consumption		
		food consumption: normal (1.observation)	DSNG	1-85,87-183,185-267,269-274
		fasting overnight	DSNG	86,184,268,275
2/F	19857F	Excretion		
		feces: normal (1.observation)	PRED	1,2,6,8,9,12-15
		feces: liquid feces (1.observation)	PRED	3,4,7,10
		feces: soft feces (1.observation)	PRED	5,11
		feces: normal (1.observation)	DSNG	1-275
		Qualitative food consumption		
		food consumption: normal (1.observation)	DSNG	1-85,87-183,185-267,269-274
		fasting overnight	DSNG	86,184,268,275

Table
Individual Clinical Observations
Test Item (dosage) 1 2 3 4

[redacted]

Group/ Sex	Animal Number	Observation	Phase	Day(s)
2/F	19931F	Excretion		
		feces: normal (1.observation)	PRED	1-6,8,9,12-15
		feces: liquid feces (1.observation)	PRED	7,10
		feces: soft feces (1.observation)	PRED	11
		feces: normal (1.observation)	DSNG	1-275
		Qualitative food consumption		
		food consumption: normal (1.observation)	DSNG	1-85,87-183,185-267,269-274
		fasting overnight	DSNG	86,184,268,275
2/F	19965F	Excretion		
		feces: normal (1.observation)	PRED	1-6,8,9,12-15
		feces: liquid feces (1.observation)	PRED	7,10
		feces: soft feces (1.observation)	PRED	11
		feces: normal (1.observation)	DSNG	1-275
		Qualitative food consumption		
		food consumption: normal (1.observation)	DSNG	1-85,87-183,185-267,269-274
		fasting overnight	DSNG	86,184,268,275

Table
Individual Clinical Observations
Test Item (dosage) 1 2 3 4

[redacted]

Group/ Sex	Animal Number	Observation	Phase	Day(s)
3/F	70178F	Excretion		
		feces: normal (1.observation)	PRED	1-15
			DSNG	1-275
		Qualitative food consumption		
		food consumption: normal (1.observation)	DSNG	1-85,87-183,185-267,269-274
		fasting overnight	DSNG	86,184,268,275
3/F	70202F	Excretion		
		feces: normal (1.observation)	PRED	1-15
			DSNG	1-275
		Qualitative food consumption		
		food consumption: normal (1.observation)	DSNG	1-85,87-183,185-267,269-274
		fasting overnight	DSNG	86,184,268,275
3/F	70226F	Excretion		
		feces: normal (1.observation)	PRED	1-15
			DSNG	1-275
		Qualitative food consumption		
		food consumption: normal (1.observation)	DSNG	1-85,87-183,185-267,269-274
		fasting overnight	DSNG	86,184,268,275

Table
Individual Clinical Observations
Test Item (dosage) 1 2 3 4

[redacted]

Group/ Sex	Animal Number	Observation	Phase	Day(s)
3/F	70228F	Excretion		
		feces: normal (1.observation)	PRED	1-15
			DSNG	1-275
		Qualitative food consumption		
		food consumption: normal (1.observation)	DSNG	1-85,87-183,185-267,269-274
		fasting overnight	DSNG	86,184,268,275
4/F	70134F	Excretion		
		feces: normal (1.observation)	PRED	1-15
			DSNG	1-19,21-275
		Qualitative food consumption		
		food consumption: normal (1.observation)	DSNG	1-85,87-183,185-267,269-274
		Excretion		
		feces: soft feces (1.observation)	DSNG	20
		Qualitative food consumption		
		fasting overnight	DSNG	86,184,268,275

Table
Individual Clinical Observations
Test Item (dosage) 1 2 3 4

[redacted]

Group/ Sex	Animal Number	Observation	Phase	Day(s)
4/F	70184F	Excretion		
		feces: normal (1.observation)	PRED	1-15
			DSNG	1-19,21-275
		Qualitative food consumption		
		food consumption: normal (1.observation)	DSNG	1-85,87-183,185-267,269-274
		Excretion		
		feces: soft feces (1.observation)	DSNG	20
		Qualitative food consumption		
		fasting overnight	DSNG	86,184,268,275
4/F	70185F	Excretion		
		feces: normal (1.observation)	PRED	1-15
			DSNG	1-19,21-275
		Qualitative food consumption		
		food consumption: normal (1.observation)	DSNG	1-85,87-183,185-267,269-274
		Excretion		
		feces: soft feces (1.observation)	DSNG	20
		Qualitative food consumption		
		fasting overnight	DSNG	86,184,268,275

Table
Individual Clinical Observations
Test Item (dosage) 1 2 3 4

[redacted]

Group/ Sex	Animal Number	Observation	Phase	Day(s)
4/F	70190F	Excretion		
		feces: normal (1.observation)	PRED	1-15
			DSNG	1-19,21-275
		Qualitative food consumption		
		food consumption: normal (1.observation)	DSNG	1-85,87-183,185-267,269-274
		Excretion		
		feces: soft feces (1.observation)	DSNG	20
		Qualitative food consumption		
		fasting overnight	DSNG	86,184,268,275
4/F	70191F	Excretion		
		feces: normal (1.observation)	PRED	1-15
			DSNG	1-19,21-275
		Qualitative food consumption		
		food consumption: normal (1.observation)	DSNG	1-85,87-183,185-267,269-274
		Excretion		
		feces: soft feces (1.observation)	DSNG	20
		Qualitative food consumption		
		fasting overnight	DSNG	86,184,268,275

Table
Individual Clinical Observations
Test Item (dosage) 1 2 3 4

[redacted]

Group/ Sex	Animal Number	Observation	Phase	Day(s)
4/F	70192F	Excretion		
		feces: normal (1.observation)	PRED	1-15
			DSNG	1-19,21-274
		Qualitative food consumption		
		food consumption: normal (1.observation)	DSNG	1-85,87-183,185-267,269-274
		Excretion		
		feces: soft feces (1.observation)	DSNG	20
		Qualitative food consumption		
		fasting overnight	DSNG	86,184,268
		Excretion		
		feces: normal (1.observation)	RECO	1-182
		Qualitative food consumption		
		food consumption: normal (1.observation)	RECO	1-181
		fasting overnight	RECO	182

Table
Individual Clinical Observations
Test Item (dosage) 1 2 3 4

[redacted]

Group/ Sex	Animal Number	Observation	Phase	Day(s)
4/F	70215F	Excretion		
		feces: normal (1.observation)	PRED	1-15
			DSNG	1-19,21-274
		Qualitative food consumption		
		food consumption: normal (1.observation)	DSNG	1-85,87-183,185-267,269-274
		Excretion		
		feces: soft feces (1.observation)	DSNG	20
		Qualitative food consumption		
		fasting overnight	DSNG	86,184,268
		Excretion		
		feces: normal (1.observation)	RECO	1-182
		Qualitative food consumption		
		food consumption: normal (1.observation)	RECO	1-181
		fasting overnight	RECO	182

Data in Support of Table 2: Study Q, Serum GLDH

Table
Summary of Clinical Chemistry
[redacted] tem [redacted] ge) [redacted]

Group/Sex	Phase / Day	Predose 6	Dosing 23	Dosing 92	Recovery 29
		GLDH. U/L			
1/M	Mean	51.74	-	25.03	24.42
	SD	54.361	-	19.811	19.474
	N	6	-	6	2
2/M	Mean	16.88	-	10.53	-
	SD	6.873	-	1.471	-
	N	4	-	3	-
3/M	Mean	21.21	-	10.42	-
	SD	9.609	-	5.775	-
	N	4	-	4	-
4/M	Mean	23.07	5.10	22.92	13.01
	SD	12.655	-	11.768	7.311
	N	6	1	5	2
	Statistics	AT	X	AT	X7

A = ANOVA and Dunnett's
T = Rank-transformed data
X = No analysis performed
X7 = Not analyzed (mean of actual group sizes too small)

Table
Summary of Clinical Chemistry
[redacted]

Group/ Sex	Phase / Day	BU. mmol/L Predose 8	BU. mmol/L Dosing 92	BU. mmol/L Recovery 29	GLDH. U/L Predose 8	GLDH. U/L Dosing 92	GLDH. U/L Recovery 29
1/F	Mean	4.83	4.52	3.88	5.27	12.36	9.25
	SD	1.048	0.572	0.332	1.043	4.048	1.534
	N	6	6	2	6	6	2
2/F	Mean	4.87	4.62	-	6.24	12.96	-
	SD	1.075	0.705	-	3.689	2.260	-
	N	4	4	-	4	4	-
3/F	Mean	4.93	4.31	-	9.00	13.70	-
	SD	1.483	0.686	-	4.209	6.818	-
	N	4	4	-	4	4	-
4/F	Mean	5.76	5.17	4.51	7.41	205.63	7.46
	SD	2.412	1.350	-	3.121	184.433	-
	N	6	6	1	6	6	1
	Statistics	A	A	X	A	AT	X

A = ANOVA and Dunnett's
X = No analysis performed
T = Rank-transformed data

Data in Support of Table 2: Study Q, Serum AST

Table
Summary of Clinical Chemistry
[redacted] tem [redacted] ge) [redacted]

Group/ Sex	Phase / Day	Predose 6	AST. U/L Dosing 23	Dosing 92	Recovery 29
1/M	Mean	218.42	-	115.11	120.40
	SD	42.537	-	33.240	20.775
	N	6	-	6	2
2/M	Mean	243.97	-	111.29	-
	SD	68.212	-	5.633	-
	N	4	-	3	-
3/M	Mean	233.82	-	116.32	-
	SD	63.241	-	13.580	-
	N	4	-	4	-
4/M	Mean	223.02	252.99	121.22	111.12
	SD	59.454	-	37.080	45.863
	N	6	1	5	2
	Statistics	A	X	A	X7

A = ANOVA and Dunnett's
X = No analysis performed
X7 = Not analyzed (mean of actual group sizes too small)

Table
Summary of Clinical Chemistry
[redacted]tem [redacted]ge) [redacted]

Group/Sex	Phase / Day	AST. U/L Predose 8	AST. U/L Dosing 92	AST. U/L Recovery 29	ALT. U/L Predose 8	ALT. U/L Dosing 92	ALT. U/L Recovery 29
1/F	Mean	100.54	139.78	126.94	4.44	2.40	1.20
	SD	16.524	43.387	77.435	2.756	1.887	0.064
	N	6	6	2	6	5	2
2/F	Mean	115.39	134.57	-	4.47	1.12	-
	SD	45.642	35.811	-	3.596	0.612	-
	N	4	4	-	4	4	-
3/F	Mean	123.67	188.51	-	11.14	1.85	-
	SD	30.694	62.053	-	9.641	0.714	-
	N	4	4	-	4	4	-
4/F	Mean	115.42	231.08*	82.05	3.14	11.43	2.49
	SD	35.407	76.417	-	1.979	11.692	-
	N	6	6	1	6	6	1
	Statistics	A	A	X	AT	AT	X

* P<=0.05
** P<=0.01
*** P<=0.001
A = ANOVA and Dunnett's
X = No analysis performed
T = Rank-transformed data

Data in Support of Table 2: Study Q, Serum ALT

Table
Summary of Clinical Chemistry
[redacted]

Group/ Sex	Phase / Day	Predose 6	ALT. U/L Dosing 23	Dosing 92	Recovery 29
1/M	Mean	13.24	-	1.57	3.71
	SD	12.726	-	0.594	2.128
	N	6	-	5	2
2/M	Mean	8.18	-	2.30	-
	SD	4.622	-	2.087	-
	N	4	-	3	-
3/M	Mean	5.84	-	0.91	-
	SD	4.124	-	0.813	-
	N	4	-	3	-
4/M	Mean	12.35	6.54	2.59	0.93
	SD	8.225	-	2.584	0.792
	N	6	1	5	2
	Statistics	A	X	A	X7

A = ANOVA and Dunnett's
X = No analysis performed
X7 = Not analyzed (mean of actual group sizes too small)

Table
Summary of Clinical Chemistry
[redacted]

Group/ Sex	Phase / Day	AST. U/L Predose 8	AST. U/L Dosing 92	AST. U/L Recovery 29	ALT. U/L Predose 8	ALT. U/L Dosing 92	ALT. U/L Recovery 29
1/F	Mean	100.54	139.78	126.94	4.44	2.40	1.20
	SD	16.524	43.387	77.435	2.756	1.887	0.064
	N	6	6	2	6	5	2
2/F	Mean	115.39	134.57	-	4.47	1.12	-
	SD	45.642	35.811	-	3.596	0.612	-
	N	4	4	-	4	4	-
3/F	Mean	123.67	188.51	-	11.14	1.85	-
	SD	30.694	62.053	-	9.641	0.714	-
	N	4	4	-	4	4	-
4/F	Mean	115.42	231.08*	82.05	3.14	11.43	2.49
	SD	35.407	76.417	-	1.979	11.692	-
	N	6	6	1	6	6	1
	Statistics	A	A	X	AT	AT	X

* P<=0.05
** P<=0.01
*** P<=0.001
A = ANOVA and Dunnett's
X = No analysis performed
T = Rank-transformed data

Data in Support of Table 2: Study Q, Serum GGT

Table
Summary of Clinical Chemistry
[redacted]tem [redacted]ge) [redacted]

Group/ Sex	Phase / Day	GGT. U/L Predose 6	GGT. U/L Dosing 23	GGT. U/L Dosing 92	GGT. U/L Recovery 29
1/M	Mean	15.00	-	10.49	9.80
	SD	6.822	-	2.717	1.506
	N	6	-	6	2
2/M	Mean	5.74	-	6.53	-
	SD	1.560	-	0.087	-
	N	3	-	3	-
3/M	Mean	8.27	-	8.49	-
	SD	2.662	-	2.833	-
	N	4	-	4	-
4/M	Mean	15.64	18.57	19.56	8.14
	SD	5.845	-	8.388	1.662
	N	6	1	5	2
	Statistics	A	X	AT	X7

A = ANOVA and Dunnett's
X = No analysis performed
T = Rank-transformed data
X7 = Not analyzed (mean of actual group sizes too small)

Table
Summary of Clinical Chemistry
[redacted] tem [redacted] ge) [redacted]

Group/ Sex	Phase / Day	ALP. U/L Predose 8	ALP. U/L Dosing 92	ALP. U/L Recovery 29	GGT. U/L Predose 8	GGT. U/L Dosing 92	GGT. U/L Recovery 29
1/F	Mean	78.80	128.81	92.31	9.29	12.06	4.87
	SD	15.722	39.769	49.356	3.000	3.560	0.354
	N	6	6	2	6	6	2
2/F	Mean	84.00	144.64	-	8.06	10.58	-
	SD	21.664	33.730	-	1.268	1.175	-
	N	4	4	-	4	4	-
3/F	Mean	89.46	124.86	-	8.04	13.97	-
	SD	26.911	26.956	-	1.412	8.250	-
	N	4	4	-	4	4	-
4/F	Mean	68.59	160.09	65.11	8.12	155.37*	7.68
	SD	13.373	59.631	-	2.809	168.089	-
	N	6	6	1	6	6	1
	Statistics	A	A	X	A	AT	X

* P<=0.05
** P<=0.01
*** P<=0.001
A = ANOVA and Dunnett's
X = No analysis performed
T = Rank-transformed data

Data in Support of Table 2: Study Q, Vomiting

Test Item (dosage) 1 2 3 4

[redacted] [redacted] [redacted]

Group/ Sex	Animal Number	Observation	Phase	Day(s)
1/M	P0001	NORMAL		
		No remarkable observations	PRED	15
			DSNG	1,8,15,22,29,36,43,50,57,64, 71,78,85,91
1/M	P0002	NORMAL		
		No remarkable observations	PRED	15
			DSNG	1,8,22,29,36,43,50,57,64,71, 78,85,91
		Eye/s		
		discolored skin, right eyebrow, red, small area	DSNG	15
1/M	P0003	NORMAL		
		No remarkable observations	PRED	15
			DSNG	1,8,15,22,29,36,43,50,57,64, 71,78,85,91
			RECO	1,8,15,22,28
1/M	P0004	NORMAL		
		No remarkable observations	PRED	15
			DSNG	1,8,15,29,36,43,50,57,64,71, 78,85,91
			RECO	1,8,15,22,28
		Mouth/ vomiting		
		lesion/s, right angle of mouth, crusted	DSNG	22
		Trunk		
		spots, lower abdomen, brown	DSNG	22

Table
Individual Clinical Observations
Test Item (dosage) 1 2 3 4

[redacted] [redacted] [redacted]

Group/ Sex	Animal Number	Observation	Phase	Day(s)
1/M	P0005	NORMAL		
		No remarkable observations	PRED	15
			DSNG	1,8,15,29,36,43,50,57,64,71, 78,85,91
		Behavior/appearance		
		fur abnormalities: glued	DSNG	22
1/M	P0006	NORMAL		
		No remarkable observations	PRED	15
			DSNG	1,8,29,36,43,50,57,64,71,78, 85,91
		Mouth/ vomiting		
		lesion/s, both angles of mouth, crusted	DSNG	22
		lesion/s, left angle of mouth, crusted	DSNG	15
2/M	P0101	NORMAL		
		No remarkable observations	PRED	15
			DSNG	1,8,15,22,29,36,43,50,57,64, 71,78,85,91
2/M	P0102	NORMAL		
		No remarkable observations	PRED	15
			DSNG	1,8,15,22,29,36,43,50,57,64, 71,78,85,91
2/M	P0103	NORMAL		
		No remarkable observations	PRED	15
			DSNG	1,8

Table
Individual Clinical Observations
Test Item (dosage) 1 2 3 4

[redacted] [redacted] [redacted]

Group/ Sex	Animal Number	Observation	Phase	Day(s)
2/M	P0104	NORMAL		
		No remarkable observations	PRED	15
			DSNG	1,8,15,22,29,36,43,50,57,64, 71,78,85,91
3/M	P0201	NORMAL		
		No remarkable observations	PRED	15
			DSNG	1,8,15,22,29,36,43,50,57,64, 71,78,85,91
3/M	P0202	NORMAL		
		No remarkable observations	PRED	15
			DSNG	22,29,36,43,50,57,64,71,78, 85,91
		Extremity/ies lesion/s, left finger/s, crusted	DSNG	1,8,15
3/M	P0203	NORMAL		
		No remarkable observations	PRED	15
			DSNG	1,8,15,22,29,36,43,50,57,64, 71,78,85,91
3/M	P0204	NORMAL		
		No remarkable observations	PRED	15
			DSNG	1,8,15,22,29,36,43,50,57,64, 71,78,85,91

Table
Individual Clinical Observations
Test Item (dosage) 1 2 3 4

[redacted] [redacted] [redacted]

Group/ Sex	Animal Number	Observation	Phase	Day(s)
4/M	P0301	NORMAL		
		No remarkable observations	PRED	15
			DSNG	1,36,43,50,57,64,71,85,91
			RECO	1,8,15,22,28
		Head		
		swelling/s, moderate, left cheek, indurated	DSNG	78
		Mouth/ vomiting		
		lesion/s, right angle of mouth, crusted	DSNG	8,15,22,29
4/M	P0302	NORMAL		
		No remarkable observations	PRED	15
		Mouth/ vomiting		
		lesion/s, left angle of mouth, bloody	DSNG	15
		Trunk		
		swelling/s, severe, chest, soft	DSNG	1,8,15,22
4/M	P0303	NORMAL		
		No remarkable observations	PRED	15
			DSNG	1,8,15,22,29,36,43,50,57,64, 71,78,85,91

Table
Individual Clinical Observations
Test Item (dosage) 1 2 3 4

[redacted] [redacted] [redacted]

Group/ Sex	Animal Number	Observation	Phase	Day(s)
4/M	P0304	NORMAL		
		No remarkable observations	PRED	15
			DSNG	1,8,15,22,29,36,43,50,57,64, 71,78,85,91
			RECO	1,8,15,22,28
4/M	P0305	NORMAL		
		No remarkable observations	PRED	15
			DSNG	1,8,15,22,29,36,43,50,57,64, 71,78,85,91
4/M	P0306	NORMAL		
		No remarkable observations	PRED	15
			DSNG	1,8,15,22,29,36,43,50,57,64, 71,78,85,91

Table
Individual Clinical Observations
Test Item (dosage) 1 2 3 4

[redacted] [redacted] [redacted]

Group/ Sex	Animal Number	Observation	Phase	Day(s)
1/F	P0401	NORMAL		
		No remarkable observations	PRED	20
			DSNG	1,8,15,22,29,36,43,50,57,64, 71,78,85,91
1/F	P0402	NORMAL		
		No remarkable observations	PRED	20
			DSNG	1,8,15,22,29,43,50,57,64,71, 78,85,91
			RECO	1,8,15,22,28
		Head		
		discolored skin, forehead, red, small area	DSNG	36
1/F	P0403	NORMAL		
		No remarkable observations	PRED	20
			DSNG	1,15,22,29,36,43,50,57,64, 71,78,85,91
		Extremity/ies		
		lesion/s, right finger/s, bloody	DSNG	8
1/F	P0404	NORMAL		
		No remarkable observations	PRED	20
			DSNG	1,8,15,22,29,36,43,50,57,85, 91
		Trunk		
		lesion/s, upper part of back, crusted	DSNG	64,71,78

Table
Individual Clinical Observations
Test Item (dosage) 1 2 3 4

[redacted] [redacted] [redacted]

Group/ Sex	Animal Number	Observation	Phase	Day(s)
1/F	P0405	NORMAL		
		No remarkable observations	PRED	20
			DSNG	1,8,15,22,29,36,43,50,57,64, 71,78,85,91
			RECO	1,8,15,22,28
1/F	P0406	NORMAL		
		No remarkable observations	PRED	20
			DSNG	22,29,43,50,57,64,71,78,85, 91
		Eye/s		
		lesion/s, left eyebrow, crusted	DSNG	15
		Mouth/ vomiting		
		lesion/s, left angle of mouth, crusted	DSNG	36
		Trunk		
		discolored skin, upper abdomen, white, small area	DSNG	1,8,15
2/F	P0501	NORMAL		
		No remarkable observations	PRED	20
			DSNG	1,8,15,22,29,36,43,50,57,64, 71,78,85,91

Table
Individual Clinical Observations
Test Item (dosage) 1 2 3 4

[redacted] [redacted] [redacted]

Group/ Sex	Animal Number	Observation	Phase	Day(s)
2/F	P0502	NORMAL		
		No remarkable observations	PRED	20
			DSNG	1,8,15,36,43,50,57,64,71,78, 85,91
		Mouth/ vomiting		
		lesion/s, left angle of mouth, crusted	DSNG	22,29
2/F	P0503	NORMAL		
		No remarkable observations	PRED	20
			DSNG	1,8,15,22,29,36,43,50,57,64, 71,78,85,91
2/F	P0504	NORMAL		
		No remarkable observations	PRED	20
			DSNG	1,22,29,36,43,50,57,64,71, 78,85,91
		Extremity/ies		
		lesion/s, right finger/s, bloody	DSNG	8
		Eye/s		
		lesion/s, left eyebrow, crusted	DSNG	15
3/F	P0601	NORMAL		
		No remarkable observations	PRED	20
			DSNG	1,8,15,22,29,36,43,50,57,64, 71,78,85,91

Table
Individual Clinical Observations
Test Item (dosage) 1 2 3 4

[redacted] [redacted] [redacted]

Group/ Sex	Animal Number	Observation	Phase	Day(s)
3/F	P0602	NORMAL		
		No remarkable observations	PRED	20
			DSNG	1,8,15,22,29,36,43,50,57,64, 71,78,85,91
3/F	P0603	NORMAL		
		No remarkable observations	PRED	20
			DSNG	1,8,15,22,29,36,43,50,57,64, 71,78,85,91
3/F	P0604	NORMAL		
		No remarkable observations	PRED	20
			DSNG	1,8,15,22,29,36,43,50,57,64, 71,78,85,91
4/F	P0701	NORMAL		
		No remarkable observations	PRED	20
			DSNG	1,8,15,22,29,36,43,50,57,64, 71,78,85,91
4/F	P0702	NORMAL		
		No remarkable observations	PRED	20
			DSNG	1,8,15,22,29,36,43,50,57,64, 71,78,85,91
			RECO	1,8,15,22,28

Table
Individual Clinical Observations
Test Item (dosage) 1 2 3 4

[redacted] [redacted] [redacted]

Group/ Sex	Animal Number	Observation	Phase	Day(s)
4/F	P0703	NORMAL		
		No remarkable observations	PRED	20
			DSNG	1,8,15,22,36,43,50,57,64,71, 78,85,91
		Mouth/ vomiting		
		lesion/s, tongue, bloody	DSNG	29
4/F	P0704	NORMAL		
		No remarkable observations	PRED	20
			DSNG	1,8,15,22,29,36,43,50,57,64, 71,78,85,91
			RECO	1,8,15,22,28
4/F	P0705	NORMAL		
		No remarkable observations	PRED	20
			DSNG	1,22,29,36,43,50,57,64,71, 78,85,91
		Extremity/ies		
		lesion/s, right finger/s, crusted	DSNG	8,15
4/F	P0706	NORMAL		
		No remarkable observations	PRED	20
			DSNG	1,8,15,22,29,36,43,50,57,64, 71,78,85
		Extremity/ies		
		lesion/s, right arm, crusted	DSNG	91

Data in Support of Table 2: Study Q, Body weight

[redacted]tem[redacted]ge)[redacted]

Group/ Sex	Phase / Day	PRED 1	PRED 15	PRED 20	DSNG 1	DSNG 8	DSNG 15
		Data Presented in "g"					
1/M	Mean	468	442	-	449	429	412
	SD	21.9	29.4	-	28.1	25.1	31.5
	N	6	6	-	6	6	6
2/M	Mean	425	403	-	407	384	376
	SD	46.1	30.1	-	31.1	13.5	12.4
	N	4	4	-	4	4	3
3/M	Mean	432	403	-	406	382	377
	SD	40.7	47.8	-	49.4	43.2	45.7
	N	4	4	-	4	4	4
4/M	Mean	444	429	-	424	408	402
	SD	29.6	27.7	-	31.8	34.0	22.9
	N	6	6	-	6	6	6
	Statistics	A	A	-	A	A	AT

A = ANOVA and Dunnett's
T = Rank-transformed data

Table
Summary of Body Weight
[redacted] tem [redacted] ge) [redacted]

Group/ Sex	Phase / Day	Data Presented in "g"					
		DSNG					
		22	29	36	43	50	57
1/M	Mean	401	406	401	405	401	410
	SD	30.6	29.8	28.7	27.6	27.2	26.3
	N	6	6	6	6	6	6
2/M	Mean	366	372	375	381	377	383
	SD	18.6	16.1	20.4	24.6	29.1	31.0
	N	3	3	3	3	3	3
3/M	Mean	377	383	385	394	394	393
	SD	39.5	44.2	49.1	46.9	48.0	49.0
	N	4	4	4	4	4	4
4/M	Mean	394	402	399	400	401	408
	SD	26.9	22.4	17.5	20.4	23.9	28.2
	N	6	5	5	5	5	5
	Statistics	A	AT	AT	AT	A	A

A = ANOVA and Dunnett's
T = Rank-transformed data

Table
Summary of Body Weight
[redacted] tem [redacted] ge) [redacted]

Group/ Sex	Phase / Day	DSNG 64	DSNG 71	DSNG 78	DSNG 85	DSNG 91	RECO 1
		Data Presented in "g"					
1/M	Mean	409	420	417	426	418	412
	SD	23.3	22.1	20.6	22.9	20.5	2.1
	N	6	6	6	6	6	2
2/M	Mean	384	390	391	391	392	-
	SD	27.8	24.6	31.9	23.6	33.6	-
	N	3	3	3	3	3	-
3/M	Mean	388	394	391	396	397	-
	SD	53.0	49.2	48.6	51.5	52.3	-
	N	4	4	4	4	4	-
4/M	Mean	404	415	411	415	414	383
	SD	33.8	36.2	43.3	43.7	40.9	35.4
	N	5	5	5	5	5	2
	Statistics	A	A	A	A	A	X7

A = ANOVA and Dunnett's
X7 = Not analyzed (mean of actual group sizes too small)

Table
Summary of Body Weight
tem ge)
[redacted] [redacted] [redacted]

Group/ Sex	Phase / Day	RECO 8	RECO 15	RECO 22	RECO 28
		Data Presented in "g"			
1/M	Mean	420	425	428	413
	SD	9.2	21.2	12.0	21.9
	N	2	2	2	2
4/M	Mean	376	373	380	363
	SD	43.8	46.0	43.8	41.7
	N	2	2	2	2
	Statistics	X7	X7	X7	X7

X7 = Not analyzed (mean of actual group sizes too small)

Table
Summary of Body Weight
[redacted] tem [redacted] ge) [redacted]

Group/ Sex	Phase / Day	PRED 1	PRED 15	PRED 20	DSNG 1	DSNG 8	DSNG 15
		Data Presented in "g"					
1/F	Mean	496	-	469	478	451	443
	SD	69.5	-	67.0	71.6	70.7	71.0
	N	6	-	6	6	6	6
2/F	Mean	459	-	424	427	408	405
	SD	67.2	-	64.9	63.4	52.7	51.4
	N	4	-	4	4	4	4
3/F	Mean	449	-	426	420	402	391
	SD	48.4	-	52.6	46.5	43.5	36.5
	N	4	-	4	4	4	4
4/F	Mean	467	-	449	449	432	431
	SD	25.1	-	36.4	37.8	43.4	43.2
	N	6	-	6	6	6	6
	Statistics	A	-	A	A	A	A

A = ANOVA and Dunnett's

Table
Summary of Body Weight
[redacted] tem [redacted] ge) [redacted]

Group/ Sex	Phase / Day	DSNG 22	29	36	43	50	57
		Data Presented in "g"					
1/F	Mean	438	434	440	436	444	440
	SD	70.2	66.6	67.8	72.3	73.9	80.9
	N	6	6	6	6	6	6
2/F	Mean	393	396	401	400	404	405
	SD	45.4	50.9	42.4	41.8	38.7	38.9
	N	4	4	4	4	4	4
3/F	Mean	385	381	393	392	394	398
	SD	38.4	43.2	34.1	36.3	37.3	42.0
	N	4	4	4	4	4	4
4/F	Mean	420	428	429	430	431	436
	SD	45.9	53.6	50.2	49.3	48.3	54.3
	N	6	6	6	6	6	6
	Statistics	A	A	A	A	A	A

A = ANOVA and Dunnett's

Table
Summary of Body Weight
[redacted]tem[redacted]ge)[redacted]

Group/Sex	Phase / Day	DSNG 64	DSNG 71	DSNG 78	DSNG 85	DSNG 91	RECO 1
		Data Presented in "g"					
1/F	Mean	447	445	455	456	451	405
	SD	76.7	76.4	75.4	77.5	76.6	109.6
	N	6	6	6	6	6	2
2/F	Mean	406	400	410	410	405	-
	SD	40.6	42.3	44.8	43.1	45.2	-
	N	4	4	4	4	4	-
3/F	Mean	404	405	402	409	407	-
	SD	41.1	44.3	49.1	49.7	53.8	-
	N	4	4	4	4	4	-
4/F	Mean	446	445	453	453	449	429
	SD	57.0	53.7	58.8	59.4	60.6	67.2
	N	6	6	6	6	6	2
	Statistics	A	A	A	A	A	X7

A = ANOVA and Dunnett's
X7 = Not analyzed (mean of actual group sizes too small)

Table
Summary of Body Weight
[redacted] tem [redacted] ge) [redacted]

Group/ Sex	Phase / Day	RECO 8	RECO 15	RECO 22	RECO 28
		Data Presented in "g"			
1/F	Mean	438	446	417	415
	SD	132.9	133.6	115.3	114.6
	N	2	2	2	2
4/F	Mean	459	451	459	439
	SD	55.9	66.5	63.6	64.3
	N	2	2	2	2
	Statistics	X7	X7	X7	X7

X7 = Not analyzed (mean of actual group sizes too small)

Data in Support of Table 2: Study Q, Serum albumin

Table
Summary of Clinical Chemistry
[redacted] tem [redacted] ge) [redacted]

Group/ Sex	Phase / Day	Predose 6	ALB. g/L Dosing 23	ALB. g/L Dosing 92	Recovery 29
1/M	Mean	53.51	-	42.73	42.44
	SD	7.512	-	1.038	0.078
	N	6	-	6	2
2/M	Mean	47.61	-	40.41	-
	SD	8.203	-	1.235	-
	N	4	-	3	-
3/M	Mean	44.63	-	36.59	-
	SD	8.988	-	5.553	-
	N	4	-	4	-
4/M	Mean	43.70	23.26	42.01	37.58
	SD	9.071	-	7.970	5.438
	N	6	1	5	2
	Statistics	A	X	A	X7

A = ANOVA and Dunnett's
X = No analysis performed
X7 = Not analyzed (mean of actual group sizes too small)

Table
Summary of Clinical Chemistry
[redacted] tem [redacted] ge) [redacted]

Group/Sex	Phase / Day	TP. g/L Predose 8	TP. g/L Dosing 92	TP. g/L Recovery 29	ALB. g/L Predose 8	ALB. g/L Dosing 92	ALB. g/L Recovery 29
1/F	Mean	75.32	69.82	64.82	44.19	38.90	37.29
	SD	5.172	5.211	3.493	8.339	2.658	2.291
	N	6	6	2	6	6	2
2/F	Mean	76.08	71.87	-	42.01	39.89	-
	SD	4.089	0.517	-	2.033	0.493	-
	N	4	4	-	4	4	-
3/F	Mean	74.11	65.69	-	41.67	37.45	-
	SD	0.847	4.142	-	2.134	3.159	-
	N	4	4	-	4	4	-
4/F	Mean	79.15	71.23	74.16	44.85	39.77	39.79
	SD	4.552	4.497	-	5.318	2.918	-
	N	6	6	1	6	6	1
	Statistics	A	A	X	A	A	X

A = ANOVA and Dunnett's
X = No analysis performed

Data in Support of Table 2: Study R, Blood CD20+ lymphocytes

Table
Summary of Immunophenotyping
Test Item (dosage) 1 2 3
[redacted]

Group/ Sex	Phase / Day	CD20+ B cells absolute (10E9/L) and % of Lymphocytes: Predose, 8 (10E9/L)	Predose, 8 (%)	Predose, 16 (10E9/L)	Predose, 16 (%)	Dosing, 85 (10E9/L)	Dosing, 85 (%)	Recovery, 50 (10E9/L)	Recovery, 50 (%)
1/M	Mean	1.42	34.09	1.60	30.16	1.29	33.25	1.56	43.94
	SD	1.09	15.83	0.96	15.75	1.18	14.69	-	-
	N	6	6	6	6	6	6	2	2
2/M	Mean	1.24	23.80	1.26	21.22	0.32 **	6.36 **	0.82	17.44
	SD	0.38	6.16	0.48	6.16	0.37	7.00	-	-
	N	6	6	6	6	6	6	2	2
3/M	Mean	1.90	25.86	2.09	23.59	0.00 ***	0.01 ***	0.00	0.00
	SD	0.93	7.37	1.04	7.03	0.00	0.01	-	-
	N	6	6	6	6	6	6	2	2
	Statistics	A	AT	A	AT	AT	AT	X	X

Standard deviation not calculated for less than three values

Statistical analysis was performed using SAS release 9.2
A = ANOVA and Dunnett's * P≤ 0.05
T = Rank-transformed data ** P≤ 0.01
X = No analysis performed *** P≤ 0.001

Table
Summary of Immunophenotyping
Test Item (dosage) 1 2 3
[redacted]

Group/ Sex	Phase / Day	CD20+ B cells absolute (10E9/L) and % of Lymphocytes							
	Phase	Predose				Dosing		Recovery	
	Day	65		71		85		52	
		(10E9/L)	(%)	(10E9/L)	(%)	(10E9/L)	(%)	(10E9/L)	(%)
1/F	Mean	0.96	23.87	1.14	21.40	0.86	25.40	1.15	28.28
	SD	0.47	8.65	0.49	5.97	0.25	8.09	-	-
	N	6	6	6	6	6	6	2	2
2/F	Mean	0.71	23.17	0.86	21.87	0.12 ***	4.19 **	0.00	0.09
	SD	0.46	10.02	0.52	7.99	0.29	10.24	-	-
	N	6	6	6	6	6	6	2	2
3/F	Mean	1.15	30.09	1.60	30.34	0.00 ***	0.00 ***	0.00	0.00
	SD	0.58	8.00	0.75	7.87	0.00	0.00	-	-
	N	6	6	6	6	6	6	2	2
	Statistics	A	A	A	A	AT	AT	X	X

Standard deviation not calculated for less than three values

Statistical analysis was performed using SAS release 9.2
A = ANOVA and Dunnett's * P≤ 0.05
T = Rank-transformed data ** P≤ 0.01
X = No analysis performed *** P≤ 0.001

Data in Support of Table 2: Study R, Mic (spleen)

Incidence of Microscopic Observations
Terminal Sacrifice
Test Item (dosage) 1 2 3

[redacted]

Tissue/ Observation	Group/Sex: / Number of Animals:	1/M 4	2/M 4	3/M 4	1/F 4	2/F 4	3/F 4
Prostate	Number Examined:	4	4	4	0	0	0
	Unremarkable:	4	4	4	0	0	0
Seminal Vesicle	Number Examined:	4	4	4	0	0	0
	Unremarkable:	4	4	4	0	0	0
Spleen	Number Examined:	4	4	4	4	4	4
	Unremarkable:	4	1	0	3	1	1
Decreased cellularity, lymphoid follicles		0	1	4	0	0	2
Germinal centers, absent/reduced		0	3	4	1	3	3
Testis	Number Examined:	4	4	4	0	0	0
[redacted]	:	3	4	2	0	0	0
		1	0	2	0	0	0
Thymus	Number Examined:	4	4	4	4	4	4
	Unremarkable:	0	0	0	1	2	1
[redacted]		4	4	4	3	2	3
Uterus/Cervix	Number Examined:	0	0	0	4	4	4
	Unremarkable:	0	0	0	4	3	3
[redacted]		0	0	0	0	1	0
		0	0	0	0	0	1

Data in Support of Table 2: Study R, Mic (lymph node)

Incidence of Microscopic Observations
Terminal Sacrifice
Test Item (dosage)   1 2 3
--------- ------------------------- - -- ---

[redacted]

Tissue/ Observation	Group/Sex: / Number of Animals:	1/M	2/M	3/M	1/F	2/F	3/F
		4	4	4	4	4	4
Kidney	Number Examined:	0	0	0	0	0	1
	Unremarkable:	0	0	0	0	0	0
[redacted]		0	0	0	0	0	1
[redacted]		0	0	0	0	0	1
Lymph Node, Mandibular	Number Examined:	4	4	4	4	4	4
	Unremarkable:	4	0	0	3	2	1
Foamy macrophages		0	0	0	1	0	0
Germinal centers, absent/reduced		0	4	4	0	2	3
Hematopoiesis, extramedullary		0	0	2	1	0	1
Lymph Node, Mesenteric	Number Examined:	4	4	4	4	4	4
	Unremarkable:	4	2	0	4	2	1
Germinal centers, absent/reduced		0	2	4	0	2	3
Hematopoiesis, extramedullary		0	0	1	0	0	0
Mammary Gland	Number Examined:	4	4	4	4	4	4
	Unremarkable:	4	3	4	4	4	4
[redacted]		0	1	0	0	0	0
Ovary	Number Examined:	0	0	0	4	4	4
	Unremarkable:	0	0	0	4	4	3
[redacted]e		0	0	0	0	0	1

Data in Support of Table 2: Study S, Serum IgG

Table
Summary of Clinical Chemistry
[redacted] em [redacted]

Group/Sex	Phase / Day / Session / Name	Predose 1 / 2 / Coag,CC	Predose 18 / 2 / Coag,CC	Predose 19 / 2 / Coag,CC	Predose 33 / 2 / Coag,CC	Dosing 25 / 2 / Coag,CC	Dosing 38 / 3 / ALB
		IGG. g/L					
1/M	Mean	7.94	7.50	8.75	8.57	7.68	7.53
	SD	2.372	3.111	2.481	2.277	2.258	3.111
	N	3	2	3	2	5	2
2/M	Mean	8.25	-	8.57	-	5.04	-
	SD	1.037	-	0.860	-	0.951	-
	N	4	-	4	-	4	-
3/M	Mean	-	9.17	-	9.24	7.13	7.41
	SD	-	2.206	-	2.197	4.959	4.836
	N	-	6	-	6	6	6
	Statistics	S	X9	S	X9	A	X9

S = Two-sample t-test
X9 = Not analysed (too few groups for Levenes test)
A = ANOVA and Dunnett's

Table
Summary of Clinical Chemistry
em
[redacted]

Group/ Sex	Phase / Day / Session / Name	IGG. g/L Dosing 43 / 2 / Coag,CC	56 / 3 / ALB	70 / 3 / ALB	73 / 1 / check	79 / 3 / ALB	86 / 2 / Coag,CC
1/M	Mean	8.58	8.30	8.33	-	9.33	8.48
	SD	1.634	2.106	1.926	-	2.782	1.670
	N	5	5	5	-	3	5
2/M	Mean	6.52	5.82	5.71	-	5.87	5.57
	SD	2.330	1.875	2.278	-	2.497	1.852
	N	4	4	4	-	4	4
3/M	Mean	7.63	5.87	6.71	-	-	5.14
	SD	3.910	3.972	4.050	-	-	3.583
	N	6	6	6	-	-	6
	Statistics	A	A	A	-	S	A

A = ANOVA and Dunnett's
S = Two-sample t-test

Table
Summary of Clinical Chemistry
em

[redacted] [redacted] [redacted]

Group/ Sex	Phase / Day / Session / Name	Dosing 86 / 3 / ALB	IGG. g/L Recovery 6 / 3 / ALB	18 / 3 / ALB	35 / 3 / ALB	49 / 3 / ALB	54 / 2 / Coag,CC
1/M	Mean	9.01	7.85	10.65	10.94	10.83	9.89
	SD	2.114	2.305	0.587	0.127	0.742	0.346
	N	2	2	2	2	2	2
2/M	Mean	-	-	-	-	-	-
	SD	-	-	-	-	-	-
	N	-	-	-	-	-	-
3/M	Mean	5.54	4.14	2.96	8.16	9.40	9.00
	SD	4.521	0.354	0.226	0.827	0.679	0.552
	N	6	2	2	2	2	2
	Statistics	X9	X7	X7	X7	X7	X7

X9 = Not analysed (too few groups for Levenes test)
X7 = Not analyzed (mean of actual group sizes
too small)

Table
Summary of Clinical Chemistry
em
[redacted]

		IGG. g/L					
	Phase	Predose				Dosing	
Group/ Sex	Day<br>Session<br>Name	1<br>2<br>Coag,CC	18<br>2<br>Coag,CC	19<br>2<br>Coag,CC	33<br>2<br>Coag,CC	25<br>2<br>Coag,CC	38<br>3<br>ALB
1/F	Mean	10.76	10.92	11.03	10.12	10.08	10.23
	SD	3.237	0.431	3.278	0.658	1.670	0.297
	N	3	2	3	2	5	2
2/F	Mean	9.77	-	10.34	-	4.75**	-
	SD	1.741	-	1.659	-	1.054	-
	N	4	-	4	-	4	-
3/F	Mean	-	9.15	-	10.14	6.76*	5.96
	SD	-	1.942	-	1.520	2.383	2.194
	N	-	6	-	6	6	6
	Statistics	S	X9	S	X9	A	X9

* P<=0.05
** P<=0.01
*** P<=0.001
S = Two-sample t-test
X9 = Not analysed (too few groups for Levenes test)

A = ANOVA and Dunnett's

Table
Summary of Clinical Chemistry
em
[redacted] [redacted] [redacted]

Group/Sex	Phase / Day / Session / Name	IGG. g/L Dosing 43 / 2 / Coag,CC	56 / 3 / ALB	70 / 3 / ALB	73 / 1 / check	79 / 3 / ALB	86 / 2 / Coag,CC
1/F	Mean	10.68	10.03	9.18	-	10.52	9.75
	SD	1.499	1.892	1.737	-	2.288	1.565
	N	5	5	5	-	3	5
2/F	Mean	6.07**	5.02**	4.24**	-	5.40*	4.61***
	SD	1.610	1.486	1.158	-	1.570	1.171
	N	4	4	4	-	4	4
3/F	Mean	6.69**	5.45**	4.42***	3.85	-	4.44***
	SD	2.256	2.514	1.657	-	-	1.751
	N	6	6	6	1	-	6
	Statistics	A	A	A	X	S	A

* P<=0.05
** P<=0.01
*** P<=0.001
A = ANOVA and Dunnett's
X = No analysis performed
S = Two-sample t-test

Table
Summary of Clinical Chemistry
[redacted] em [redacted] [redacted]

		IGG. g/L					
	Phase	Dosing	Recovery				
Group/ Sex	Day<br>Session<br>Name	86<br>3<br>ALB	6<br>3<br>ALB	18<br>3<br>ALB	35<br>3<br>ALB	49<br>3<br>ALB	54<br>2<br>Coag,CC
1/F	Mean	10.80	9.27	9.96	11.04	10.85	9.79
	SD	0.431	0.552	0.707	0.071	0.636	1.096
	N	2	2	2	2	2	2
2/F	Mean	-	-	-	-	-	-
	SD	-	-	-	-	-	-
	N	-	-	-	-	-	-
3/F	Mean	4.79	2.34	3.12	8.85	9.53	9.14
	SD	1.447	1.846	-	1.909	1.527	2.256
	N	6	2	1	2	2	2
	Statistics	X9	X7	X	X7	X7	X7

X9 = Not analysed (too few groups for Levenes test)
X7 = Not analyzed (mean of actual group sizes
too small)
X = No analysis performed

Data in Support of Table 2: Study S, Serum albumin

Table
Summary of Clinical Chemistry
[redacted] em [redacted]

Group/ Sex	Phase / Day / Session / Name	Predose 1 2 Coag,CC	Predose 18 2 Coag,CC	Predose 19 2 Coag,CC	Predose 33 2 Coag,CC	Dosing 8 3 ALB	Dosing 13 3 ALB
		ALB. g/L					
1/M	Mean	40.78	38.36	46.15	44.15	41.39	42.31
	SD	3.018	8.888	5.716	12.671	1.331	6.801
	N	3	2	3	2	3	5
4/M	Mean	-	41.33	-	43.82	-	31.10*
	SD	-	1.857	-	3.199	-	1.476
	N	-	4	-	4	-	4
	Statistics	X	X9	X	X9	X	S

* P<=0.05
** P<=0.01
*** P<=0.001
X = No analysis performed
X9 = Not analysed (too few groups for Levenes test)

S = Two-sample t-test

Table
Summary of Clinical Chemistry
[redacted] em [redacted] [redacted]

Group/ Sex	Phase / Day / Session / Name	ALB. g/L Dosing					
		25	27	38	43	56	70
		2	2	3	2	3	3
		Coag,CC	Coag,CC	ALB	Coag,CC	ALB	ALB
1/M	Mean	42.57	-	38.90	40.37	38.90	41.44
	SD	7.435	-	4.537	4.177	4.201	6.726
	N	5	-	5	5	5	5
4/M	Mean	-	31.10	-	-	-	-
	SD	-	2.187	-	-	-	-
	N	-	4	-	-	-	-
	Statistics	X	X	X	X	X	X

X = No analysis performed

Table
Summary of Clinical Chemistry
[redacted] em [redacted] [redacted]

	ALB. g/L					
Phase	Dosing			Recovery		
Group/Sex / Day	79	86		6	18	35
Session	3	2	3	3	3	3
Name	ALB	Coag,CC	ALB	ALB	ALB	ALB
1/M Mean	42.08	40.47	39.82	35.74	37.10	37.76
SD	1.004	2.668	5.360	6.619	8.987	4.448
N	3	5	2	2	2	2
4/M Mean	-	-	-	-	-	-
SD	-	-	-	-	-	-
N	-	-	-	-	-	-
Statistics	X	X	X	X	X	X

X = No analysis performed

Table
Summary of Clinical Chemistry
[redacted] em [redacted] [redacted]

Group/ Sex		Phase / Day / Session / Name	ALB. g/L Recovery 49 / 3 / ALB	54 / 2 / Coag,CC
1/M	Mean		38.29	39.01
	SD		2.878	2.383
	N		2	2
4/M	Mean		-	-
	SD		-	-
	N		-	-
	Statistics		X	X

X = No analysis performed

Table
Summary of Clinical Chemistry
em
[redacted]

		ALB. g/L					
	Phase	Predose				Dosing	
Group/ Sex	Day	1	18	19	33	8	13
	Session	2	2	2	2	3	3
	Name	Coag,CC	Coag,CC	Coag,CC	Coag,CC	ALB	ALB
1/F	Mean	40.99	39.83	42.34	40.05	41.11	40.64
	SD	1.342	3.550	1.993	1.556	0.445	1.916
	N	3	2	3	2	3	5
4/F	Mean	-	42.15	-	42.46	-	30.19***
	SD	-	1.642	-	0.680	-	1.064
	N	-	4	-	4	-	4
	Statistics	X	X9	X	X9	X	S

* P<=0.05
** P<=0.01
*** P<=0.001
X = No analysis performed
X9 = Not analysed (too few groups for Levenes test)

S = Two-sample t-test

Table
Summary of Clinical Chemistry
[redacted] em [redacted]

Group/ Sex	Phase / Day / Session / Name	ALB. g/L Dosing 25 / 2 / Coag,CC	27 / 2 / Coag,CC	38 / 3 / ALB	43 / 2 / Coag,CC	56 / 3 / ALB	70 / 3 / ALB
1/F	Mean	40.21	-	39.51	41.56	39.13	39.49
	SD	2.056	-	1.312	1.480	1.597	2.687
	N	5	-	5	5	5	5
4/F	Mean	-	28.52	-	-	-	-
	SD	-	1.148	-	-	-	-
	N	-	4	-	-	-	-
	Statistics	X	X	X	X	X	X

X = No analysis performed

Table
Summary of Clinical Chemistry
em
[redacted]

ALB. g/L

Group/ Sex	Phase / Day / Session / Name	Dosing 79 3 ALB	Dosing 86 2 Coag,CC	Dosing 86 3 ALB	Recovery 6 3 ALB	Recovery 18 3 ALB	Recovery 35 3 ALB
1/F	Mean	40.87	40.57	39.96	38.19	38.74	37.64
	SD	0.576	2.986	3.055	4.278	3.946	2.432
	N	3	5	2	2	2	2
4/F	Mean	-	-	-	-	-	-
	SD	-	-	-	-	-	-
	N	-	-	-	-	-	-
	Statistics	X	X	X	X	X	X

X = No analysis performed

Table
Summary of Clinical Chemistry
em
[redacted]

Group/ Sex		Phase / Day / Session / Name	ALB. g/L Recovery 49 / 3 / ALB	54 / 2 / Coag,CC
1/F	Mean		37.70	37.41
	SD		2.623	2.850
	N		2	2
4/F	Mean		-	-
	SD		-	-
	N		-	-
	Statistics		X	X

X = No analysis performed

Data in Support of Table 2: Study S, TDAR

8337376

Summary of Anti KLH IgG - Males

[redacted] em [redacted]

Group/ Sex	Phase Day	KLHG Dosing 35	42	49	56	70	77
1/M	Mean	154	4131	11548	11476	11209	44504
	SD	84.2	2786.6	1188.5	1236.1	1038.8	12544.8
	N	5	5	5	5	5	5
2/M	Mean	100	1619	2568***	3023***	1405	17788
	SD	0.0	710.3	790.9	1977.1	831.1	13503.4
	N	4	4	4	4	4	4
3/M	Mean	100	3076	8275*	6053**	2019	37904
	SD	0.0	2644.5	3021.7	3312.2	1199.0	19342.2
	N	6	6	6	6	6	6
	Statistics	X1	A	AT	AT	X1	A

* P<=0.05
** P<=0.01
*** P<=0.001
X1 = No analysis required
A = ANOVA and Dunnett's

T = Rank-transformed data

[redacted] em [redacted]

Group/ Sex	Phase / Day	Dosing 84	Dosing 91	KLHG Recovery 28	Recovery 35	Recovery 42	Recovery 49
1/M	Mean	34001	40451	11614	74621	56608	52637
	SD	14053.9	12652.7	283.5	21121.3	3222.3	3908.9
	N	5	5	2	2	2	2
2/M	Mean	8511*	5976***	-	-	-	-
	SD	2185.5	4686.5	-	-	-	-
	N	4	4	-	-	-	-
3/M	Mean	18789	7431**	3180	71941	36365	50518
	SD	12715.0	4046.1	1764.9	51268.8	33639.2	13578.6
	N	6	6	2	2	2	2
	Statistics	A	AT	X7	X7	X7	X7

* P<=0.05
** P<=0.01
*** P<=0.001
A = ANOVA and Dunnett's
T = Rank-transformed data

X7 = Not analyzed (mean of actual group sizes too small)

8337376
Summary of Anti KLH IgG - Females
em
[redacted]

Group/ Sex	Phase / Day	KLHG Dosing 35	42	49	56	70	77
1/F	Mean	105	4499	31203	33505	19746	57114
	SD	10.3	3396.0	19250.2	15463.2	11993.2	4618.3
	N	5	5	5	5	5	5
2/F	Mean	100	1945	7734	8189**	3818	30330
	SD	0.0	322.8	4131.0	4214.3	2152.7	14708.4
	N	4	4	4	4	4	4
3/F	Mean	100	3535	15858	7657***	2954	63096
	SD	0.0	1543.8	11676.1	2766.4	1080.7	29912.2
	N	6	6	6	6	6	6
	Statistics	X1	AT	A	AT	X1	A

* P<=0.05
** P<=0.01
*** P<=0.001
X1 = No analysis required
A = ANOVA and Dunnett's

T = Rank-transformed data

[redacted] em [redacted]

KLHG

Group/ Sex	Phase / Day	Dosing 84	Dosing 91	Recovery 28	Recovery 35	Recovery 42	Recovery 49
1/F	Mean	50796	54117	17986	123619	76231	54776
	SD	3619.9	3473.5	5294.1	89181.7	25496.9	5413.6
	N	5	5	2	2	2	2
2/F	Mean	18191***	8614***	-	-	-	-
	SD	12240.8	2479.4	-	-	-	-
	N	4	4	-	-	-	-
3/F	Mean	29852*	12723***	6764	118645	88053	52206
	SD	12365.1	4950.2	1030.3	89169.0	51862.0	11817.2
	N	6	6	2	2	2	2
	Statistics	A	A	X7	X7	X7	X7

* P<=0.05
** P<=0.01
*** P<=0.001
A = ANOVA and Dunnett's
X7 = Not analyzed (mean of actual group sizes too small)

Data in Support of Table 2: Study T, Mic (injection site)

Incidence of Microscopic Observations
Terminal Sacrifice
Test Item (dosage) 1 2 3

[redacted] ----- [redacted]

Tissue/ Observation	Group/Sex: Number of Animals:	1/M 4	2/M 4	3/M 4	1/F 4	2/F 4	3/F 4
Subcutaneous Injection Site	Number Examined:	4	4	4	4	4	4
	Unremarkable:	4	3	0	3	3	2
Infiltration of inflammatory cells; dermis/subcutis/muscle		0	1	4	1	1	2
Testis	Number Examined:	4	4	4	0	0	0
[redacted]	kable:	0	0	0	0	0	0
[redacted]		1	0	1	0	0	0
[redacted],		0	1	0	0	0	0
[redacted]		4	4	4	0	0	0
Thymus	Number Examined:	4	4	4	4	4	4
	Unremarkable:	0	0	1	1	2	4
[redacted]		1	1	1	2	1	0
		4	4	3	2	1	0
Thyroid	Number Examined:	4	4	4	4	4	4
	Unremarkable:	4	1	2	3	4	2
[redacted]		0	1	2	1	0	0
		0	1	0	0	0	0
		0	1	0	0	0	2
