# Supplementary material for: How important are concurrent vehicle control groups in (sub)chronic non-human primate toxicity studies conducted in pharmaceutical development? An opportunity to reduce animal numbers
Source: PLoS One. 2023 Aug 3;18(8):e0282404. doi: 10.1371/journal.pone.0282404 (PMC10399820; doi:10.1371/journal.pone.0282404)
Supplement: S2 Table — Mean anti-KLH IgG titer from a 26-week intravenous toxicity study in cynomolgus monkeys. Animals (n = 6 per group) were allocated to 3 groups (VEH, vehicle control; LD, low dose test article; HD, high dose test article) and were immunized on days 32 and 60 of the dosing phase. Samples were taken pre-dosing (PD) and throughout dosing of the test article between dosing day 32 (D32) and dosing day 81 (D81). (PDF) [file pone.0282404.s002.pdf]

Supplemental data

Data in support of **Figure 1**: Mean anti-KLH IgG titer from a 26-week intravenous toxicity study in cynomolgus monkeys. Animals (n=6 per group) were allocated to 3 groups (VEH, vehicle control; LD, low dose test article; HD, high dose test article) and were immunized on days 32 and 60 of the dosing phase. Samples were taken pre-dosing (PD) and throughout dosing of the test article between dosing day 32 (D32) and dosing day 81 (D81).

Group	Animal ID	PD	D32	D39	D46	D53	D60	D67	D74	D81
VEH	P0301	100	100	1959	9571	30778	16999	224706	175574	73411
	P0302	100	100	1458	5120	33472	26466	210072	117319	60181
	P0303	100	100	2162	11855	40429	12144	60602	61950	56342
	P0001	181	100	1190	497	581	437	467	353	452
	P0002	999	984	7772	2467	4120	2090	7472	2306	2487
	P0003	496	474	9633	7903	10906	4754	4921	4425	7653
LD	P0401	317	206	1122	351	4788	2262	4577	9863	4460
	P0402	200	100	2691	11742	11600	8708	10576	11262	9052
	P0403	100	100	2486	11323	10160	4763	15677	34179	11093
	P0101	236	129	1925	702	1049	484	481	510	494
	P0102	297	196	7809	2325	4759	2251	2449	2309	2471
	P0103	483	411	2172	1329	1343	963	939	964	1294
HD	P0501	100	235	2466	6971	5968	2442	4261	6166	2414
	P0502	282	297	1841	2367	2352	2218	7910	8506	6403
	P0503	378	334	6825	8093	7838	2599	35325	30756	11725
	P0201	100	100	1347	1403	468	387	429	459	456
	P0202	252	100	2002	2722	1949	1531	1832	2128	2103
	P0203	100	100	1555	1527	726	478	1493	1491	1232
