# Supplementary material for: How important are concurrent vehicle control groups in (sub)chronic non-human primate toxicity studies conducted in pharmaceutical development? An opportunity to reduce animal numbers
Source: PLoS One. 2023 Aug 3;18(8):e0282404. doi: 10.1371/journal.pone.0282404 (PMC10399820; doi:10.1371/journal.pone.0282404)
Supplement: S3 Table — Statistical analysis using mixed-design ANOVA. (PDF) [file pone.0282404.s003.pdf]

Analyse

Studie A

BT female

In folgender ANOVA muss die Annahme der Sphärizität nicht überprüft werden, da nur 2 Zeitpunkte vorlagen. Kommende Tabelle zeigt die Ergebnisse zu dem Haupteffekt Zeit und der Interaktion aus Zeit und Gruppe. Dabei ist der Haupteffekt Zeit signifikant, $F(1, 11) = 41{,}14, p = 0{,}000, \eta^2 = 0{,}79$. Der Effekt ist weiterhin als stark zu beurteilen. Ebenfalls ist die Interaktion signifikant, $F(2, 11) = 6{,}54, p = 0{,}013, \eta^2 = 0{,}54$. Auch der Effekt der Interaktion ist stark.

Tests der Innersubjekteffekte

Maß: MASS_1

Quelle		Typ III Quadratsumme	df	Mittel der Quadrate	F	Sig.	Partielles Eta-Quadrat
Zeit	Sphärizität angenommen	3,658	1	3,658	41,142	,000	,789
	Greenhouse-Geisser	3,658	1,000	3,658	41,142	,000	,789
	Huynh-Feldt (HF)	3,658	1,000	3,658	41,142	,000	,789
	Untergrenze	3,658	1,000	3,658	41,142	,000	,789
Zeit * Gruppe	Sphärizität angenommen	1,162	2	,581	6,536	,013	,543
	Greenhouse-Geisser	1,162	2,000	,581	6,536	,013	,543
	Huynh-Feldt (HF)	1,162	2,000	,581	6,536	,013	,543
	Untergrenze	1,162	2,000	,581	6,536	,013	,543
Fehler(Zeit)	Sphärizität angenommen	,978	11	,089			
	Greenhouse-Geisser	,978	11,000	,089			
	Huynh-Feldt (HF)	,978	11,000	,089			
	Untergrenze	,978	11,000	,089			

Der Haupteffekt Gruppe ist in diesem Modell nicht-signifikant, $F(2, 11) = 0{,}65, p = 0{,}543, \eta^2 = 0{,}11$. Der Effekt zwischen den Gruppen ist mittelstark.

Tests der Zwischensubjekteffekte

Maß: MASS_1

Transformierte Variable: Mittel

Quelle	Typ III Quadratsumme	df	Mittel der Quadrate	F	Sig.	Partielles Eta-Quadrat
Konstanter Term	40609,059	1	40609,059	122958,958	,000	1,000
Gruppe	,427	2	,214	,647	,543	,105
Fehler	3,633	11	,330			

Kommende Grafik zeigt die geschätzten Mittelwerte visuelle. Die Interaktion ist hier klar zu erkennen. Der Anstieg in den Gruppen MD und HD ist höher als bei LD. Weiterhin hat LD zu PRED09 die den höchsten Mittelwert. ZU

DSNG176 jedoch den niedrigsten.

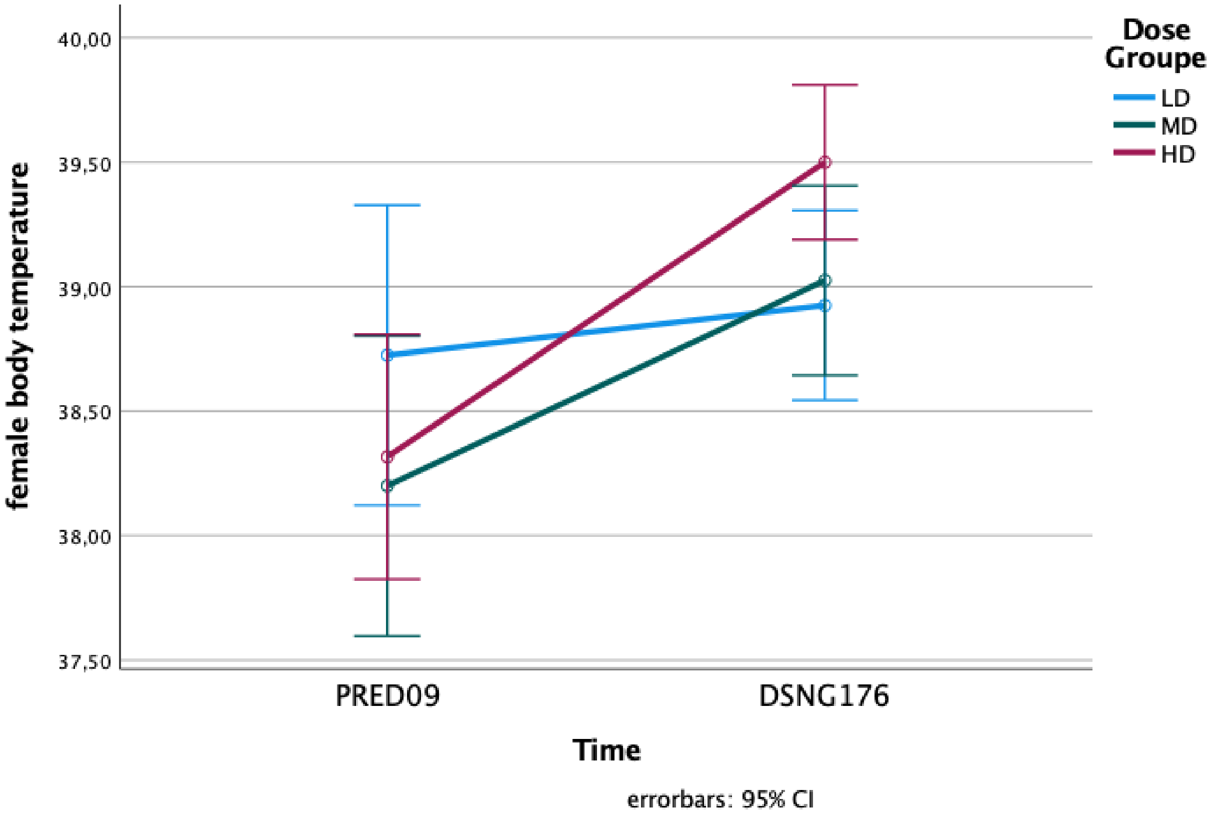

TRIG male

Mauchly-Test auf Sphärizität[a]

Maß: MASS_1

Innersubjekteffekt	Mauchly-W	Ungefähres Chi-Quadrat	df	Sig.	Epsilon[b] Greenhouse-Geisser	Huynh-Feldt (HF)	Untergrenze
Zeit	,246	14,021	2	,001	,570	,708	,500

Prüft die Nullhypothese, dass sich die Fehlerkovarianz-Matrix der orthonormalisierten transformierten abhängigen Variablen proportional zur Einheitsmatrix verhält.

a. Design: Konstanter Term + Group
Innersubjektdesign: Zeit

b. Kann zum Korrigieren der Freiheitsgrade für die gemittelten Signifikanztests verwendet werden. In der Tabelle mit den Tests der Effekte innerhalb der Subjekte werden korrigierte Tests angezeigt.

Tests der Innersubjekteffekte

Maß: MASS_1

Quelle		Typ III Quadratsumme	df	Mittel der Quadrate	F	Sig.	Partielles Eta-Quadrat
Zeit	Sphärizität angenommen	2,103	2	1,052	9,373	,001	,460
	Greenhouse-Geisser	2,103	1,140	1,845	9,373	,008	,460
	Huynh-Feldt (HF)	2,103	1,416	1,485	9,373	,004	,460
	Untergrenze	2,103	1,000	2,103	9,373	,011	,460
Zeit * Group	Sphärizität angenommen	,673	4	,168	1,499	,237	,214
	Greenhouse-Geisser	,673	2,281	,295	1,499	,262	,214
	Huynh-Feldt (HF)	,673	2,833	,237	1,499	,254	,214
	Untergrenze	,673	2,000	,336	1,499	,266	,214
Fehler(Zeit)	Sphärizität angenommen	2,469	22	,112			
	Greenhouse-Geisser	2,469	12,543	,197			
	Huynh-Feldt (HF)	2,469	15,579	,158			
	Untergrenze	2,469	11,000	,224			

Tests der Zwischensubjekteffekte

Maß: MASS_1

Transformierte Variable: Mittel

Quelle	Typ III Quadratsumme	df	Mittel der Quadrate	F	Sig.	Partielles Eta-Quadrat
Konstanter Term	13,193	1	13,193	121,762	,000	,917
Group	1,748	2	,874	8,066	,007	,595
Fehler	1,192	11	,108			

Paarweise Vergleiche

Maß: MASS_1

(I) Group	(J) Group	Mittelwertdifferenz (I-J)	Std.-Fehler	Sig.[b]	95% Konfidenzintervall für Differenz[b]	
					Untergrenze	Obergrenze
LD	MD	-,002	,134	1,000	-,381	,377
	HD	-,413*	,123	,019	-,759	-,067
MD	LD	,002	,134	1,000	-,377	,381
	HD	-,411*	,123	,019	-,757	-,065
HD	LD	,413*	,123	,019	,067	,759
	MD	,411*	,123	,019	,065	,757

Basiert auf geschätzten Randmitteln

*. Die Mittelwertdifferenz ist in Stufe ,05 signifikant.

b. Anpassung für Mehrfachvergleiche: Bonferroni.

Paarweise Vergleiche

Maß: MASS_1

(I) Zeit	(J) Zeit	Mittelwertdifferenz (I-J)	Std.-Fehler	Sig.[b]	95% Konfidenzintervall für Differenz[b]	
					Untergrenze	Obergrenze
1	2	-,554*	,145	,008	-,962	-,146
	3	-,219*	,050	,004	-,361	-,077
2	1	,554*	,145	,008	,146	,962
	3	,335	,163	,191	-,123	,794
3	1	,219*	,050	,004	,077	,361
	2	-,335	,163	,191	-,794	,123

Basiert auf geschätzten Randmitteln

*. Die Mittelwertdifferenz ist in Stufe ,05 signifikant.

b. Anpassung für Mehrfachvergleiche: Bonferroni.

Studie E

GLOB male

Da der Mauchly-Test nicht berechnet werden konnte, werden robuste Schätzer zu Beurteilung der Effekte verwendet. Es wird die Untergrenze als Schätzung benutzt.

Mauchly-Test auf Sphärizität

Maß: MASS_1

Innersubjekteffekt	Mauchly-W	Ungefähres Chi-Quadrat	df	Sig.	Epsilon		
					Greenhouse-Geisser	Huynh-Feldt (HF)	Untergrenze
Zeit	,000	.	14	.	,433	1,000	,200

Dabei zeigt sich ein signifikanter Haupteffekt Zeit, $F(1,3)P = 21{,}07, p = \ 0{,}019, \eta^2 = 0{,}88$. Der Effekt ist stark. Weiterhin zeigt sich eine nicht-signifikante Interaktion, $F(1,3) = 3{,}9, p = 0{,}143, \eta^2 = 0{,}57$.

Tests der Innersubjekteffekte

Maß: MASS_1

Quelle		Typ III Quadratsumme	df	Mittel der Quadrate	F	Sig.	Partielles Eta-Quadrat
Zeit	Sphärizität angenommen	502,042	5	100,408	21,069	,000	,875
	Greenhouse-Geisser	502,042	2,163	232,125	21,069	,001	,875
	Huynh-Feldt (HF)	502,042	5,000	100,408	21,069	,000	,875

	Untergrenze	502,042	1,000	502,042	21,069	,019	,875
Zeit * Group	Sphärizität angenommen	92,923	5	18,585	3,900	,018	,565
	Greenhouse-Geisser	92,923	2,163	42,964	3,900	,075	,565
	Huynh-Feldt (HF)	92,923	5,000	18,585	3,900	,018	,565
	Untergrenze	92,923	1,000	92,923	3,900	,143	,565
Fehler(Zeit)	Sphärizität angenommen	71,484	15	4,766			
	Greenhouse-Geisser	71,484	6,488	11,017			
	Huynh-Feldt (HF)	71,484	15,000	4,766			
	Untergrenze	71,484	3,000	23,828			

Der Haupteffekt Gruppe hat einen signifikanten Effekt, $F(1,3) = 21{,}73, p = 0{,}019, \eta^2 = 0{,}88$. Der Effekt ist stark.

Tests der Zwischensubjekteffekte

Maß: MASS_1

Transformierte Variable: Mittel

Quelle	Typ III Quadratsumme	df	Mittel der Quadrate	F	Sig.	Partielles Eta-Quadrat
Konstanter Term	36235,585	1	36235,585	1327,621	,000	,998
Group	592,961	1	592,961	21,725	,019	,879
Fehler	81,881	3	27,294			

Auf Grund von signifikanten Unterschieden in der Zeit, wird nun paarweise geschaut, welcher zwei Zeitpunkte sich signifikant unterscheiden. Dabei zeigten sich nur zwischen DSNG16 und DSNG36 signifikante Unterschiede, p = 0,044.

Paarweise Vergleiche

Maß: MASS_1

(I) Zeit	(J) Zeit	Mittelwertdifferenz (I-J)	Std.-Fehler	Sig.	95% Konfidenzintervall für Differenz: Untergrenze	95% Konfidenzintervall für Differenz: Obergrenze
1	2	-6,350	1,321	,257	-17,676	4,976
	3	-9,750	2,179	,312	-28,434	8,934
	4	-8,917	1,287	,092	-19,952	2,118
	5	-12,392	1,763	,089	-27,509	2,725
	6	-11,867	1,818	,110	-27,458	3,725
2	1	6,350	1,321	,257	-4,976	17,676
	3	-3,400	1,761	1,000	-18,505	11,705
	4	-2,567	,346	,076	-5,536	,403
	5	-6,042	,675	,044	-11,826	-,258
	6	-5,517	,744	,076	-11,895	,862
3	1	9,750	2,179	,312	-8,934	28,434
	2	3,400	1,761	1,000	-11,705	18,505

	4	,833	1,432	1,000	-11,450	13,117
	5	-2,642	1,931	1,000	-19,200	13,917
	6	-2,117	1,913	1,000	-18,522	14,289
4	1	8,917	1,287	,092	-2,118	19,952
	2	2,567	,346	,076	-,403	5,536
	3	-,833	1,432	1,000	-13,117	11,450
	5	-3,475	,856	,404	-10,813	3,863
	6	-2,950	,901	,700	-10,680	4,780
5	1	12,392	1,763	,089	-2,725	27,509
	2	6,042	,675	,044	,258	11,826
	3	2,642	1,931	1,000	-13,917	19,200
	4	3,475	,856	,404	-3,863	10,813
	6	,525	,085	,130	-,207	1,257
6	1	11,867	1,818	,110	-3,725	27,458
	2	5,517	,744	,076	-,862	11,895
	3	2,117	1,913	1,000	-14,289	18,522
	4	2,950	,901	,700	-4,780	10,680
	5	-,525	,085	,130	-1,257	,207

Kommende Grafik zeigt die Unterschiede zwischen den Gruppen im zeitlichen Verlauf. Die Mittelwerte in DG2 sind über die gesamte Zeit höher. Weiterhin steigen die Mittelwerte in beiden Gruppen an. Wobei sie in der DG2 ab DSNG60 leicht rückläufig sind.

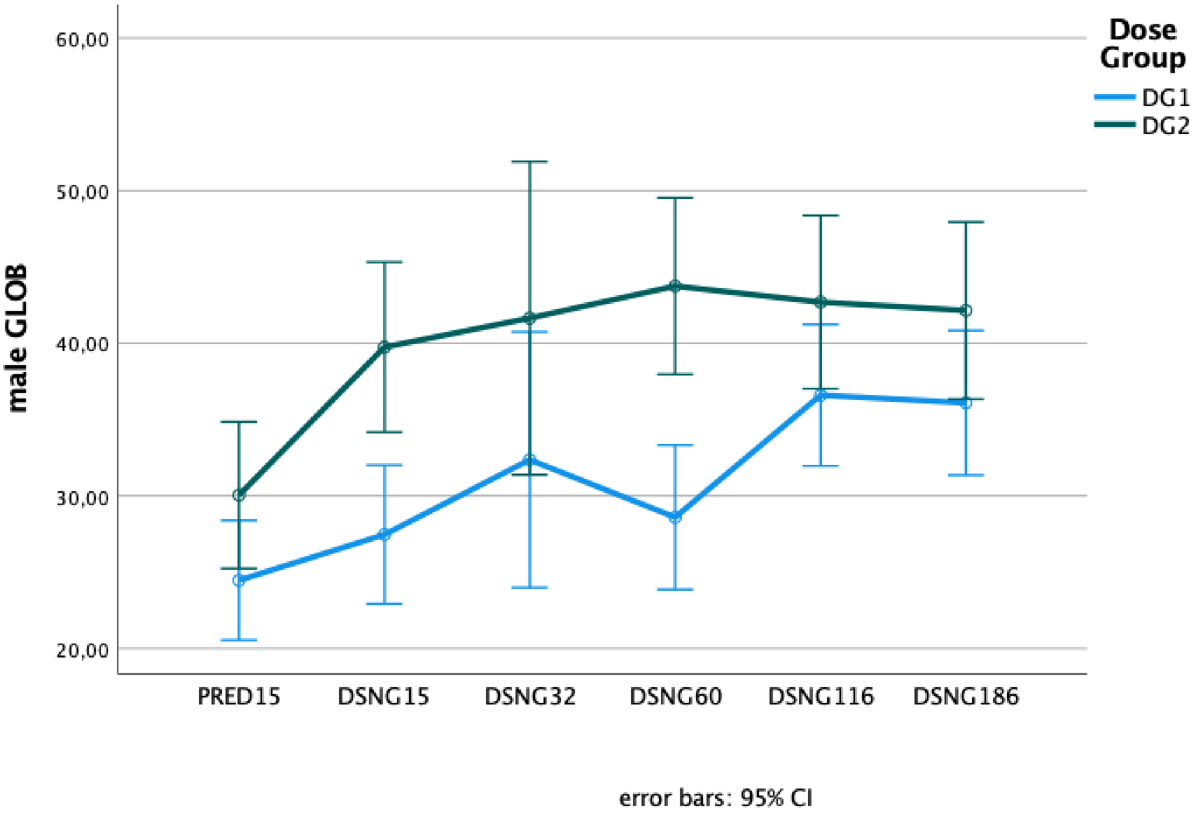

A:G male

Auch hier konnte der Mauchly-Test nicht berechnet werden. Somit werden die robusten Ergebnisse der Untergrenze betrachtet.

Mauchly-Test auf Sphärizität

Maß: MASS_1

Innersubjekteffekt	Mauchly-W	Ungefähres Chi-Quadrat	df	Sig.	Epsilon		
					Greenhouse-Geisser	Huynh-Feldt (HF)	Untergrenze
Zeit	,000	.	14	.	,441	1,000	,200

Dabei zeigt sich ein signifikanter Haupteffekt Zeit, $F(1,3) = 20{,}93, p = 0{,}020,\ \eta^2 = 0{,}88$. Der Effekt ist stark. Die Interaktion war jedoch nicht signifikant, $F(1,3) = 4{,}30, p = 0{,}130, \eta^2 = 0{,}59$. Der Effekt der Interaktion ist stark.

Tests der Innersubjekteffekte

Maß: MASS_1

Quelle		Typ III Quadratsumme	df	Mittel der Quadrate	F	Sig.	Partielles Eta-Quadrat
Zeit	Sphärizität angenommen	2,648	5	,530	20,929	,000	,875
	Greenhouse-Geisser	2,648	2,203	1,202	20,929	,001	,875
	Huynh-Feldt (HF)	2,648	5,000	,530	20,929	,000	,875
	Untergrenze	2,648	1,000	2,648	20,929	,020	,875
Zeit * Group	Sphärizität angenommen	,544	5	,109	4,299	,013	,589
	Greenhouse-Geisser	,544	2,203	,247	4,299	,061	,589
	Huynh-Feldt (HF)	,544	5,000	,109	4,299	,013	,589
	Untergrenze	,544	1,000	,544	4,299	,130	,589
Fehler(Zeit)	Sphärizität angenommen	,379	15	,025			
	Greenhouse-Geisser	,379	6,608	,057			
	Huynh-Feldt (HF)	,379	15,000	,025			
	Untergrenze	,379	3,000	,126			

Weiterhin ist der Haupteffekt Gruppe signifikant, $F(1,3) = 22{,}66, p =\ 0{,}018, \eta^2 = 0{,}88$. Der Effekt ist stark.

Tests der Zwischensubjekteffekte

Maß: MASS_1

Transformierte Variable: Mittel

Quelle	Typ III Quadratsumme	df	Mittel der Quadrate	F	Sig.	Partielles Eta-Quadrat
Konstanter Term	55,189	1	55,189	476,795	,000	,994
Group	2,623	1	2,623	22,663	,018	,883
Fehler	,347	3	,116			

Kommende Grafik zeigt die geschätzten Mittelwerte visuell. Dabei sind die Mittelwerte der DG1 über die Zeit höher als in DG2. In beiden Gruppen fallen die Mittelwerte über die Zeit. Ab DSNG60 scheinen die Mittelwerte in der DG2 zu stagnieren. Am Ende des Beobachtungszeitraums sind die Mittelwerte etwa anglichen. Eine Stagnation ist in der DG1 ab DSNG116 zuerkennen.

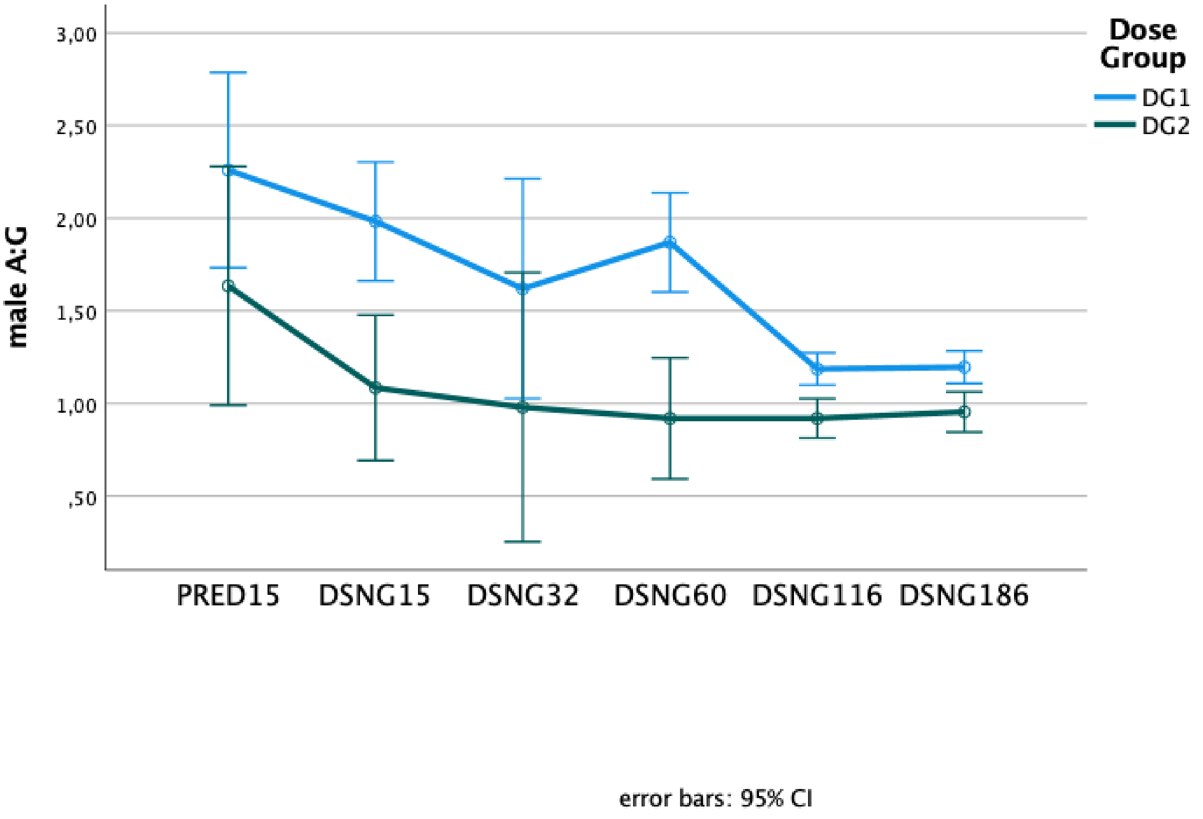

Studie H

APL female

Im Weiteren wird nun der female ALP untersucht. Es zeigt sich, dass die Annahme der Sphärizität verletzt ist. Der Mauchly-Test liefert ein signifikantes Ergebnis, $\chi^2(5) = 16{,}25, p = 0{,}006$. Somit werden die Effekte mittels der robusten Untergrenze geschätzt.

Mauchly-Test auf Sphärizität

Maß: MASS_1

Innersubjekteffekt	Mauchly-W	Ungefähres Chi-Quadrat	df	Sig.	Epsilon: Greenhouse-Geisser	Epsilon: Huynh-Feldt (HF)	Epsilon: Untergrenze
Zeit	,279	16,245	5	,006	,589	,811	,333

Es zeigt sich dabei ein nicht-signifikanter Haupteffekt Zeit, $F(1,14) = 2{,}68, p = 0{,}124, \eta^2 = 0{,}16$. Der Effekt ist stark. Weiterhin ist die Interaktion nicht-signifikant, $F(3,14) = 2{,}72, p = 0{,}084, \eta^2 = 0{,}37.$ Der Effekt ist stark.

Tests der Innersubjekteffekte

Maß: MASS_1

Quelle		Typ III Quadratsumme	df	Mittel der Quadrate	F	Sig.	Partielles Eta-Quadrat
Zeit	Sphärizität angenommen	101665,828	3	33888,609	2,684	,059	,161
	Greenhouse-Geisser	101665,828	1,766	57582,431	2,684	,094	,161
	Huynh-Feldt (HF)	101665,828	2,434	41766,701	2,684	,073	,161
	Untergrenze	101665,828	1,000	101665,828	2,684	,124	,161
Zeit * Group	Sphärizität angenommen	309090,031	9	34343,337	2,720	,014	,368
	Greenhouse-Geisser	309090,031	5,297	58355,088	2,720	,041	,368
	Huynh-Feldt (HF)	309090,031	7,302	42327,139	2,720	,022	,368
	Untergrenze	309090,031	3,000	103030,010	2,720	,084	,368
Fehler(Zeit)	Sphärizität angenommen	530369,583	42	12627,847			
	Greenhouse-Geisser	530369,583	24,718	21456,830			
	Huynh-Feldt (HF)	530369,583	34,078	15563,445			
	Untergrenze	530369,583	14,000	37883,542			

Im Falle des Haupteffekts Gruppe zeigt sich ein nicht-signifikantes Ergebnis, $F(3,14) = 2{,}59, p = 0{,}094, \eta^2 = 0{,}36$. Der Effekt ist stark.

Tests der Zwischensubjekteffekte

Maß: MASS_1

Transformierte Variable: Mittel

Quelle	Typ III Quadratsumme	df	Mittel der Quadrate	F	Sig.	Partielles Eta-Quadrat
Konstanter Term	55920739,220	1	55920739,220	283,577	,000	,953
Group	1534138,748	3	511379,583	2,593	,094	,357
Fehler	2760772,896	14	197198,064			

Kommende Grafik zeigt die Mittelwerte nach Gruppe und Zeit. HD hat dabei über die Zeit die höchsten Mittelwerte. LD-PD hat über die meiste Zeit den niedrigsten. Weiterhin liegt der Mittelwert von LD zu Beginn über MD. ZU DSNG174 ist dies umgekehrt.

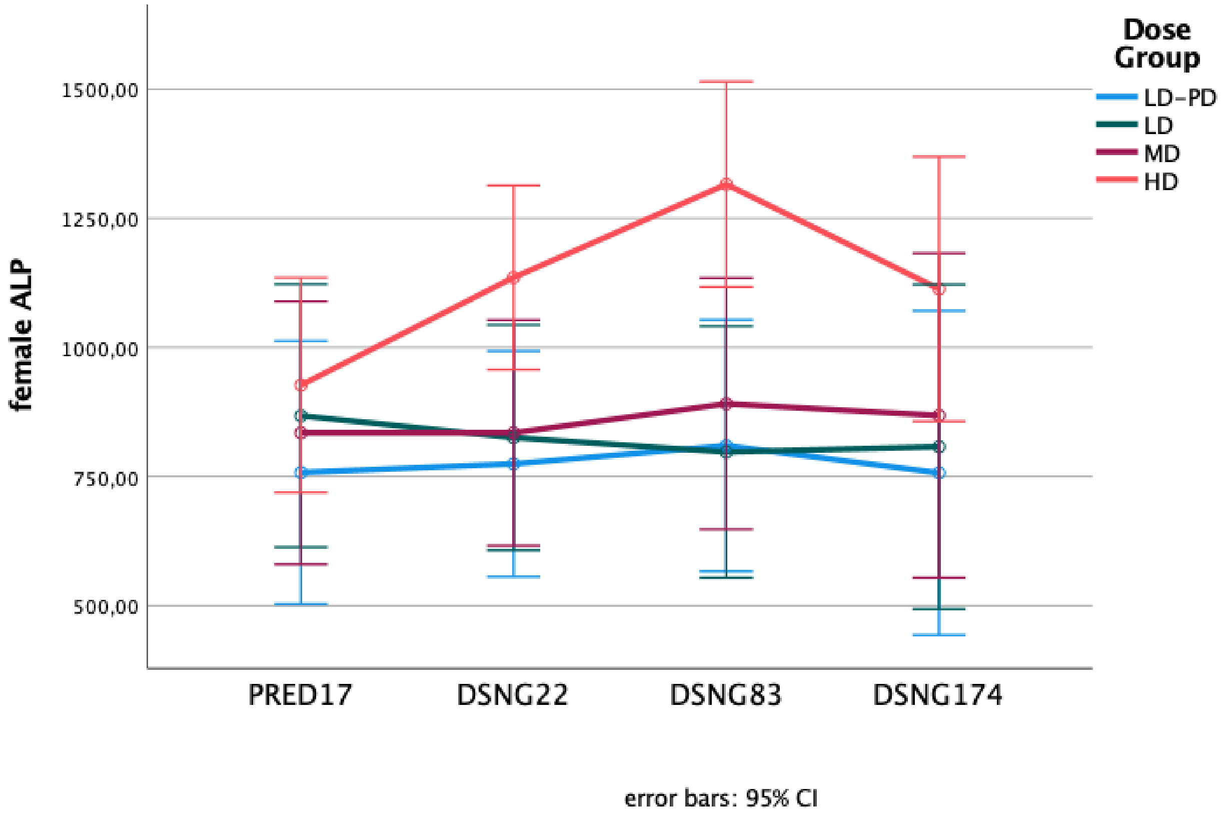

Studie L

GLOB female

Im Falle von GLOB female liefert der Mauchly-Test ein nicht-signifikantes Ergebnis, $\chi^2(20) = 22{,}76, p = 0{,}332$. Damit ist die Sphärizitätsannahme erfüllt.

Mauchly-Test auf Sphärizität

Maß: MASS_1

Innersubjekteffekt	Mauchly-W	Ungefähres Chi-Quadrat	df	Sig.	Epsilon Greenhouse-Geisser	Epsilon Huynh-Feldt (HF)	Epsilon Untergrenze
Zeit	,075	22,755	20	,332	,536	,922	,167

Hierbei zeigt sich ein signifikanter Haupteffekt Zeit, $F(6, 66) = 16{,}27, p = 0{,}000, \eta^2 = 0{,}60$. Der Effekt ist stark. Die Interaktion übt hingegen keinen signifikanten Einfluss aus, $F(12, 66) = 1{,}13, p = 0{,}352, \eta^2 = 0{,}17$. Der Effekt ist stark.

Tests der Innersubjekteffekte

Maß: MASS_1

Quelle		Typ III Quadratsumme	df	Mittel der Quadrate	F	Sig.	Partielles Eta-Quadrat
Zeit	Sphärizität angenommen	254,202	6	42,367	16,267	,000	,597
	Greenhouse-Geisser	254,202	3,217	79,019	16,267	,000	,597
	Huynh-Feldt (HF)	254,202	5,530	45,970	16,267	,000	,597
	Untergrenze	254,202	1,000	254,202	16,267	,002	,597
Zeit * Group	Sphärizität angenommen	35,305	12	2,942	1,130	,352	,170
	Greenhouse-Geisser	35,305	6,434	5,487	1,130	,366	,170
	Huynh-Feldt (HF)	35,305	11,059	3,192	1,130	,355	,170
	Untergrenze	35,305	2,000	17,652	1,130	,358	,170
Fehler(Zeit)	Sphärizität angenommen	171,896	66	2,604			
	Greenhouse-Geisser	171,896	35,387	4,858			
	Huynh-Feldt (HF)	171,896	60,827	2,826			
	Untergrenze	171,896	11,000	15,627			

Der Haupteffekt Gruppe ist ebenfalls nicht-signifikant, $F(2, 11) = 0{,}08, p = 0{,}920, \eta^2 = 0{,}02$. Der Effekt ist schwach.

Tests der Zwischensubjekteffekte

Maß: MASS_1

Transformierte Variable: Mittel

Quelle	Typ III Quadratsumme	df	Mittel der Quadrate	F	Sig.	Partielles Eta-Quadrat
Konstanter Term	78174,857	1	78174,857	2137,761	,000	,995
Group	6,140	2	3,070	,084	,920	,015
Fehler	402,254	11	36,569			

Auf Grund eines signifikanten Haupteffekts Zeit wird eine Post-Hoc-Analyse durchgeführt. Es zeigt sich dabei das zwischen PRED21 vs. DSNG57, PRED21 vs. DSNG281, DSNG57 vs. DSNG253, DSNG141 vs. DSNG253, DSNG197 vs. DSNG253, DSNG197 vs. DSNG281 und DSNG253 vs. DSNG281 signifikante Unterschiede vorliegen.

Paarweise Vergleiche

Maß: MASS_1

(I) Zeit	(J) Zeit	Mittelwertdifferenz (I-J)	Std.-Fehler	Sig.	95% Konfidenzintervall für Differenz	
					Untergrenze	Obergrenze
1	2	2,364	,501	,013	,399	4,329
	3	1,325	,441	,252	-,406	3,056
	4	2,517	,846	,266	-,803	5,836
	5	1,256	,587	1,000	-1,048	3,559
	6	-1,636	,821	1,000	-4,857	1,585
	7	3,736	,551	,001	1,576	5,897
2	1	-2,364	,501	,013	-4,329	-,399
	3	-1,039	,398	,507	-2,599	,521
	4	,153	,630	1,000	-2,318	2,624
	5	-1,108	,495	,986	-3,052	,835
	6	-4,000	,739	,004	-6,900	-1,100
	7	1,372	,481	,331	-,515	3,260
3	1	-1,325	,441	,252	-3,056	,406
	2	1,039	,398	,507	-,521	2,599
	4	1,192	,757	1,000	-1,777	4,160
	5	-,069	,579	1,000	-2,342	2,203
	6	-2,961	,845	,104	-6,278	,355
	7	2,411	,649	,072	-,136	4,958
4	1	-2,517	,846	,266	-5,836	,803
	2	-,153	,630	1,000	-2,624	2,318
	3	-1,192	,757	1,000	-4,160	1,777
	5	-1,261	,692	1,000	-3,975	1,452
	6	-4,153	,626	,001	-6,609	-1,696
	7	1,219	,538	,938	-,892	3,331
5	1	-1,256	,587	1,000	-3,559	1,048
	2	1,108	,495	,986	-,835	3,052
	3	,069	,579	1,000	-2,203	2,342
	4	1,261	,692	1,000	-1,452	3,975
	6	-2,892	,632	,017	-5,373	-,411
	7	2,481	,434	,003	,780	4,182
6	1	1,636	,821	1,000	-1,585	4,857
	2	4,000	,739	,004	1,100	6,900
	3	2,961	,845	,104	-,355	6,278
	4	4,153	,626	,001	1,696	6,609
	5	2,892	,632	,017	,411	5,373
	7	5,372	,498	,000	3,420	7,325

7	1	-3,736	,551	,001	-5,897	-1,576
	2	-1,372	,481	,331	-3,260	,515
	3	-2,411	,649	,072	-4,958	,136
	4	-1,219	,538	,938	-3,331	,892
	5	-2,481	,434	,003	-4,182	-,780
	6	-5,372	,498	,000	-7,325	-3,420

Kommende Tabelle zeigt die Verläufe der Mittelwerte nach Dosisgruppe über die Zeit. Die Mittelwerte bei LD sind ab DSNG57 am höchsten. Ansonsten ist der Verlauf nach Gruppen sehr ähnlich.

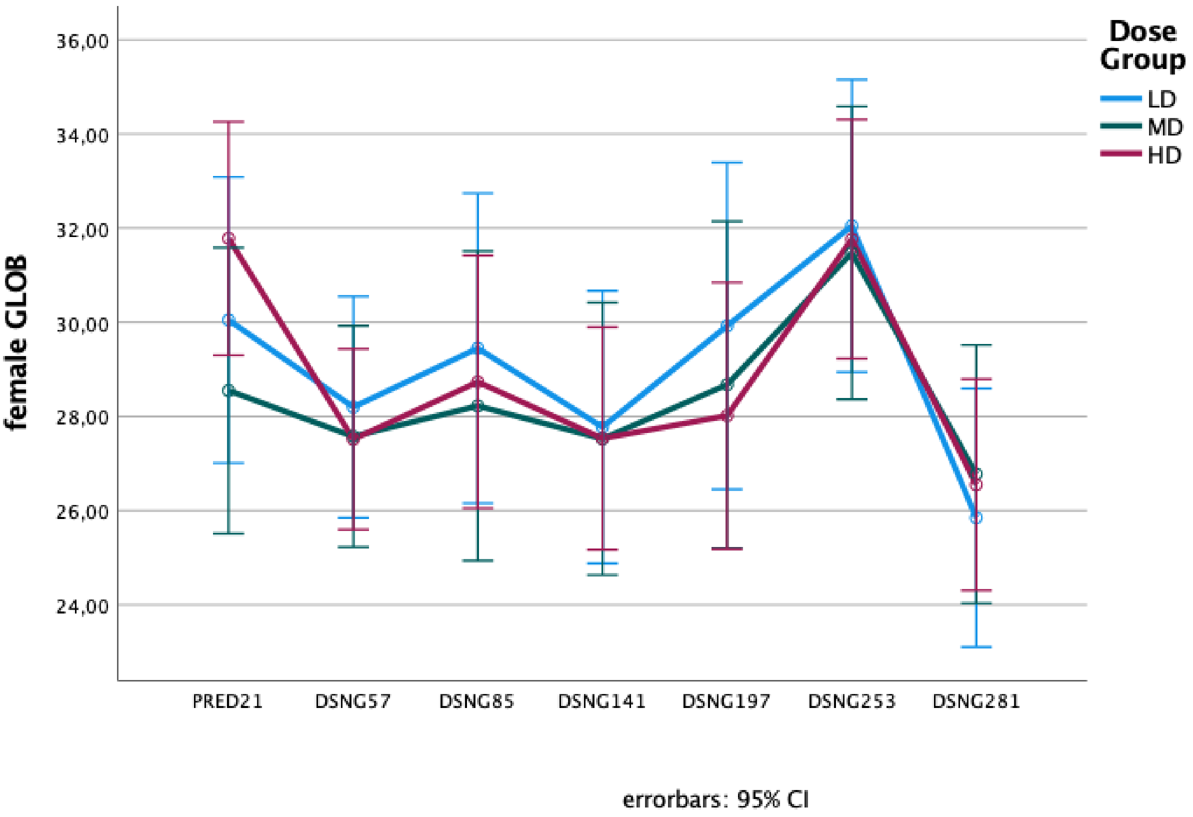

TP female

Bei der vorliegenden Analyse des TP zeigte sich ein nicht-signifikantes Ergebnis bei dem Mauchly-Test, $\chi^2(20) = 25{,}88, p = 0{,}195.$ Somit ist die Sphärizitätsannahme erfüllt.

Mauchly-Test auf Sphärizität

Maß: MASS_1

Innersubjekteffekt	Mauchly-W	Ungefähres Chi-Quadrat	df	Sig.	Epsilon: Greenhouse-Geisser	Epsilon: Huynh-Feldt (HF)	Epsilon: Untergrenze
Zeit	,052	25,884	20	,195	,500	,833	,167

Es zeigt sich hierbei ein signifikanter Haupteffekt Zeit, $F(6, 66) = 11{,}34, p = 0{,}000, \eta^2 = 0{,}51$. Der Effekt ist stark. Die Interaktion übt keinen signifikanten Effekt aus, $F(12, 66) = 0{,}43, p = 0{,}946, \eta^2 = 0{,}07$. Der Effekt ist mittelstark.

Tests der Innersubjekteffekte

Maß: MASS_1

Quelle		Typ III Quadratsumme	df	Mittel der Quadrate	F	Sig.	Partielles Eta-Quadrat
Zeit	Sphärizität angenommen	458,956	6	76,493	11,337	,000	,508
	Greenhouse-Geisser	458,956	2,998	153,080	11,337	,000	,508
	Huynh-Feldt (HF)	458,956	4,996	91,872	11,337	,000	,508
	Untergrenze	458,956	1,000	458,956	11,337	,006	,508
Zeit * Group	Sphärizität angenommen	34,856	12	2,905	,431	,946	,073
	Greenhouse-Geisser	34,856	5,996	5,813	,431	,853	,073
	Huynh-Feldt (HF)	34,856	9,991	3,489	,431	,925	,073
	Untergrenze	34,856	2,000	17,428	,431	,661	,073
Fehler(Zeit)	Sphärizität angenommen	445,294	66	6,747			
	Greenhouse-Geisser	445,294	32,980	13,502			
	Huynh-Feldt (HF)	445,294	54,952	8,103			
	Untergrenze	445,294	11,000	40,481			

Der Haupteffekt Gruppe übt keinen signifikanten Effekt auf TP aus, $F(2, 11) = 0{,}03, p = 0{,}968, \eta^2 < 0{,}01$. Der Effekt ist unbedeutsam.

Tests der Zwischensubjekteffekte

Maß: MASS_1

Transformierte Variable: Mittel

Quelle	Typ III Quadratsumme	df	Mittel der Quadrate	F	Sig.	Partielles Eta-Quadrat
Konstanter Term	440560,654	1	440560,654	4034,834	,000	,997
Group	7,149	2	3,575	,033	,968	,006
Fehler	1201,082	11	109,189			

Auf Grund des signifikanten Haupteffekts Zeit wird eine Post-Hoc-Analyse durchgeführt. Es zeigt sich dabei das zwischen PPRED21 vs. DSNG281, DSNG57 vs. DSNG281, DSNG141 vs. DSNG253, DSNG197 vs. DSNG281, und DSNG253 vs. DSNG281 lagen signifikante Unterschiede vor.

Paarweise Vergleiche

Maß: MASS_1

(I) Zeit	(J) Zeit	Mittelwertdifferenz (I-J)	Std.-Fehler	Sig.	95% Konfidenzintervall für Differenz: Untergrenze	95% Konfidenzintervall für Differenz: Obergrenze
1	2	3,022	,979	,218	-,820	6,864
	3	2,689	,815	,149	-,508	5,886
	4	4,806	1,258	,060	-,129	9,740
	5	2,911	,910	,178	-,659	6,481
	6	-,414	1,504	1,000	-6,315	5,487
	7	6,228	,961	,001	2,458	9,997
2	1	-3,022	,979	,218	-6,864	,820
	3	-,333	,566	1,000	-2,554	1,887
	4	1,783	,768	,850	-1,231	4,797
	5	-,111	,772	1,000	-3,141	2,919
	6	-3,436	1,314	,505	-8,592	1,720
	7	3,206	,799	,043	,072	6,339
3	1	-2,689	,815	,149	-5,886	,508
	2	,333	,566	1,000	-1,887	2,554
	4	2,117	1,015	1,000	-1,865	6,099
	5	,222	,936	1,000	-3,449	3,894
	6	-3,103	1,459	1,000	-8,828	2,622
	7	3,539	1,027	,115	-,488	7,566
4	1	-4,806	1,258	,060	-9,740	,129
	2	-1,783	,768	,850	-4,797	1,231
	3	-2,117	1,015	1,000	-6,099	1,865
	5	-1,894	,799	,780	-5,030	1,241
	6	-5,219	,957	,004	-8,972	-1,467
	7	1,422	,875	1,000	-2,011	4,855
5	1	-2,911	,910	,178	-6,481	,659
	2	,111	,772	1,000	-2,919	3,141
	3	-,222	,936	1,000	-3,894	3,449
	4	1,894	,799	,780	-1,241	5,030
	6	-3,325	,945	,101	-7,034	,384
	7	3,317	,646	,007	,782	5,852
6	1	,414	1,504	1,000	-5,487	6,315
	2	3,436	1,314	,505	-1,720	8,592
	3	3,103	1,459	1,000	-2,622	8,828
	4	5,219	,957	,004	1,467	8,972

	5	3,325	,945	,101	-,384	7,034
	7	6,642	1,085	,002	2,387	10,896
7	1	-6,228	,961	,001	-9,997	-2,458
	2	-3,206	,799	,043	-6,339	-,072
	3	-3,539	1,027	,115	-7,566	,488
	4	-1,422	,875	1,000	-4,855	2,011
	5	-3,317	,646	,007	-5,852	-,782
	6	-6,642	1,085	,002	-10,896	-2,387

Kommende Grafik zeigt die Verläufe der Mittelwerte zwischen den Gruppen über die Zeit. Es zeigt sich, dass zu Beginn HD den höchsten Mittelwert aufweist. Zum Ende des Versuchs hat HD den niedrigsten. Der Mittelwert von LD ist zu Beginn am niedrigsten. Zum Ende hat diese Gruppe den zweithöchsten. Die Gruppe MD weist zu Beginn den zweithöchsten Mittelwert auf. Zum Ende den höchsten. Insgesamt sind die Verläufe der Gruppen sehr ähnlich.

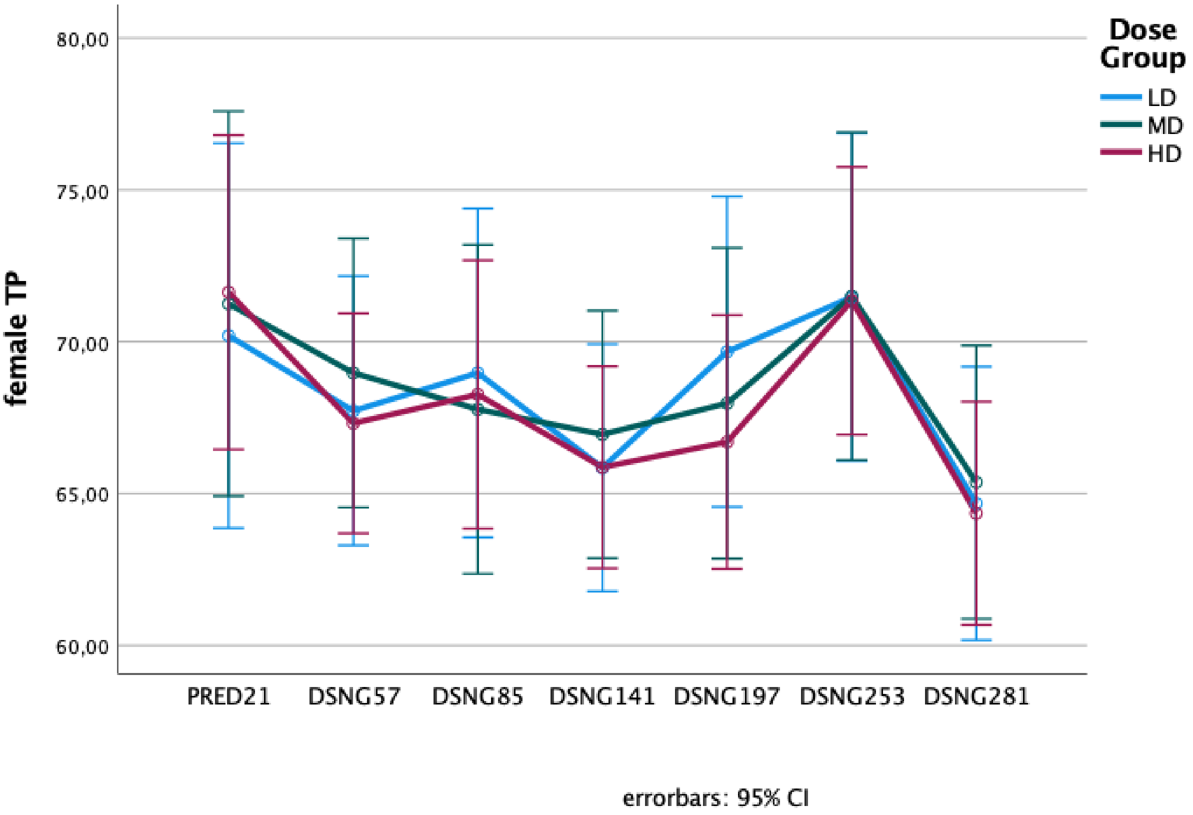

Studie M
IGG male

Im Falle von IgG male zeigte sich ein nicht-signifikantes Ergebnis bei dem Mauchly-Test, $\chi^2(9) = 12{,}72, p = 0{,}222$. Somit ist die Sphärizitätsannahme erfüllt.

Mauchly-Test auf Sphärizität

Maß: MASS_1

Innersubjekteffekt	Mauchly-W	Ungefähres Chi-	df	Sig.	Epsilon

		Quadrat			Greenhouse-Geisser	Huynh-Feldt (HF)	Untergrenze
Zeit	,024	12,723	9	,222	,385	,522	,250

Dabei zeigt sich ein signifikanter Haupteffekt Zeit, $F(4,20) = 94{,}88, p = 0{,}000, \eta^2 = 0{,}95$. Der Effekt ist stark.

Tests der Innersubjekteffekte

Maß: MASS_1

Quelle		Typ III Quadratsumme	df	Mittel der Quadrate	F	Sig.	Partielles Eta-Quadrat
Zeit	Sphärizität angenommen	228,388	4	57,097	94,884	,000	,950
	Greenhouse-Geisser	228,388	1,539	148,439	94,884	,000	,950
	Huynh-Feldt (HF)	228,388	2,089	109,318	94,884	,000	,950
	Untergrenze	228,388	1,000	228,388	94,884	,000	,950
Fehler(Zeit)	Sphärizität angenommen	12,035	20	,602			
	Greenhouse-Geisser	12,035	7,693	1,564			
	Huynh-Feldt (HF)	12,035	10,446	1,152			
	Untergrenze	12,035	5,000	2,407			

Da der Haupteffekt signifikant war, werden die paarweisen Vergleiche innerhalb einer Post-Hoc-Analyse untersucht. Es zeigt sich, dass zwischen PRED50 vs. allen übrigen Zeitpunkten, DSNG31 vs. DSNG86, DSNG31 vs. DSNG176, DSNG86 vs. DSNG136, DSNG86 vs. DSNG176 und DSNG136 vs. DSNG176 signifikante Unterschiede vorliegen.

Paarweise Vergleiche

Maß: MASS_1

(I) Zeit	(J) Zeit	Mittelwertdifferenz (I-J)	Std.-Fehler	Sig.	95% Konfidenzintervall für Differenz Untergrenze	Obergrenze
1	2	7,687	,759	,002	4,065	11,308
	3	4,865	,552	,003	2,232	7,498
	4	7,285	,596	,001	4,442	10,128
	5	5,835	,549	,001	3,216	8,454
2	1	-7,687	,759	,002	-11,308	-4,065
	3	-2,822	,406	,009	-4,759	-,884
	4	-,402	,264	1,000	-1,664	,861
	5	-1,852	,338	,028	-3,467	-,236
3	1	-4,865	,552	,003	-7,498	-2,232
	2	2,822	,406	,009	,884	4,759
	4	2,420	,241	,002	1,270	3,570
	5	,970	,154	,015	,234	1,706
4	1	-7,285	,596	,001	-10,128	-4,442

	2	,402	,264	1,000	-,861	1,664
	3	-2,420	,241	,002	-3,570	-1,270
	5	-1,450	,198	,007	-2,394	-,506
5	1	-5,835	,549	,001	-8,454	-3,216
	2	1,852	,338	,028	,236	3,467
	3	-,970	,154	,015	-1,706	-,234
	4	1,450	,198	,007	,506	2,394

Kommende Grafik zeigt die geschätzten Mittelwerte. Es zeigt sich, dass der Mittelwert von PRED50 zu DSNG31 fällt. Danach schwankt er, jedoch bleibt der Mittelwert über die übrigen Zeitpunkte etwa auf einem Niveau.

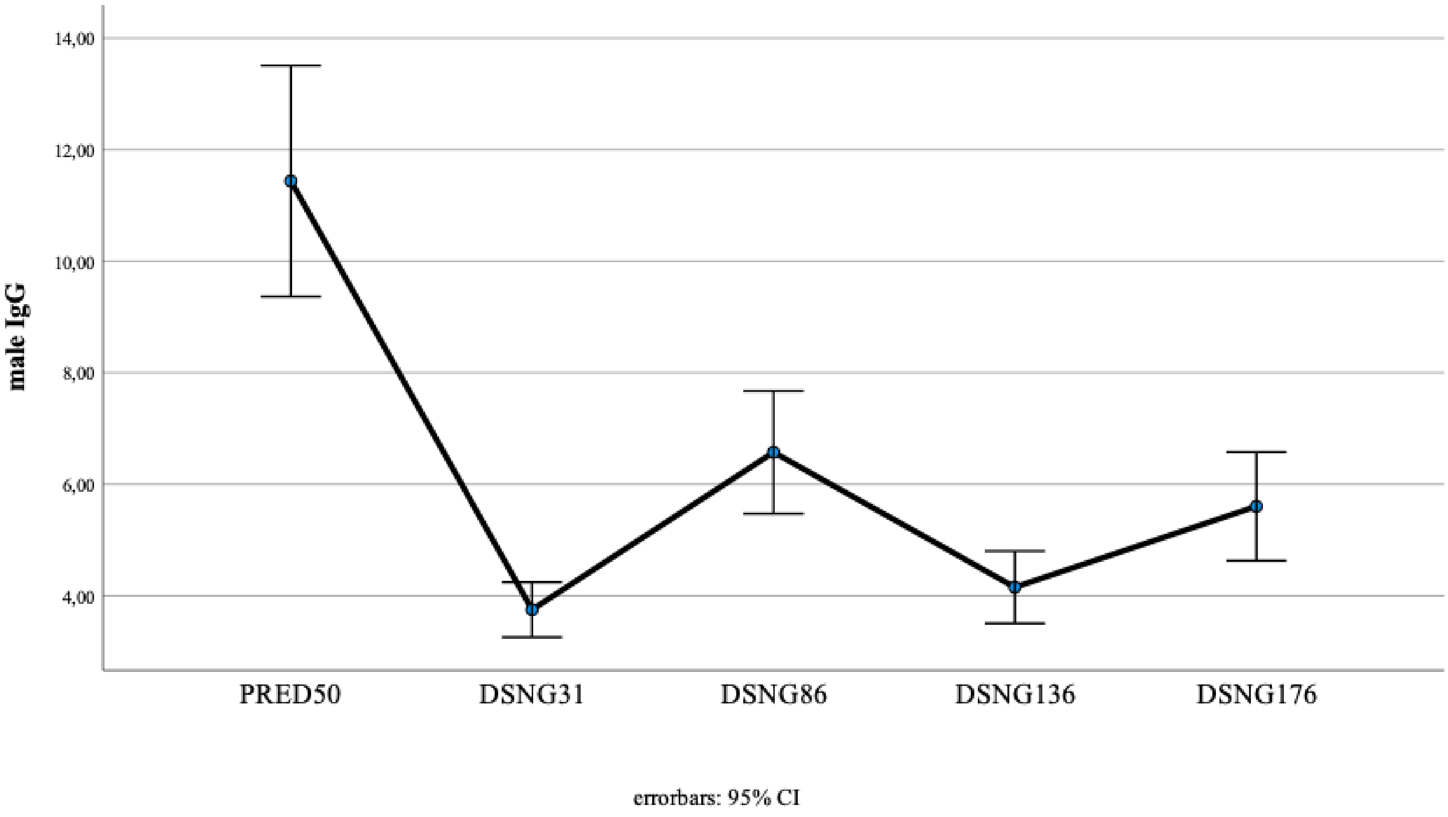

TP male

Im Falle von TP zeigte sich ein nicht-signifikantes Ergebnis bei dem Mauchly-Test, $\chi^2(9) = 14{,}21, p = 0{,}154$. Somit ist die Sphärizität gegeben.

Mauchly-Test auf Sphärizität

Maß: MASS_1

Innersubjekteffekt	Mauchly-W	Ungefähres Chi-	df	Sig.	Epsilon

		Quadrat			Greenhouse-Geisser	Huynh-Feldt (HF)	Untergrenze
Zeit	,016	14,206	9	,154	,512	,870	,250

Zwischen den Zeitpunkten zeigten sich signifikante Unterschiede, $F(4, 20) = 36{,}31, p = 0{,}000, \eta^2 = 0{,}88$. Der Effekt ist somit stark.

Tests der Innersubjekteffekte

Maß: MASS_1

Quelle		Typ III Quadratsumme	df	Mittel der Quadrate	F	Sig.	Partielles Eta-Quadrat
Zeit	Sphärizität angenommen	706,066	4	176,517	36,308	,000	,879
	Greenhouse-Geisser	706,066	2,047	344,932	36,308	,000	,879
	Huynh-Feldt (HF)	706,066	3,482	202,788	36,308	,000	,879
	Untergrenze	706,066	1,000	706,066	36,308	,002	,879
Fehler(Zeit)	Sphärizität angenommen	97,233	20	4,862			
	Greenhouse-Geisser	97,233	10,235	9,500			
	Huynh-Feldt (HF)	97,233	17,409	5,585			
	Untergrenze	97,233	5,000	19,447			

Da ein signifikante Haupteffekt vorliegt, werden die paarweisen Vergleiche berechnet. Dabei zeigt sich, dass sich nur PRED50 von allen übrigen Zeitpunkten signifikant unterscheidet.

Paarweise Vergleiche

Maß: MASS_1

(I) Zeit	(J) Zeit	Mittelwertdifferenz (I-J)	Std.-Fehler	Sig.	95% Konfidenzintervall für Differenz	
					Untergrenze	Obergrenze
1	2	12,297	1,467	,004	5,296	19,298
	3	12,318	1,893	,013	3,281	21,356
	4	12,648	,941	,000	8,158	17,139
	5	10,852	1,033	,001	5,923	15,780
2	1	-12,297	1,467	,004	-19,298	-5,296
	3	,022	1,829	1,000	-8,710	8,753
	4	,352	,963	1,000	-4,244	4,948
	5	-1,445	,843	1,000	-5,470	2,580
3	1	-12,318	1,893	,013	-21,356	-3,281
	2	-,022	1,829	1,000	-8,753	8,710
	4	,330	1,146	1,000	-5,139	5,799
	5	-1,467	1,386	1,000	-8,084	5,150
4	1	-12,648	,941	,000	-17,139	-8,158

	2	-,352	,963	1,000	-4,948	4,244
	3	-,330	1,146	1,000	-5,799	5,139
	5	-1,797	,548	,219	-4,411	,818
5	1	-10,852	1,033	,001	-15,780	-5,923
	2	1,445	,843	1,000	-2,580	5,470
	3	1,467	1,386	1,000	-5,150	8,084
	4	1,797	,548	,219	-,818	4,411

Kommende Grafik zeigt die geschätzten Mittelwerte. Es zeigt sich, dass der Mittelwert von PRED50 zu DSNG31 fällt. Danach schwankt er, jedoch bleibt der Mittelwert über die übrigen Zeitpunkte etwa auf einem Niveau.

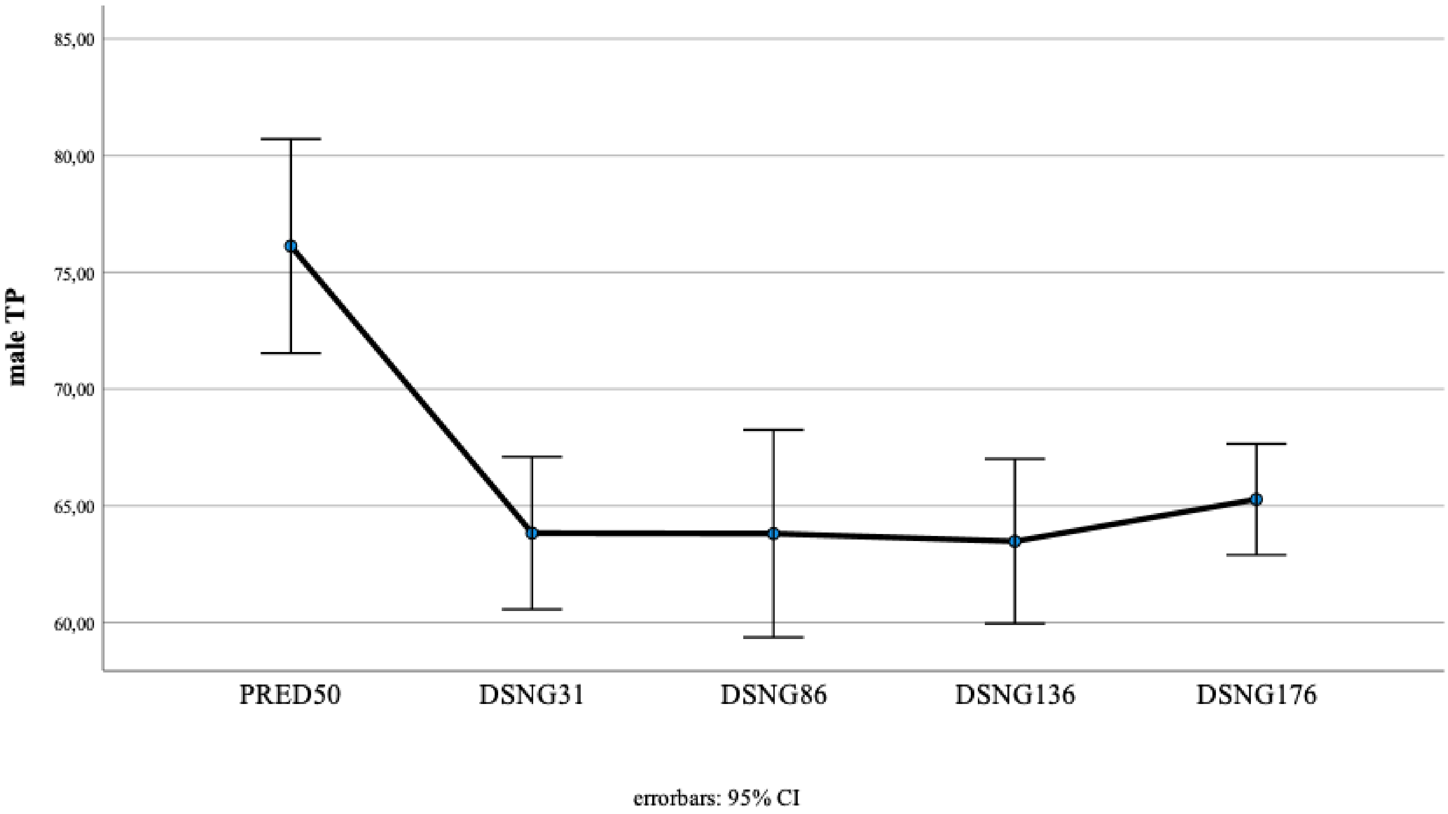

GLOB male

Hier liefert der Mauchly-Test ein nicht-signifikantes Ergebnis, $\chi^2(9) = 14{,}20, p = 0{,}154$. Damit ist die Sphärizitätsannahme erfüllt.

Mauchly-Test auf Sphärizität

Maß: MASS_1

					Epsilon		
Innersubjekteffekt	Mauchly-W	Ungefähres Chi-Quadrat	df	Sig.	Greenhouse-Geisser	Huynh-Feldt (HF)	Untergrenze

Zeit	,016	14,198	9	,154	,424	,619	,250

Der Haupteffekt Zeit hat dabei einen signifikanten Einfluss, $F(4, 20) = 12{,}73, p = 0{,}000, \eta^2 = 0{,}72$. Der Effekt ist stark.

Tests der Innersubjekteffekte

Maß: MASS_1

Quelle		Typ III Quadratsumme	df	Mittel der Quadrate	F	Sig.	Partielles Eta-Quadrat
Zeit	Sphärizität angenommen	188,547	4	47,137	12,727	,000	,718
	Greenhouse-Geisser	188,547	1,697	111,133	12,727	,003	,718
	Huynh-Feldt (HF)	188,547	2,476	76,148	12,727	,001	,718
	Untergrenze	188,547	1,000	188,547	12,727	,016	,718
Fehler(Zeit)	Sphärizität angenommen	74,075	20	3,704			
	Greenhouse-Geisser	74,075	8,483	8,732			
	Huynh-Feldt (HF)	74,075	12,380	5,983			
	Untergrenze	74,075	5,000	14,815			

Auf Grund des signifikanten Haupteffekts erfolgt eine Post-Hoc-Analyse. Hierbei war nur der Vergleich DSNG136 vs. DSNG176 signifikant.

Paarweise Vergleiche

Maß: MASS_1

(I) Zeit	(J) Zeit	Mittelwertdifferenz (I-J)	Std.-Fehler	Sig.	95% Konfidenzintervall für Differenz Untergrenze	Obergrenze
1	2	7,350	1,815	,098	-1,312	16,012
	3	4,122	1,352	,285	-2,333	10,576
	4	6,212	1,381	,064	-,381	12,804
	5	4,493	1,366	,217	-2,027	11,013
2	1	-7,350	1,815	,098	-16,012	1,312
	3	-3,228	1,195	,427	-8,933	2,477
	4	-1,138	,739	1,000	-4,666	2,389
	5	-2,857	,623	,059	-5,832	,119
3	1	-4,122	1,352	,285	-10,576	2,333
	2	3,228	1,195	,427	-2,477	8,933
	4	2,090	,707	,317	-1,285	5,465
	5	,372	,709	1,000	-3,013	3,756
4	1	-6,212	1,381	,064	-12,804	,381

	2	1,138	,739	1,000	-2,389	4,666
	3	-2,090	,707	,317	-5,465	1,285
	5	-1,718	,292	,020	-3,114	-,322
5	1	-4,493	1,366	,217	-11,013	2,027
	2	2,857	,623	,059	-,119	5,832
	3	-,372	,709	1,000	-3,756	3,013
	4	1,718	,292	,020	,322	3,114

Kommende Grafik zeigt die geschätzten Mittelwerte. Es zeigt sich, dass der Mittelwert von PRED50 zu DSNG31 fällt. Danach schwankt er, jedoch bleibt der Mittelwert über die übrigen Zeitpunkte etwa auf einem Niveau.

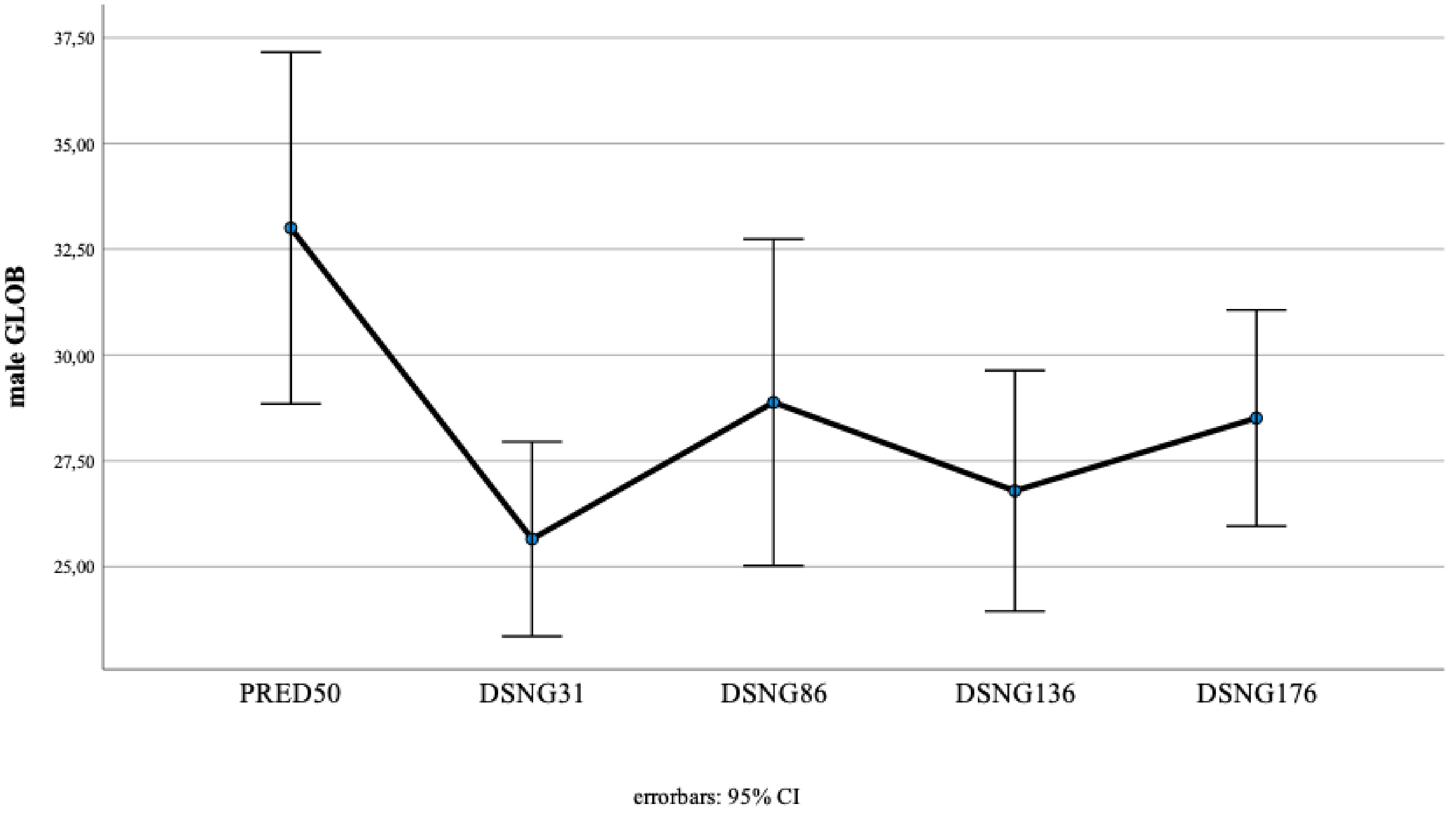

ALB male

Der Mauchly-Test liefert ein signifikantes Ergebnis, $\chi^2(9) = 20{,}67, p = 0{,}025$. Somit ist die Sphärizitätsannahme hier verletzt. Die Schätzung des Haupteffekts wird hierbei mittels der robusten Untergrenze erfolgen.

Mauchly-Test auf Sphärizität

Maß: MASS_1

Innersubjekteffekt	Mauchly-W	Ungefähres Chi-Quadrat	df	Sig.	Epsilon Greenhouse-Geisser	Epsilon Huynh-Feldt (HF)	Epsilon Untergrenze
Zeit	,002	20,665	9	,025	,319	,379	,250

Dabei zeigen sich signifikante Unterschiede zwischen den Zeitpunkten, $F(1,5) = 14{,}21, p = 0{,}013, \eta^2 = 0{,}74$. Der Effekt ist dabei stark.

Tests der Innersubjekteffekte

Maß: MASS_1

Quelle		Typ III Quadratsumme	df	Mittel der Quadrate	F	Sig.	Partielles Eta-Quadrat
Zeit	Sphärizität angenommen	233,727	4	58,432	14,205	,000	,740
	Greenhouse-Geisser	233,727	1,274	183,393	14,205	,007	,740
	Huynh-Feldt (HF)	233,727	1,516	154,206	14,205	,004	,740
	Untergrenze	233,727	1,000	233,727	14,205	,013	,740
Fehler(Zeit)	Sphärizität angenommen	82,268	20	4,113			
	Greenhouse-Geisser	82,268	6,372	12,910			
	Huynh-Feldt (HF)	82,268	7,578	10,856			
	Untergrenze	82,268	5,000	16,454			

Auf Grund des signifikanten Haupteffekts wird eine Post-Hoc-Analyse erfolgen. Dabei zeigt sich nur der paarweise Vergleich zwischen DSNG31 und DSNG136 signifikant.

Paarweise Vergleiche

Maß: MASS_1

(I) Zeit	(J) Zeit	Mittelwertdifferenz (I-J)	Std.-Fehler	Sig.	95% Konfidenzintervall für Differenz Untergrenze	95% Konfidenzintervall für Differenz Obergrenze
1	2	4,947	1,452	,191	-1,983	11,877
	3	8,197	2,072	,108	-1,692	18,085
	4	6,437	1,538	,086	-,905	13,779
	5	6,358	1,661	,123	-1,569	14,285
2	1	-4,947	1,452	,191	-11,877	1,983
	3	3,250	,824	,109	-,684	7,184
	4	1,490	,308	,047	,022	2,958
	5	1,412	,342	,091	-,219	3,042
3	1	-8,197	2,072	,108	-18,085	1,692
	2	-3,250	,824	,109	-7,184	,684

	4	-1,760	,707	,551	-5,133	1,613
	5	-1,838	,824	,759	-5,769	2,093
4	1	-6,437	1,538	,086	-13,779	,905
	2	-1,490	,308	,047	-2,958	-,022
	3	1,760	,707	,551	-1,613	5,133
	5	-,078	,346	1,000	-1,732	1,575
5	1	-6,358	1,661	,123	-14,285	1,569
	2	-1,412	,342	,091	-3,042	,219
	3	1,838	,824	,759	-2,093	5,769
	4	,078	,346	1,000	-1,575	1,732

Kommende Grafik zeigt die geschätzten Mittelwerte. Es zeigt sich, dass der Mittelwert von PRED50 zu DSNG31 fällt. Danach schwankt er, jedoch bleibt der Mittelwert über die übrigen Zeitpunkte etwa auf einem Niveau.

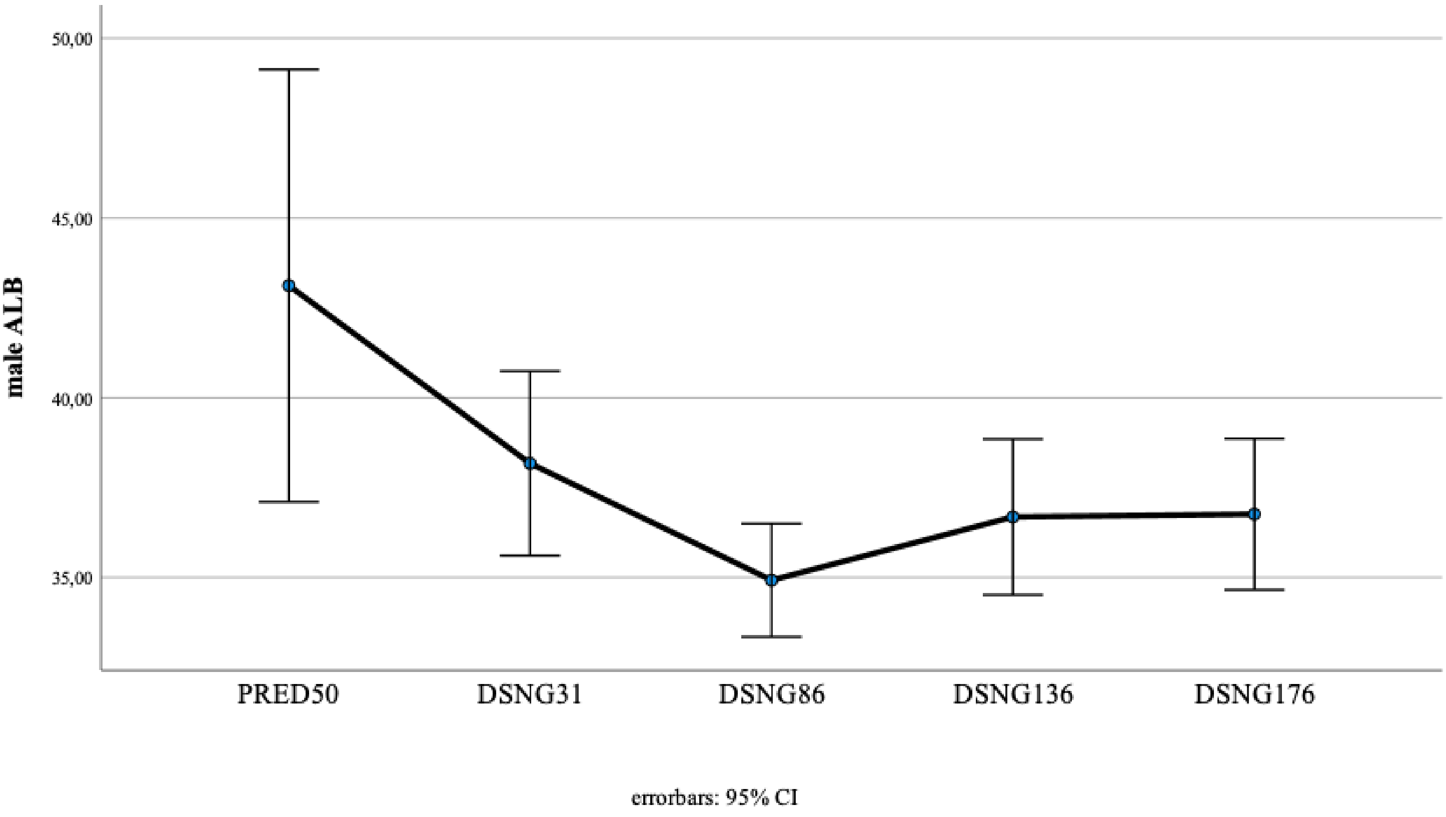

CHOL male

Hier liefert der Mauchly-Test ein nicht-signifikantes Ergebnis, $\chi^2(9) = 12{,}43, (9) = 12{,}43, p = 0{,}238$. Damit ist

die Annahme der Sphärizität erfüllt.

Mauchly-Test auf Sphärizität

Maß: MASS_1

Innersubjekteffekt	Mauchly-W	Ungefähres Chi-Quadrat	df	Sig.	Epsilon: Greenhouse-Geisser	Epsilon: Huynh-Feldt (HF)	Epsilon: Untergrenze
Zeit	,026	12,433	9	,238	,585	1,000	,250

Es zeigt sich ein signifikanter Haupteffekt Zeit, $F(4, 20) = 19{,}28, p = 0{,}000,\ \eta^2 = 0{,}79$. Der Effekt ist stark.

Tests der Innersubjekteffekte

Maß: MASS_1

Quelle		Typ III Quadratsumme	df	Mittel der Quadrate	F	Sig.	Partielles Eta-Quadrat
Zeit	Sphärizität angenommen	7,876	4	1,969	19,282	,000	,794
	Greenhouse-Geisser	7,876	2,339	3,367	19,282	,000	,794
	Huynh-Feldt (HF)	7,876	4,000	1,969	19,282	,000	,794
	Untergrenze	7,876	1,000	7,876	19,282	,007	,794
Fehler(Zeit)	Sphärizität angenommen	2,042	20	,102			
	Greenhouse-Geisser	2,042	11,697	,175			
	Huynh-Feldt (HF)	2,042	20,000	,102			
	Untergrenze	2,042	5,000	,408			

Auf Grund des signifikanten Haupteffekts erfolgt eine Post-Hoc-Analyse. Es zeigt sich dabei, dass sich nur PRED50 vs. DSNG31, PRED50 vs. DSNG136 und PRED50 vs. DSNG176 signifikant unterscheiden.

Paarweise Vergleiche

Maß: MASS_1

(I) Zeit	(J) Zeit	Mittelwertdifferenz (I-J)	Std.-Fehler	Sig.	95% Konfidenzintervall für Differenz: Untergrenze	95% Konfidenzintervall für Differenz: Obergrenze
1	2	-1,030	,151	,010	-1,752	-,308
	3	-,757	,211	,159	-1,766	,253
	4	-1,195	,137	,003	-1,848	-,542
	5	-1,515	,095	,000	-1,970	-1,060

2	1	1,030	,151	,010	,308	1,752
	3	,273	,204	1,000	-,699	1,246
	4	-,165	,180	1,000	-1,024	,694
	5	-,485	,222	,811	-1,547	,577
3	1	,757	,211	,159	-,253	1,766
	2	-,273	,204	1,000	-1,246	,699
	4	-,438	,210	,911	-1,440	,564
	5	-,758	,253	,304	-1,968	,451
4	1	1,195	,137	,003	,542	1,848
	2	,165	,180	1,000	-,694	1,024
	3	,438	,210	,911	-,564	1,440
	5	-,320	,116	,394	-,872	,232
5	1	1,515	,095	,000	1,060	1,970
	2	,485	,222	,811	-,577	1,547
	3	,758	,253	,304	-,451	1,968
	4	,320	,116	,394	-,232	,872

Kommende Grafik zeigt die geschätzten Mittelwerte visuell. Es zeigt sich, dass CHOL über die Zeit ansteigt.

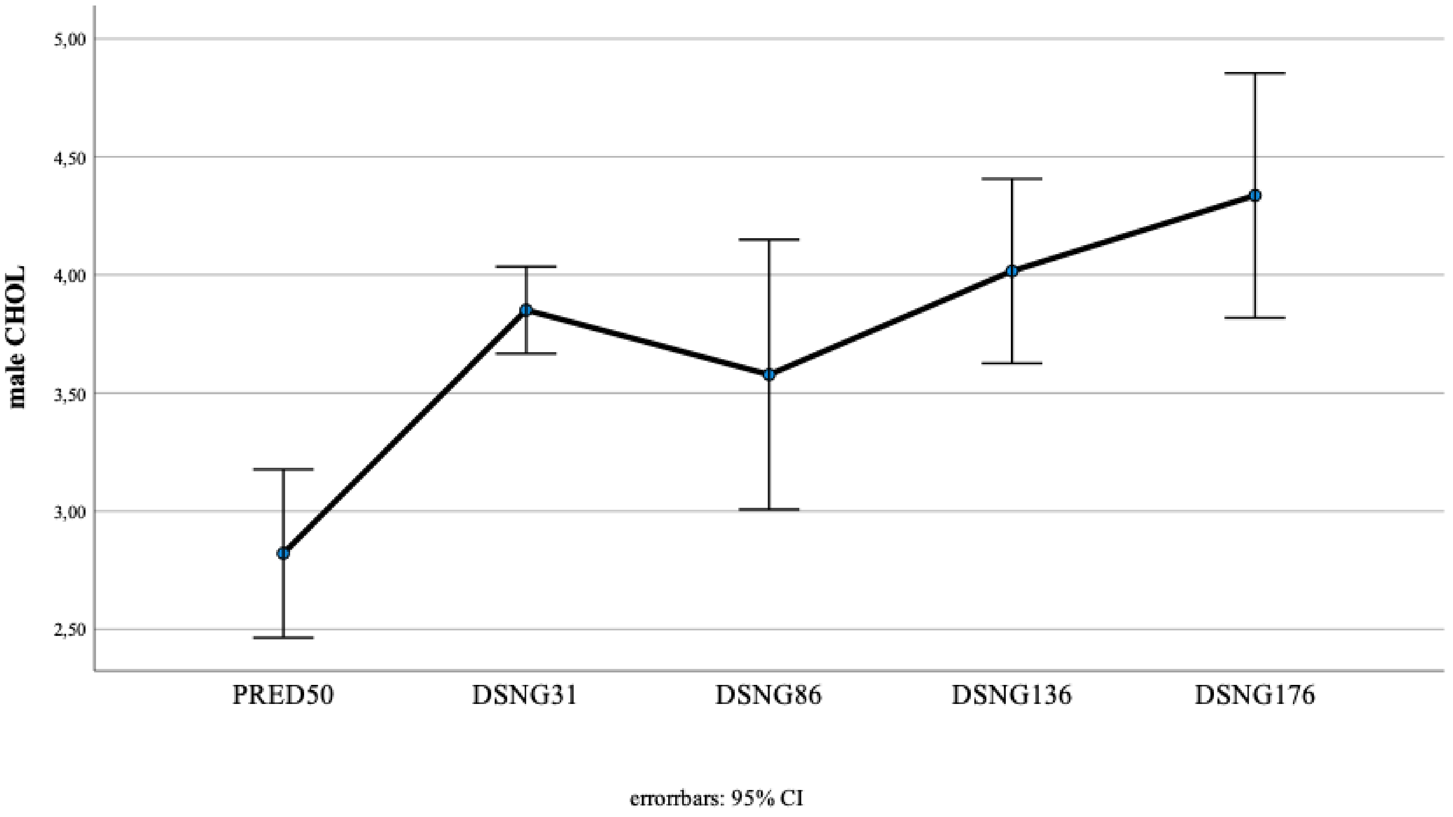

Studie P

ALT female

Im Falle von ALT zeigte sich, dass der Mauchly-Test ein signifikantes Ergebnis liefert, $\chi^2(27) = 146{,}60, p = 0{,}000$ Damit wird die Varianzanalyse mittels der robusten Untergrenze berechnet.

Mauchly-Test auf Sphärizität

Maß: MASS_1

Innersubjekteffekt	Mauchly-W	Ungefähres Chi-Quadrat	df	Sig.	Epsilon: Greenhouse-Geisser	Epsilon: Huynh-Feldt (HF)	Epsilon: Untergrenze
Zeit	,000	146,600	27	,000	,170	,213	,143

Hierbei zeigt sich ein nicht-signifikanter Haupteffekt Zeit, $F(1, 11) = 1{,}03, p = 0{,}331, \eta^2 = 0{,}09$. Der Effekt ist mittelstark. Die Interaktion zeigt dabei einen nicht-signifikanten Einfluss, $F(2, 11) = 0{,}90, p = 0{,}433, \eta^2 > 0{,}14$. Damit ist der Effekt stark.

Tests der Innersubjekteffekte

Maß: MASS_1

Quelle		Typ III Quadratsumme	df	Mittel der Quadrate	F	Sig.	Partielles Eta-Quadrat
Zeit	Sphärizität angenommen	13716,456	7	1959,494	1,034	,415	,086
	Greenhouse-Geisser	13716,456	1,188	11547,076	1,034	,343	,086
	Huynh-Feldt (HF)	13716,456	1,491	9199,290	1,034	,356	,086
	Untergrenze	13716,456	1,000	13716,456	1,034	,331	,086
Zeit * Group	Sphärizität angenommen	23976,754	14	1712,625	,903	,558	,141
	Greenhouse-Geisser	23976,754	2,376	10092,308	,903	,445	,141
	Huynh-Feldt (HF)	23976,754	2,982	8040,310	,903	,460	,141
	Untergrenze	23976,754	2,000	11988,377	,903	,433	,141
Fehler(Zeit)	Sphärizität angenommen	145965,702	77	1895,658			
	Greenhouse-Geisser	145965,702	13,067	11170,902			
	Huynh-Feldt (HF)	145965,702	16,401	8899,601			
	Untergrenze	145965,702	11,000	13269,609			

Der Haupteffekt Gruppe ist dabei nicht-signifikant, $F(2, 11) = 0{,}053, \eta^2 = 0{,}41$. Der Effekt ist stark.

Tests der Zwischensubjekteffekte

Maß: MASS_1

Transformierte Variable: Mittel

Quelle	Typ III Quadratsumme	df	Mittel der Quadrate	F	Sig.	Partielles Eta-Quadrat
Konstanter Term	370673,007	1	370673,007	66,911	,000	,859
Group	42972,478	2	21486,239	3,879	,053	,414
Fehler	60937,713	11	5539,792			

Kommende Grafik zeigt die Mittelwerte nochmals visuell. HD hat zu allen Zeitpunkten bis auf dem letzten die höchsten Mittelwerte. Bis DSNG93 sind die Mittelwerte von LD und MD etwa gleich stark ausgeprägt. Ab DSNG135 liegen die Mittelwerte von MD oberhalb von LD. Zu DSNG275 liegt der Mittelwert von MG über dem von HD.

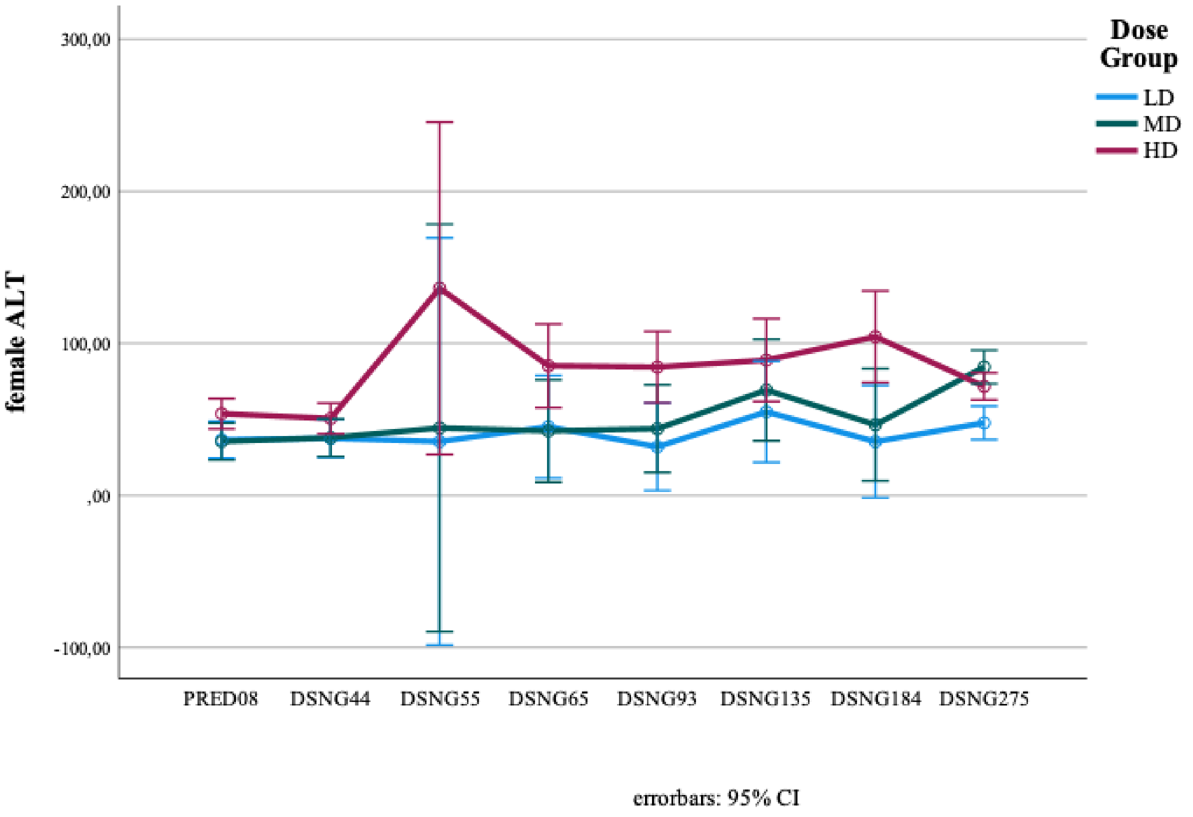

GLDH female

Im Falle von GLDH zeigt sich mittels Mauchly-Test ein signifikantes Ergebnis, $\chi^2(27) = 143{,}86, p = 0{,}000.$ Aus diesem Grunde wird die ANOVA mittels der robusten Untergrenze berechnet.

Mauchly-Test auf Sphärizität

Maß: MASS_1

Innersubjekteffekt	Mauchly-W	Ungefähres Chi-Quadrat	df	Sig.	Epsilon Greenhouse-Geisser	Huynh-Feldt (HF)	Untergrenze

Zeit	,000	143,863	27	,000	,199	,260	,143

Dabei zeigt sich ein nicht-signifikanter Haupteffekt Zeit, $F(1, 11) = 1{,}11, p = 0{,}315, \eta^2 = 0{,}09$. Der Effekt ist mittelstark. Weiterhin übt die Interaktion einen nicht-signifikanten Einfluss auf GLDH aus, $F(2, 11) = 0{,}81, p = 0{,}471, \eta^2 = 0{,}13$. Der Effekt ist mittelstark.

Tests der Innersubjekteffekte

Maß: MASS_1

Quelle		Typ III Quadratsumme	df	Mittel der Quadrate	F	Sig.	Partielles Eta-Quadrat
Zeit	Sphärizität angenommen	5334,940	7	762,134	1,108	,367	,092
	Greenhouse-Geisser	5334,940	1,392	3833,861	1,108	,333	,092
	Huynh-Feldt (HF)	5334,940	1,819	2932,286	1,108	,344	,092
	Untergrenze	5334,940	1,000	5334,940	1,108	,315	,092
Zeit * Group	Sphärizität angenommen	7777,169	14	555,512	,808	,658	,128
	Greenhouse-Geisser	7777,169	2,783	2794,463	,808	,501	,128
	Huynh-Feldt (HF)	7777,169	3,639	2137,314	,808	,525	,128
	Untergrenze	7777,169	2,000	3888,585	,808	,471	,128
Fehler(Zeit)	Sphärizität angenommen	52951,634	77	687,684			
	Greenhouse-Geisser	52951,634	15,307	3459,342			
	Huynh-Feldt (HF)	52951,634	20,013	2645,839			
	Untergrenze	52951,634	11,000	4813,785			

Der Haupteffekt Gruppe ist hierbei signifikant, $F(2, 11) = 4{,}25, p = 0{,}043, \eta^2 = 0{,}44$. Der Effekt ist stark.

Tests der Zwischensubjekteffekte

Maß: MASS_1

Transformierte Variable: Mittel

Quelle	Typ III Quadratsumme	df	Mittel der Quadrate	F	Sig.	Partielles Eta-Quadrat
Konstanter Term	67354,953	1	67354,953	40,806	,000	,788
Group	14017,208	2	7008,604	4,246	,043	,436
Fehler	18156,936	11	1650,631			

Da zwischen den Gruppen signifikante Unterschiede vorliegen, erfolgt nun die Post-Hoc-Analyse für den Haupteffekt Gruppe. Es zeigt sich, dass keiner der Effekte signifikant war. Dies liegt an der kleinen Stichprobe und damit einer geringen statistischen Power. Die Power ist die Wahrscheinlichkeit einen Effekt nachzuweisen, wenn er tatsächlich in der Grundgesamtheit vorliegt.

Paarweise Vergleiche

Maß: MASS_1

(I) Dose Group	(J) Dose Group	Mittelwertdifferenz (I-J)	Std.-Fehler	Sig.	95% Konfidenzintervall für Differenz	
					Untergrenze	Obergrenze
LD	MD	-3,289	10,157	1,000	-31,932	25,354
	HD	-24,111	9,272	,074	-50,258	2,036
MD	LD	3,289	10,157	1,000	-25,354	31,932
	HD	-20,822	9,272	,139	-46,969	5,326
HD	LD	24,111	9,272	,074	-2,036	50,258
	MD	20,822	9,272	,139	-5,326	46,969

Kommende Grafik zeigt die Unterschiede in den Mittelwerten visuell. Es zeigt sich, dass HD über den gesamten Zeitraum die höchsten Mittelwerte hat. MD überholt LD ab DSNG65. Davor sind beide Gruppen etwa gleich auf.

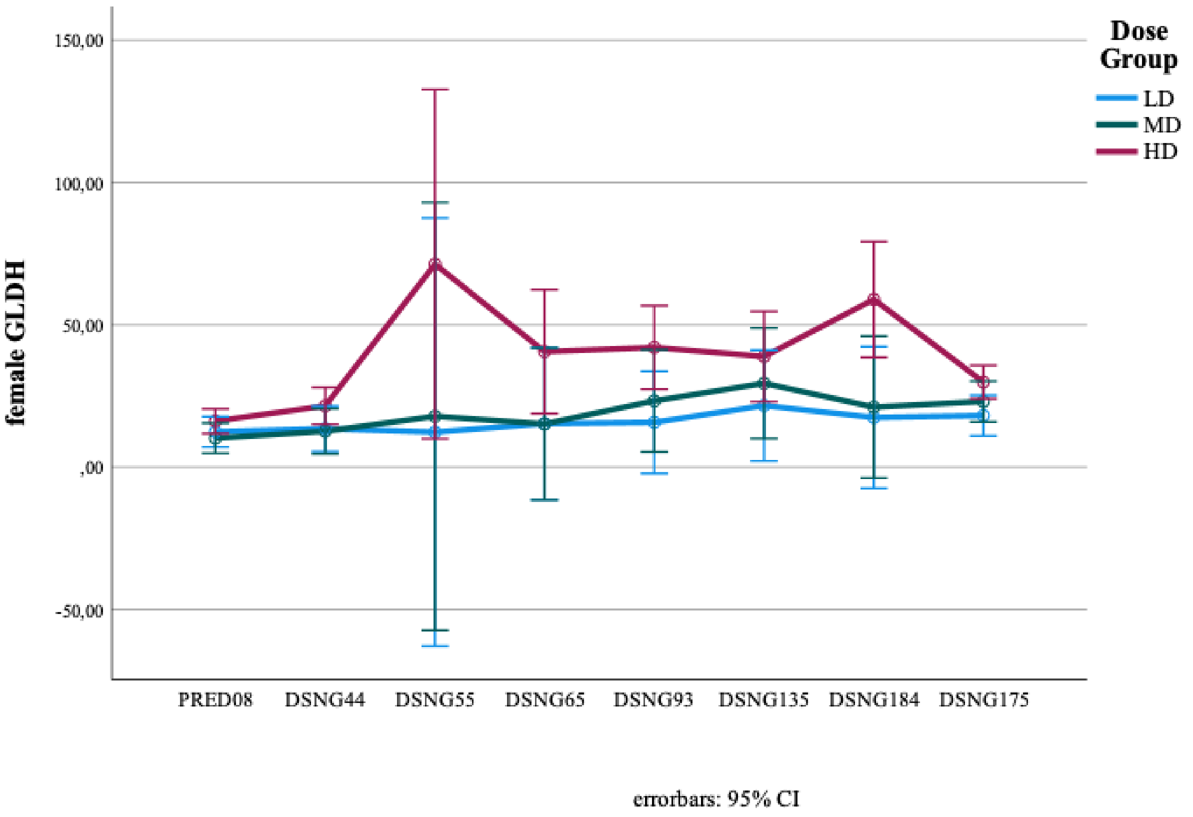

Studie Q

AST female

Der Haupteffekt Zeit ist im Falle von AST signifikant, $F(1,11) = 20{,}13, p = 0{,}001, \eta^2 = 0{,}65$. Der Effekt ist dabei stark. Weiterhin zeigt sich eine nicht-signifikante Interaktion, $F(2,11) = 3{,}83, p = 0{,}055, \eta^2 = 0{,}41$. Der Effekt ist ebenfalls stark.

Tests der Innersubjekteffekte

Maß: MASS_1

Quelle		Typ III Quadratsumme	df	Mittel der Quadrate	F	Sig.	Partielles Eta-Quadrat
Zeit	Sphärizität angenommen	29903,578	1	29903,578	20,127	,001	,647
	Greenhouse-Geisser	29903,578	1,000	29903,578	20,127	,001	,647
	Huynh-Feldt (HF)	29903,578	1,000	29903,578	20,127	,001	,647
	Untergrenze	29903,578	1,000	29903,578	20,127	,001	,647
Zeit * Group	Sphärizität angenommen	11381,816	2	5690,908	3,830	,055	,411
	Greenhouse-Geisser	11381,816	2,000	5690,908	3,830	,055	,411
	Huynh-Feldt (HF)	11381,816	2,000	5690,908	3,830	,055	,411
	Untergrenze	11381,816	2,000	5690,908	3,830	,055	,411
Fehler(Zeit)	Sphärizität angenommen	16343,010	11	1485,728			
	Greenhouse-Geisser	16343,010	11,000	1485,728			
	Huynh-Feldt (HF)	16343,010	11,000	1485,728			
	Untergrenze	16343,010	11,000	1485,728			

Zwischen den Gruppen konnte jedoch kein signifikanter Unterschied nachgewiesen werden, $F(2,11) = 1{,}41, p = 0{,}284, \eta^2 = 0{,}21$. Der Effekt ist stark.

Tests der Zwischensubjekteffekte

Maß: MASS_1

Transformierte Variable: Mittel

Quelle	Typ III Quadratsumme	df	Mittel der Quadrate	F	Sig.	Partielles Eta-Quadrat

Konstanter Term	619213,172	1	619213,172	156,232	,000	,934
Group	11211,514	2	5605,757	1,414	,284	,205
Fehler	43597,685	11	3963,426			

Kommende Grafik zeigt die Mittelwert im Verlauf der Zeit zwischen den Gruppen. Es zeigt sich, dass bei alle Gruppen die Mittelwerte ansteigen. Je nach Dosisstärke, verstärkt sich auch der Anstieg in den Mittelwerten.

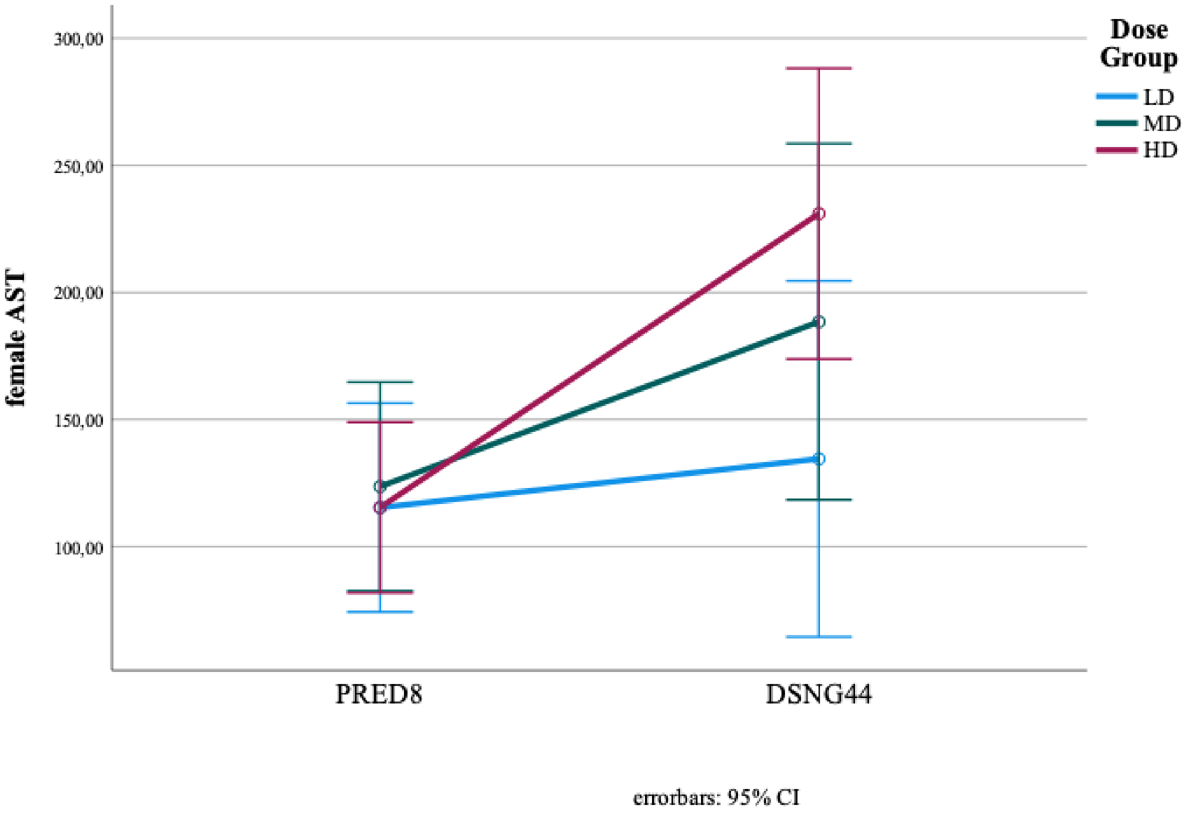

GGT female

Im Falle von GGT zeigte sich ein nicht-signifikanter Haupteffekt Zeit, $F(1, 11) = 2{,}85, p = 0{,}120, \eta^2 = 0{,}21$. Der Effekt ist stark. Die Interaktion ist ebenfalls nicht-signifikant, $F(2, 11) = 2{,}75, p = 0{,}108, \eta^2 = 0{,}33$. Der Effekt ist auch stark.

Tests der Innersubjekteffekte

Maß: MASS_1

Quelle		Typ III Quadratsumme	df	Mittel der Quadrate	F	Sig.	Partielles Eta-Quadrat
Zeit	Sphärizität angenommen	18181,089	1	18181,089	2,848	,120	,206
	Greenhouse-Geisser	18181,089	1,000	18181,089	2,848	,120	,206

	Huynh-Feldt (HF)	18181,089	1,000	18181,089	2,848	,120	,206
	Untergrenze	18181,089	1,000	18181,089	2,848	,120	,206
Zeit * Group	Sphärizität angenommen	35077,646	2	17538,823	2,748	,108	,333
	Greenhouse-Geisser	35077,646	2,000	17538,823	2,748	,108	,333
	Huynh-Feldt (HF)	35077,646	2,000	17538,823	2,748	,108	,333
	Untergrenze	35077,646	2,000	17538,823	2,748	,108	,333
Fehler(Zeit)	Sphärizität angenommen	70209,595	11	6382,690			
	Greenhouse-Geisser	70209,595	11,000	6382,690			
	Huynh-Feldt (HF)	70209,595	11,000	6382,690			
	Untergrenze	70209,595	11,000	6382,690			

Zwischen den Gruppen zeigten sich ebenfalls nicht-signifikante Unterschiede, $F(2,11) = 2{,}71, p = 0{,}110, \eta^2 = 0{,}33$.

Tests der Zwischensubjekteffekte

Maß: MASS_1

Transformierte Variable: Mittel

Quelle	Typ III Quadratsumme	df	Mittel der Quadrate	F	Sig.	Partielles Eta-Quadrat
Konstanter Term	31255,875	1	31255,875	4,821	,050	,305
Group	35149,399	2	17574,700	2,711	,110	,330
Fehler	71318,922	11	6483,538			

Kommende Grafik zeigt die Mittewerte visuell. Es ist zu erkennen, dass nur der Mittelwert der HD sich über die Zeit verändert.

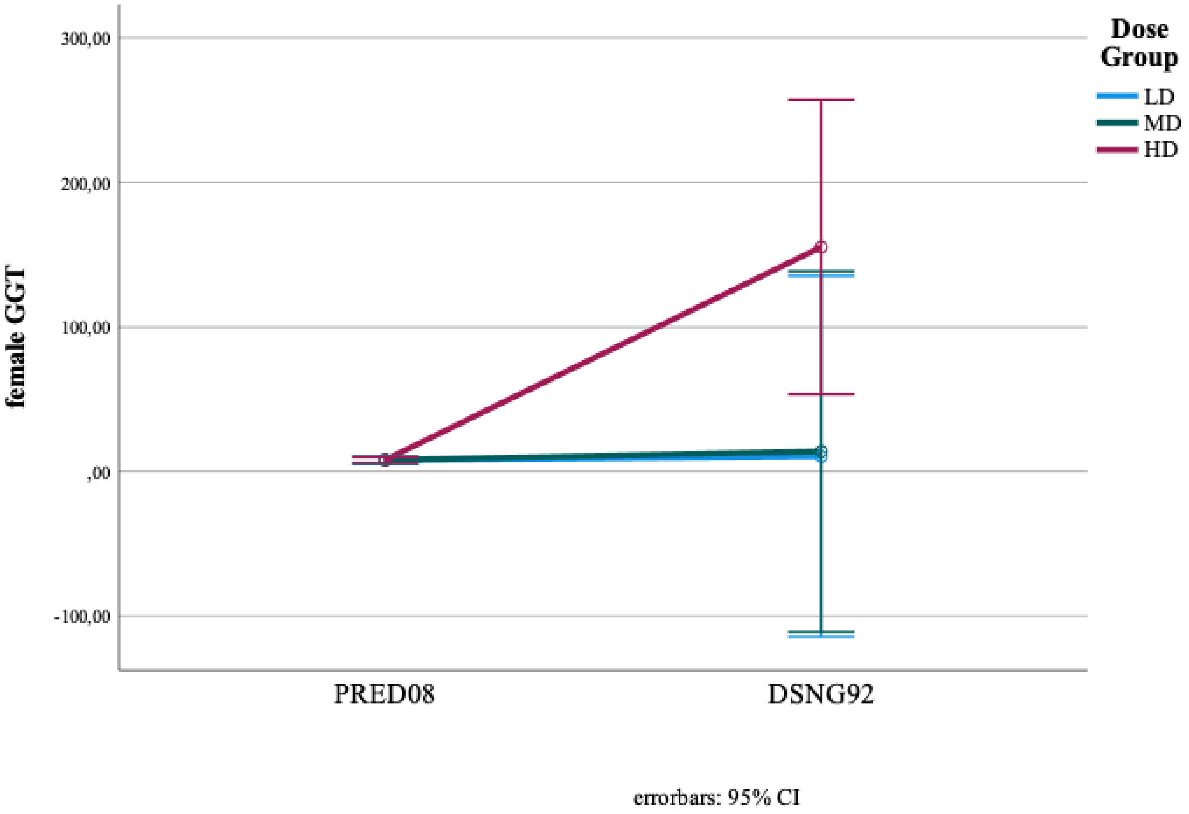

Studie R

Im Falle von CD20+ B-cells female zeigt sich ein signifikanter Haupteffekt Zeit, $F(1,10) = 50{,}21, p = 0{,}000, \eta^2 = 0{,}83.$ Der Effekt ist stark. Weiterhin ist die Interaktion signifikant, $F(1,10) = 6{,}75, p = 0{,}027, \eta^2 = 0{,}40.$ Dieser Effekt ist ebenfalls stark.

Tests der Innersubjekteffekte

Maß: MASS_1

Quelle		Typ III Quadratsumme	df	Mittel der Quadrate	F	Sig.	Partielles Eta-Quadrat
Zeit	Sphärizität angenommen	8,225	1	8,225	50,207	,000	,834
	Greenhouse-Geisser	8,225	1,000	8,225	50,207	,000	,834
	Huynh-Feldt (HF)	8,225	1,000	8,225	50,207	,000	,834
	Untergrenze	8,225	1,000	8,225	50,207	,000	,834
Zeit * Group	Sphärizität angenommen	1,105	1	1,105	6,746	,027	,403
	Greenhouse-Geisser	1,105	1,000	1,105	6,746	,027	,403
	Huynh-Feldt (HF)	1,105	1,000	1,105	6,746	,027	,403
	Untergrenze	1,105	1,000	1,105	6,746	,027	,403
Fehler(Zeit)	Sphärizität angenommen	1,638	10	,164			
	Greenhouse-Geisser	1,638	10,000	,164			
	Huynh-Feldt (HF)	1,638	10,000	,164			
	Untergrenze	1,638	10,000	,164			

Zwischen den Gruppen lagen wiederrum keine signifikanten Unterschiede vor, $F(1, 10) = 1{,}97, p = 0{,}191, \eta^2 = 0{,}16$. Der Effekt ist stark.

Tests der Zwischensubjekteffekte

Maß: MASS_1

Transformierte Variable: Mittel

Quelle	Typ III Quadratsumme	df	Mittel der Quadrate	F	Sig.	Partielles Eta-Quadrat
Konstanter Term	9,972	1	9,972	33,864	,000	,772
Group	,580	1	,580	1,969	,191	,164
Fehler	2,945	10	,294			

Kommende Grafik zeigt die Mittelwerte visuell. Es ist zu erkennen, dass zu PRED71 die D2 einen höheren Mittelwert aufweist als die DG1. Zu DSNG85 ist dies umgekehrt.

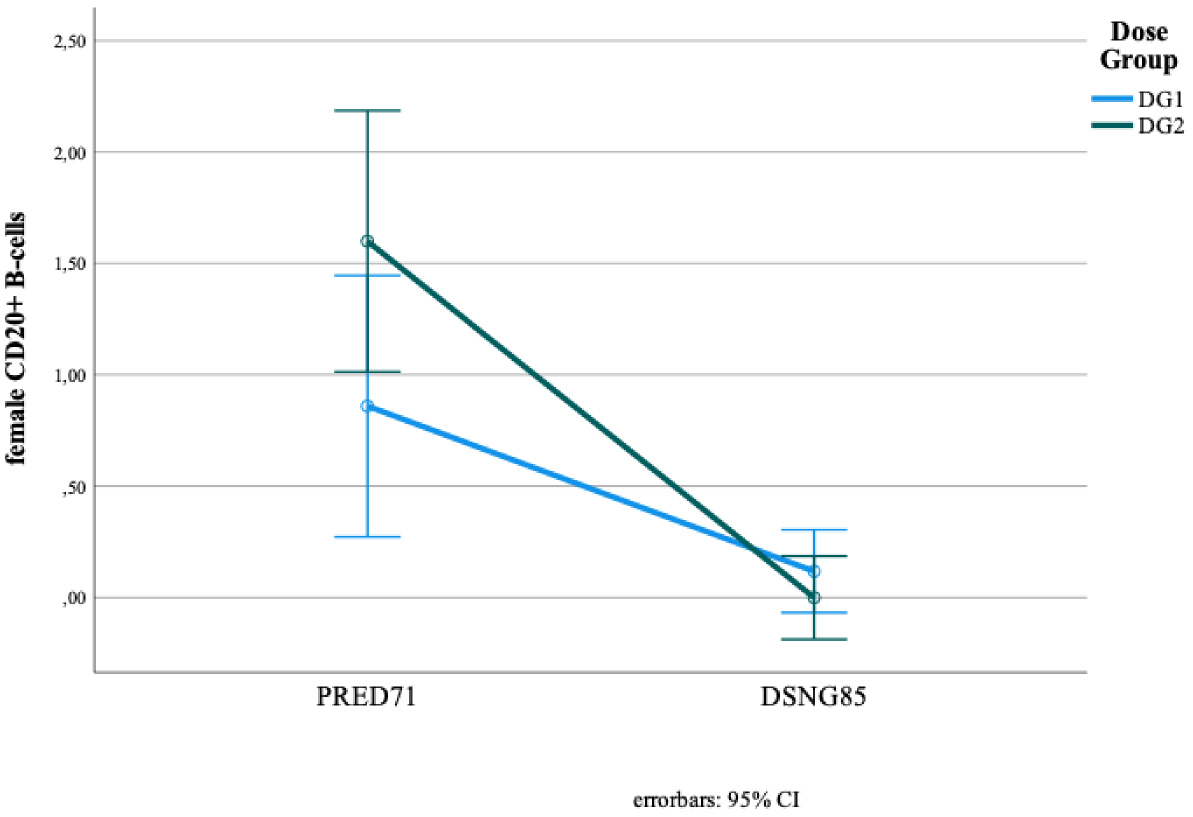

Studie S

IgG female

Im Falle von IgG zeigt sich ein signifikantes Ergebnis bei dem Mauchly-Test, $\chi^2(5) = 11{,}26, p = 0{,}049$. Somit ist die Annahme der Sphärizität hier verletzt. Es wird die robuste Untergrenze als Schätzer für die ANOVA benutzt.

Mauchly-Test auf Sphärizität

Maß: MASS_1

Innersubjekteffekt	Mauchly-W	Ungefähres Chi-Quadrat	df	Sig.	Epsilon: Greenhouse-Geisser	Epsilon: Huynh-Feldt (HF)	Epsilon: Untergrenze
Zeit	,187	11,258	5	,049	,689	1,000	,333

Es zeigt sich hierbei ein signifikanter Haupteffekt Zeit, $F(1,8) = 36{,}43, p = 0{,}000, \eta^2 = 0{,}82$. Der Effekt ist stark. Die Interaktion übt einen nicht-signifikanten Einfluss aus, $F(1,8) = 1{,}42, p = 0{,}268, \eta^2 = 0{,}15$. Der Effekt ist ebenfalls stark.

Tests der Innersubjekteffekte

Maß: MASS_1

Quelle		Typ III Quadratsumme	df	Mittel der Quadrate	F	Sig.	Partielles Eta-Quadrat
Zeit	Sphärizität angenommen	165,164	3	55,055	36,433	,000	,820
	Greenhouse-Geisser	165,164	2,067	79,893	36,433	,000	,820
	Huynh-Feldt (HF)	165,164	3,000	55,055	36,433	,000	,820
	Untergrenze	165,164	1,000	165,164	36,433	,000	,820
Zeit * Group	Sphärizität angenommen	6,425	3	2,142	1,417	,262	,150
	Greenhouse-Geisser	6,425	2,067	3,108	1,417	,271	,150
	Huynh-Feldt (HF)	6,425	3,000	2,142	1,417	,262	,150
	Untergrenze	6,425	1,000	6,425	1,417	,268	,150
Fehler(Zeit)	Sphärizität angenommen	36,267	24	1,511			
	Greenhouse-Geisser	36,267	16,538	2,193			
	Huynh-Feldt (HF)	36,267	24,000	1,511			
	Untergrenze	36,267	8,000	4,533			

Der Haupteffekt Gruppe übt keinen signifikanten Einfluss auf IgG aus, $F(1,8) = 0{,}44, p = 0{,}527, \eta^2 = 0{,}05$. Der Effekt ist schwach.

Tests der Zwischensubjekteffekte

Maß: MASS_1
Transformierte Variable: Mittel

Quelle	Typ III Quadratsumme	df	Mittel der Quadrate	F	Sig.	Partielles Eta-Quadrat
Konstanter Term	1768,823	1	1768,823	168,957	,000	,955
Group	4,573	1	4,573	,437	,527	,052
Fehler	83,753	8	10,469			

Da der Haupteffekt Zeit einen signifikanten Einfluss ausübt werden in einer Post-Hoc-Analyse die Zeitpunkte paarweise verglichen. Hierbei zeigt sich, dass sich PRED19/33 sich jeweils signifikant von DSNG25/27, DSNG43 und DSNG86 unterscheidet. Weiterhin unterscheiden sich DSNG43 und DSNG86 signifikant voneinander.

Paarweise Vergleiche

Maß: MASS_1

(I) Zeit	(J) Zeit	Mittelwertdifferenz (I-J)	Std.-Fehler	Sig.	95% Konfidenzintervall für Differenz	
					Untergrenze	Obergrenze
1	2	4,485	,571	,000	2,498	6,471
	3	3,864	,694	,003	1,450	6,278
	4	5,465	,627	,000	3,285	7,645
2	1	-4,485	,571	,000	-6,471	-2,498
	3	-,620	,364	,763	-1,888	,647
	4	,980	,620	,914	-1,175	3,136
3	1	-3,864	,694	,003	-6,278	-1,450
	2	,620	,364	,763	-,647	1,888
	4	1,601	,415	,029	,158	3,044
4	1	-5,465	,627	,000	-7,645	-3,285
	2	-,980	,620	,914	-3,136	1,175
	3	-1,601	,415	,029	-3,044	-,158

Kommende Grafik zeigt die Effekt visuell. Es ist zu erkennen, dass Zu Beginn der Mittelwert in DG1 marginal höher ist als in DG2. Die übrigen Zeitpunkte liegt der Mittelwert von DG2 oberhalb den von DG1. Ansonsten

wirken die Verläufe zwischen den Gruppen ähnlich.

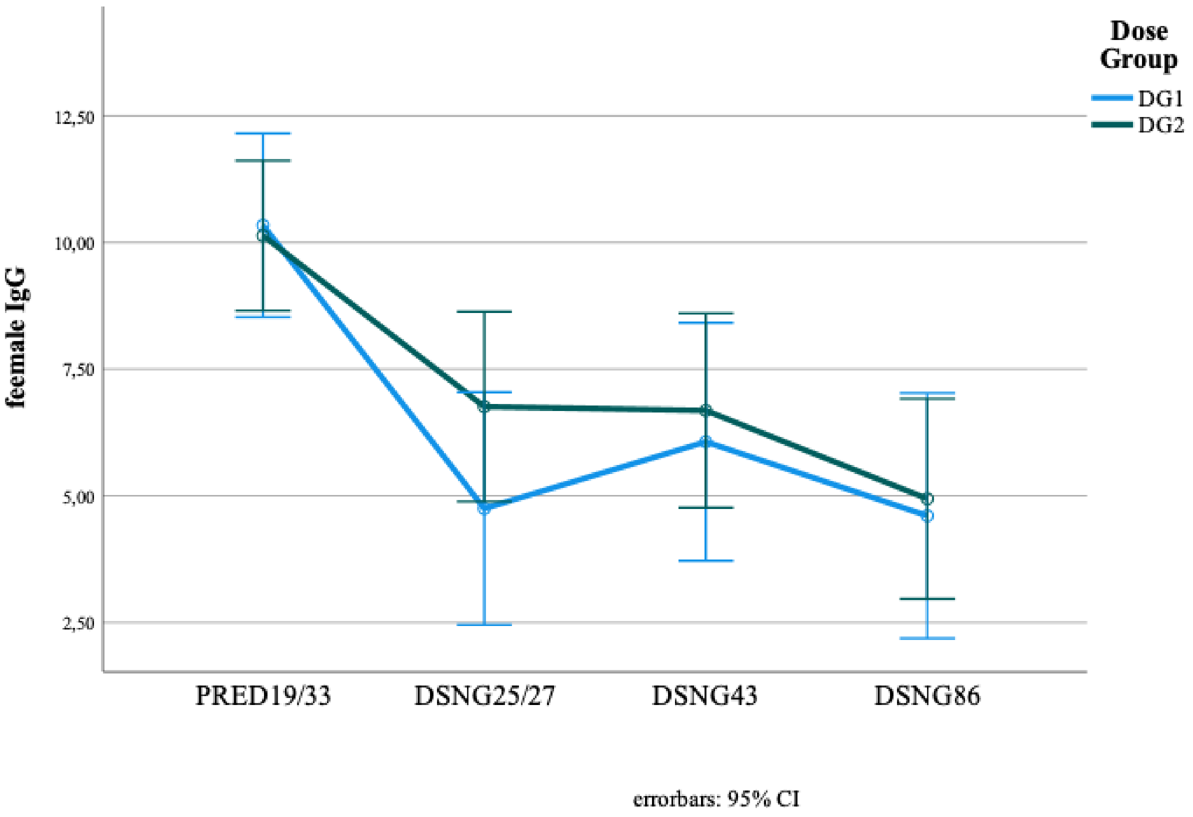

CA female

Bei der Untersuchung von CA zeigt sich ein signifikantes Ergebnis bei dem Mauchly-Test, $\chi^2(5) = 41{,}08, p = 0{,}000$. Somit wird die robuste Untergrenze bei der ANOVA zur Schätzung verwendet.

Mauchly-Test auf Sphärizität

Maß: MASS_1

Innersubjekteffekt	Mauchly-W	Ungefähres Chi-Quadrat	df	Sig.	Epsilon Greenhouse-Geisser	Epsilon Huynh-Feldt (HF)	Epsilon Untergrenze
Zeit	,002	41,082	5	,000	,347	,403	,333

Es zeigt sich hierbei ein nicht-signifikanter Haupteffekt Zeit, $F(1,8) = 5{,}01, p = 0{,}056, \eta^2 = 0{,}39$. Der Effekt ist stark. Weiterhin übt die Interaktion ebenfalls einen nicht-signifikanten Einfluss aus, $F(1,8) = 1{,}28, p = 0{,}290, \eta^2 = 0{,}14$. Der Effekt ist mittelstark.

Tests der Innersubjekteffekte

Maß: MASS_1

Quelle		Typ III Quadratsumme	df	Mittel der Quadrate	F	Sig.	Partielles Eta-Quadrat
Zeit	Sphärizität angenommen	3,513	3	1,171	5,011	,008	,385
	Greenhouse-Geisser	3,513	1,042	3,372	5,011	,053	,385
	Huynh-Feldt (HF)	3,513	1,210	2,904	5,011	,045	,385
	Untergrenze	3,513	1,000	3,513	5,011	,056	,385
Zeit * Group	Sphärizität angenommen	,899	3	,300	1,283	,303	,138
	Greenhouse-Geisser	,899	1,042	,863	1,283	,292	,138
	Huynh-Feldt (HF)	,899	1,210	,743	1,283	,296	,138
	Untergrenze	,899	1,000	,899	1,283	,290	,138
Fehler(Zeit)	Sphärizität angenommen	5,607	24	,234			
	Greenhouse-Geisser	5,607	8,333	,673			
	Huynh-Feldt (HF)	5,607	9,676	,580			
	Untergrenze	5,607	8,000	,701			

Ebenfalls hat der Haupteffekt Gruppe einen nicht-signifikanten Einfluss auf CA, $F(1,\ 8) =\ 3{,}07, p = 0{,}118, \eta^2 = 0{,}28$. Der Effekt ist stark.

Tests der Zwischensubjekteffekte

Maß: MASS_1
Transformierte Variable: Mittel

Quelle	Typ III Quadratsumme	df	Mittel der Quadrate	F	Sig.	Partielles Eta-Quadrat
Konstanter Term	221,818	1	221,818	1188,014	,000	,993
Group	,573	1	,573	3,071	,118	,277
Fehler	1,494	8	,187			

Kommende Grafik zeigt die Verläufe der Mittelwerte über die Zeit zwischen den Gruppen. Es ist zu erkennen, dass zu Beginn der Mittelwert der DG1 stärker ausgeprägt ist als in der DG2. Über die übrigen Zeitpunkte sind sie etwa gleich stark ausgeprägt. Auch zum Ende des Versuchs ist der Mittelwert in D1 höher. Im Vergleich zu Beginn des Versuchs jedoch eher marginal.

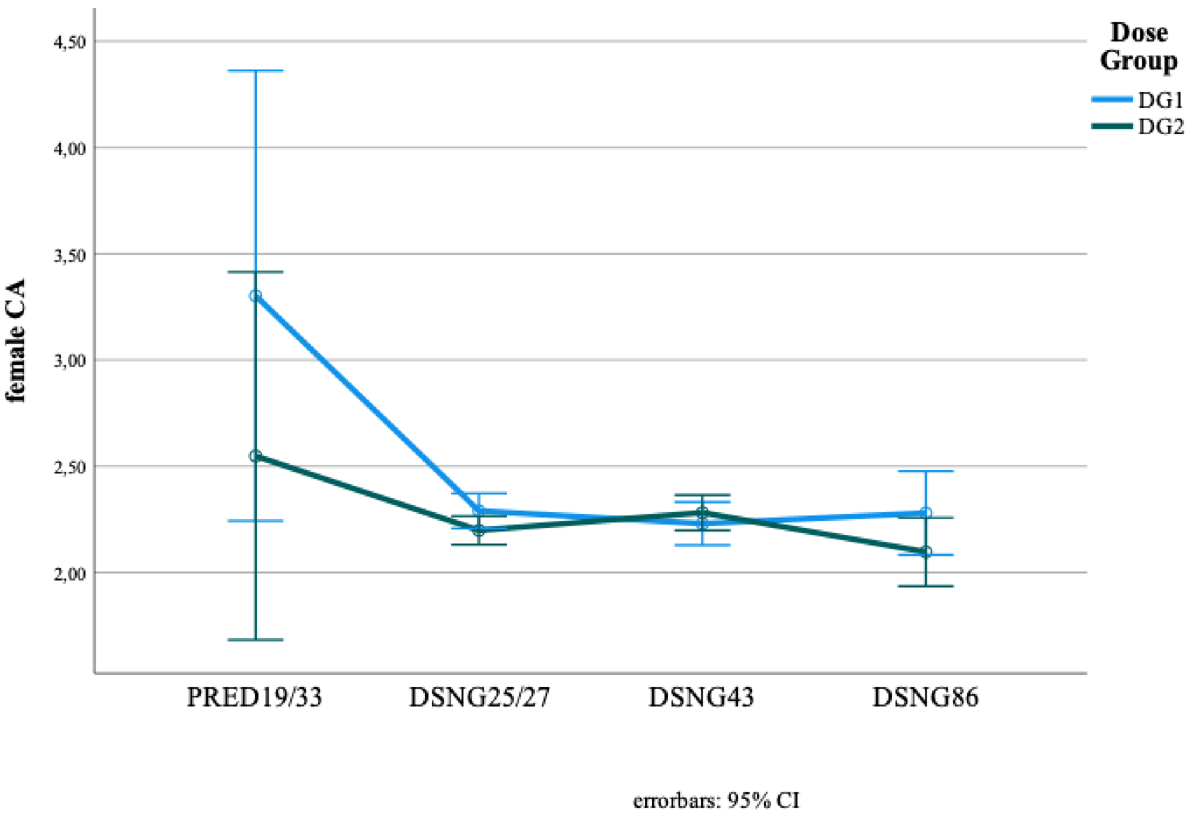

TP female

Im Kontext der Unterschungen des TP zeigte sich mittels des Mauchly-Tests ein signifikantes Ergebnis, $\chi^2(5) = 13{,}47, p = 0{,}021.$ Somit ist die Annahme der Sphärizität verletzt. Es wird zur Berechnung der ANOVA die robuste Untergrenze verwendet.

Mauchly-Test auf Sphärizität

Maß: MASS_1

Innersubjekteffekt	Mauchly-W	Ungefähres Chi-Quadrat	df	Sig.	Epsilon Greenhouse-Geisser	Epsilon Huynh-Feldt (HF)	Epsilon Untergrenze
Zeit	,135	13,470	5	,021	,690	1,000	,333

Dabei zeigt sich ein signifikanter Haupteffekt Zeit, $F(1,8) = 95{,}98, p = 0{,}000, \eta^2 = 0{,}92$. Der Effekt ist stark. Weiterhin ist der Interaktionseffekt nicht-signifikant, $F(1,\ 8) = 2{,}09, p = 0{,}186, \eta^2 = 0{,}21.$ Der Effekt ist ebenfalls stark.

Tests der Innersubjekteffekte

Maß: MASS_1

Quelle		Typ III Quadratsumme	df	Mittel der Quadrate	F	Sig.	Partielles Eta-Quadrat
Zeit	Sphärizität angenommen	2086,106	3	695,369	95,977	,000	,923

	Greenhouse-Geisser	2086,106	2,069	1008,469	95,977	,000	,923
	Huynh-Feldt (HF)	2086,106	3,000	695,369	95,977	,000	,923
	Untergrenze	2086,106	1,000	2086,106	95,977	,000	,923
Zeit * Group	Sphärizität angenommen	45,462	3	15,154	2,092	,128	,207
	Greenhouse-Geisser	45,462	2,069	21,977	2,092	,154	,207
	Huynh-Feldt (HF)	45,462	3,000	15,154	2,092	,128	,207
	Untergrenze	45,462	1,000	45,462	2,092	,186	,207
Fehler(Zeit)	Sphärizität angenommen	173,883	24	7,245			
	Greenhouse-Geisser	173,883	16,549	10,507			
	Huynh-Feldt (HF)	173,883	24,000	7,245			
	Untergrenze	173,883	8,000	21,735			

Zwischen den Gruppen konnten keine signifikanten Unterschiede nachgewiesen werden, $F(1,8) = 3{,}75, p = 0{,}089, \eta^2 = 0{,}32$. Der Effekt ist stark.

Tests der Zwischensubjekteffekte

Maß: MASS_1

Transformierte Variable: Mittel

Quelle	Typ III Quadratsumme	df	Mittel der Quadrate	F	Sig.	Partielles Eta-Quadrat
Konstanter Term	148655,526	1	148655,526	4603,254	,000	,998
Group	120,970	1	120,970	3,746	,089	,319
Fehler	258,349	8	32,294			

Da der Haupteffekt Zeit signifikant war, werden die Unterschiede zwischen den Zeitpunkten innerhalb einer Post-Hoc-Analyse untersucht. Hierbei zeigt sich, dass sich PRED19/33 sich jeweils signifikant von DSNG25/27, DSNG43 und DSNG86 unterscheidet. Weiterhin unterscheiden sich DSNG43 und DSNG86 signifikant voneinander.

Paarweise Vergleiche

Maß: MASS_1

(I) Zeit	(J) Zeit	Mittelwertdifferenz (I-J)	Std.-Fehler	Sig.	95% Konfidenzintervall für Differenz: Untergrenze	95% Konfidenzintervall für Differenz: Obergrenze
1	2	15,823	1,351	,000	11,121	20,524
	3	11,093	1,424	,000	6,138	16,047
	4	19,641	,593	,000	17,579	21,703
2	1	-15,823	1,351	,000	-20,524	-11,121
	3	-4,730	1,503	,082	-9,960	,500
	4	3,819	1,304	,114	-,719	8,356
3	1	-11,093	1,424	,000	-16,047	-6,138
	2	4,730	1,503	,082	-,500	9,960

	4	8,549	,943	,000	5,269	11,829
4	1	-19,641	,593	,000	-21,703	-17,579
	2	-3,819	1,304	,114	-8,356	,719
	3	-8,549	,943	,000	-11,829	-5,269

In kommender Grafik können die Entwicklungen der Mittelwerte über die Zeit betrachtet werden. Es zeigt sich, dass den gesamten Versuch über die Mittelwerte von DG2 höher sind als von DG2. Weiterhin werden die Mittelwerte in beiden Gruppen über den Versuch kleiner. Die Verläufe sind in beiden Gruppen ähnlich.

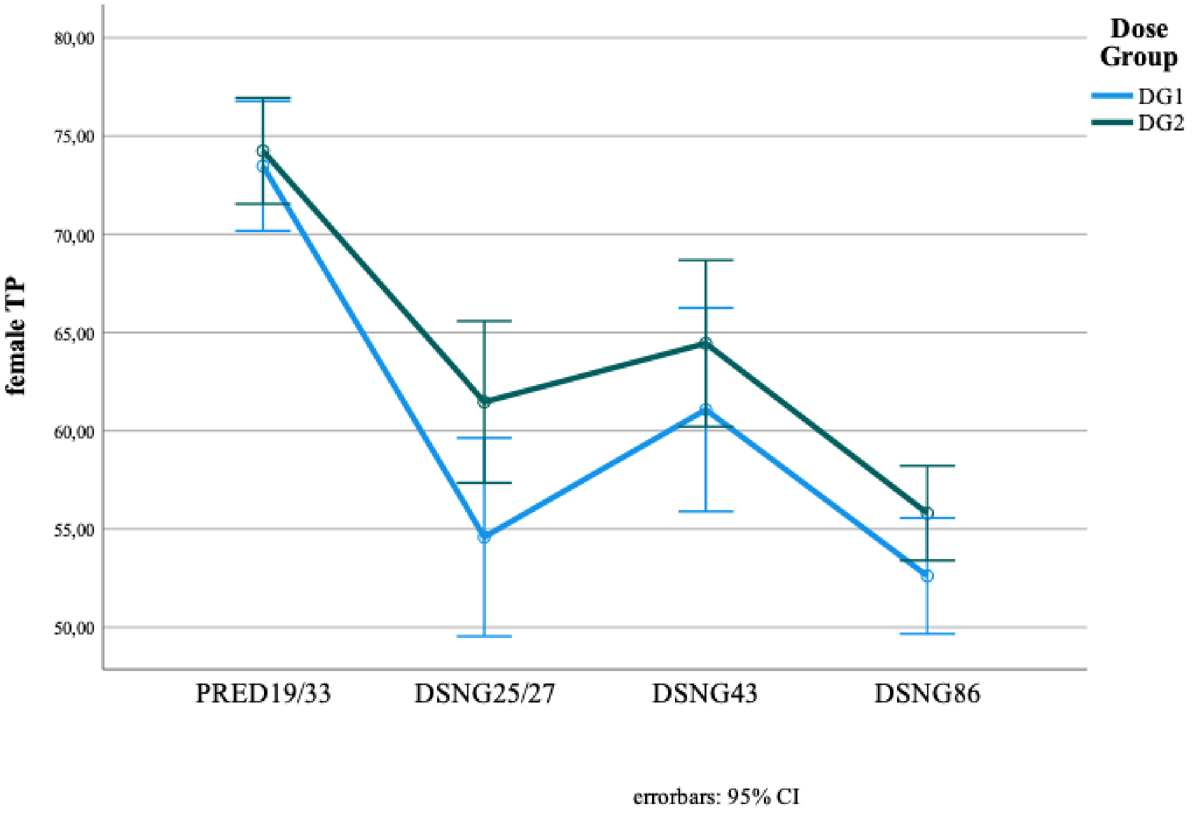

ALB female

Im Kontext von ALB zeigt sich ein nicht-signifikantes Ergebnis beim Mauchly-Test, $\chi^2(5) = 6{,}91, p = 0{,}232$. Damit ist die Annahme der Sphärizität erfüllt.

Mauchly-Test auf Sphärizität

Maß: MASS_1

Innersubjekteffekt	Mauchly-W	Ungefähres Chi-Quadrat	df	Sig.	Epsilon Greenhouse-Geisser	Epsilon Huynh-Feldt (HF)	Epsilon Untergrenze
Zeit	,358	6,910	5	,232	,724	1,000	,333

Es zeigt sich bei der ANOVA ein signifikanter Haupteffekt Zeit, $F(3, 24) = 302{,}80, p = 0{,}000, \eta^2 = 0{,}97$. Der

Effekt ist stark. Die Interaktion übt demgegenüber einen nicht-signifikanten Einfluss auf ALB aus, $F(3, 24) = 0{,}65, p = 0{,}591, \eta^2 = 0{,}08$. Der Effekt ist mittelstark.

Tests der Innersubjekteffekte

Maß: MASS_1

Quelle		Typ III Quadratsumme	df	Mittel der Quadrate	F	Sig.	Partielles Eta-Quadrat
Zeit	Sphärizität angenommen	1461,062	3	487,021	302,802	,000	,974
	Greenhouse-Geisser	1461,062	2,171	672,840	302,802	,000	,974
	Huynh-Feldt (HF)	1461,062	3,000	487,021	302,802	,000	,974
	Untergrenze	1461,062	1,000	1461,062	302,802	,000	,974
Zeit * Group	Sphärizität angenommen	3,131	3	1,044	,649	,591	,075
	Greenhouse-Geisser	3,131	2,171	1,442	,649	,547	,075
	Huynh-Feldt (HF)	3,131	3,000	1,044	,649	,591	,075
	Untergrenze	3,131	1,000	3,131	,649	,444	,075
Fehler(Zeit)	Sphärizität angenommen	38,601	24	1,608			
	Greenhouse-Geisser	38,601	17,372	2,222			
	Huynh-Feldt (HF)	38,601	24,000	1,608			
	Untergrenze	38,601	8,000	4,825			

Zwischen den Gruppen konnten keine signifikanten Unterschiede nachgewiesen werden, $F(1, 8) = 1{,}74, p = 0{,}224, \eta^2 = 0{,}18$. Der Effekt ist stark.

Tests der Zwischensubjekteffekte

Maß: MASS_1

Transformierte Variable: Mittel

Quelle	Typ III Quadratsumme	df	Mittel der Quadrate	F	Sig.	Partielles Eta-Quadrat
Konstanter Term	35034,900	1	35034,900	5050,223	,000	,998
Group	12,060	1	12,060	1,738	,224	,179
Fehler	55,498	8	6,937			

Da der Haupteffekt Zeit signifikant war, werden die Unterschiede zwischen den Zeitpunkten innerhalb einer Post-Hoc-Analyse untersucht. Hierbei zeigt sich, dass sich PRED19/33 sich jeweils signifikant von DSNG25/27, DSNG43 und DSNG86 unterscheidet.

Paarweise Vergleiche

Maß: MASS_1

(I) Zeit	(J) Zeit	Mittelwertdifferenz (I-J)	Std.-Fehler	Sig.	95% Konfidenzintervall für Differenz Untergrenze	Obergrenze
1	2	13,468	,713	,000	10,988	15,949
	3	14,082	,586	,000	12,045	16,119
	4	15,015	,299	,000	13,976	16,053
2	1	-13,468	,713	,000	-15,949	-10,988
	3	,614	,697	1,000	-1,811	3,038
	4	1,546	,572	,162	-,444	3,536
3	1	-14,082	,586	,000	-16,119	-12,045
	2	-,614	,697	1,000	-3,038	1,811
	4	,932	,507	,619	-,831	2,696
4	1	-15,015	,299	,000	-16,053	-13,976
	2	-1,546	,572	,162	-3,536	,444
	3	-,932	,507	,619	-2,696	,831

Kommende Grafik zeigt die Veränderung der Mittelwert zwischen den Gruppen über die Zeit. Die Gruppe DG2 hat dabei über den gesamten Zeitraum höhere Mittelwerte als DG1. In beiden Gruppen ist ein Abfall zu erkennen. Wobei der Verlauf der Mittelwerte zwischen den Gruppen sich sehr ähnelt.

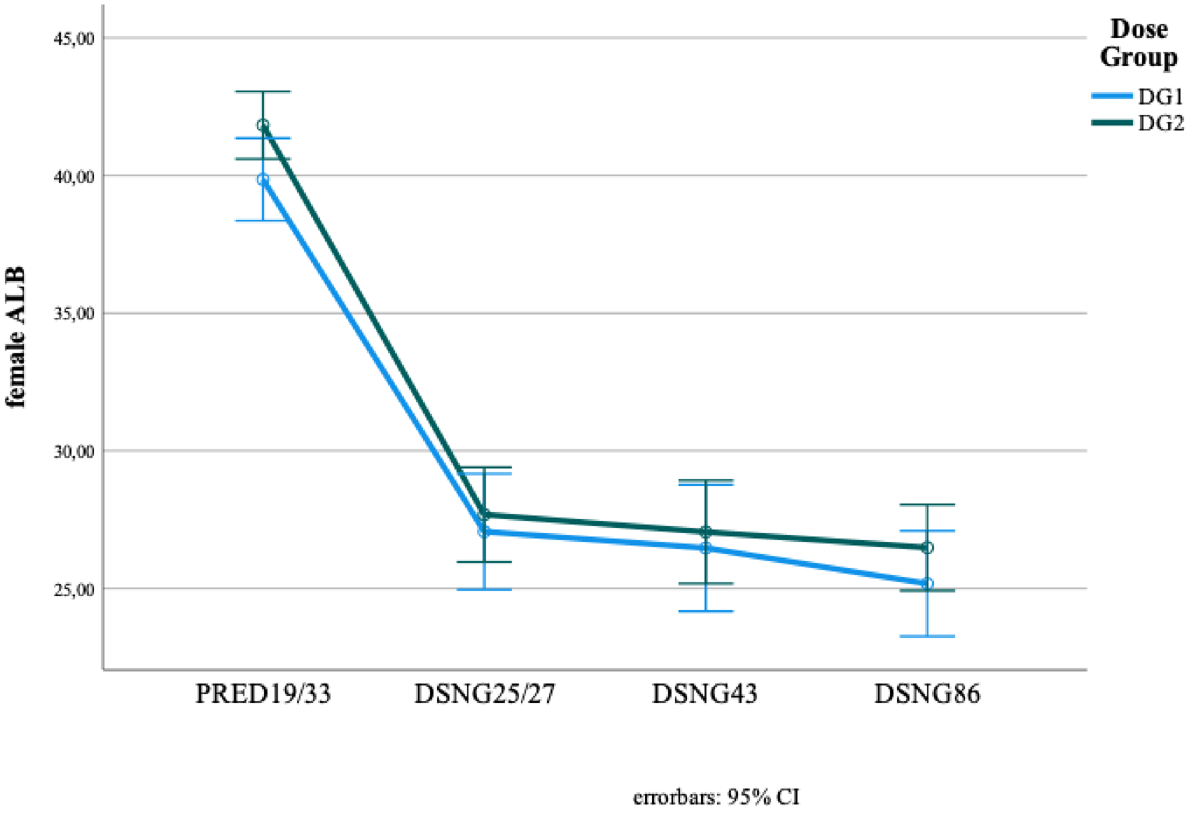

A:G female

Im Falle des Parameter A:G zeigt sich ein nicht-signifikantes Ergebnis bei dem Mauchly-Test, $\chi^2(5) = 10{,}73, p = 0{,}059$. Damit ist die Annahme der Sphärizität erfüllt.

Mauchly-Test auf Sphärizität

Maß: MASS_1

Innersubjekteffekt	Mauchly-W	Ungefähres Chi-Quadrat	df	Sig.	Epsilon: Greenhouse-Geisser	Epsilon: Huynh-Feldt (HF)	Epsilon: Untergrenze
Zeit	,203	10,729	5	,059	,548	,757	,333

Dabei zeigt sich in der ANOVA ein signifikanter Haupteffekt Zeit, $F(3, 24) = 101{,}11, p = 0{,}000, \eta^2 = 0{,}93$. Der Effekt ist stark. Weiterhin übt die Interaktion ebenfalls einen signifikanten Effekt auf A:G aus, $F(3, 24) = 7{,}39, p = 0{,}001, \eta^2 = 0{,}48$. Der Effekt ist stark.

Tests der Innersubjekteffekte

Maß: MASS_1

Quelle		Typ III Quadratsumme	df	Mittel der Quadrate	F	Sig.	Partielles Eta-Quadrat
Zeit	Sphärizität angenommen	1,267	3	,422	101,106	,000	,927
	Greenhouse-Geisser	1,267	1,644	,771	101,106	,000	,927
	Huynh-Feldt (HF)	1,267	2,272	,558	101,106	,000	,927
	Untergrenze	1,267	1,000	1,267	101,106	,000	,927
Zeit * Group	Sphärizität angenommen	,093	3	,031	7,394	,001	,480
	Greenhouse-Geisser	,093	1,644	,056	7,394	,009	,480
	Huynh-Feldt (HF)	,093	2,272	,041	7,394	,003	,480
	Untergrenze	,093	1,000	,093	7,394	,026	,480
Fehler(Zeit)	Sphärizität angenommen	,100	24	,004			
	Greenhouse-Geisser	,100	13,152	,008			
	Huynh-Feldt (HF)	,100	18,175	,006			
	Untergrenze	,100	8,000	,013			

Zwischen den Gruppen liegen dabei keine signifikanten Unterschiede vor, $F(1, 8) = 0{,}46, p = 0{,}518, \eta^2 = 0{,}05$. Der Effekt ist mittelstark.

Tests der Zwischensubjekteffekte

Maß: MASS_1

Transformierte Variable: Mittel

Quelle	Typ III Quadratsumme	df	Mittel der Quadrate	F	Sig.	Partielles Eta-Quadrat
Konstanter Term	34,816	1	34,816	2296,059	,000	,997
Group	,007	1	,007	,457	,518	,054
Fehler	,121	8	,015			

Da der Haupteffekt Zeit signifikant war, werden die einzelnen Zeitpunkte in einer Post-Hoc-Analyse miteinander verglichen. Folgende paarweisen Vergleiche waren dabei signifikant, vergleiche die kommende Tabelle: PRED19/33 vs. DSNG25/27, PRED19/33 vs. DSNG43, PRED19/33 vs. DSNG86, DSNG25/27 vs. DSNG43 und DSNG43 vs. DSNG86.

Paarweise Vergleiche

Maß: MASS_1

(I) Zeit	(J) Zeit	Mittelwertdifferenz (I-J)	Std.-Fehler	Sig.	95% Konfidenzintervall für Differenz: Untergrenze	95% Konfidenzintervall für Differenz: Obergrenze
1	2	,340	,041	,000	,198	,482
	3	,499	,037	,000	,370	,628
	4	,332	,032	,000	,222	,442
2	1	-,340	,041	,000	-,482	-,198
	3	,158	,016	,000	,103	,214
	4	-,008	,019	1,000	-,075	,058
3	1	-,499	,037	,000	-,628	-,370
	2	-,158	,016	,000	-,214	-,103
	4	-,167	,024	,001	-,249	-,084
4	1	-,332	,032	,000	-,442	-,222
	2	,008	,019	1,000	-,058	,075
	3	,167	,024	,001	,084	,249

Kommende Grafik zeigt die Mittelwerte. Es zeigt sich, dass zu Beginn der Mittelwert der DG2 höher ist als der der

DG1. Ab DSNG25/27 liegt der Mittelwert von DG2 unterhalb von DG1, ehe sie sich zu DSNG86 angleichen.

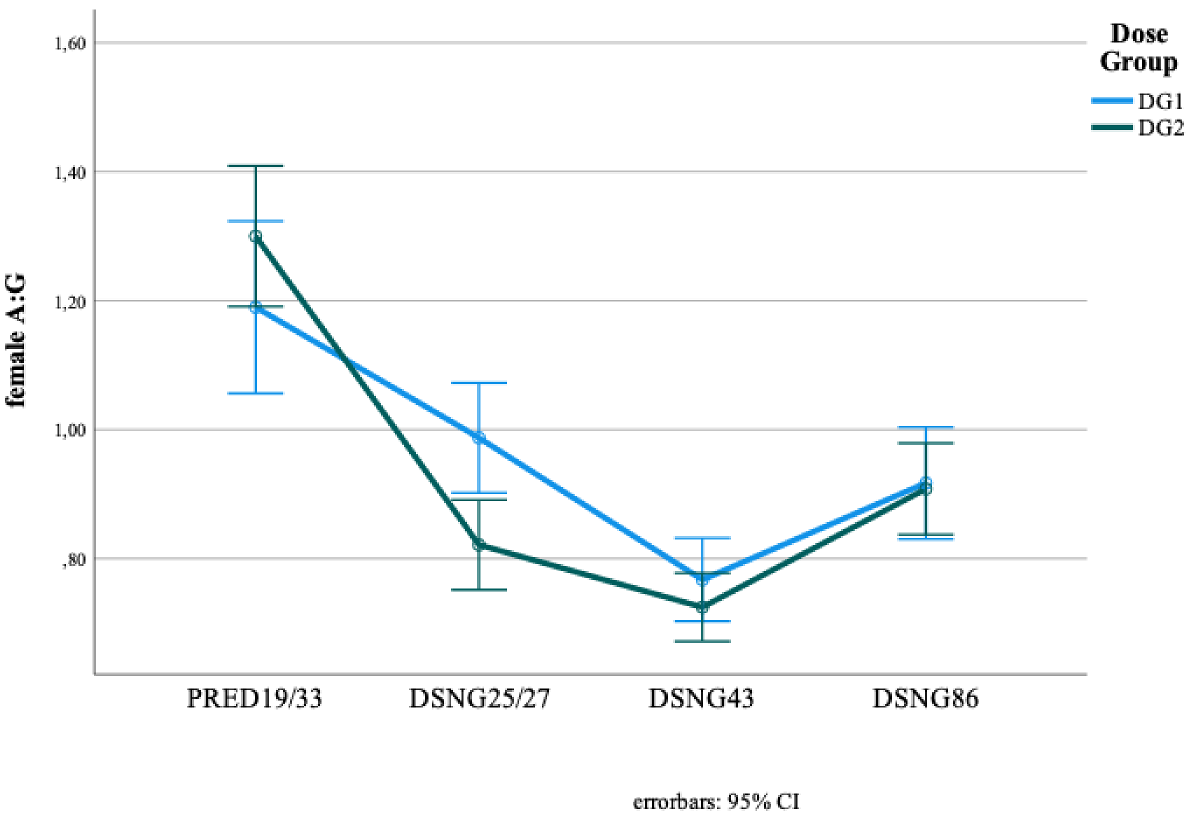

Anhang

Studie A

TP female

Tests auf Normalverteilung

	Kolmogorov-Smirnov			Shapiro-Wilk		
	Statistik	df	Signifikanz	Statistik	df	Signifikanz
Standardisiertes Residuum für PRED09	,167	14	,200	,957	14	,666
Standardisiertes Residuum für DSNG176	,122	14	,200	,984	14	,992

TRIG male

Tests auf Normalverteilung

	Kolmogorov-Smirnov[a]			Shapiro-Wilk		
	Statistik	df	Signifikanz	Statistik	df	Signifikanz
Standardisiertes Residuum für PRED08	,163	14	,200*	,953	14	,604
Standardisiertes Residuum für DSNG90	,197	14	,147	,965	14	,807
Standardisiertes Residuum für DSNG181	,147	14	,200*	,955	14	,640

*. Dies ist eine untere Grenze der echten Signifikanz.

a. Signifikanzkorrektur nach Lilliefors

Studie E

GLOB male

Tests auf Normalverteilung

	Kolmogorov-Smirnov			Shapiro-Wilk		
	Statistik	df	Signifikanz	Statistik	df	Signifikanz
Standardisiertes Residuum für PRED15	,234	5	,200	,845	5	,178
Standardisiertes Residuum für DSNG15	,193	5	,200	,918	5	,515
Standardisiertes Residuum für DSNG32	,165	5	,200	,964	5	,833
Standardisiertes Residuum für DSNG60	,223	5	,200	,903	5	,424
Standardisiertes Residuum für DSNG116	,208	5	,200	,969	5	,866
Standardisiertes Residuum für DSNG186	,189	5	,200	,981	5	,941

A:G male

Tests auf Normalverteilung

	Kolmogorov-Smirnov			Shapiro-Wilk		
	Statistik	df	Signifikanz	Statistik	df	Signifikanz
Standardisiertes Residuum für PRED15_AG	,314	5	,121	,827	5	,132
Standardisiertes Residuum für DSNG15_AG	,234	5	,200	,944	5	,692
Standardisiertes Residuum für DSNG32_AG	,243	5	,200	,941	5	,673
Standardisiertes Residuum für DSNG60_AG	,268	5	,200	,930	5	,599
Standardisiertes Residuum für DSNG116_AG	,316	5	,114	,828	5	,135
Standardisiertes Residuum für DSNG186_AG	,256	5	,200	,851	5	,197

TP female

Tests auf Normalverteilung

	Kolmogorov-Smirnov			Shapiro-Wilk		
	Statistik	df	Signifikanz	Statistik	df	Signifikanz
Standardisiertes Residuum für PRED78_TP	,252	6	,200	,836	6	,121
Standardisiertes Residuum für DSNG15_TP	,166	6	,200	,961	6	,826
Standardisiertes Residuum für DSNG32_TP	,264	6	,200	,945	6	,699
Standardisiertes Residuum für DSNG60_TP	,198	6	,200	,979	6	,945
Standardisiertes Residuum für DSNG116_TP	,278	6	,162	,929	6	,570
Standardisiertes Residuum für DSNG186_TP	,257	6	,200	,882	6	,277

Studie H

Tests auf Normalverteilung

	Kolmogorov-Smirnov			Shapiro-Wilk		
	Statistik	df	Signifikanz	Statistik	df	Signifikanz
Standardisiertes Residuum für PRED17	,218	18	,023	,839	18	,006

Standardisiertes Residuum für DSNG22	,097	18	,200	,968	18	,755
Standardisiertes Residuum für DSNG83	,148	18	,200	,948	18	,390
Standardisiertes Residuum für DSNG174	,222	18	,019	,904	18	,069

Studie L

GLOB female

Tests auf Normalverteilung

	Kolmogorov-Smirnov			Shapiro-Wilk		
	Statistik	df	Signifikanz	Statistik	df	Signifikanz
Standardisiertes Residuum für PRED21_GLOB	,202	14	,124	,925	14	,256
Standardisiertes Residuum für DSNG57_GLOB	,134	14	,200	,940	14	,416
Standardisiertes Residuum für DSNG85_GLOB	,178	14	,200	,881	14	,061
Standardisiertes Residuum für DSNG141_GLOB	,098	14	,200	,973	14	,919
Standardisiertes Residuum für DSNG197_GLOB	,183	14	,200	,922	14	,233
Standardisiertes Residuum für DSNG253_GLOB	,151	14	,200	,946	14	,494
Standardisiertes Residuum für DSNG281_GLOB	,099	14	,200	,965	14	,801

TP female

Tests auf Normalverteilung

	Kolmogorov-Smirnov			Shapiro-Wilk		
	Statistik	df	Signifikanz	Statistik	df	Signifikanz
Standardisiertes Residuum für PRED21_TP	,205	14	,113	,858	14	,028
Standardisiertes Residuum für DSNG57_TP	,150	14	,200	,961	14	,734
Standardisiertes Residuum für DSNG85_TP	,168	14	,200	,939	14	,404
Standardisiertes Residuum für DSNG141_TP	,098	14	,200	,958	14	,689
Standardisiertes Residuum für DSNG197_TP	,149	14	,200	,887	14	,073

Standardisiertes Residuum für DSNG253_TP	,136	14	,200	,944	14	,477
Standardisiertes Residuum für DSNG281_TP	,186	14	,200	,939	14	,412

Studie M
IGG male

Tests auf Normalverteilung

	Kolmogorov-Smirnov			Shapiro-Wilk		
	Statistik	df	Signifikanz	Statistik	df	Signifikanz
Standardisiertes Residuum für PRED50_IgG	,211	6	,200	,965	6	,856
Standardisiertes Residuum für DSNG31_IgG	,248	6	,200	,928	6	,566
Standardisiertes Residuum für DSNG86_IgG	,204	6	,200	,950	6	,742
Standardisiertes Residuum für DSNG136_IgG	,197	6	,200	,958	6	,802
Standardisiertes Residuum für DSNG176_IgG	,173	6	,200	,979	6	,948

TP male

Tests auf Normalverteilung

	Kolmogorov-Smirnov			Shapiro-Wilk		
	Statistik	df	Signifikanz	Statistik	df	Signifikanz
Standardisiertes Residuum für PRED50_TP	,272	6	,187	,868	6	,218
Standardisiertes Residuum für DSNG31_TP	,211	6	,200	,913	6	,459
Standardisiertes Residuum für DSNG86_TP	,215	6	,200	,904	6	,397
Standardisiertes Residuum für DSNG136_TP	,226	6	,200	,906	6	,408
Standardisiertes Residuum für DSNG176_TP	,227	6	,200	,888	6	,307

GLOB male

Tests auf Normalverteilung

	Kolmogorov-Smirnov			Shapiro-Wilk		
	Statistik	df	Signifikanz	Statistik	df	Signifikanz
Standardisiertes Residuum für PRED50_GLOB	,216	6	,200	,934	6	,613
Standardisiertes Residuum für DSNG31_GLOB	,199	6	,200	,978	6	,940
Standardisiertes Residuum für DSNG86_GLOB	,184	6	,200	,975	6	,923
Standardisiertes Residuum für DSNG136_GLOB	,210	6	,200	,897	6	,358
Standardisiertes Residuum für DSNG176_GLOB	,158	6	,200	,965	6	,860

ALB male

Tests auf Normalverteilung

	Kolmogorov-Smirnov			Shapiro-Wilk		
	Statistik	df	Signifikanz	Statistik	df	Signifikanz
Standardisiertes Residuum für PRED50_ALB	,286	6	,137	,813	6	,077
Standardisiertes Residuum für DSNG31_ALB	,202	6	,200	,963	6	,844
Standardisiertes Residuum für DSNG86_ALB	,157	6	,200	,983	6	,964
Standardisiertes Residuum für DSNG136_ALB	,358	6	,016	,832	6	,112
Standardisiertes Residuum für DSNG176_ALB	,211	6	,200	,965	6	,856

CHOL male

Tests auf Normalverteilung

	Kolmogorov-Smirnov			Shapiro-Wilk		
	Statistik	df	Signifikanz	Statistik	df	Signifikanz
Standardisiertes Residuum für PRED50_CHOL	,262	6	,200	,844	6	,141
Standardisiertes Residuum für DSNG31_CHOL	,196	6	,200	,902	6	,383
Standardisiertes Residuum für DSNG86_CHOL	,386	6	,006	,725	6	,011
Standardisiertes Residuum für DSNG136_CHOL	,248	6	,200	,930	6	,582

Standardisiertes Residuum für DSNG176_CHOL	,323	6	,050	,731	6	,013

Studie P

ALT female

Tests auf Normalverteilung

	Kolmogorov-Smirnov			Shapiro-Wilk		
	Statistik	df	Signifikanz	Statistik	df	Signifikanz
Standardisiertes Residuum für PRED08_ALT	,128	14	,200	,964	14	,789
Standardisiertes Residuum für DSNG44_ALT	,171	14	,200	,932	14	,324
Standardisiertes Residuum für DSNG55_ALT	,403	14	,000	,597	14	,000
Standardisiertes Residuum für DSNG65_ALT	,149	14	,200	,943	14	,455
Standardisiertes Residuum für DSNG93_ALT	,101	14	,200	,964	14	,795
Standardisiertes Residuum für DSNG135_ALT	,156	14	,200	,936	14	,370
Standardisiertes Residuum für DSNG184_ALT	,131	14	,200	,938	14	,390
Standardisiertes Residuum für DSNG275_ALT	,164	14	,200	,917	14	,196

GDLH female

Tests auf Normalverteilung

	Kolmogorov-Smirnov			Shapiro-Wilk		
	Statistik	df	Signifikanz	Statistik	df	Signifikanz
Standardisiertes Residuum für PRED08_GLDH	,118	14	,200	,972	14	,907
Standardisiertes Residuum für DSNG44_GLDH	,148	14	,200	,947	14	,519
Standardisiertes Residuum für DSNG55_GLDH	,393	14	,000	,611	14	,000
Standardisiertes Residuum für DSNG65_GLDH	,253	14	,015	,770	14	,002
Standardisiertes Residuum für DSNG93_GLDH	,141	14	,200	,945	14	,487
Standardisiertes Residuum für DSNG135_GLDH	,241	14	,027	,853	14	,025
Standardisiertes Residuum für DSNG184_GLDH	,216	14	,076	,906	14	,138

Standardisiertes Residuum für DSNG275_GLDH	,161	14	,200	,912	14	,170

Studie Q

AST female

Tests auf Normalverteilung

	Kolmogorov-Smirnov			Shapiro-Wilk		
	Statistik	df	Signifikanz	Statistik	df	Signifikanz
Standardisiertes Residuum für PRED08	,241	14	,027	,866	14	,037
Standardisiertes Residuum für DSNG44	,142	14	,200	,972	14	,908

GGT female

Tests auf Normalverteilung

	Kolmogorov-Smirnov			Shapiro-Wilk		
	Statistik	df	Signifikanz	Statistik	df	Signifikanz
Standardisiertes Residuum für PRED08_GGT	,144	14	,200	,966	14	,823
Standardisiertes Residuum für DSNG44_GGT	,313	14	,001	,717	14	,001

Studie R

Tests auf Normalverteilung

	Kolmogorov-Smirnov			Shapiro-Wilk		
	Statistik	df	Signifikanz	Statistik	df	Signifikanz
Standardisiertes Residuum für PRED71	,124	12	,200	,960	12	,791
Standardisiertes Residuum für DSNG85	,417	12	,000	,561	12	,000

Studie S

IgG female

Tests auf Normalverteilung

	Kolmogorov-Smirnov	Shapiro-Wilk

	Statistik	df	Signifikanz	Statistik	df	Signifikanz
Standardisiertes Residuum für PRED1933_IGG	,175	10	,200	,951	10	,685
Standardisiertes Residuum für DSNG2527_IGG	,197	10	,200	,920	10	,361
Standardisiertes Residuum für DSNG43_IGG	,227	10	,152	,880	10	,132
Standardisiertes Residuum für DSNG86_IGG	,200	10	,200	,916	10	,327

CA female

Tests auf Normalverteilung

	Kolmogorov-Smirnov			Shapiro-Wilk		
	Statistik	df	Signifikanz	Statistik	df	Signifikanz
Standardisiertes Residuum für PRED1933_CA	,331	10	,003	,743	10	,003
Standardisiertes Residuum für DSNG2527_CA	,182	10	,200	,927	10	,417
Standardisiertes Residuum für DSNG43_CA	,148	10	,200	,970	10	,895
Standardisiertes Residuum für DSNG86_CA	,140	10	,200	,930	10	,447

TP female

Tests auf Normalverteilung

	Kolmogorov-Smirnov			Shapiro-Wilk		
	Statistik	df	Signifikanz	Statistik	df	Signifikanz
Standardisiertes Residuum für PRED1933_TP	,131	10	,200	,973	10	,920
Standardisiertes Residuum für DSNG2527_TP	,154	10	,200	,947	10	,628
Standardisiertes Residuum für DSNG43_TP	,210	10	,200	,889	10	,165
Standardisiertes Residuum für DSNG86_TP	,174	10	,200	,908	10	,266

ALB female

Tests auf Normalverteilung

	Kolmogorov-Smirnov			Shapiro-Wilk		
	Statistik	df	Signifikanz	Statistik	df	Signifikanz
Standardisiertes Residuum für PRED1933_ALB	,153	10	,200	,981	10	,969
Standardisiertes Residuum für DSNG2527_ALB	,184	10	,200	,903	10	,237
Standardisiertes Residuum für DSNG43_ALB	,132	10	,200	,957	10	,755
Standardisiertes Residuum für DSNG86_ALB	,171	10	,200	,973	10	,914

A:G female

Tests auf Normalverteilung

	Kolmogorov-Smirnov			Shapiro-Wilk		
	Statistik	df	Signifikanz	Statistik	df	Signifikanz
Standardisiertes Residuum für PRED1933_AG	,236	10	,120	,930	10	,448
Standardisiertes Residuum für DSNG2527_AG	,179	10	,200	,973	10	,919
Standardisiertes Residuum für DSNG43_AG	,282	10	,023	,797	10	,013
Standardisiertes Residuum für DSNG86_AG	,225	10	,162	,864	10	,085
